# Synthesis and Catalytic Activity of Bifunctional Phase-Transfer Organocatalysts Based on Camphor

**DOI:** 10.3390/molecules28031515

**Published:** 2023-02-03

**Authors:** Luka Ciber, Franc Požgan, Helena Brodnik, Bogdan Štefane, Jurij Svete, Mario Waser, Uroš Grošelj

**Affiliations:** 1 Chair of Organic Chemistry, Faculty of Chemistry and Chemical Technology, University of Ljubljana, Večna pot 113, 1000 Ljubljana, Slovenia; 2 Institute of Organic Chemistry, Johannes Kepler University Linz, Altenbergerstrasse 69, 4040 Linz, Austria

**Keywords:** asymmetric organocatalysis, quaternary ammonium salts, phase-transfer catalysts (PTCs), camphor, camphor-derived diamines, β-keto esters, enantioselective α-fluorination, electrophilic α-chlorination, asymmetric Michael addition

## Abstract

Ten novel bifunctional quaternary ammonium salt phase-transfer organocatalysts were synthesized in four steps from (+)-camphor-derived 1,3-diamines. These quaternary ammonium salts contained either (thio)urea or squaramide hydrogen bond donor groups in combination with either trifluoroacetate or iodide as the counteranion. Their organocatalytic activity was evaluated in electrophilic heterofunctionalizations of β-keto esters and in the Michael addition of a glycine Schiff base with methyl acrylate. α-Fluorination and chlorination of β-keto esters proceeded with full conversion and low enantioselectivities (up to 29% ee). Similarly, the Michael addition of a glycine Schiff base with methyl acrylate proceeded with full conversion and up to 11% ee. The new catalysts have been fully characterized; the stereochemistry at the C-2 chiral center was unambiguously determined.

## 1. Introduction

**Figure 1:**
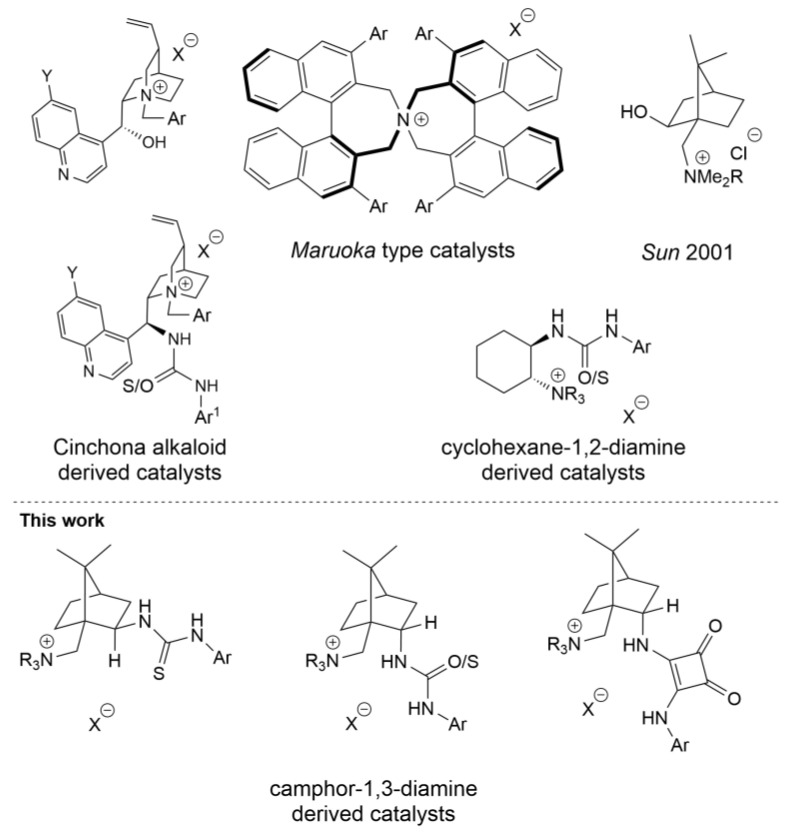
Selected efficient chiral quaternary ammonium salt phase-transfer catalysts (PTCs) and novel bifunctional camphor-based PTCs reported herein [20].

Since the seminal contributions of Wynberg [1], Dolling [2], and O’Donnell [3] in the 1970s and 1980s, in which chiral quaternary ammonium salts based on Cinchona alkaloids were used as catalysts for enantioselective epoxidations and α-alkylations of prochiral substrates, the use of chiral quaternary ammonium salts as phase-transfer catalysts (PTCs) has been successfully demonstrated in a multitude of asymmetric organic transformations and now represents an established fundamental catalysis principle in asymmetric organocatalysis [4,5,6,7,8]. In addition to Cinchona alkaloid-based PTCs, other chiral backbones have also been successfully used to access high-performance catalysts. A group of highly efficient binaphthyl-based ammonium salts was introduced by Maruoka (the so-called Maruoka catalysts) [9,10], which have since established themselves as the second most privileged class of chiral ammonium salt PTCs, alongside Cinchona alkaloids (Figure 1). Over the years, efficient chiral quaternary ammonium salts based on tartaric acid [11,12], α-amino acids [13,14], *trans*-cyclohexane-1,2-diamine [15], and others [16] have been developed. Many of the developed quaternary ammonium salts, especially catalysts based on Cinchona alkaloids and some Maruoka-type catalysts, possess a hydrogen-bonding donor in the form of a OH group, which leads to improved catalytic properties [17]. The incorporation of (thio)urea-containing hydrogen bond donors in catalysts based on Cinchona alkaloids, and, in particular, in catalysts based on amino acids and cyclohexane-1,2-diamine, contributed significantly to the diversification of the available catalysts and extended the scope of catalyzed asymmetric transformations [13,15,18,19].

Camphor is one of nature’s most privileged scaffolds, readily available in both enantiomeric forms. In addition, camphor undergoes a variety of interesting chemical transformations that functionalize, at first sight, inactive positions [21,22], allowing the synthesis of structurally and functionally very different products [23,24,25,26,27], thus making camphor a desirable starting material. The first reports on the application of camphor-derived organocatalysts date back to 2001. Camphor-derived phase-transfer organocatalysts were employed to catalyze the α-alkylation of a glycine Schiff base with enantioselectivities up to 39% ee (Figure 1) [20]. Since then, several types of camphor-based organocatalysts have been reported, exhibiting covalent or noncovalent activation modes, both those with a camphor backbone as the sole chiral fragment and those in which the camphor backbone is covalently linked to a chiral amino acid, usually proline, via a suitable spacer [28].

As part of our ongoing study of camphor-based diamines as potential organocatalyst scaffolds [29], we reported the synthesis of 1,3-diamine-based bifunctional squaramide organocatalysts prepared from camphor and their application as efficient catalysts in Michael additions of 1,3-dicarbonyl compounds and pyrrolones as nucleophiles to *trans*-β-nitrostyrene derivatives [30,31]. Extending this work, we report here the synthesis of a new type of 1,3-diamine-based bifunctional quaternary ammonium salt phase-transfer organocatalyst (Figure 1) and its evaluation in the electrophilic α-functionalization of β-keto ester and the alkylation of a glycine-derived Schiff base with methyl acrylate.

## 2. Results and Discussion

### 2.1. Synthesis

**Scheme 1:**
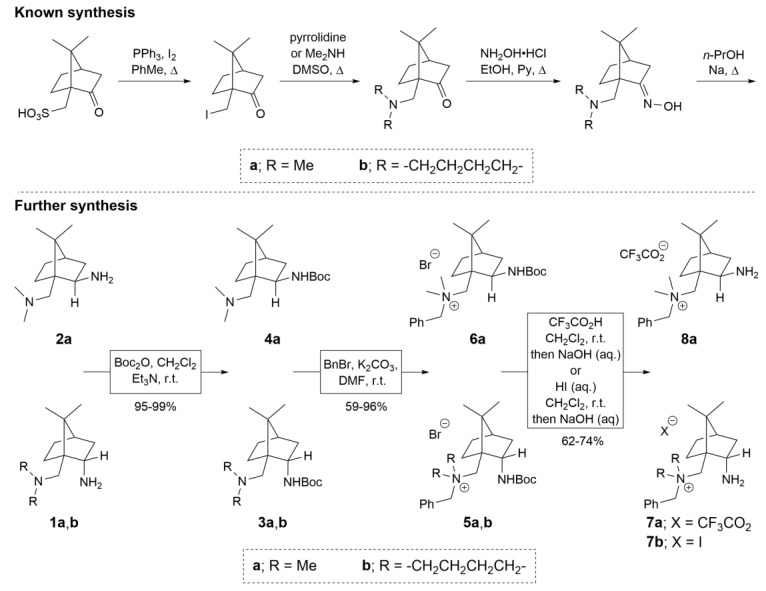
Synthesis of camphor-derived *endo*- **7a**,**b** and *exo*-quaternary ammonium salts **8a**.

Camphor-derived *endo*-diamines **1a**,**b** and *exo*-diamines **2a** were prepared in four steps from commercially available (1*S*)-(+)-10-camphorsulfonic acid (Scheme 1) [29,30]. Camphorsulfonic acid was transformed into 10-iodocamphor in an Apple-type reaction, followed by nucleophilic substitution with pyrrolidine or dimethylamine in dimethyl sulfoxide. The thus formed tertiary amines were transformed into the corresponding oximes. The final oxime reduction with sodium in isopropanol gave a mixture of the corresponding major *endo*-diamines **1a**,**b** and minor *exo*-diamines **2a**, separable by column chromatography.

Next, the primary amino group of diamines **1a**,**b** and **2a** was Boc-protected, yielding **3a**,**b** and **4a**, respectively. In the following step, we introduced the benzyl group to the tertiary amine (benzyl groups have been very successfully established as useful motives for numerous quaternary ammonium salt phase-transfer catalysts [4,5,6,7,8]). Alkylation with benzyl bromide thus gave the quaternary ammonium salts **5a**,**b** and **6a**. Potassium carbonate was added to ensure complete conversion. They were subsequently Boc-deprotected with trifluoroacetic acid or aqueous hydrogen iodide, furnishing ammonium salts **7a**,**b** and **8a**, respectively (Scheme 1).

**Scheme 2:**
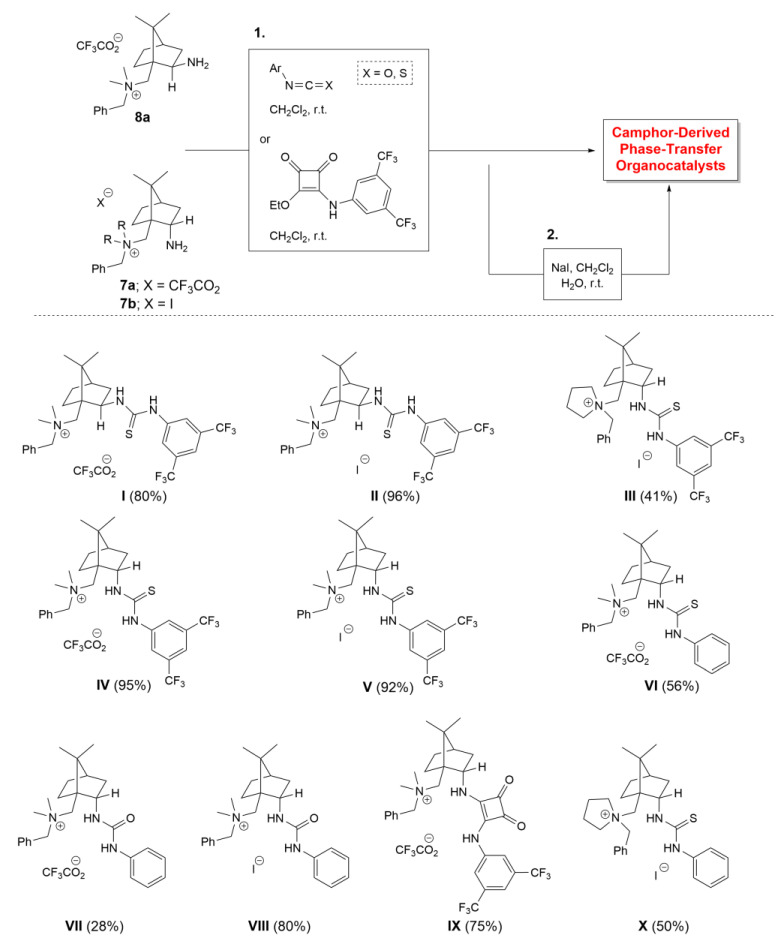
Synthesis of camphor-derived phase-transfer organocatalysts.

Finally, the ammonium salts **7a**,**b** and **8a** were reacted with aromatic iso(thio)cyanates **9** and squaramate **10** to give the quaternary trifluoroacetate ammonium salts **I**, **IV**, **VI**, **VII**, and **IX**. The quaternary iodide ammonium salts **II**, **V**, and **VIII** were formed from the corresponding trifluoroacetates **I**, **IV**, and **VII**, respectively, via anion metathesis with excess NaI in dichloromethane (Scheme 2). The quaternary iodide ammonium salts **III** and **X** were formed directly from the iodide ammonium salt **7b** and the corresponding isothiocyanate. The catalysts thus formed have either (thio)urea or squaramide hydrogen bond donors (Supplementary Materials).

### 2.2. Structure Determination

**Figure 2:**
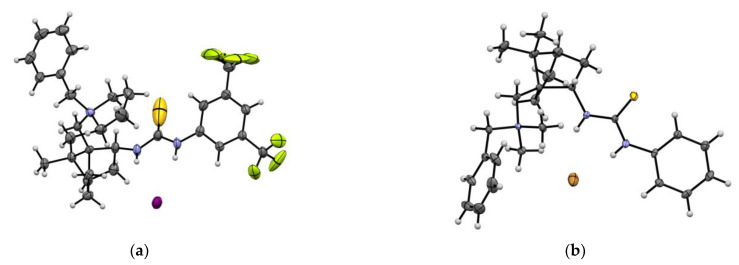
Molecular structures of products **III** (**a**) and **VI-Br** (**b**). Thermal ellipsoids are shown at 50% probability.

**Figure 3:**
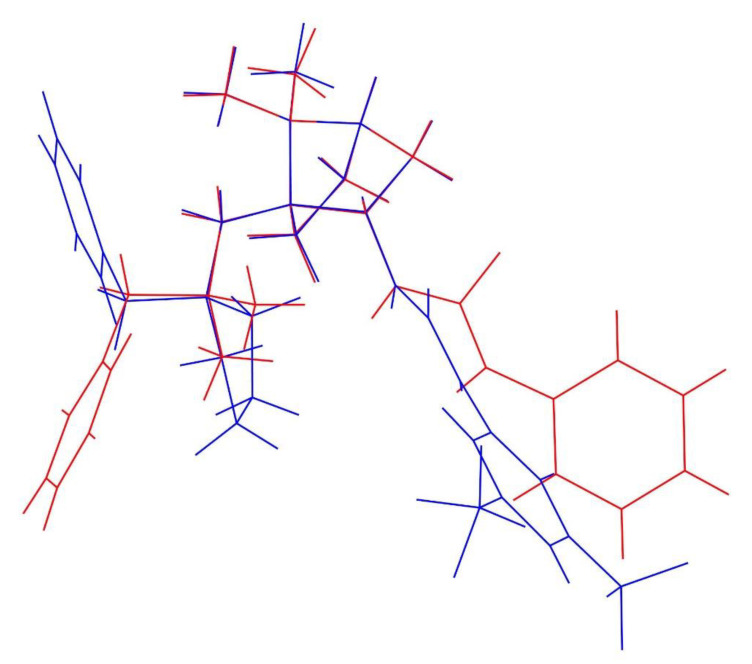
Stick presentation of the superimposed molecular structures of products **III** (blue) and **VI-Br** (red).

The intermediates **3**–**8** were characterized by ^1^H- and ^13^C-NMR, IR, and HRMS. Compounds **1a** and **2a** were characterized by ^1^H-NMR. Phase-transfer catalysts **I**–**X** have been fully characterized. The structures of the thioureas **III** and **VI-Br** (the bromide analog of the compound **VI**) were determined by single-crystal X-ray analysis (Figure 2). In both structures, the *endo*-stereochemistry was confirmed at the C-2 chiral center. The conformational differences in the two structures in the solid state are shown in Figure 3. The main differences are due to the conformation of the benzyl group and the arylthiourea structural elements.

**Figure 4:**
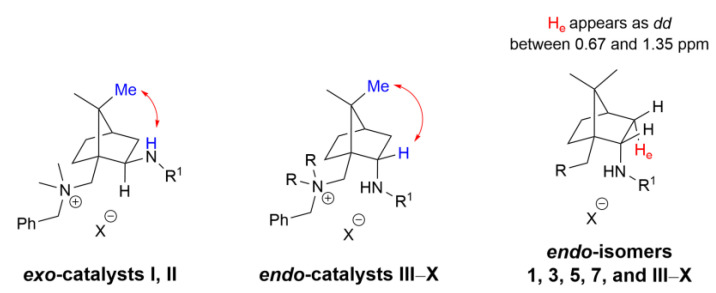
The determination of stereochemistry at the C-2 chiral center of compounds **I**–**X** by NOESY experiments and the correlation between the multiplicity of the H–C(3)-*endo* proton (H_e_) and the *endo* absolute configuration at the C-2 chiral center of compounds **1**, **3**, **5**, **7**, and **III**–**X** (Supplementary Materials).

The *endo*-stereochemistry at the C-2 chiral center of compounds **III**–**X** was further confirmed by NOESY measurements based on the cross-peak between the methyl group and the *exo*-H(2) proton (Figure 4). Similarly, the *exo*-stereochemistry at the C-2 chiral center of compounds **I** and **II** was in line with the cross-peak between the methyl group and the *exo*-H–N proton observed in the NOESY spectra (Supplementary Materials).

Finally, the stereochemistry at the C-2 chiral center can be correlated on the basis of the multiplicity of the H–C(3)-*endo* proton (H_e_) (Figure 4). Exclusively in the *endo*-isomes of compounds **1, 3**, **5**, **7**, and **III**–**X**, the H–C(3)-*endo* proton appears as a doublet of doublet between 0.67 and 1.35 ppm (Table S7 in Supplementary Materials).

### 2.3. Organocatalytic Activity

**Scheme 3:**
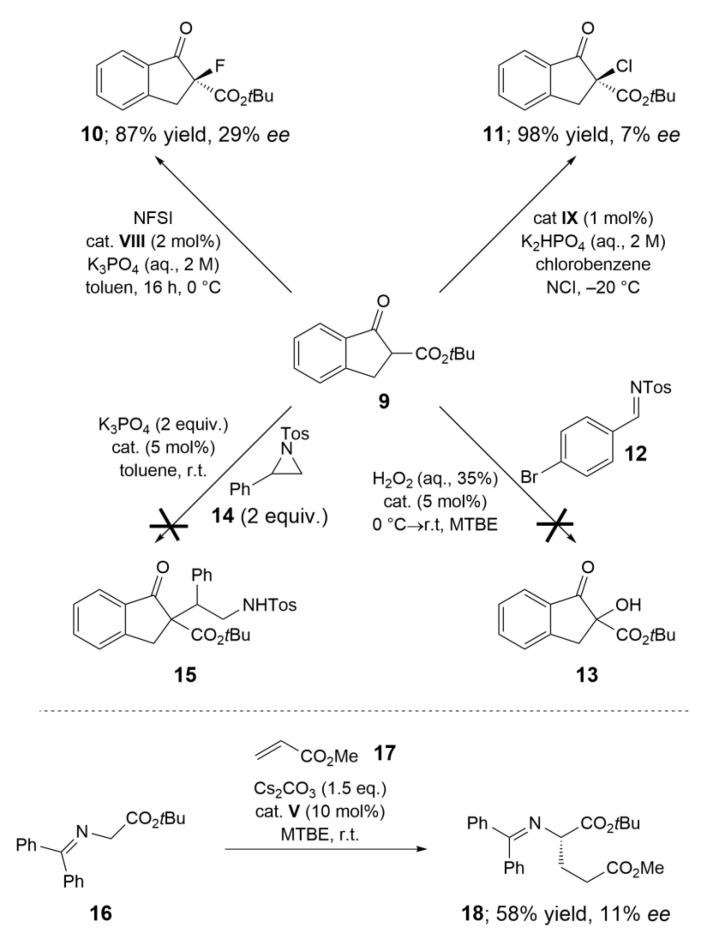
Organocatalytic activity of camphor-derived phase-transfer organocatalysts; MTBE: methyl *tert*-butyl ether.

First, the organocatalytic activity of camphor-derived phase-transfer organocatalysts **I**–**IX** was tested in electrophilic functionalizations of β-keto ester **9** (Scheme 3). Details of the optimization reactions can be found in the Supporting Information. The asymmetric α-fluorination of β-keto ester **9** with *N*-fluorobenzenesulfonimide (NFSI) proceeded under complete conversion and gave the product **10** with low enantioselectivity (87% yield and up to 29% ee). The best result was obtained with the catalyst **VIII** in toluene in the presence of K_3_PO_4_. In contrast, up to 86% ee has been reported in the literature for the α-fluorination of β-keto ester **9** with NFSI [15,32]. Similarly, the α-chlorination of **9** with *N*-chlorosuccinimide (NCI) gave product **11** in complete conversion and a meager 7% ee when squaramide PTC **IX** was used (for comparison, up to 80% ee has been reported in the literature when using alternative catalyst scaffolds [33]). Disappointingly, both the α-hydroxylation [34] of **9** with tosylimine **12**/H_2_O_2_ and the ring opening of arylaziridine **14** [35] with **9** did not give the expected products **13** and **16**, respectively. In both cases, no conversion was observed. Finally, the asymmetric Michael addition of glycine Schiff base **16** to methyl acrylate (**17**) was investigated, with up to 90% ee reported in the literature [36]. Catalyst **V** gave the expected product **18** with full conversion but low enantioselectivity (11% ee).

## 3. Materials and Methods

Solvents for extractions and chromatography were of technical grade and were distilled prior to use. Extracts were dried over technical-grade anhydrous Na_2_SO_4_. Melting points were determined on a Kofler micro hot stage. The NMR spectra were obtained on a Bruker Avance DPX 300 and Bruker Avance III 300 at 300 MHz for ^1^H nucleus, Bruker UltraShield 500 plus (Bruker, Billerica, MA, USA) at 500 MHz for ^1^H and 126 MHz for ^13^C nucleus, and Bruker Ascend 600 (Bruker, Billerica, MA, USA) at 600 MHz for ^1^H and 151 MHz for ^13^C nucleus, using DMSO-*d_6_* and CDCl_3_, with TMS as the internal standard, as solvents. Mass spectra were recorded on an Agilent 6224 Accurate Mass TOF LC/MS (Agilent Technologies, Santa Clara, CA, USA), and IR spectra on a Perkin-Elmer Spectrum BX FTIR spectrophotometer (PerkinElmer, Waltham, MA, USA). CD spectra were recorded on a J-1500 Circular Dichroism Spectrophotometer (JASCO corporation, Tokyo, Japan). Column chromatography (CC) was performed on silica gel (Silica gel 60, particle size: 0.035–0.070 mm (Sigma-Aldrich, St. Louis, MI, USA)). HPLC analyses were performed on an Agilent 1260 Infinity LC (Agilent Technologies, Santa Clara, CA, USA) and Dionex Summit HPLC system (Dionex Corporation, Sunnyvale, CA, USA) using CHIRALPAK AD-H (0.46 cm ø × 25 cm) and CHIRALPAK OJ-H (0.46 cm ø × 25 cm), as the chiral columns (Chiral Technologies, Inc., West Chester, PA, United States). All the commercially available chemicals used were purchased from Sigma-Aldrich (St. Louis, MI, USA). In addition, (1*S*,2*S*,4*R*)-7,7-dimethyl-1-(pyrrolidin-1-ylmethyl)bicyclo[2.2.1]heptan-2-amine (**1b**) was prepared following the literature procedure [30].

### 3.1. Reduction of (1S,4R,E)-1-[(Dimethylamino)methyl]-7,7-dimethylbicyclo[2.2.1]heptan-2-one oxime

Oxime (7.6 mmol, 1.6 g) was dissolved in propan-1-ol (86 mL) and heated to 95 ℃. Then, small pieces of sodium (approximately 50 mg) were added continuously for 1 h at 95 ℃; care was taken to ensure that the unreacted sodium (excess sodium) remained present in the reaction mixture at all times during the reaction. After completion of the reaction, the volatiles were evaporated in vacuo. The residue was dissolved in a mixture of water (20 mL) and Et_2_O (80 mL). The organic phase was washed with water (2 × 20 mL) and NaCl (aq. sat., 1 × 20 mL), dried over anhydrous Na_2_SO_4_, and the volatiles were evaporated in vacuo. Diastereomers **1a** and **2a** were formed in a ratio of 2.6:1. The diastereomers were separated by column chromatography (Silica gel 60, EtOAc/MeOH/Et_3_N = 4:1:1).

#### 3.1.1. (1*S*,2*R*,4*R*)-1-[(Dimethylamino)methyl]-7,7-dimethylbicyclo[2.2.1]heptan-2-amine (**2a**)

Elutes first from the column. Yield: 175 mg (0.89 mmol, 12%) of colorless oil. ^1^H-NMR (500 MHz, CDCl_3_): δ 0.79 (s, 3H), 1.05 (s, 3H), 1.06–1.13 (m, 1H), 1.34 (ddd, *J* = 12.8, 9.4, 3.9, 1H), 1.54–1.61 (m, 3H), 1.63 (t, *J* = 4.3, 1H), 1.64–1.75 (m, 2H), 1.93 (d, *J* = 11.4, 1H), 2.02 (d, *J* = 13.0, 1H), 2.27 (s, 6H), 2.74 (d, *J* = 13.0, 1H), 3.11 (dd, *J* = 8.7, 5.1, 1H).

#### 3.1.2. (1*S*,2*S*,4*R*)-1-[(Dimethylamino)methyl]-7,7-dimethylbicyclo[2.2.1]heptan-2-amine (**1a**)

Elutes second from the column. Yield: 850 mg (4.33 mmol, 57%) of colorless oil. ^1^H-NMR (500 MHz, CDCl_3_): δ 0.67 (dd, *J* = 12.9, 4.3, 1H), 0.86 (s, 3H), 0.89 (s, 3H), 1.22 (ddd, *J* = 12.3, 9.5, 4.4, 1H), 1.38 (ddd, *J* = 12.3, 4.5, 2.0, 1H), 1.49 (t, *J* = 4.6, 1H), 1.70–1.79 (m, 1H), 1.80 (br s, 2H), 2.10 (d, *J* = 13.1, 1H), 2.13–2.17 (m, 1H), 2.20 (s, 6H), 2.21–2.26 (m, 1H), 2.45 (d, *J* = 13.0, 1H), 3.36 (ddd, *J* = 10.6, 4.3, 2.0, 1H).

### 3.2. Boc Protection of Chiral Amines—General Procedure 1 (GP1)

To a solution of amine **1** or **2** and triethylamine (1.4 equivalents) in anhydrous CH_2_Cl_2_ was added di-*tert*-butyl dicarbonate (1.4 equivalents). The resulting reaction mixture was stirred at 25 °C for 24 h. Dichloromethane was evaporated in vacuo and the residue was purified by column chromatography (CC). The fractions containing product **3** or **4** were combined and the volatiles were evaporated in vacuo.

#### 3.2.1. *tert*-Butyl {(1*S*,2*S*,4*R*)-1-[(dimethylamino)methyl]-7,7-dimethylbicyclo[2.2.1]heptan-2-yl}carbamate (**3a**)

Following *GP1*. Prepared from *endo*-amine **1a** (4.69 mmol, 920 mg) and di-*tert*-butyl dicarbonate (6.56 mmol, 1.431 g), Et_3_N (6.56 mmol, 915 µL), CH_2_Cl_2_ (20 mL), 25 °C, 24 h. Isolation by column chromatography (Silica gel 60, EtOAc/petroleum ether = 1:5). Yield: 1.39 g (4.69 mmol, 99%) of colorless oil. [α]^r.t.^_D_ = +11.2 (0.15, MeOH). EI-HRMS: *m*/*z* = 297.2646 (MH)^+^; C_17_H_33_N_2_O_2_^+^ requires: *m*/*z* = 297.2536 (MH)^+^; ν_max_ 3346, 2935, 2819, 2765, 1698, 1483, 1454, 1389, 1364, 1297, 1242, 1167, 1114, 1065, 1040, 1014, 946, 874, 837, 780 cm^−1^. ^1^H-NMR (500 MHz, CDCl_3_): δ 0.86 (s, 3H), 0.90 (s, 3H), 1.04 (dd, *J* = 13.4, 4.3, 1H), 1.21 (ddd, *J* = 12.2, 9.5, 4.4, 1H), 1.43 (s, 9H), 1.45–1.51 (m, 1H), 1.56 (t, *J* = 4.6, 1H), 1.72 (tq, *J* = 12.1, 4.1, 1H), 1.86 (br t, 1H), 2.21 (s, 6H), 2.24 (d, *J* = 13.6, 1H), 2.28–2.33 (m, 1H), 2.36 (d, *J* = 13.8, 1H), 3.75 (s, 1H), 6.00 (s, 1H). ^13^C-NMR (126 MHz, CDCl_3_): δ 19.19, 20.31, 25.40, 28.39, 28.64, 37.92, 45.07, 48.10, 48.33, 50.98, 56.25, 61.97, 78.72, 157.52.

#### 3.2.2. *tert*-Butyl {(1*S*,2*R*,4*R*)-1-[(dimethylamino)methyl]-7,7-dimethylbicyclo[2.2.1]heptan-2-yl}carbamate (**4a**)

Following *GP1*. Prepared from *exo*-amine **2a** (0.81 mmol, 160 mg) and di-*tert*-butyl dicarbonate (1.134 mmol, 247 mg), Et_3_N (1.19 mmol, 166 µL), CH_2_Cl_2_ (4 mL), 25 °C, 24 h. Isolation by column chromatography (Silica gel 60, EtOAc/petroleum ether = 1:5). Yield: 230 mg (0.78 mmol, 95%) of colorless oil. [α]^r.t.^_D_ = +25.7 (0.175, MeOH). EI-HRMS: *m*/*z* = 297.2536 (MH)^+^; C_17_H_33_N_2_O_2_^+^ requires: *m*/*z* = 297.2537 (MH)^+^; ν_max_ 3344, 2935, 2819, 2765, 1698, 1484, 1453, 1389, 1364, 1297, 1243, 1167, 1113, 1065, 1040, 1004, 943, 874, 837, 780 cm^−1^. ^1^H-NMR (500 MHz, CDCl_3_): δ 0.87 (s, 3H), 0.99 (s, 3H), 1.09–1.17 (m, 1H), 1.34 (t, *J* = 9.4, 1H), 1.42–1.45 (m, 1H), 1.43 (s, 9H), 1.67 (d, *J* = 3.5, 2H), 1.69–1.75 (m, 1H), 1.86 (d, *J* = 8.4, 1H), 2.24 (s, 6H), 2.25 (d, *J* = 13.9, 1H), 2.40 (d, *J* = 13.9, 1H), 3.71 (br s, 1H), 5.58 (br s, 1H). ^13^C-NMR (126 MHz, CDCl_3_): δ 20.95, 27.15, 28.50, 28.67, 30.48, 33.79, 40.55, 45.67, 48.03, 50.94, 57.44, 58.86, 78.90, 155.72.

#### 3.2.3. *tert*-Butyl [(1*S*,2*S*,4*R*)-7,7-dimethyl-1-(pyrrolidin-1-ylmethyl)bicyclo[2.2.1]heptan-2-yl}carbamate (**3b**)

Following *GP1*. Prepared from *endo*-amine **1b** (3.91 mmol, 869 mg) and di-*tert*-butyl dicarbonate (5.474 mmol, 1.194 g), Et_3_N (5.474 mmol, 763 µL), CH_2_Cl_2_ (20 mL), 25 °C, 24 h. Isolation by column chromatography (Silica gel 60, EtOAc/petroleum ether = 1:5). Yield: 1.251 g (3.88 mmol, 99%) of brownish oil. [α]^r.t.^_D_ = +1.1 (0.295, MeOH). EI-HRMS: *m*/*z* = 323.2688 (MH)^+^; C_19_H_35_N_2_O_2_^+^ requires: *m*/*z* = 323.2693 (MH)^+^; ν_max_ 3300, 2979, 2937, 2879, 2794, 1808, 1757, 1715, 1460, 1395, 1371, 1306, 1250, 1211, 1168, 1113, 1062, 950, 844, 775, 664 cm^−1^. ^1^H-NMR (600 MHz, CDCl_3_): δ 0.85 (s, 3H), 0.90 (s, 3H), 1.07 (dd, *J* = 13.4, 4.4, 1H), 1.21 (ddd, *J* = 12.8, 9.5, 4.5, 1H), 1.4–1.45 (m, 1H), 1.41 (s, 9H), 1.56 (t, *J* = 4.6, 1H), 1.65–1.73 (m, 6H), 1.90 (ddd, *J* = 13.6, 8.9, 4.1, 1H), 2.31 (s, 1H), 2.37 (d, *J* = 13.4, 1H), 2.41–2.46 (m, 2H), 2.56–2.60 (m, 2H), 2.66 (d, *J* = 13.4, 1H), 3.72 (br s, 1H), 6.31 (br s, 1H). ^13^C-NMR (151 MHz, CDCl_3_): δ 19.18, 20.23, 24.16, 26.03, 28.43, 28.61, 37.58, 45.22, 47.88, 50.81, 56.47, 56.82, 58.03, 78.48, 157.80.

### 3.3. Benzylation of Tertiary Amines—General Procedure 2 (GP2)

To a stirred mixture of tertiary amine **3** or **4** and K_2_CO_3_ (1.1 equivalents) in anhydrous DMF was added benzyl bromide (1.1 equivalents). The resulting reaction mixture was stirred at 25 °C for 24 h. DMF was evaporated in vacuo and the residue was purified by column chromatography (CC). The fractions containing product **5** or **6** were combined and the volatiles were evaporated in vacuo.

#### 3.3.1. *N*-Benzyl-1-{(1*S*,2*S*,4*R*)-2-[(tert-butoxycarbonyl)amino]-7,7-dimethylbicyclo[2.2.1]heptan-1-yl}-*N*,*N*-dimethylmethanaminium Bromide (**5a**)

Following *GP2*. Prepared from compound **3a** (1.06 mmol, 315 mg) and benzyl bromide (1.16 mmol, 139 µL), K_2_CO_3_ (1.16 mmol, 160 mg), DMF (5 mL), 25 °C, 24 h. Isolation by column chromatography (Silica gel 60, EtOAc/MeOH = 4:1). Yield: 340 mg (0.73 mmol, 69%) of colorless oil. [α]^r.t.^_D_ = +14.0 (0.087, MeOH). EI-HRMS: *m*/*z* = 387.3003 (M)^+^; C_24_H_39_N_2_O_2_ requires: *m*/*z* = 387.3006 (M)^+^; ν_max_ 3369, 3197, 2951, 2199, 2163, 2098, 1989, 1685, 1540, 1490, 1477, 1454, 1392, 1379, 1366, 1299, 1284, 1271, 1252, 1217, 1158, 1125, 1065, 1042, 1012, 947, 917, 882, 868, 854, 839, 783, 752, 732, 706 cm^−1^. ^1^H-NMR (500 MHz, CDCl_3_): δ 0.87–0.93 (m, 1H), 0.94 (s, 3H), 0.98 (s, 3H), 1.29–1.34 (m, 1H), 1.36 (s, 9H), 1.58 (t, *J* = 4.4, 1H), 1.86 (br t, *J* = 11.7, 1H), 1.93–2.03 (m, 1H), 2.21 (br t, *J* = 13.1, 1H), 2.47 (d, *J* = 11.7, 1H), 3.17 (s, 3H), 3.25 (s, 3H), 3.44 (br d, *J* = 13.6, 1H), 4.11 (br d, *J* = 13.5, 1H), 4.18 (br t, *J* = 9.9, 1H), 4.97 (d, *J* = 12.3, 1H), 5.03 (br s, 1H), 5.20 (d, *J* = 12.2, 1H), 7.32–7.43 (m, 3H), 7.61 (d, *J* = 7.4, 2H). ^13^C-NMR (126 MHz, CDCl_3_): δ 19.48, 20.76, 27.34, 28.28, 28.75, 40.19, 43.67, 50.40, 51.55, 53.72, 54.15, 69.24, 70.42, 80.67, 127.78, 128.94, 130.51, 133.47, 156.14.

#### 3.3.2. *N*-Benzyl-1-{(1*S*,2*R*,4*R*)-2-[(*tert*-butoxycarbonyl)amino]-7,7-dimethylbicyclo[2.2.1]heptan-1-yl}-*N*,*N*-dimethylmethanaminium Bromide (**6a**)

Following *GP2*. Prepared from compound **4a** (2.53 mmol, 748 mg) and benzyl bromide (3.795 mmol, 453 µL), K_2_CO_3_ (2.78 mmol, 385 mg), DMF (13 mL), 25 °C, 24 h. Isolation by column chromatography (Silica gel 60, EtOAc/MeOH = 4:1). Yield: 904 mg (1.93 mmol, 76%) of colorless oil. [α]^r.t.^_D_ = −4.3 (0.26, MeOH). EI-HRMS: *m*/*z* = 387.3000 (M)^+^; C_24_H_39_N_2_O_2_^+^ requires: *m*/*z* = 387.3006 (M)^+^; ν_max_ 3341, 2965, 2885, 2156, 1698, 1606, 1508, 1475, 1456, 1365, 1278, 1247, 1168, 1060, 1019, 953, 860, 782, 732, 706 cm^−1^. ^1^H-NMR (500 MHz, CDCl_3_): δ 0.92 (dd, *J* = 13.4, 3.5, 1H), 0.97 (s, 3H), 1.03 (s, 3H), 1.35 (t, *J* = 4.8, 1H), 1.39 (s, 9H), 1.62 (t, *J* = 4.5, 1H), 1.88–1.99 (m, 2H), 2.29 (br t, *J* = 12.9, 1H), 2.46–2.56 (m, 1H), 3.19 (s, 3H), 3.27 (s, 3H), 3.43 (br d, *J* = 14.3, 1H), 4.16–4.26 (m, 2H), 4.92 (d, *J* = 10.9, 1H), 4.97 (d, *J* = 12.4, 1H), 5.21 (d, *J* = 12.4, 1H), 7.37–7.46 (m, 3H), 7.63 (d, *J* = 6.9, 2H). ^13^C-NMR (126 MHz, CDCl_3_): δ 19.60, 20.95, 27.47, 28.40, 28.91, 40.49, 43.82, 50.54, 51.73, 53.85, 54.30, 69.39, 70.63, 80.94, 127.79, 129.12, 130.72, 133.57, 156.18.

#### 3.3.3. 1-Benzyl-1-({(1*S*,2*S*,4*R*)-2-[(*tert*-butoxycarbonyl)amino]-7,7-dimethylbicyclo[2.2.1]heptan-1-yl}methyl)pyrrolidin-1-ium Bromide (**5b**)

Following *GP2*. Prepared from compound **3b** (2.48 mmol, 828 mg) and benzyl bromide (2.73 mmol, 324 µL), K_2_CO_3_ (2.73 mmol, 377 mg), DMF (13 mL), 25 °C, 24 h. Isolation by column chromatography (Silica gel 60, EtOAc/MeOH = 4:1). Yield: 469 mg (1.45 mmol, 59%) of brownish semisolid. [α]^r.t.^_D_ = +17.7 (0.12, MeOH). EI-HRMS: *m*/*z* = 413.3161 (M)^+^; C_26_H_41_N_2_O_2_^+^ requires: *m*/*z* = 413.3162 (M)^+^; ν_max_ 3323, 3270, 2965, 2923, 1708, 1639, 1531, 1452, 1388, 1363, 1307, 1247, 1159, 1121, 1066, 1028, 1002, 923, 901, 855, 839, 780, 710 cm^−1^. ^1^H-NMR (500 MHz, CDCl_3_): δ 0.92 (s, 3H), 0.91–0.96 (m, 1H), 0.96 (s, 3H), 1.32–1.35 (br t, 1H), 1.37 (s, 9H), 1.55 (t, *J* = 4.5, 1H), 1.62–1.73 (m, 1H), 1.74–1.86 (m, 2H), 1.88–2.04 (m, 2H), 2.04–2.13 (m, 1H), 2.17–2.27 (m, 1H), 2.39–2.48 (m, 1H), 3.39 (d, *J* = 14.0, 1H), 3.41–3.50 (m, 1H), 3.58–3.74 (m, 2H), 3.96 (ddd, *J* = 12.3, 8.1, 6.3, 1H), 4.13 (d, *J* = 14.0, 1H), 4.24 (tt, *J* = 10.8, 3.1, 1H), 4.59 (d, *J* = 12.6, 1H), 5.22 (br s, 1H), 5.26 (d, *J* = 10.8, 1H), 7.28–7.39 (m, 3H), 7.57 (d, *J* = 7.0, 2H). ^13^C-NMR (126 MHz, CDCl_3_): δ 19.33, 20.70, 21.60, 22.07, 27.88, 28.29, 28.81, 39.70, 43.63, 51.48, 53.44, 53.74, 59.55, 62.11, 63.58, 67.05, 80.48, 128.21, 128.99, 130.40, 133.30, 156.03.

### 3.4. Boc Deprotection of Amines—General Procedure 3 (GP3)

To a solution of amine **5** or **6** in anhydrous CH_2_Cl_2_ (2.5 mL/mmol) was added trifluoroacetic acid (2.5 mL/mmol). The resulting reaction mixture was stirred at 25 °C for 2 h. Dichloromethane and trifluoroacetic acid were evaporated in vacuo and the residue was dissolved in CH_2_Cl_2_ (2.5 mL/mmol). The organic phase was washed with NaOH (aq., 2 M, 2 × 2.5 mL/mmol) and NaCl (aq. sat., 1 × 2.5 mL/mmol). The volatiles were evaporated in vacuo to give product **7** or **8**.

#### 3.4.1. (1*S*,2*S*,4*R*)-1-[(Benzyldimethylammonio)methyl]-7,7-dimethylbicyclo[2.2.1]heptan-2-aminium 2,2,2-Trifluoroacetate (**7a**)

Following *GP3*. Prepared from compound **5a** (2.1 mmol, 1 g), trifluoroacetic acid (5 mL), CH_2_Cl_2_ (5 mL), 25 °C, 2 h. Volatile components were evaporated in vacuo, and the residue was dissolved in dichloromethane and washed with NaOH (aq., 2 M) and NaCl (aq. sat.). Yield: 605 mg (1.51 mmol, 72%) of colorless solid, mp = 179.9–182.1 °C. [α]^r.t.^_D_ = +16.2 (0.125, MeOH). EI-HRMS: *m*/*z* = 287.2483 (M)^+^; C_19_H_31_N_2_^+^ requires: *m*/*z* = 287.2482 (M)^+^; ν_max_ 3377, 3292, 3042, 2943, 2881, 1685, 1585, 1479, 1457, 1401, 1372, 1302, 1196, 1157, 1113, 1048, 1025, 1010, 989, 935, 917, 881, 854, 819, 780, 785, 753, 733, 716, 707, 632, 607 cm^−1^. ^1^H-NMR (500 MHz, CDCl_3_): δ 0.89 (dd, *J* = 3.4, 13.1, 1H); 0.93 (s, 3H); 0.97 (s, 3H); 1.31–1.38 (m, 1H); 1.61 (t, *J* = 4.6, 1H); 1.77–1.84 (m, 1H); 1.86–1.96 (m, 1H); 2.05–2.13 (m, 1H); 2.44–2.53 (m, 1H); 3.28 (s, 3H); 3.37 (s, 3H); 3.43–3.49 (m, 1H); 3.68 (d, *J* = 13.9, 1H); 3.72 (d, *J* = 13.9, 1H); 4.97 (d, *J* = 12.4, 1H); 5.11 (d, *J* = 12.3, 1H); 7.40–7.48 (m, 3H); 7.56–7.60 (m, 2H), signal for NH_2_ is missing. ^13^C-NMR (126 MHz, CDCl_3_): δ 19.41, 20.51, 26.70, 29.29, 44.19, 44.63, 49.97, 50.49, 52.44, 53.20, 53.69, 69.11, 71.98, 117.64 (q, *J* = 297.3), 128.29, 129.14, 130.57, 133.49, 161.16 (q, *J* = 32.7).

#### 3.4.2. (1*S*,2*R*,4*R*)-1-[(Benzyldimethylammonio)methyl]-7,7-dimethylbicyclo[2.2.1]heptan-2-aminium 2,2,2-Trifluoroacetate (**8a**)

Following *GP3*. Prepared from compound **6a** (1.92 mmol, 900 mg), trifluoroacetic acid (5 mL), CH_2_Cl_2_ (5 mL), 25 °C, 2 h. Volatile components were evaporated in vacuo, and the residue was dissolved in dichloromethane and washed with NaOH (aq., 2 M) and NaCl (aq. sat.). Yield: 567 mg (1.41 mmol, 74%) of colorless solid, mp = 157.1–158.8 °C. [α]^r.t.^_D_ = −9.8 (0.11, MeOH). EI-HRMS: *m*/*z* = 287.2477 (M)^+^; C_19_H_31_N_2_^+^ requires: *m*/*z* = 287.2482 (M)^+^; ν_max_ 2953, 2883, 1684, 1476, 1456, 1393, 1371, 1311, 1196, 1153, 1113, 1035, 1009, 936, 911, 851, 820, 798, 784, 735, 714, 631 cm^−1^. ^1^H-NMR (600 MHz, CDCl_3_): δ 0.86 (s, 3H), 0.90 (s, 3H), 1.15–1.22 (m, 1H), 1.34–1.41 (m, 1H), 1.50–1.55 (m, 1H), 1.67 (s, 1H), 1.77 (d, *J* = 13.9, 3H), 1.92 (dd, *J* = 13.0, 7.3, 2H), 3.01 (dd, *J* = 9.0, 4.9, 1H), 3.19 (s, 6H), 3.33 (d, *J* = 13.5, 1H), 4.14 (d, *J* = 13.5, 1H), 4.75 (d, *J* = 12.5, 1H), 4.86 (d, *J* = 12.8, 1H), 7.35–7.41 (m, 3H), 7.56 (d, *J* = 7.4, 2H). ^13^C-NMR (126 MHz, CDCl_3_): δ 20.58, 21.40, 27.73, 32.27, 44.01, 44.29, 50.60, 51.35, 51.53, 52.44, 57.11, 64.64, 71.26, 117.56 (q, *J* = 296.8), 127.79, 129.26, 130.81, 133.53, 161.25 (q, *J* = 32.7).

#### 3.4.3. Synthesis of 1-{[(1*S*,2*S*,4*R*)-2-Ammonio-7,7-dimethylbicyclo[2.2.1]heptan-1-yl]methyl}-1-benzylpyrrolidin-1-ium Iodide (**7b**)

Compound **5b** (0.55 mmol, 270 mg) was dissolved in anhydrous CH_2_Cl_2_ (8 mL), and then HI (aq., 48%, 5 equivalents, 2.75 mmol, 495 µL) was added. The reaction mixture was stirred for 4 h at 25 °C. Volatile components were evaporated in vacuo, and the residue was dissolved in dichloromethane (5 mL) and washed with NaOH (aq., 2 M, 2 × 5mL) and NaCl (aq. sat., 1 × 5mL). Yield: 150 mg (0.34 mmol, 62%) of yellowish semisolid. [α]^r.t.^_D_ = +18.8 (0.15, MeOH). EI-HRMS: *m*/*z* = 313.2635 (M)^+^; C_21_H_33_N_2_^+^ requires: *m*/*z* = 313.2635 (M)^+^; ν_max_ 3273, 2951, 2881, 2188, 2152, 1969, 1594, 1458, 1372, 1303, 1217, 1142, 1077, 1033, 1004, 917, 822, 764, 725, 641 cm^−1^. ^1^H-NMR (600 MHz, CDCl_3_): δ 1.03–1.06 (m, 1H), 1.06 (s, 3H), 1.07 (s, 3H), 1.41–1.50 (m, 1H), 1.67 (t, *J* = 4.6, 1H), 1.77 (td, *J* = 10.7, 9.7, 6.5, 1H), 1.81–1.88 (m, 1H), 1.92–1.99 (m, 1H), 2.05–2.11 (m, 1H), 2.12–2.21 (m, 2H), 2.26 (ddd, *J* = 13.4, 9.4, 4.1, 1H), 2.36–2.75 (m, 3H), 3.68 (dt, *J* = 10.5, 3.1, 1H), 3.75 (ddd, *J* = 12.3, 8.5, 5.9, 1H), 3.81–3.87 (m, 1H), 3.83 (d, *J* = 14.1, 1H), 3.89 (d, *J* = 14.2, 1H), 4.07 (ddd, *J* = 12.2, 8.3, 6.3, 1H), 4.16 (ddd, *J* = 11.9, 8.2, 6.2, 1H), 5.06 (d, *J* = 12.6, 1H), 5.25 (d, *J* = 12.6, 1H), 7.42–7.50 (m, 3H), 7.66 (d, *J* = 6.6, 2H). ^13^C-NMR (151 MHz, CDCl_3_): δ 19.88, 21.16, 21.96, 22.08, 26.80, 29.23, 42.76, 44.69, 52.63, 53.69, 54.48, 60.85, 61.96, 63.75, 67.23, 128.66, 129.39, 130.71, 133.42.

### 3.5. Synthesis of Phase-Transfer Bifunctional Catalysts—General Procedure 4 (GP4)

Amine **7** or **8** was dissolved in anhydrous CH_2_Cl_2_, the appropriate electrophile was added (1.2–1.4 equivalents), and the reaction mixture was stirred for 16 h at room temperature. The volatiles were evaporated in vacuo. The residue was purified by column chromatography (CC). The fractions containing product **I**–**X** were combined and the volatiles were evaporated in vacuo.

### 3.6. Trifluoroacetate Anion Exchange—General Procedure 5 (GP5)

The column was packed with NaI (5 g) and conditioned with ethyl acetate. The trifluoroacetate phase-transfer catalyst was dissolved in ethyl acetate and applied to the NaI column. The fractions containing the product were combined and the volatiles were evaporated in vacuo. Based on the ^19^F NMR spectra (presence of a signal for fluorine from trifluoroacetate anion), the procedure was repeated as necessary.

#### 3.6.1. *N*-Benzyl-1-((1*R*,2*R*,4*R*)-2-{3-[3,5-bis(trifluoromethyl)phenyl]thioureido}-7,7-dimethylbicyclo[2.2.1]heptan-1-yl)-*N*,*N*-dimethylmethanaminium 2,2,2-Trifluoroacetate (**I**)

Following *GP4*. Prepared from compound **8a** (0.585 mmol, 300 mg) and 3,5-bis(trifluoromethyl)phenyl isothiocyanate (1.05 mmol, 192 µL), CH_2_Cl_2_ (4 mL), 25 °C, 16 h. Isolation by evaporation followed by column chromatography (Silica gel 60, EtOAc/MeOH = 4:1). Yield: 316 mg (0.47 mmol, 80%) of yellowish solid, mp = 87.5–89.0 °C. [α]^r.t.^_D_ = +6.8 (0.13, MeOH). EI-HRMS: *m*/*z* = 558.2374 (M)^+^; C_28_H_34_F_6_N_3_S^+^ requires: *m*/*z* = 558.2372 (M)^+^; ν_max_ 3260, 2962, 2885, 2091, 1679, 1622, 1523, 1472, 1382, 1333, 1274, 1218, 1171, 1125, 999, 970, 883, 848, 828, 780, 760, 727, 700, 680 cm^−1^. ^1^H-NMR (500 MHz, CDCl_3_): δ 0.89 (s, 3H); 1.18 (s, 3H); 1.36–1.45 (m, 1H); 1.66–1.74 (m, 1H); 1.81–1.94 (m, 4H); 2.22 (dd, *J* = 8.6, 13.4, 1H); 3.00 (s, 3H); 3.08 (s, 3H); 3.29 (d, *J* = 14.5, 1H); 4.65 (d, *J* = 12.6, 1H); 4.74–4.80 (m, 1H); 4.84–4.93 (m, 2H); 7.40–7.54 (m, 6H); 8.36 (s, 2H); 8.79 (d, *J* = 7.9, 1H); 10.97 (s, 1H). ^13^C NMR (126 MHz, CDCl_3_): δ 20.76, 20.93, 27.84, 34.28, 41.11, 43.92, 50.38, 50.44, 51.25, 53.08, 58.99, 66.90, 71.59, 113.57, 116.74–117.00 (m), 117.08 (q, *J* = 294.0), 122.13 (d, *J* = 4.0), 123.41 (q, *J* = 272.6), 124.49, 126.58, 129.53, 131.34 (q, *J* = 33.2), 131.37, 133.09, 141.70, 162.07 (q, *J* = 34.3), 180.02.

#### 3.6.2. *N*-Benzyl-1-((1*R*,2*R*,4*R*)-2-{3-[3,5-bis(trifluoromethyl)phenyl]thioureido}-7,7-dimethylbicyclo[2.2.1]heptan-1-yl)-*N*,*N*-dimethylmethanaminium Iodide (**II**)

Following *GP5*. Prepared from catalyst **I** (0.21 mmol, 140 mg), dissolved in ethyl acetate (4 mL) and filtered through a pad of NaI. Volatile components were evaporated in vacuo. Yield: 140 mg (0.20 mmol, 96%) of colorless solid, mp = 61.4–62.9 °C. [α]^r.t.^_D_ = +5 (0.19, MeOH). EI-HRMS: *m*/*z* = 558.2365 (M)^+^; C_28_H_34_F_6_N_3_S^+^ requires: *m*/*z* = 558.2372 (M)^+^; ν_max_ 3305, 2964, 1674, 1536, 1473, 1384, 1336, 1275, 1172, 1123, 1000, 971, 884, 846, 801, 780, 759, 725, 701, 679 cm^−1^. ^1^H-NMR (500 MHz, CDCl_3_): δ 0.96 (s, 3H); 1.28 (s, 3H); 1.39–1.46 (m, 1H); 1.64–1.73 (m, 1H); 1.81–2.01 (m, 4H); 2.23 (dd, *J* = 8.8, 13.4, 1H); 3.08 (s, 3H); 3.14 (s, 3H); 3.51 (d, *J* = 14.3, 1H); 4.81–4.89 (m, 1H); 4.96 (d, *J* = 12.5, 1H); 5.07 (d, *J* = 13.9, 1H); 5.17 (d, *J* = 12.7, 1H); 7.38–7.45 (m, 2H); 7.46–7.51 (m, 1H); 7.54 (s, 1H); 7.55–7.60 (m, 2H); 8.08 (d, *J* = 8.1, 1H); 8.43 (s, 2H); 10.52 (s, 1H). ^13^C-NMR (126 MHz, CDCl_3_): δ 21.37, 21.94, 27.90, 34.23, 41.04, 43.83, 50.02, 50.70, 51.55, 53.44, 59.26, 66.70, 70.85, 117.35, 122.27, 123.36 (q, *J* = 272.7), 126.56, 129.49, 131.36, 131.36 (q, *J* = 33.3), 133.31, 141.23, 179.91.

#### 3.6.3. 1-Benzyl-1-[((1*S*,2*S*,4*R*)-2-{3-[3,5-bis(trifluoromethyl)phenyl]thioureido}-7,7-dimethylbicyclo[2.2.1]heptan-1-yl)methyl]yrrolidine-1-ium Iodide (**III**)

Following *GP4*. Prepared from compound **7b** (0.34 mmol, 150 mg) and 3,5-bis(trifluoromethyl)phenyl isothiocyanate (0.68 mmol, 124 µL), CH_2_Cl_2_ (3 mL), 25 °C, 16 h. Isolation by column chromatography (Silica gel 60, EtOAc/MeOH = 10:1). Yield: 99 mg (0.14 mmol, 41%) of brownish semisolid. [α]^r.t.^_D_ = +20 (0.047, MeOH). EI-HRMS: *m*/*z* = 584.2519 (M)^+^; C_30_H_36_F_6_N_3_S^+^ requires: *m*/*z* = 584.2529 (M)^+^; ν_max_ 3194, 3126, 2968, 2149, 1625, 1589, 1542, 1492, 1472, 1381, 1324, 1271, 1249, 1222, 1166, 1136, 1108, 1094, 1061, 1025, 999, 967, 909, 885, 847, 756, 721, 701, 679, 612 cm^−1^. ^1^H-NMR (500 MHz, CDCl_3_): δ 1.03 (s, 3H); 1.06 (s, 3H); 1.15 (dd, *J* = 13.4, 3.9, 1H); 1.51–1.93 (m, 5H); 1.98–2.06 (m, 1H); 2.16–2.31 (m, 2H), 2.61–2.70 (m, 1H); 2.99–3.07 (m, 1H); 3.41–3.51 (m, 2H), 3.67–3.83 (m, 3H); 3.83–3.93 (m, 1H); 4.63 (d, *J* = 12.9, 1H); 4.96 (d, *J* = 12.8, 1H); 5.34–5.45 (m, 1H); 7.39–7.45 (m, 2H); 7.47–7.52 (m, 1H); 7.54–7.58 (m, 2H); 7.60 (s, 1H); 8.34–8.43 (m, 3H); 10.67 (s, 1H). ^13^C-NMR (126 MHz, CDCl_3_): δ 19.98, 20.65, 21.49, 21.93, 28.65, 29.57, 38.39, 43.68, 51.83, 54.54, 57.00, 60.89, 61.91, 64.34, 67.34, 117.85–118.26 (m), 123.08–123.29 (m), 123.38 (q, *J* = 209.2), 127.38, 129.67, 131.28, 131.57 (q, *J* = 33.4), 133.39, 140.92, 182.20.

#### 3.6.4. *N*-Benzyl-1-((1*R*,2*S*,4*R*)-2-{3-[3,5-bis(trifluoromethyl)phenyl]thioureido}-7,7-dimethylbicyclo[2.2.1]heptan-1-yl)-*N*,*N*-dimethylmethanaminium 2,2,2-Trifluoroacetate (**IV**)

Following *GP4*. Prepared from compound **7a** (0.39 mmol, 200 mg) and 3,5-bis(trifluoromethyl)phenyl isothiocyanate (0.70 mmol, 128 µL), CH_2_Cl_2_ (4 mL), 25 °C, 16 h. Isolation by column chromatography (Silica gel 60, EtOAc/MeOH = 4:1). Yield: 250 mg (0.37 mmol, 95%) of colorless solid, mp = 153–155 °C. [α]^r.t.^_D_ = +2.1 (0.11, MeOH). EI-HRMS: *m*/*z* = 558.2363 (M)^+^; C_28_H_34_F_6_N_3_S^+^ requires: *m*/*z* = 558.2372 (M)^+^; ν_max_ 3275, 3247, 3047, 2961, 2890, 1682, 1542, 1473, 1385, 1278, 1177, 1132, 966, 887, 848, 801, 719, 702, 680 cm^−1^. ^1^H-NMR (500 MHz, CDCl_3_): δ 0.97 (s, 3H), 1.05 (s, 3H), 1.10 (dd, *J* = 13.4, 3.7, 1H), 1.51 (ddd, *J* = 13.4, 9.2, 4.7, 1H), 1.73 (t, *J* = 4.6, 1H), 1.78–1.89 (m, 1H), 1.95–2.12 (m, 1H), 2.59–2.67 (m, 1H), 2.68–2.76 (m, 1H), 3.03 (s, 3H), 3.04 (s, 3H), 3.47 (d, *J* = 13.7, 1H), 3.72 (d, *J* = 13.7, 1H), 4.55 (d, *J* = 12.6, 1H), 4.71 (d, *J* = 12.6, 1H), 5.22 (tt, *J* = 10.2, 3.0, 1H), 7.34–7.40 (m, 4H), 7.43–7.48 (m, 1H), 7.53 (s, 1H), 8.29 (s, 2H), 8.91 (d, *J* = 9.8, 1H), 11.09 (s, 1H). ^13^C-NMR (126 MHz, CDCl_3_): δ 19.81, 20.25, 28.05, 28.57, 38.68, 43.75, 50.78, 51.03, 51.76, 54.43, 56.70, 69.58, 73.03, 117.02 (q, *J* = 294.4), 117.24–117.53 (m), 122.72 (d, *J* = 3.4), 123.39 (q, *J* = 272.7), 126.96, 129.47, 131.24, 131.48 (q, *J* = 33.4), 133.03, 141.44, 161.62 (q, *J* = 34.1), 181.96.

#### 3.6.5. *N*-Benzyl-1-((1*R*,2*S*,4*R*)-2-{3-[3,5-bis(trifluoromethyl)phenyl]thioureido}-7,7-dimethylbicyclo[2.2.1]heptan-1-yl)-*N*,*N*-dimethylmethanaminium Iodide (**V**)

Following *GP5*. Prepared from catalyst **IV** (0.17 mmol, 116 mg), dissolved in ethyl acetate (3 mL) and filtered through a pad of NaI. Volatile components were evaporated in vacuo. Yield: 109 mg (0.16 mmol, 92%) of white solid, mp = decomposition above 350 °C. [α]^r.t.^_D_ = +69.2 (0.013, MeOH). EI-HRMS: *m*/*z* = 558.2368 (M)^+^; C_28_H_34_F_6_N_3_S^+^ requires: *m*/*z* = 558.2372 (M)^+^; ν_max_ 3247, 2960, 2928, 2857, 2175, 2163, 2135, 2034, 1996, 1954, 1722, 1595, 1534, 1473, 1385, 1277, 1177, 1135, 965, 887, 730, 701, 680 cm^−1^. ^1^H-NMR (500 MHz, DMSO-*d*_6_): δ 0.98 (s, 3H), 1.02 (dd, *J* = 13.0, 3.7, 1H), 1.05 (s, 3H), 1.43–1.52 (m, 1H), 1.72 (t, *J* = 4.4, 1H), 1.90–2.06 (m, 2H), 2.16–2.24 (m, 1H), 2.45–2.49 (m, 1H), 2.95 (s, 3H), 2.99 (s, 3H), 3.60 (d, *J* = 14.1, 1H), 3.71 (d, *J* = 14.0, 1H), 4.52–4.63 (m, 2H), 5.04–5.12 (m, 1H), 7.43–7.59 (m, 5H), 7.81 (s, 1H), 8.28 (s, 2H), 8.38 (d, *J* = 9.8, 1H), 10.32 (s, 1H). ^13^C-NMR (126 MHz, DMSO-*d_6_*): δ 19.25, 20.04, 26.97, 28.11, 38.10, 42.97, 49.08, 50.46, 51.23, 53.88, 56.13, 67.81, 70.46, 116.76–117.00 (m), 122.27–122.44 (m), 123.17 (q, *J* = 272.8), 128.03, 128.86, 130.17 (q, *J* = 32.8), 130.38, 133.06, 141.46, 181.00.

#### 3.6.6. *N*-Benzyl-1-[(1*R*,2*S*,4*R*)-7,7-dimethyl-2-(3-phenylthioureido)bicyclo[2.2.1]heptan-1-yl]-*N*,*N*-dimethylmethanaminium 2,2,2-Trifluoroacetate (**VI**)

Following *GP4*. Prepared from compound **7a** (0.39 mmol, 200 mg) and phenyl isothiocyanate (0.70 mmol, 84 µL), CH_2_Cl_2_ (4 mL), 25 °C, 16 h. Isolation by evaporation followed by column chromatography (Silica gel 60, EtOAc/MeOH = 4:1). Yield: 119 mg (0.22 mmol, 56 %) of colorless solid, mp = 180–183 °C. [α]^r.t.^_D_ = +6.7 (0.06, MeOH). EI-HRMS: *m*/*z* = 422.2618 (M)^+^; C_26_H_36_N_3_S^+^ requires: *m*/*z* = 422.2624 (M)^+^; ν_max_ 3244, 2959, 2884, 1683, 1540, 1507, 1489, 1473, 1457, 1362, 1317, 1202, 1148, 1056, 1033, 851, 801, 727 cm^−1^. ^1^H-NMR (500 MHz, CDCl_3_): δ 0.96 (s, 3H); 1.04 (s, 3H); 1.08 (dd, *J* = 13.3, 3.7, 1H); 1.45–1.53 (m, 1H); 1.67–1.79 (m, 2H); 1.96–2.06 (m, 1H); 2.57–2.65 (m, 1H); 2.67–2.76 (m, 1H); 2.98 (s, 3H); 3.03 (s, 3H); 3.41 (d, *J* = 13.6, 1H); 3.73 (d, *J* = 13.8, 1H); 4.54 (d, *J* = 12.5, 1H); 4.79 (d, *J* = 12.4, 1H), 5.24–5.31 (m, 1H), 7.04–7.10 (m, 1H); 7.22–7.29 (m, 2H); 7.33–7.47 (m, 5H); 7.60–7.68 (m, 2H); 8.64 (d, *J* = 9.9, 1H); 10.45 (s, 1H). ^13^C-NMR (126 MHz, CDCl_3_): δ 19.95, 20.50, 28.53, 28.65, 38.67, 43.85, 51.11, 51.22, 51.72, 54.34, 56.50, 69.08, 72.83, 123.82, 124.84, 127.30, 128.46, 129.38, 130.98, 133.35, 139.65, 182.21 (two signals missing).

#### 3.6.7. *N*-Benzyl-1-[(1*R*,2*S*,4*R*)-7,7-dimethyl-2-(3-phenylureido)bicyclo[2.2.1]heptan-1-yl]-*N*,*N*-dimethylmethanaminium 2,2,2-Trifluoroacetate (**VII**)

Following *GP4*. Prepared from compound **7a** (0.39 mmol, 200 mg) and phenyl isocyanate (0.69 mmol, 76 µL), CH_2_Cl_2_ (4 mL), 25 °C, 16 h. Isolation by evaporation followed by column chromatography (Silica gel 60, EtOAc/MeOH = 5:1). Yield: 57 mg (0.11 mmol, 28%) of colorless solid, mp = 120.0–123.8 °C. [α]^r.t.^_D_ = +5 (0.08, MeOH). EI-HRMS: *m*/*z* = 406.2850 (M)^+^; C_26_H_36_N_3_O^+^ requires: *m*/*z* = 406.2853 (M)^+^; ν_max_ 3261, 2960, 2886, 2150, 1683, 1598, 1550, 1489, 1457, 1313, 1202, 1139, 846, 801, 727, 702 cm^−1^. ^1^H-NMR (500 MHz, CDCl_3_): δ 0.95 (s, 3H), 1.00 (s, 3H), 1.12 (dd, *J* = 13.3, 3.6, 1H), 1.44–1.53 (m, 1H), 1.55–1.68 (m, 2H), 2.03–2.11 (m, 1H), 2.47–2.55 (m, 1H), 2.60–2.67 (m, 1H), 3.04 (s, 3H), 3.08 (s, 3H), 3.36 (d, *J* = 13.7, 1H), 3.88 (d, *J* = 13.7, 1H), 4.52–4.59 (m, 1H), 4.73 (d, *J* = 12.4, 1H), 4.97 (d, *J* = 12.3, 1H), 6.91–6.98 (m, 1H), 7.18–7.24 (m, 2H), 7.25–7.30 (m, 3H), 7.35–7.40 (m, 1H), 7.42–7.47 (m, 2H), 7.51–7.57 (m, 2H), 9.31 (s, 1H). ^13^C-NMR (126 MHz, CDCl_3_): δ 19.71, 20.51, 28.46, 28.68, 39.90, 44.00, 50.46, 51.29, 51.76, 52.02, 54.06, 68.41, 72.90, 117.34 (d, *J* = 295.3), 118.79, 122.19, 127.49, 128.82, 129.24, 130.75, 133.32, 139.93, 156.51, 161.52 (q, *J* = 33.5).

#### 3.6.8. *N*-Benzyl-1-[(1*R*,2*S*,4*R*)-7,7-dimethyl-2-(3-phenylureido)bicyclo[2.2.1]heptan-1-yl]-*N*,*N*-dimethylmethanaminium Iodide (**VIII**)

Following *GP5*. Prepared from catalyst **VII** (0.26 mmol, 139 mg), dissolved in ethyl acetate (5 mL), and filtered through a pad of NaI. All volatile components were evaporated in vacuo. Yield: 111 mg (0.21 mmol, 80%) of colorless solid, mp = 153–155 °C. [α]^r.t.^_D_ = +78 (0.073, MeOH). EI-HRMS: *m*/*z* = 406.2850 (M)^+^; C_26_H_36_N_3_O^+^ requires: *m*/*z* = 406.2853 (M)^+^; ν_max_ 3277, 2967, 2881, 1678, 1597, 1543, 1487, 1442, 1377, 1311, 1217, 1158, 1128, 1030, 949, 852, 816, 753, 729, 694 cm^−1^. ^1^H-NMR (500 MHz, CDCl_3_): δ 1.01 (s, 6H), 1.14 (dd, *J* = 13.3; 3.6, 1H), 1.46–1.60 (m, 2H), 1.62 (t, *J* = 4.5, 1H), 2.23–2.31 (m, 1H), 2.48–2.56 (m, 1H), 2.67–2.76 (m, 1H), 3.08 (s, 3H), 3.11 (s, 3H), 3.34 (d, *J* = 13.7, 1H), 4.07 (d, *J* = 13.6, 1H), 4.54–4.65 (m, 1H), 4.80 (d, *J* = 12.3, 1H), 5.05 (d, *J* = 12.3, 1H), 6.75 (d, *J* = 10.8, 1H), 6.94–7.00 (m, 1H), 7.19–7.25 (m, 2H), 7.31 (t, *J* = 7.6, 2H), 7.38–7.43 (m, 1H), 7.48–7.53 (m, 2H), 7.56–7.62 (m, 2H), 8.93 (s, 1H). ^13^C-NMR (126 MHz, CDCl_3_): δ 19.88, 20.81, 28.41, 29.13, 39.96, 44.02, 50.51, 51.57, 51.95, 52.34, 54.45, 68.01, 72.56, 118.82, 122.49, 127.33, 128.83, 129.29, 130.86, 133.38, 139.60, 156.34.

#### 3.6.9. *N*-Benzyl-1-{(1*R*,2*S*,4*R*)-2-[(2-{[3,5-bis(trifluoromethyl)phenyl]amino}-3,4-dioxocyclobut-1-en-1-yl)amino]-7,7-dimethylbicyclo[2.2.1]heptan-1-yl}-*N*,*N*-dimethylmethanaminium 2,2,2-Trifluoroacetate (**IX**)

Following *GP4*. Prepared from compound **7a** (0.19 mmol, 100 mg) and 3-((3,5-bis(trifluoromethyl)phenyl)amino)-4-ethoxycyclobut-3-ene-1,2-dione (0.30 mmol, 106.4 mg), CH_2_Cl_2_ (2 mL), 25 °C, 16 h. Isolation by evaporation followed by column chromatography (Silica gel 60, EtOAc/MeOH = 4:1). Yield: 106 mg (0.15 mmol, 75%) of colorless solid, mp = 148.9–150.1 °C. [α]^r.t.^_D_ = +65 (0.006, MeOH). EI-HRMS: *m*/*z* = 594.2545 (M)^+^; C_31_H_34_F_6_N_3_O_2_ requires: *m*/*z* = 594.2550 (M)^+^; ν_max_ 3420, 3153, 3034, 2967, 2888, 1791, 1686, 1603, 1551, 1475, 1427, 1377, 1276, 1176, 1127, 948, 931, 880, 848, 831, 730, 701, 684, 666 cm^−1^. ^1^H-NMR (500 MHz, CDCl_3_): δ 1.02 (s, 3H), 1.17 (s, 3H), 1.35 (dd, *J* = 13.2, 3.6, 1H), 1.60–1.66 (m, 1H), 1.77 (t, *J* = 4.5, 1H), 1.89 (br t, *J* = 13.3, 1H), 1.93–2.05 (m, 1H), 2.52–2.62 (m, 1H), 3.01 (s, 1H), 3.14 (s, 3H), 3.16 (s, 3H), 3.42 (d, *J* = 13.8, 1H), 4.33 (d, *J* = 13.9, 1H), 4.63 (d, *J* = 12.5, 1H), 4.76 (d, *J* = 12.5, 1H), 5.27 (t, *J* = 9.9, 1H), 7.41 (t, *J* = 7.4, 2H), 7.45–7.50 (m, 2H), 7.55–7.66 (m, 2H), 8.21 (s, 2H), 9.13 (d, *J* = 9.2, 1H), 11.34 (s, 1H). ^13^C-NMR (126 MHz, CDCl_3_): δ 19.80, 20.66, 26.61, 28.93, 41.29, 44.05, 50.80, 51.47, 52.79, 55.04, 58.56, 70.65, 73.17, 116.24, 119.01, 123.33 (q, *J* = 272.9), 126.85, 129.59, 131.38, 132.65 (q, *J* = 33.4), 133.40, 140.74, 165.81, 169.02, 181.07, 185.00 (two carbons missing).

#### 3.6.10. 1-Benzyl-1-{[(1*S*,2*S*,4*R*)-7,7-dimethyl-2-(3-phenylthioureido)bicyclo[2.2.1]heptan-1-yl]methyl}pyrrolidin-1-ium Iodide (**X**)

Following *GP4*. Prepared from compound (**7b**) (0.25 mmol, 109 mg) and phenyl isothiocyanate (0.38 mmol, 45 µL), CH_2_Cl_2_ (2 mL), 25 °C, 16 h. Isolation by column chromatography (Silica gel 60, EtOAc/MeOH = 10:1). Yield: 72 mg (0.13 mmol, 50%) of colorless solid, mp = 178–180 °C. [α]^r.t.^_D_ = +7.4 (0.14, MeOH). EI-HRMS: *m*/*z* = 448.2776 (M)^+^; C_28_H_38_N_3_S requires: *m*/*z* = 448.2781 (M)^+^; ν_max_ 3209, 3030, 2953, 1685, 1597, 1528, 1495, 1450, 1360, 1308, 1243, 1144, 1089, 1027, 1002, 915, 758, 716, 698, 607 cm^−1^. ^1^H-NMR (500 MHz, CDCl_3_): δ 0.99 (s, 3H), 1.02 (s, 3H), 1.12 (dd, *J* = 13.4, 3.9, 1H), 1.42–1.52 (m, 2H), 1.65 (t, *J* = 4.3, 1H), 1.71–1.89 (m, 2H), 1.96–2.07 (m, 1H), 2.15–2.23 (m, 2H), 2.53–2.64 (br t, *J* = 11.8, 1H), 2.97 (br s, 1H), 3.40 (d, *J* = 13.9, 1H), 3.42–3.48 (m, 1H), 3.70 (dt, *J* = 12.3, 7.4, 1H), 3.81 (d, *J* = 13.8, 1H), 3.83–3.99 (m, 2H), 4.64 (d, *J* = 12.7, 1H), 5.12 (d, *J* = 12.7, 1H), 5.40 (br t, *J* = 10.6, 1H), 7.14 (t, *J* = 7.3, 1H), 7.31 (t, *J* = 7.6, 2H), 7.41 (t, *J* = 7.4, 2H), 7.46 (t, *J* = 7.3, 1H), 7.60 (d, *J* = 6.8, 2H), 7.76 (d, *J* = 7.2, 2H), 8.10 (d, *J* = 10.4, 1H), 10.10 (s, 1H). ^13^C-NMR (126 MHz, CDCl_3_): δ 19.98, 20.70, 21.34, 22.00, 28.48, 29.80, 38.39, 43.70, 51.66, 54.42, 56.54, 60.24, 62.02, 63.82, 67.18, 123.99, 125.16, 127.84, 128.55, 129.49, 130.93, 133.61, 139.32, 182.27.

### 3.7. General Procedure for the α-Fluorination of β-Keto Ester **9**

Aqueous K_3_PO_4_ or other base (2 M, 2 equivalents, 0.1 mL) was added to a mixture of β-keto ester **9** (0.1 mmol, 24.4 mg, ω = 95%) and organocatalyst **I**–**IX** (2 mol%) in toluene or in CH_2_Cl_2_ (2 mL) under argon atmosphere. The mixture was cooled to −10℃ and NFSI (1.1 equivalents, 34.7 mg) was added in two portions over 2 h. The reaction mixture was stirred for another 12 h at −10 °C. After completion, the reaction was quenched by addition of NH_4_Cl (aq. sat, 4 mL) and extracted with CH_2_Cl_2_ (10 mL). The organic phase was dried over anhydrous Na_2_SO_4_, filtered, and the volatiles were evaporated in vacuo. The residue was purified by column chromatography (Silica gel 60, EtOAc/*n*-Heptane = 1:15). Enantiomeric excess (ee) was determined by HPLC (Chiralpak AD-H, *n*-Hexane/*i*-PrOH = 200:1, flow rate 0.75 mL/min, λ = 250 nm, 10 °C) after isolation by column chromatography.

### 3.8. General Procedure for the α-Chlorination of β-Keto Ester **9**

To a mixture of β-keto ester **9** (0.1 mmol, 24.4 mg, ω = 95%), organocatalyst **II**, **III**, **VI**–**VIII**, or **IX** (1 mol%), and K_2_HPO_4_ (solid, 1 equivalent, 17.4 mg) in chlorobenzene (2 mL), at −20 °C under argon atmosphere, was added *N*-chlorosuccinimide (NCS, 1.2 equivalents, 16 mg), and the reaction mixture was stirred for 2 h at −20 °C. After completion, the reaction was quenched by addition of NH_4_Cl (aq. sat, 4 mL) and extracted with CH_2_Cl_2_ (10 mL). The organic phase was dried over anhydrous Na_2_SO_4_, filtered, and the volatiles were evaporated in vacuo. The residue was purified by column chromatography (Silica gel 60, EtOAc/*n*-Heptane = 1:12). Enantiomeric excess (ee) was determined by HPLC (Chiralpak OJ-H, *n*-Hexane/*i*-PrOH = 70:30, flow rate 0.7 mL/min, λ = 250 nm, 10 °C) isolation with column chromatography (Silica gel 60, EtOAc/*n*-Heptane = 1:12).

### 3.9. General Procedure for the α-Hydroxylation of β-Keto Ester **9**

Into a flame-dried Schlenk flask under argon atmosphere at 0℃, a mixture of β-keto ester **9** (0.1 mmol, 24.4 mg, ω = 95%) and *N*-(4-bromobenzylidene)-4-methylbenzenesulfonamide (**12**) (1 equivalent, 33.8 mg) was added. Catalyst **III**, **IV**, **VII**, **VIII**, or **IX** (5 mol%) was dissolved in anhydrous methyl *tert*-butyl ether (MTBE, 5 mL) and slowly added via syringe into the reaction mixture. After addition of H_2_O_2_ (1 equivalent, 35% in water, 8.6 µL), the reaction mixture was stirred for 20 h at room temperature. After 24 h at 25 °C, the reaction mixture was filtrated trough a plug of anhydrous Na_2_SO_4_ and washed with dichloromethane.

### 3.10. General Procedure for the Ring-Opening of Aryl-Aziridine **14** with β-Keto Ester **9**

To a mixture of β-keto ester **9** (0.1 mmol, 24.4 mg, ω = 95%), catalyst **IV**, **VIII**, or **IX** (5 mol%), and K_3_PO_4_ (2 equivalents, 42 mg) in toluene (2.5 mL) under argon atmosphere, 2-phenyl-1-tosylaziridine (**14**) (2 equivalent, 54.6 mg) was added and stirred at room temperature for 24 h. After 24 h at 25 °C, the reaction mixture was filtrated through a plug of anhydrous Na_2_SO_4_ and washed with dichloromethane.

### 3.11. General Procedure for the Michael Addition of Glycine Schiff Base **16** with Methyl Acrylate (**17**)

Degassed solvent (2.5 mL) was added to a mixture of *tert*-butyl 2-((diphenylmethylene)amino)acetate (**16**) (0.05 mmol, 14.8 mg), catalyst **I**, **III**–**V**, **VII**, **VIII**, or **IX** (10 mol%), and Cs_2_CO_3_ (1.5–10.0 equivalents) in a Schlenk tube at 25 °C or 0 °C; then, methyl acrylate (**17**) (1.5 equivalents, 6.8 µL) was added. After 24 h at 25 °C or 0 °C, the reaction mixture was filtrated through a plug of anhydrous Na_2_SO_4_ and washed with ethyl acetate. The volatiles were evaporated in vacuo. The crude product **18** was purified by column chromatography (Silica gel 60, EtOAc/Heptane = 1:15). Enantiomeric excess (ee) was determined by HPLC (Chiralpak AD-H, *n*-Hexane/*i*-PrOH = 95:5, flow rate 0.5 mL/min, λ = 250 nm, 10 °C) after filtration of the reaction mixture through a plug of Na_2_SO_4_.

### 3.12. X-ray Crystallography

Single-crystal X-ray diffraction data were collected on an Agilent Technologies SuperNova Dual diffractometer with an Atlas detector using monochromated Mo-K_α_ radiation (λ = 0.71073 Å) at 150 K. The data were processed using CrysAlis PRO [37]. Using Olex2.1.2. [38], the structures were solved by direct methods implemented in SHELXS [39] or SHELXT [40] and refined by a full-matrix least-squares procedure based on F^2^ with SHELXT-2014/7 [41]. All nonhydrogen atoms were refined anisotropically. Hydrogen atoms were placed in geometrically calculated positions and were refined using a riding model. The drawings and the analysis of bond lengths, angles, and intermolecular interactions were carried out using Mercury [42] and Platon [43]. Structural and other crystallographic details on data collection and refinement for compounds **VI**-**Br** and **III** have been deposited with the Cambridge Crystallographic Data Centre as a supplementary publication under CCDC Deposition Numbers 2204647 and 2204648, respectively. These data can be obtained free of charge via www.ccdc.cam.ac.uk/conts/retrieving.html (or from the CCDC, 12 Union Road, Cambridge CB2 1EZ, UK; fax: +44 1223 336033; e-mail: deposit@ccdc.cam.ac.uk).

## 4. Conclusions

Ten novel quaternary ammonium salt bifunctional phase-transfer organocatalysts based on a chiral (+)-camphor framework were prepared. Starting from camphor-derived 1,3-diamines, catalysts **I**–**X** were synthesized in a four-step sequence: Boc protection–benzylation–Boc deprotection–reaction with iso(thio)cyanate/squaramate. The catalysts prepared bear either a (thio)urea or squaramide hydrogen bond donor and possess either a trifluoroacetate or iodide counteranion. The quaternary iodide ammonium salts **II**, **V**, and **VIII** were formed from the corresponding trifluoroacetates **I**, **IV**, and **VII**, respectively, via anion methathesis with an excess of NaI. The phase-transfer catalysts have been fully characterized; the stereochemistry at the C-2 chiral center was unambiguously determined. Their organocatalytic activity was investigated in the electrophilic functionalization of the β-keto ester **9**. The α-fluorination and chlorination of β-keto ester **9** proceeded to full conversion, affording the desired products **10** and **11** with low enantioselectivity (up to 29% ee). α-Hydroxylation and ring opening of *N*-tosylaziridine **14** gave no product. Finally, the Michael addition of glycine Schiff base **16** to methyl acrylate (**17**) gave the expected product **18** with full conversion and up to 11% ee.

## Data Availability

Not applicable.

## Supplementary Materials

The following supporting information can be downloaded at: https://www.mdpi.com/article/10.3390/molecules28031515/s1. Synthesis and characterization data; HPLC data; copies of ^1^H- and ^13^C-NMR spectra; copies of HRMS reports; structure determination by X-ray diffraction analysis. Figure S1. Applied organocatalysts **I**–**IX**. Figure S2. Molecular structure of compound **VI-Br**. Thermal ellipsoids are shown at 50% probability. Figure S3. Molecular structure of compound **III**. Thermal ellipsoids are shown at 50% probability. Table S1. Evaluation of organocatalysts **I**–**IX** in the fluorination of β-keto ester **9**. Table S2. Further evaluation of organocatalysts **III**, **VIII**, and **IX** in the fluorination of β-keto ester **9**. Table S3. Evaluation of organocatalysts **II**, **III**, **VI**–**VIII**, and **IX** in the chlorination of β-keto ester **9**. Table S4. Evaluation of organocatalysts **III**, **IV**, **VII**, **VIII**, and **IX** in the hydroxylation of β-keto ester **9**. Table S5. Evaluation of organocatalysts **IV**, **VIII**, and **IX** in the addition of β-keto ester **9** to tosylaziridine **14**. Table S6. Evaluation of organocatalysts **I**, **III**–**V**, **VII**, **VIII**, and **IX** in the addition of *tert*-butyl 2-((diphenylmethylene)amino)acetate (**16**) to methyl acrylate (**17**). Table S7. Correlation between the multiplicity of the H–C(3)-*endo* proton (He) and the *endo* absolute configuration at the C-2 chiral center of compounds **1a**, **3b**, **5a**, **7a**,**b**, and **III**–**X**. Table S8. Crystal data and structure refinement for compound **VI-Br**. Table S9. Crystal data and structure refinement for compound **III**.

## Sample Availability

Samples of the compounds are available from the authors.
