# Supplementary material for: Synthesis and Catalytic Activity of Bifunctional Phase-Transfer Organocatalysts Based on Camphor
Source: Molecules. 2023 Feb 3;28(3):1515. doi: 10.3390/molecules28031515 (PMC9921693; doi:10.3390/molecules28031515)
Supplement: Supplementary file 1 [file molecules-28-01515-s001.zip › molecules-2200743-supplementary.pdf]

SUPPORTING INFORMATION

Synthesis and Catalytic Activity of Bifunctional Phase-Transfer Organocatalysts Based on Camphor

Luka Ciber,[1] Franc Požgan,[1] Helena Brodnik,[1] Bogdan Štefane,[1] Jurij Svete,[1] Mario Waser,[2] and Uroš Grošelj*[1]

[1] Chair of Organic Chemistry, Faculty of Chemistry and Chemical Technology, University of Ljubljana, Večna pot 113, 1000 Ljubljana, Slovenia; E-mail: uros.groselj@fkkt.uni-lj.si

[2] Institute of Organic Chemistry, Johannes Kepler University Linz, Altenbergerstrasse 69, 4040 Linz, Austria; E-mail: mario.waser@jku.at; https://www.jku.at/orc/waser

Table of contents

1. Materials and methods, syntheses, and characterization 2
2. HPLC data 28
3. Copies of ^{1}H- and ^{13}C-NMR spectra 69
4. Determination of the absolute configuration at the C-2 chiral center 101
5. Copies of HRMS reports of products 102
6. X-Ray crystallography 123
7. References 128

1. Materials and methods, syntheses, and characterization

Solvents for extractions and chromatography were of technical grade and were distilled prior to use. Extracts were dried over technical grade anhydrous Na_2SO_4. Melting points were determined on a Kofler micro hot stage. The NMR spectra were obtained on a Bruker Avance DPX 300 and Bruker Avance III 300 at 300 MHz for ^1H nucleus, Bruker UltraShield 500 plus (Bruker, Billerica, Massachusetts, United States) at 500 MHz for ^1H and 126 MHz for ^{13}C nucleus, and Bruker Ascend 600 (Bruker, Billerica, Massachusetts, United States) at 600 MHz for ^1H and 151 MHz for ^{13}C nucleus using DMSO-d_6 and $CDCl_3$ with TMS as the internal standard, as solvents. Mass spectra were recorded on an Agilent 6224 Accurate Mass TOF LC/MS (Agilent Technologies, Santa Clara, California, United States), IR spectra on a Perkin-Elmer Spectrum BX FTIR spectrophotometer (PerkinElmer, Waltham, Massachusetts, United States). CD spectra were recorded on a J-1500 Circular Dichroism Spectrophotometer (JASCO corporation, Tokyo, Japan). Column chromatography (CC) was performed on silica gel (Silica gel 60, particle size: 0.035-0.070 mm (Sigma-Aldrich, St. Louis, Missouri, United States)). HPLC analyses were performed on an Agilent 1260 Infinity LC (Agilent Technologies, Santa Clara, California, United States) and Dionex Summit HPLC system (Dionex Corporation, Sunnyvale, California, United States) using CHIRALPAK AD-H (0.46 cm ø × 25 cm) and CHIRALPAK OJ-H (0.46 cm ø × 25 cm), as chiral column (Chiral Technologies, Inc., West Chester, Pennsylvania, United States). All the commercially available chemicals used were purchased from Sigma-Aldrich (St. Louis, Missouri, United States). (1*S*,2*S*,4*R*)-7,7-dimethyl-1-(pyrrolidin-1-ylmethyl)bicyclo[2.2.1]heptan-2-amine (**1b**) was prepared following the literature procedure[1].

Reduction of (1*S*,4*R*,*E*)-1-((dimethylamino)methyl)-7,7-dimethylbicyclo[2.2.1]heptan-2-one oxime

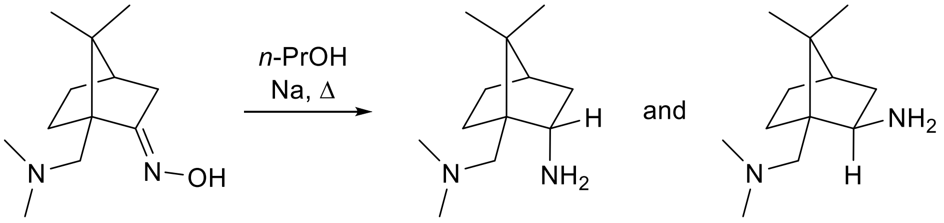

1a **2a**

Oxime (7.6 mmol, 1.6 g) was dissolved in propan-1-ol (86 mL) and heated to 95 °C. Then small pieces of sodium (about 50 mg) were added continuously for 1 h at 95 °C; care was taken to ensure that the unreacted sodium (excess sodium) remained present in the reaction mixture at all times during the reaction. After completion of the reaction, the volatiles were evaporated *in vacuo*. The residue was dissolved in a mixture of water (20 mL) and Et_2O (80 mL). The organic phase was washed with water (2 × 20 mL) and NaCl (aq. sat., 1 × 20 mL), dried over anhydrous Na_2SO_4, and the volatiles were evaporated *in vacuo*. Diastereomers **1a** and **2a** were formed in a ratio of 2.6 : 1. The diastereomers were separated by column chromatography (Silica gel 60, EtOAc/MeOH/Et_3N = 4 : 1 : 1).

(1*S*,2*R*,4*R*)-1-((Dimethylamino)methyl)-7,7-dimethylbicyclo[2.2.1]heptan-2-amine (2a)

Elutes first from the column. Yield: 175 mg (0.89 mmol, 12%) of colorless oil. ^{1}H-NMR (500 MHz, $CDCl_3$): δ 0.79 (*s*, 3H), 1.05 (*s*, 3H), 1.06 – 1.13 (*m*, 1H), 1.34 (*ddd*, *J* = 12.8, 9.4, 3.9, 1H), 1.54 – 1.61 (*m*, 3H), 1.63 (*t*, *J* = 4.3, 1H), 1.64 – 1.75 (*m*, 2H), 1.93 (*d*, *J* = 11.4, 1H), 2.02 (*d*, *J* = 13.0, 1H), 2.27 (*s*, 6H), 2.74 (*d*, *J* = 13.0, 1H), 3.11 (*dd*, *J* = 8.7, 5.1, 1H).

(1*S*,2*S*,4*R*)-1-((Dimethylamino)methyl)-7,7-dimethylbicyclo[2.2.1]heptan-2-amine (1a)

Elutes second from the column. Yield: 850 mg (4.33 mmol, 57%) of colorless oil. ^{1}H-NMR (500 MHz, $CDCl_3$): δ 0.67 (*dd*, *J* = 12.9, 4.3, 1H), 0.86 (*s*, 3H), 0.89 (*s*, 3H), 1.22 (*ddd*, *J* = 12.3, 9.5, 4.4, 1H), 1.38 (*ddd*, *J* = 12.3, 4.5, 2.0, 1H), 1.49 (*t*, *J* = 4.6, 1H), 1.70 – 1.79 (*m*, 1H), 1.80 (br *s*, 2H), 2.10 (*d*, *J* = 13.1, 1H), 2.13 – 2.17 (*m*, 1H), 2.20 (*s*, 6H), 2.21 – 2.26 (*m*, 1H), 2.45 (*d*, *J* = 13.0, 1H), 3.36 (*ddd*, *J* = 10.6, 4.3, 2.0, 1H).

Boc protection of chiral amines – *General procedure 1* (*GP1*)

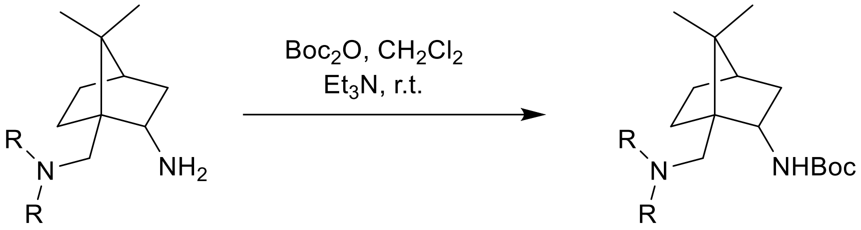

To a solution of amine **1** or **2** and triethylamine (1.4 equivalents) in anhydrous CH_2Cl_2 was added di-*tert*-butyl dicarbonate (1.4 equivalents). The resulting reaction mixture was stirred at 25°C for 24 hours. Dichloromethane was evaporated *in vacuo* and the residue was purified by column chromatography (CC). The fractions containing product **3** or **4** were combined and the volatiles were evaporated *in vacuo*.

tert-Butyl ((1_S_,2_S_,4_R_)-1-((dimethylamino)methyl)-7,7-dimethylbicyclo[2.2.1]heptan-2-yl)carbamate (3a)

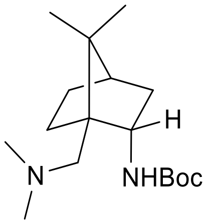

Following _GP1_. Prepared from (1_S_,2_S_,4_R_)-1-((dimethylamino)methyl)-7,7-dimethylbicyclo[2.2.1]heptan-2-amine (**1a**) (4.69 mmol, 920 mg) and di-_tert_-butyl dicarbonate (6.56 mmol, 1.431 g), Et_3N (6.56 mmol, 915 µL), CH_2Cl_2 (20 mL), 25°C, 24 h. Isolation by column chromatography (Silica gel 60, EtOAc/petroleum ether = 1 : 5). Yield: 1.39 g (4.69 mmol, 99%) of colorless oil. $[\alpha]_D^{r.t.}$ = +11.2 (0.15, MeOH). EI-HRMS: _m/z_ = 297.2646 $(MH)^+$; $C_{17}H_{33}N_2O_2^+$ requires: _m/z_ = 297.2536 $(MH)^+$; ν_{max} 3346, 2935, 2819, 2765, 1698, 1483, 1454, 1389, 1364, 1297, 1242, 1167, 1114, 1065, 1040, 1014, 946, 874, 837, 780 cm^{-1}. ^1H-NMR (500 MHz, $CDCl_3$): δ 0.86 (_s_, 3H), 0.90 (_s_, 3H), 1.04 (_dd_, _J_ = 13.4, 4.3, 1H), 1.21 (_ddd_, _J_ = 12.2, 9.5, 4.4, 1H), 1.43 (_s_, 9H), 1.45 – 1.51 (m, 1H), 1.56 (_t_, _J_ = 4.6, 1H), 1.72 (tq, _J_ = 12.1, 4.1, 1H), 1.86 (br _t_, 1H), 2.21 (_s_, 6H), 2.24 (_d_, _J_ = 13.6, 1H), 2.28 – 2.33 (_m_, 1H), 2.36 (_d_, _J_ = 13.8, 1H), 3.75 (_s_, 1H), 6.00 (_s_, 1H). ^{13}C-NMR (126 MHz, $CDCl_3$): δ 19.19, 20.31, 25.40, 28.39, 28.64, 37.92, 45.07, 48.10, 48.33, 50.98, 56.25, 61.97, 78.72, 157.52.

tert-Butyl ((1_S_,2_R_,4_R_)-1-((dimethylamino)methyl)-7,7-dimethylbicyclo[2.2.1]heptan-2-yl)carbamate (4a)

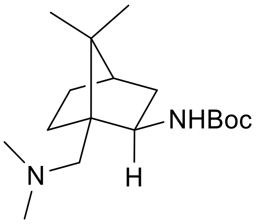

Following *GP1*. Prepared from (1*S*,2*R*,4*R*)-1-((dimethylamino)methyl)-7,7-dimethylbicyclo[2.2.1]heptan-2-amine (**2a**) (0.81 mmol, 160 mg) and di-*tert*-butyl dicarbonate (1.134 mmol, 247 mg), Et_3N (1.19 mmol, 166 µL), CH_2Cl_2 (4 mL), 25°C, 24 h. Isolation by column chromatography (Silica gel 60, EtOAc/petroleum ether = 1 : 5). Yield: 230 mg (0.78 mmol, 95%) of colorless oil. $[\alpha]_D^{r.t.}$ = +25.7 (0.175, MeOH). EI-HRMS: *m/z* = 297.2536 $(MH)^+$; $C_{17}H_{33}N_2O_2^+$ requires: *m/z* = 297.2537 $(MH)^+$; ν_{max} 3344, 2935, 2819, 2765, 1698, 1484, 1453, 1389, 1364, 1297, 1243, 1167, 1113, 1065, 1040, 1004, 943, 874, 837, 780 cm^{-1}. ^{1}H-NMR (500 MHz, $CDCl_3$): δ 0.87 (*s*, 3H), 0.99 (*s*, 3H), 1.09 – 1.17 (*m*, 1H), 1.34 (*t*, *J* = 9.4, 1H), 1.42 – 1.45 (*m*, 1H), 1.43 (*s*, 9H), 1.67 (*d*, *J* = 3.5, 2H), 1.69 – 1.75 (*m*, 1H), 1.86 (*d*, *J* = 8.4, 1H), 2.24 (*s*, 6H), 2.25 (*d*, *J* = 13.9, 1H), 2.40 (*d*, *J* = 13.9, 1H), 3.71 (br *s*, 1H), 5.58 (br *s*, 1H). ^{13}C-NMR (126 MHz, $CDCl_3$): δ 20.95, 27.15, 28.50, 28.67, 30.48, 33.79, 40.55, 45.67, 48.03, 50.94, 57.44, 58.86, 78.90, 155.72.

tert-Butyl ((1S,2S,4R)-7,7-dimethyl-1-(pyrrolidin-1-ylmethyl)bicyclo[2.2.1]heptan-2-yl)carbamate (3b)

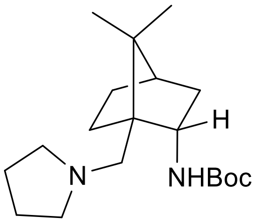

Following *GP1*. Prepared from (1*S*,2*S*,4*R*)-7,7-dimethyl-1-(pyrrolidin-1-ylmethyl)bicyclo[2.2.1]heptan-2-amine (**1b**) (3.91 mmol, 869 mg) and di-*tert*-butyl dicarbonate (5.474 mmol, 1.194 g), Et_3N (5.474 mmol, 763 µL), CH_2Cl_2 (20 mL), 25°C, 24 h. Isolation by column chromatography (Silica gel 60, EtOAc/petroleum ether = 1 : 5). Yield: 1.251 g (3.88 mmol, 99%) of brownish oil. $[\alpha]_D^{r.t.}$ = +1.1 (0.295, MeOH). EI-HRMS: *m/z* = 323.2688 $(MH)^+$; $C_{19}H_{35}N_2O_2^+$ requires: *m/z* = 323.2693 $(MH)^+$; ν_{max} 3300, 2979, 2937, 2879, 2794, 1808, 1757, 1715, 1460, 1395, 1371, 1306, 1250, 1211, 1168, 1113, 1062, 950, 844, 775, 664 cm^{-1}. ^{1}H-NMR (600 MHz, $CDCl_3$): δ 0.85 (*s*, 3H), 0.90 (*s*, 3H), 1.07 (*dd*, *J* = 13.4, 4.4, 1H), 1.21 (*ddd*, *J* = 12.8, 9.5, 4.5, 1H), 1.4 – 1.45 (*m*, 1H), 1.41 (*s*, 9H), 1.56 (*t*, *J* = 4.6, 1H), 1.65 – 1.73 (*m*, 6H), 1.90 (*ddd*, *J* = 13.6, 8.9, 4.1, 1H), 2.31 (*s*, 1H), 2.37 (*d*, *J* = 13.4, 1H), 2.41 – 2.46 (*m*, 2H), 2.56 – 2.60 (*m*, 2H), 2.66 (*d*, *J* = 13.4, 1H), 3.72 (br *s*, 1H), 6.31 (br *s*, 1H). ^{13}C-NMR (151 MHz, $CDCl_3$): δ 19.18, 20.23, 24.16, 26.03, 28.43, 28.61, 37.58, 45.22, 47.88, 50.81, 56.47, 56.82, 58.03, 78.48, 157.80.

Benzylation of tertiary amines – *General procedure 2* (*GP2*)

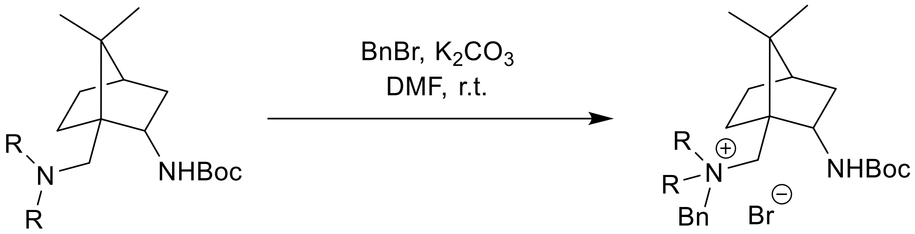

To a solution of tertiary amine **3** or **4** and K_2CO_3 (1.1 equivalents) in anhydrous DMF was added benzyl bromide (1.1 equivalents). The resulting reaction mixture was stirred at 25°C for 24 hours. DMF was evaporated *in vacuo* and the residue was purified by column chromatography (CC). The fractions containing product **5** or **6** were combined and the volatiles were evaporated *in vacuo*.

N-Benzyl-1-((1S,2S,4R)-2-((tert-butoxycarbonyl)amino)-7,7-dimethylbicyclo[2.2.1]heptan-1-yl)-N,N-dimethylmethanaminium bromide (5a)

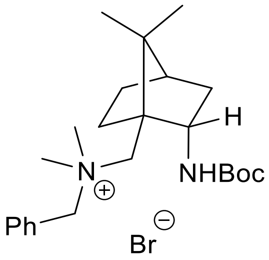

Following *GP2*. Prepared from *tert*-butyl ((1*S*,2*S*,4*R*)-1-((dimethylamino)methyl)-7,7-dimethylbicyclo[2.2.1]heptan-2-yl) (**3a**) (1.06 mmol, 315 mg) and benzyl bromide (1.16 mmol, 139 µL), K_2CO_3 (1.16 mmol, 160 mg), DMF (5 mL), 25°C, 24 h. Isolation by column chromatography (Silica gel 60, EtOAc/MeOH = 4 : 1). Yield: 340 mg (0.73 mmol, 69%) of colorless oil. $[\alpha]_D^{r.t.}$ = +14.0 (0.087, MeOH). EI-HRMS: *m/z* = 387.3003 $(M)^+$; $C_{24}H_{39}N_2O_2$ requires: *m/z* = 387.3006 $(M)^+$; ν_{max} 3369, 3197, 2951, 2199, 2163, 2098, 1989, 1685, 1540, 1490, 1477, 1454, 1392, 1379, 1366, 1299, 1284, 1271, 1252, 1217, 1158, 1125, 1065, 1042, 1012, 947, 917, 882, 868, 854, 839, 783, 752, 732, 706 cm^{-1}. ^{1}H-NMR (500 MHz, $CDCl_3$): δ 0.87 – 0.93 (*m*, 1H), 0.94 (*s*, 3H), 0.98 (*s*, 3H), 1.29 – 1.34 (*m*, 1H), 1.36 (*s*, 9H), 1.58 (*t*, *J* = 4.4, 1H), 1.86 (br *t*, *J* = 11.7, 1H), 1.93 – 2.03 (*m*, 1H), 2.21 (br *t*, *J* = 13.1, 1H), 2.47 (*d*, *J* = 11.7, 1H), 3.17 (*s*, 3H), 3.25 (*s*, 3H), 3.44 (br *d*, *J* = 13.6, 1H), 4.11 (br *d*, *J* = 13.5, 1H), 4.18 (br *t*, *J* = 9.9, 1H), 4.97 (*d*, *J* = 12.3, 1H), 5.03 (br *s*, 1H), 5.20 (*d*, *J* = 12.2, 1H), 7.32 – 7.43 (*m*, 3H), 7.61 (*d*, *J* = 7.4, 2H). ^{13}C-NMR (126 MHz, $CDCl_3$): δ 19.48, 20.76, 27.34, 28.28, 28.75, 40.19, 43.67, 50.40, 51.55, 53.72, 54.15, 69.24, 70.42, 80.67, 127.78, 128.94, 130.51, 133.47, 156.14.

N-Benzyl-1-((1S,2R,4R)-2-((tert-butoxycarbonyl)amino)-7,7-dimethylbicyclo[2.2.1]heptan-1-yl)-N,N-dimethylmethanaminium bromide (6a)

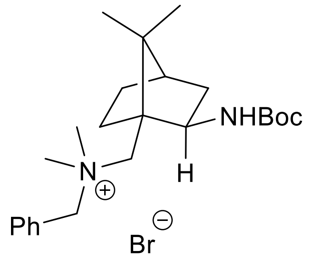

Following *GP2*. Prepared from *tert*-butyl ((1*S*,2*R*,4*R*)-1-((dimethylamino)methyl)-7,7-dimethylbicyclo[2.2.1]heptan-2-yl)carbamate (**4a**) (2.53 mmol, 748 mg) and benzyl bromide (3.795 mmol, 453 µL), K_2CO_3 (2.78 mmol, 385 mg), DMF (13 mL), 25°C, 24 h. Isolation by column chromatography (Silica gel 60, EtOAc/MeOH = 4 : 1). Yield: 904 mg (1.93 mmol, 76%) of colorless oil. $[\alpha]_D^{r.t.}$ = –4.3 (0.26, MeOH). EI-HRMS: *m/z* = 387.3004 $(M)^+$; $C_{24}H_{39}N_2O_2^+$ requires: *m/z* = 387.3006 $(M)^+$; ν_{max} 3341, 2965, 2885, 2156, 1698, 1606, 1508, 1475, 1456, 1365, 1278, 1247, 1168, 1060, 1019, 953, 860, 782, 732, 706 cm^{-1}. ^{1}H-NMR (500 MHz, $CDCl_3$): δ 0.92 (*dd*, *J* = 13.4, 3.5, 1H), 0.97 (*s*, 3H), 1.03 (*s*, 3H), 1.35 (*t*, *J* = 4.8, 1H), 1.39 (*s*, 9H), 1.62 (*t*, *J* = 4.5, 1H), 1.88 – 1.99 (*m*, 2H), 2.29 (br *t*, *J* = 12.9, 1H), 2.46 – 2.56 (*m*, 1H), 3.19 (*s*, 3H), 3.27 (*s*, 3H), 3.43 (br *d*, *J* = 14.3, 1H), 4.16 – 4.26 (*m*, 2H), 4.92 (*d*, *J* = 10.9, 1H), 4.97 (*d*, *J* = 12.4, 1H), 5.21 (*d*, *J* = 12.4, 1H), 7.37 – 7.46 (*m*, 3H), 7.63 (*d*, *J* = 6.9, 2H). ^{13}C-NMR (126 MHz, $CDCl_3$): δ 19.60, 20.95, 27.47, 28.40, 28.91, 40.49, 43.82, 50.54, 51.73, 53.85, 54.30, 69.39, 70.63, 80.94, 127.79, 129.12, 130.72, 133.57, 156.18.

1-Benzyl-1-(((1*S*,2*S*,4*R*)-2-((*tert*-butoxycarbonyl)amino)-7,7-dimethylbicyclo[2.2.1]heptan-1-yl)methyl)pyrrolidin-1-ium bromide (5b)

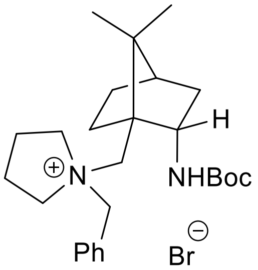

Following *GP2*. Prepared from *tert*-butyl ((1*S*,2*S*,4*R*)-7,7-dimethyl-1-(pyrrolidin-1-ylmethyl)bicyclo[2.2.1]heptan-2-yl)carbamate (**3b**) (2.48 mmol, 828 mg) and benzyl bromide (2.73 mmol, 324 µL), K_2CO_3 (2.73 mmol, 377 mg), DMF (13 mL), 25°C, 24 h. Isolation by column chromatography (Silica gel 60, EtOAc/MeOH = 4 : 1). Yield: 469 mg (1.45 mmol, 59%) of brownish semisolid. $[\alpha]_D^{r.t.}$ = +17.7 (0.12, MeOH). EI-HRMS: *m/z* = 413.3161 $(M)^+$; $C_{26}H_{41}N_2O_2^+$ requires: *m/z* = 413.3162 $(M)^+$; ν_{max} 3323, 3270, 2965, 2923, 1708, 1639, 1531, 1452, 1388, 1363, 1307, 1247, 1159, 1121, 1066, 1028, 1002, 923, 901, 855, 839, 780, 710 cm^{-1}. ^{1}H-NMR (500 MHz, $CDCl_3$): δ 0.92 (*s*, 3H), 0.91 – 0.96 (*m*, 1H), 0.96 (*s*, 3H), 1.32 – 1.35 (br *t*, 1H), 1.37 (*s*, 9H), 1.55 (*t*, *J* = 4.5, 1H), 1.62 – 1.73 (*m*, 1H), 1.74 – 1.86 (*m*, 2H), 1.88 – 2.04 (*m*, 2H), 2.04 – 2.13 (*m*, 1H), 2.17 – 2.27 (*m*, 1H), 2.39 – 2.48 (*m*, 1H), 3.39 (*d*, *J* = 14.0, 1H), 3.41 – 3.50 (*m*, 1H), 3.58 – 3.74 (*m*, 2H), 3.96 (*ddd*, *J* = 12.3, 8.1, 6.3, 1H), 4.13 (*d*, *J* = 14.0, 1H), 4.24 (*tt*, *J* = 10.8, 3.1, 1H), 4.59 (*d*, *J* = 12.6, 1H), 5.22 (br *s*, 1H), 5.26 (*d*, *J* = 10.8, 1H), 7.28 – 7.39 (*m*, 3H), 7.57 (*d*, *J* = 7.0, 2H). ^{13}C-NMR (126 MHz, $CDCl_3$): δ 19.33, 20.70, 21.60, 22.07, 27.88, 28.29, 28.81, 39.70, 43.63, 51.48, 53.44, 53.74, 59.55, 62.11, 63.58, 67.05, 80.48, 128.21, 128.99, 130.40, 133.30, 156.03.

Boc deprotection of amines – *General procedure 3* (*GP3*)

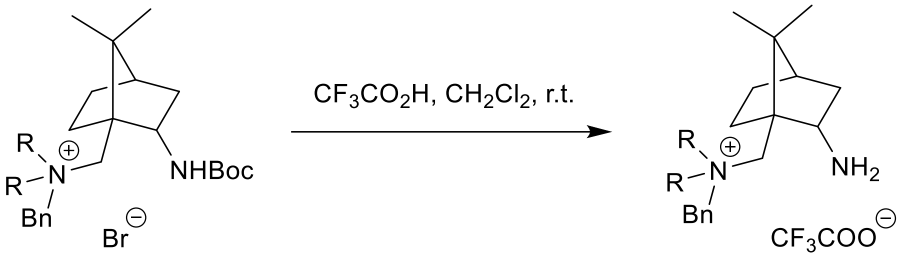

To a solution of amine **5** or **6** in anhydrous CH_2Cl_2 (2.5 mL/mmol) was added trifluoroacetic acid (2.5 mL/mmol). The resulting reaction mixture was stirred at 25°C for 2 hours. Dichloromethane and trifluoroacetic acid were evaporated *in vacuo* and the residue was dissolved in CH_2Cl_2 (2.5 mL/mmol). The organic phase was washed with NaOH (aq., 2 M, 2 × 2.5 mL/mmol) and NaCl (aq. sat., 1 × 2.5 ml/mmol). The volatiles were evaporated *in vacuo* to give product **7** or **8**.

(1*S*,2*S*,4*R*)-1-((Benzyldimethylammonio)methyl)-7,7-dimethylbicyclo[2.2.1]heptan-2-aminium 2,2,2-trifluoroacetate (7a)

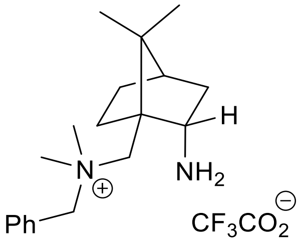

Following *GP3*. Prepared from *N*-benzyl-1-((1*S*,2*S*,4*R*)-2-((*tert*-butoxycarbonyl)amino)-7,7-dimethylbicyclo[2.2.1]heptan-1-yl)-*N*,*N*-dimethylmethanaminium bromide (**5a**) (2.1 mmol, 1 g), trifluoroacetic acid (5 mL), CH_2Cl_2 (5 mL), 25°C, 2 h. Volatile components were evaporated *in vacuo*, the residue was dissolved in dichloromethane and washed with NaOH (aq., 2 M) and NaCl (aq. sat.). Yield: 605 mg (1.51 mmol, 72%) of colorless solid, mp = 179.9–182.1°C. $[\alpha]_D^{r.t.}$ = +16.2 (0.125, MeOH). EI-HRMS: *m/z* = 287.2483 $(M)^+$; $C_{19}H_{31}N_2^+$ requires: *m/z* = 287.2482 $(M)^+$; ν_{max} 3377, 3292, 3042, 2943, 2881, 1685, 1585, 1479, 1457, 1401, 1372, 1302, 1196, 1157, 1113, 1048, 1025, 1010, 989, 935, 917, 881, 854, 819, 780, 785, 753, 733, 716, 707, 632, 607 cm^{-1}. ^{1}H-NMR (500 MHz, $CDCl_3$): δ 0.89 (*dd*, *J* = 3.4, 13.1, 1H); 0.93 (*s*, 3H); 0.97 (*s*, 3H); 1.31 – 1.38 (*m*, 1H); 1.61 (*t*, *J* = 4.6, 1H); 1.77 – 1.84 (*m*, 1H); 1.86 – 1.96 (*m*, 1H); 2.05 – 2.13 (*m*, 1H); 2.44 – 2.53 (*m*, 1H); 3.28 (*s*, 3H); 3.37 (*s*, 3H); 3.43 – 3.49 (*m*, 1H); 3.68 (*d*, *J* = 13.9, 1H); 3.72 (*d*, *J* = 13.9, 1H); 4.97 (*d*, *J* = 12.4, 1H); 5.11 (*d*, *J* = 12.3, 1H); 7.40 – 7.48 (*m*, 3H); 7.56 – 7.60 (*m*, 2H), signal for NH_2 is missing. ^{13}C-NMR (126 MHz, $CDCl_3$): δ 19.41, 20.51, 26.70, 29.29, 44.19, 44.63, 49.97, 50.49, 52.44, 53.20, 53.69, 69.11, 71.98, 117.64 (*q*, *J* = 297.3), 128.29, 129.14, 130.57, 133.49, 161.16 (*q*, *J* = 32.7).

(1*S*,2*R*,4*R*)-1-((Benzyldimethylammonio)methyl)-7,7-dimethylbicyclo[2.2.1]heptan-2-aminium 2,2,2-trifluoroacetate (8a)

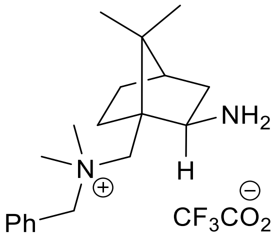

Following *GP3*. Prepared from *N*-benzyl-1-((1*S*,2*R*,4*R*)-2-((*tert*-butoxycarbonyl)amino)-7,7-dimethylbicyclo[2.2.1]heptan-1-yl)-*N*,*N*-dimethylmethanaminium bromide (**6a**) (1.92 mmol, 900 mg), trifluoroacetic acid (5 mL), CH_2Cl_2 (5 mL), 25°C, 2 h. Volatile components were evaporated *in vacuo*, the residue was dissolved in dichloromethane and washed with NaOH (aq., 2 M) and NaCl (aq. sat.). Yield: 567 mg (1.41 mmol, 74%) of colorless solid, mp = 157.1–158.8°C. $[\alpha]_D^{r.t.}$ = –9.8 (0.11, MeOH). EI-HRMS: *m/z* = 287.2477 $(M)^+$; $C_{19}H_{31}N_2^+$ requires: *m/z* = 287.2482 $(M)^+$; ν_{max} 2953, 2883, 1684, 1476, 1456, 1393, 1371, 1311, 1196, 1153, 1113, 1035, 1009, 936, 911, 851, 820, 798, 784, 735, 714, 631 cm^{-1}. ^{1}H-NMR (600 MHz, $CDCl_3$): δ 0.86 (*s*, 3H), 0.90 (*s*, 3H), 1.15 – 1.22 (*m*, 1H), 1.34 – 1.41 (*m*, 1H), 1.50 – 1.55 (*m*, 1H), 1.67 (*s*, 1H), 1.77 (*d*, *J* = 13.9, 3H), 1.92 (*dd*, *J* = 13.0, 7.3, 2H), 3.01 (*dd*, *J* = 9.0, 4.9, 1H), 3.19 (*s*, 6H), 3.33 (*d*, *J* = 13.5, 1H), 4.14 (*d*, *J* = 13.5, 1H), 4.75 (*d*, *J* = 12.5, 1H), 4.86 (*d*, *J* = 12.8, 1H), 7.35 – 7.41 (*m*, 3H), 7.56 (*d*, *J* = 7.4, 2H). ^{13}C-NMR (126 MHz, $CDCl_3$): δ 20.58, 21.40, 27.73, 32.27, 44.01, 44.29, 50.60, 51.35, 51.53, 52.44, 57.11, 64.64, 71.26, 117.56 (*q*, *J* = 296.8), 127.79, 129.26, 130.81, 133.53, 161.25 (*q*, *J* = 32.7).

Synthesis of 1-(((1*S*,2*S*,4*R*)-2-ammonio-7,7-dimethylbicyclo[2.2.1]heptan-1-yl)methyl)-1-benzylpyrrolidin-1-ium iodide (7b)

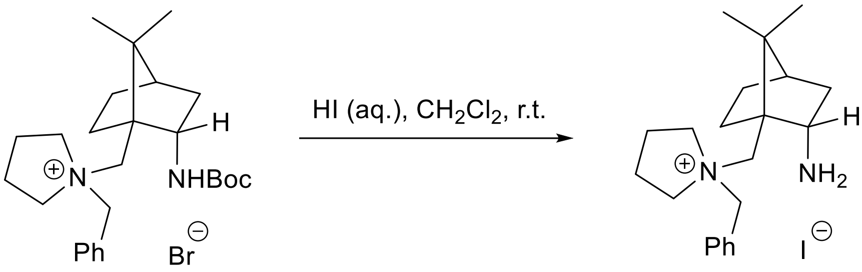

1-Benzyl-1-(((1*S*,2*S*,4*R*)-2-((*tert*-butoxycarbonyl)amino)-7,7-dimethylbicyclo[2.2.1]heptan-1-yl)methyl)pyrrolidin-1-ium bromide (**5b**) (0.55 mmol, 270 mg) was dissolved in anhydrous CH_2Cl_2 (8 mL), then HI (aq., 48%, 5 equvivlents, 2.75 mmol, 495 µL) was added. The reaction mixture was stirred for 4 h at 25°C. Volatile components were evaporated *in vacuo*, the residue was dissolved in dichloromethane (5 mL) and washed with NaOH (aq., 2 M, 2 × 5mL) and NaCl (aq. sat., 1 × 5mL). Yield: 150 mg (0.34 mmol, 62%) of yellowish semisolid. $[\alpha]_D^{r.t.}$ = +18.8 (0.15, MeOH). EI-HRMS: *m/z* = 313.2635 $(M)^+$; $C_{21}H_{33}N_2^+$ requires: *m/z* = 313.2635 $(M)^+$; ν_{max} 3273, 2951, 2881, 2188, 2152, 1969, 1594, 1458, 1372, 1303, 1217, 1142, 1077, 1033, 1004, 917, 822, 764, 725, 641 cm^{-1}. ^{1}H-NMR (600 MHz, $CDCl_3$): δ 1.03 – 1.06 (*m*, 1H), 1.06 (s, 3H), 1.07 (s, 3H), 1.41 – 1.50 (*m*, 1H), 1.67 (*t*, *J* = 4.6, 1H), 1.77 (*td*, *J* = 10.7, 9.7, 6.5, 1H), 1.81 – 1.88 (*m*, 1H), 1.92 – 1.99 (*m*, 1H), 2.05 – 2.11 (*m*, 1H), 2.12 – 2.21 (*m*, 2H), 2.26 (*ddd*, *J* = 13.4, 9.4, 4.1, 1H), 2.36 – 2.75 (*m*, 3H), 3.68 (*dt*, *J* = 10.5, 3.1, 1H), 3.75 (*ddd*, *J* = 12.3, 8.5, 5.9, 1H), 3.81 – 3.87 (*m*, 1H), 3.83 (*d*, *J* = 14.1, 1H), 3.89 (*d*, *J* = 14.2, 1H), 4.07 (*ddd*, *J* = 12.2, 8.3, 6.3, 1H), 4.16 (*ddd*, *J* = 11.9, 8.2, 6.2, 1H), 5.06 (*d*, *J* = 12.6, 1H), 5.25 (*d*, *J* = 12.6, 1H), 7.42 – 7.50 (*m*, 3H), 7.66 (*d*, *J* = 6.6, 2H). ^{13}C-NMR (151 MHz, $CDCl_3$): δ 19.88, 21.16, 21.96, 22.08, 26.80, 29.23, 42.76, 44.69, 52.63, 53.69, 54.48, 60.85, 61.96, 63.75, 67.23, 128.66, 129.39, 130.71, 133.42.

Synthesis of phase-transfer bifunctional catalysts – *General procedure 4* (*GP4*)

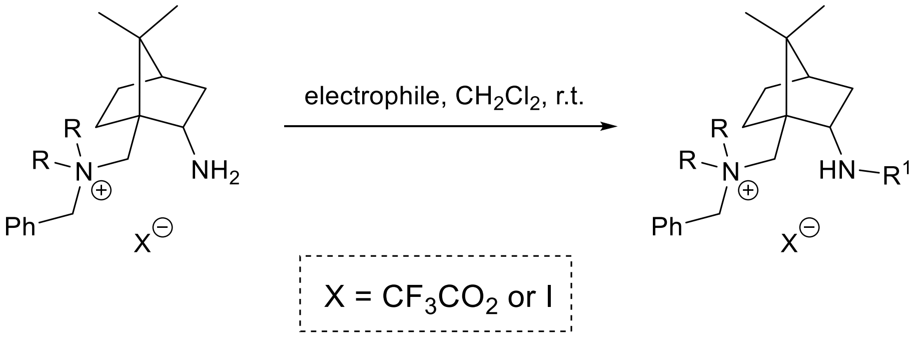

Amine **7** or **8** was dissolved in anhydrous CH_2Cl_2, the appropriate electrophile was added (1.2-1.4 equivalents), and the reaction mixture was stirred for 16 h at room temperature. The volatiles were evaporated *in vacuo*. The residue was purified by column chromatography (CC). The fractions containing product **I**–**X** were combined and the volatiles were evaporated *in vacuo*.

Trifluoroacetate anion exchange – *General procedure 5* (*GP5*)

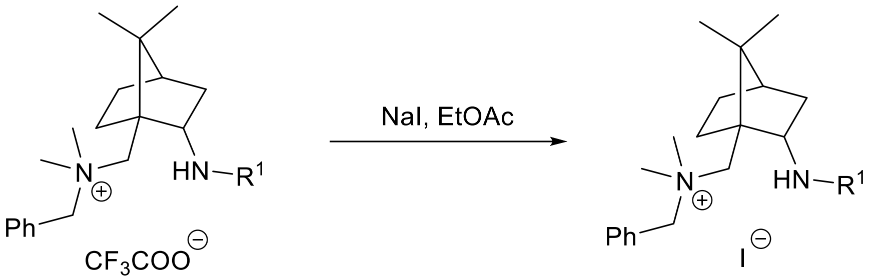

The column was packed with NaI (5 g) and conditioned with ethyl acetate. The trifluoroacetate phase transfer catalyst was dissolved in ethyl acetate and applied to the NaI column. The fractions containing the product were combined and the volatiles were evaporated *in vacuo*. Based on the ^{19}F NMR spectra (presence of a signal for fluorine from trifluoroacetate anion), the procedure was repeated as necessary.

***N*-Benzyl-1-((1*R*,2*R*,4*R*)-2-(3-(3,5-bis(trifluoromethyl)phenyl)thioureido)-7,7-dimethylbicyclo[2.2.1]heptan-1-yl)-*N*,*N*-dimethylmethanaminium 2,2,2-trifluoroacetate (I)**

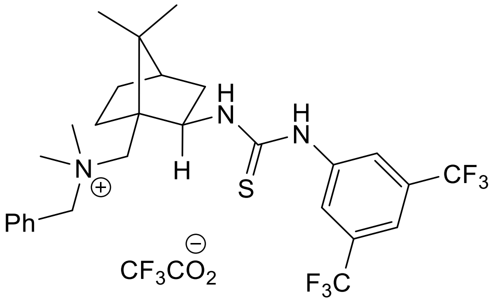

Following *GP4*. Prepared from (1*S*,2*R*,4*R*)-1-((benzyldimethylammonio)methyl)-7,7-dimethylbicyclo[2.2.1]heptan-2-aminium 2,2,2-trifluoroacetate (**8a**) (0.585 mmol, 300 mg) and 3,5-bis(trifluoromethyl)phenyl isothiocyanate (1.05 mmol, 192 µL), CH_2Cl_2 (4 mL), 25°C, 16 h. Isolation by evaporation followed by column chromatography (Silica gel 60, EtOAc/MeOH = 4 : 1). Yield: 316 mg (0.47 mmol, 80%) of yellowish solid, mp = 87.5–89.0°C. $[\alpha]_D^{r.t.}$ = +6.8 (0.13, MeOH). EI-HRMS: *m/z* = 558.2374 $(M)^+$; $C_{28}H_{34}F_6N_3S^+$ requires: *m/z* = 558.2372 $(M)^+$; ν_{max} 3260, 2962, 2885, 2091, 1679, 1622, 1523, 1472, 1382, 1333, 1274, 1218, 1171, 1125, 999, 970, 883, 848, 828, 780, 760, 727, 700, 680 cm^{-1}. ^{1}H-NMR (500 MHz, $CDCl_3$): δ 0.89 (*s*, 3H); 1.18 (*s*, 3H); 1.36 – 1.45 (*m*, 1H); 1.66 – 1.74 (*m*, 1H); 1.81 – 1.94 (*m*, 4H); 2.22 (*dd*, *J* = 8.6, 13.4, 1H); 3.00 (*s*, 3H); 3.08 (*s*, 3H); 3.29 (*d*, *J* = 14.5, 1H); 4.65 (*d*, *J* = 12.6, 1H); 4.74 – 4.80 (*m*, 1H); 4.84 – 4.93 (*m*, 2H); 7.40 – 7.54 (*m*, 6H); 8.36 (*s*, 2H); 8.79 (*d*, *J* = 7.9, 1H); 10.97 (*s*, 1H). ^{13}C NMR (126 MHz, $CDCl_3$): δ 20.76, 20.93, 27.84, 34.28, 41.11, 43.92, 50.38, 50.44, 51.25, 53.08, 58.99, 66.90, 71.59, 113.57, 116.74 – 117.00 (*m*), 117.08 (*q*, *J* = 294.0), 122.13 (*d*, *J* = 4.0), 123.41 (*q*, *J* = 272.6), 124.49, 126.58, 129.53, 131.34 (*q*, *J* = 33.2), 131.37, 133.09, 141.70, 162.07 (*q*, *J* = 34.3), 180.02.

N-Benzyl-1-((1R,2R,4R)-2-(3-(3,5-bis(trifluoromethyl)phenyl)thioureido)-7,7-dimethylbicyclo[2.2.1]heptan-1-yl)-N,N-dimethylmethanaminium iodide (II)

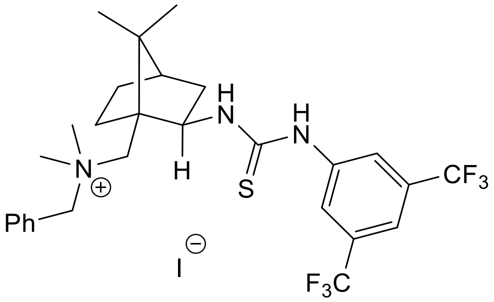

Following *GP5*. Prepared from *N*-benzyl-1-((1*R*,2*R*,4*R*)-2-(3-(3,5-bis(trifluoromethyl)phenyl)thioureido)-7,7-dimethylbicyclo[2.2.1]heptan-1-yl)-*N*,*N*-dimethylmethanaminium 2,2,2-trifluoroacetate (**I**) (0.21 mmol, 140 mg), dissolved in ethyl acetate (4 mL) and filtered through a pad of NaI. Volatile components were evaporated *in vacuo*. Yield: 140 mg (0.20 mmol, 96%) of colorless solid, mp = 61.4–62.9°C. $[\alpha]_D^{r.t.}$ = +5 (0.19, MeOH). EI-HRMS: *m/z* = 558.2365 $(M)^+$; $C_{28}H_{34}F_6N_3S^+$ requires: *m/z* = 558.2372 $(M)^+$; ν_{max} 3305, 2964, 1674, 1536, 1473, 1384, 1336, 1275, 1172, 1123, 1000, 971, 884, 846, 801, 780, 759, 725, 701, 679 cm^{-1}. ^1H-NMR (500 MHz, $CDCl_3$): δ 0.96 (*s*, 3H); 1.28 (*s*, 3H); 1.39 – 1.46 (*m*, 1H); 1.64 – 1.73 (*m*, 1H); 1.81 – 2.01 (*m*, 4H); 2.23 (*dd*, *J* = 8.8, 13.4, 1H); 3.08 (*s*, 3H); 3.14 (*s*, 3H); 3.51 (*d*, *J* = 14.3, 1H); 4.81 – 4.89 (*m*, 1H); 4.96 (*d*, *J* = 12.5, 1H); 5.07 (*d*, *J* = 13.9, 1H); 5.17 (*d*, *J* = 12.7, 1H); 7.38 – 7.45 (*m*, 2H); 7.46 – 7.51 (*m*, 1H); 7.54 (*s*, 1H); 7.55 – 7.60 (*m*, 2H); 8.08 (*d*, *J* = 8.1, 1H); 8.43 (*s*, 2H); 10.52 (*s*, 1H). ^{13}C-NMR (126 MHz, $CDCl_3$): δ 21.37, 21.94, 27.90, 34.23, 41.04, 43.83, 50.02, 50.70, 51.55, 53.44, 59.26, 66.70, 70.85, 117.35, 122.27, 123.36 (q, *J* = 272.7), 126.56, 129.49, 131.36, 131.36 (q, *J* = 33.3), 133.31, 141.23, 179.91.

1-Benzyl-1-(((1*S*,2*S*,4*R*)-2-(3-(3,5-bis(trifluoromethyl)phenyl)thioureido)-7,7-dimethylbicyclo[2.2.1]heptan-1-yl)methyl)pyrrolidin-1-ium iodide (III)

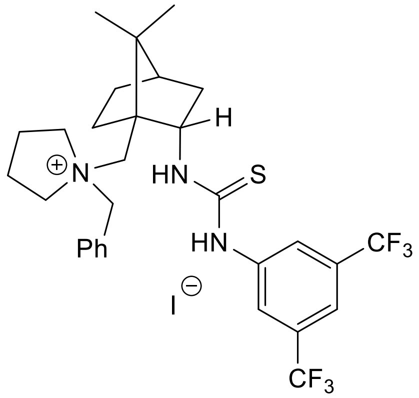

Following *GP4*. Prepared from 1-(((1*S*,2*S*,4*R*)-2-amino-7,7-dimethylbicyclo[2.2.1]heptan-1-yl)methyl)-1-benzylpyrrolidin-1-ium iodide (**7b**) (0.34 mmol, 150 mg) and 3,5-bis(trifluoromethyl)phenyl isothiocyanate (0.68 mmol, 124 µL), CH_2Cl_2 (3 mL), 25°C, 16 h. Isolation by column chromatography (Silica gel 60, EtOAc/MeOH = 10 : 1). Yield: 99 mg (0.14 mmol, 41%) of brownish semisolid. $[\alpha]_D^{r.t.}$ = +20 (0.047, MeOH). EI-HRMS: *m/z* = 584.2519 $(M)^+$; $C_{30}H_{36}F_6N_3S^+$ requires: *m/z* = 584.2529 $(M)^+$; ν_{max} 3194, 3126, 2968, 2149, 1625, 1589, 1542, 1492, 1472, 1381, 1324, 1271, 1249, 1222, 1166, 1136, 1108, 1094, 1061, 1025, 999, 967, 909, 885, 847, 756, 721, 701, 679, 612 cm^{-1}. ^1H-NMR (500 MHz, $CDCl_3$): δ 1.03 (*s*, 3H); 1.06 (*s*, 3H); 1.15 (*dd*, *J* = 13.4, 3.9, 1H); 1.51 – 1.93 (*m*, 5H); 1.98 – 2.06 (*m*, 1H); 2.16 – 2.31 (*m*, 2H), 2.61 – 2.70 (*m*, 1H); 2.99 – 3.07 (*m*, 1H); 3.41 – 3.51 (*m*, 2H), 3.67 – 3.83 (*m*, 3H); 3.83 – 3.93 (*m*, 1H); 4.63 (*d*, *J* = 12.9, 1H); 4.96 (*d*, *J* = 12.8, 1H); 5.34 – 5.45 (*m*, 1H); 7.39 – 7.45 (*m*, 2H); 7.47 – 7.52 (*m*, 1H); 7.54 – 7.58 (*m*, 2H); 7.60 (*s*, 1H); 8.34 – 8.43 (*m*, 3H); 10.67 (*s*, 1H). ^{13}C-NMR (126 MHz, $CDCl_3$): δ 19.98, 20.65, 21.49, 21.93, 28.65, 29.57, 38.39, 43.68, 51.83, 54.54, 57.00, 60.89, 61.91, 64.34, 67.34, 117.85 – 118.26 (*m*), 123.08 – 123.29 (*m*), 123.38 (*q*, *J* = 209.2), 127.38, 129.67, 131.28, 131.57 (*q*, *J* = 33.4), 133.39, 140.92, 182.20.

N-Benzyl-1-((1R,2S,4R)-2-(3-(3,5-bis(trifluoromethyl)phenyl)thioureido)-7,7-dimethylbicyclo[2.2.1]heptan-1-yl)-N,N-dimethylmethanaminium 2,2,2-trifluoroacetate (IV)

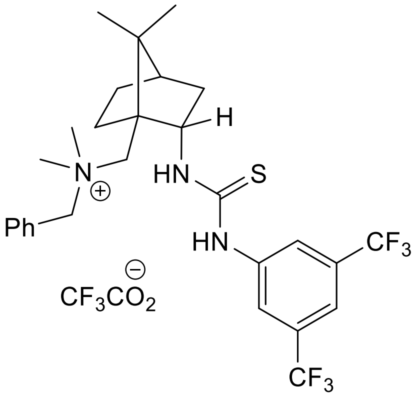

Following *GP4*. Prepared from (1*S*,2*S*,4*R*)-1-((benzyldimethylammonio)methyl)-7,7-dimethylbicyclo[2.2.1]heptan-2-aminium 2,2,2-trifluoroacetate (**7a**) (0.39 mmol, 200 mg) and 3,5-bis(trifluoromethyl)phenyl isothiocyanate (0.70 mmol, 128 µL), CH_2Cl_2 (4 mL), 25°C, 16 h. Isolation by column chromatography (Silica gel 60, EtOAc/MeOH = 4 : 1). Yield: 250 mg (0.37 mmol, 95%) of colorless solid, mp = 153–155°C. $[\alpha]_D^{r.t.}$ = +2.1 (0.11, MeOH). EI-HRMS: *m/z* = 558.2363 $(M)^+$; $C_{28}H_{34}F_6N_3S^+$ requires: *m/z* = 558.2372 $(M)^+$; ν_{max} 3275, 3247, 3047, 2961, 2890, 1682, 1542, 1473, 1385, 1278, 1177, 1132, 966, 887, 848, 801, 719, 702, 680 cm^{-1}. ^{1}H-NMR (500 MHz, $CDCl_3$): δ 0.97 (s, 3H), 1.05 (*s*, 3H), 1.10 (*dd*, *J* = 13.4, 3.7, 1H), 1.51 (*ddd*, *J* = 13.4, 9.2, 4.7, 1H), 1.73 (*t*, *J* = 4.6, 1H), 1.78 – 1.89 (*m*, 1H), 1.95 – 2.12 (*m*, 1H), 2.59 – 2.67 (*m*, 1H), 2.68 – 2.76 (*m*, 1H), 3.03 (*s*, 3H), 3.04 (*s*, 3H), 3.47 (*d*, *J* = 13.7, 1H), 3.72 (*d*, *J* = 13.7, 1H), 4.55 (*d*, *J* = 12.6, 1H), 4.71 (*d*, *J* = 12.6, 1H), 5.22 (*tt*, *J* = 10.2, 3.0, 1H), 7.34 – 7.40 (*m*, 4H), 7.43 – 7.48 (*m*, 1H), 7.53 (*s*, 1H), 8.29 (*s*, 2H), 8.91 (*d*, *J* = 9.8, 1H), 11.09 (*s*, 1H). ^{13}C-NMR (126 MHz, $CDCl_3$): δ 19.81, 20.25, 28.05, 28.57, 38.68, 43.75, 50.78, 51.03, 51.76, 54.43, 56.70, 69.58, 73.03, 117.02 (*q*, *J* = 294.4), 117.24 – 117.53 (*m*), 122.72 (*d*, *J* = 3.4), 123.39 (*q*, *J* = 272.7), 126.96, 129.47, 131.24, 131.48 (*q*, *J* = 33.4), 133.03, 141.44, 161.62 (*q*, *J* = 34.1), 181.96.

N-Benzyl-1-((1R,2S,4R)-2-(3-(3,5-bis(trifluoromethyl)phenyl)thioureido)-7,7-dimethylbicyclo[2.2.1]heptan-1-yl)-N,N-dimethylmethanaminium iodide (V)

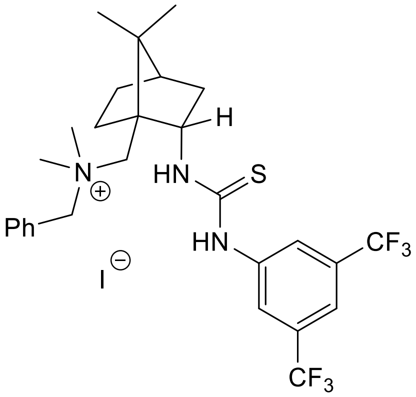

Following *GP5*. Prepared from N^1-((*S*)-(6-methoxyquinolin-4-yl)((1*S*,2*S*,4*S*,5*R*)-5-vinylquinuclidin-2-yl)methyl)-4-(trifluoromethyl)benzene-1,2-diamine (**IV**) (0.17 mmol, 116 mg), dissolved in ethyl acetate (3 mL) and filtered through a pad of NaI. Volatile components were evaporated *in vacuo*. Yield: 109 mg (0.16 mmol, 92%) of white solid, mp = decomposition above 350°C. $[\alpha]_D^{r.t.}$ = +69.2 (0.013, MeOH). EI-HRMS: *m/z* = 558.2368 $(M)^+$; $C_{28}H_{34}F_6N_3S^+$ requires: *m/z* = 558.2372 $(M)^+$; ν_{max} 3247, 2960, 2928, 2857, 2175, 2163, 2135, 2034, 1996, 1954, 1722, 1595, 1534, 1473, 1385, 1277, 1177, 1135, 965, 887, 730, 701, 680 cm^{-1}. ^{1}H-NMR (500 MHz, DMSO-d_6): δ 0.98 (*s*, 3H), 1.02 (*dd*, *J* = 13.0, 3.7, 1H), 1.05 (*s*, 3H), 1.43 – 1.52 (*m*, 1H), 1.72 (*t*, *J* = 4.4, 1H), 1.90 – 2.06 (*m*, 2H), 2.16 – 2.24 (*m*, 1H), 2.45 – 2.49 (*m*, 1H), 2.95 (*s*, 3H), 2.99 (*s*, 3H), 3.60 (*d*, *J* = 14.1, 1H), 3.71 (*d*, *J* = 14.0, 1H), 4.52 – 4.63 (*m*, 2H), 5.04 – 5.12 (*m*, 1H), 7.43 – 7.59 (*m*, 5H), 7.81 (*s*, 1H), 8.28 (*s*, 2H), 8.38 (*d*, *J* = 9.8, 1H), 10.32 (*s*, 1H). ^{13}C-NMR (126 MHz, DMSO-d_6): δ 19.25, 20.04, 26.97, 28.11, 38.10, 42.97, 49.08, 50.46, 51.23, 53.88, 56.13, 67.81, 70.46, 116.76 – 117.00 (*m*), 122.27 – 122.44 (*m*), 123.17 (*q*, *J* = 272.8), 128.03, 128.86, 130.17 (*q*, *J* = 32.8), 130.38, 133.06, 141.46, 181.00.

N-Benzyl-1-((1R,2S,4R)-7,7-dimethyl-2-(3-phenylthioureido)bicyclo[2.2.1]heptan-1-yl)-N,N-dimethylmethanaminium 2,2,2-trifluoroacetate (VI)

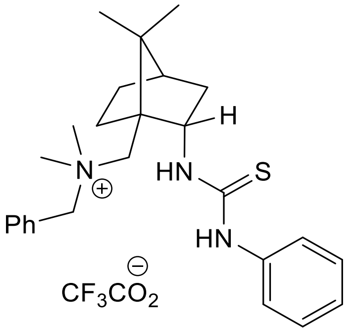

Following *GP4*. Prepared from (1*S*,2*S*,4*R*)-1-((benzyldimethylammonio)methyl)-7,7-dimethylbicyclo[2.2.1]heptan-2-aminium 2,2,2-trifluoroacetate (**7a**) (0.39 mmol, 200 mg) and phenyl isothiocyanate (0.70 mmol, 84 µL), CH_2Cl_2 (4 mL), 25°C, 16 h. Isolation by evaporation followed by column chromatography (Silica gel 60, EtOAc/MeOH = 4 : 1). Yield: 119 mg (0.22 mmol, 56 %) of colorless solid, mp = 180–183°C. $[\alpha]_D^{r.t.}$ = +6.7 (0.06, MeOH). EI-HRMS: *m/z* = 422.2618 $(M)^+$; $C_{26}H_{36}N_3S^+$ requires: *m/z* = 422.2624 $(M)^+$; ν_{max} 3244, 2959, 2884, 1683, 1540, 1507, 1489, 1473, 1457, 1362, 1317, 1202, 1148, 1056, 1033, 851, 801, 727 cm^{-1}. ^{1}H-NMR (500 MHz, $CDCl_3$): δ 0.96 (*s*, 3H); 1.04 (*s*, 3H); 1.08 (*dd*, *J* = 13.3, 3.7, 1H); 1.45 – 1.53 (*m*, 1H); 1.67 – 1.79 (*m*, 2H); 1.96 – 2.06 (*m*, 1H); 2.57 – 2.65 (*m*, 1H); 2.67 – 2.76 (*m*, 1H); 2.98 (*s*, 3H); 3.03 (*s*, 3H); 3.41 (*d*, *J* = 13.6, 1H); 3.73 (*d*, *J* = 13.8, 1H); 4.54 (*d*, *J* = 12.5, 1H); 4.79 (*d*, *J* = 12.4, 1H), 5.24 – 5.31 (*m*, 1H), 7.04 – 7.10 (*m*, 1H); 7.22 – 7.29 (*m*, 2H); 7.33 – 7.47 (*m*, 5H); 7.60 – 7.68 (*m*, 2H); 8.64 (*d*, *J* = 9.9, 1H); 10.45 (*s*, 1H). ^{13}C-NMR (126 MHz, $CDCl_3$): δ 19.95, 20.50, 28.53, 28.65, 38.67, 43.85, 51.11, 51.22, 51.72, 54.34, 56.50, 69.08, 72.83, 123.82, 124.84, 127.30, 128.46, 129.38, 130.98, 133.35, 139.65, 182.21 (two signals missing).

N-Benzyl-1-((1R,2S,4R)-7,7-dimethyl-2-(3-phenylureido)bicyclo[2.2.1]heptan-1-yl)-N,N-dimethylmethanaminium 2,2,2-trifluoroacetate (VII)

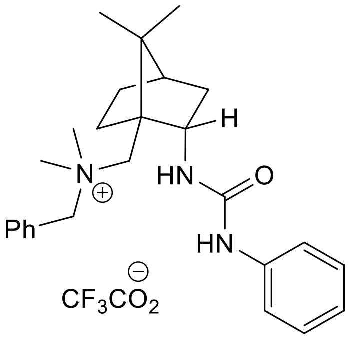

Following *GP4*. Prepared from (1*S*,2*S*,4*R*)-1-((benzyldimethylammonio)methyl)-7,7-dimethylbicyclo[2.2.1]heptan-2-aminium 2,2,2-trifluoroacetate (**7a**) (0.39 mmol, 200 mg) and phenyl isocyanate (0.69 mmol, 76 µL), CH_2Cl_2 (4 mL), 25°C, 16 h. Isolation by evaporation followed by column chromatography (Silica gel 60, EtOAc/MeOH = 5 : 1). Yield: 57 mg (0.11 mmol, 28%) of colorless solid, mp = 120.0–123.8°C. $[\alpha]_D^{r.t.}$ = +5 (0.08, MeOH). EI-HRMS: *m/z* = 406.2850 $(M)^+$; $C_{26}H_{36}N_3O^+$ requires: *m/z* = 406.2853 $(M)^+$; ν_{max} 3261, 2960, 2886, 2150, 1683, 1598, 1550, 1489, 1457, 1313, 1202, 1139, 846, 801, 727, 702 cm^{-1}. ^{1}H-NMR (500 MHz, $CDCl_3$): δ 0.95 (*s*, 3H), 1.00 (*s*, 3H), 1.12 (*dd*, *J* = 13.3, 3.6, 1H), 1.44 – 1.53 (*m*, 1H), 1.55 – 1.68 (*m*, 2H), 2.03 – 2.11 (*m*, 1H), 2.47 – 2.55 (*m*, 1H), 2.60 – 2.67 (*m*, 1H), 3.04 (*s*, 3H), 3.08 (*s*, 3H), 3.36 (*d*, *J* = 13.7, 1H), 3.88 (*d*, *J* = 13.7, 1H), 4.52 – 4.59 (*m*, 1H), 4.73 (*d*, *J* = 12.4, 1H), 4.97 (*d*, *J* = 12.3, 1H), 6.91 – 6.98 (*m*, 1H), 7.18 – 7.24 (*m*, 2H), 7.25 – 7.30 (*m*, 3H), 7.35 – 7.40 (*m*, 1H), 7.42 – 7.47 (*m*, 2H), 7.51 – 7.57 (*m*, 2H), 9.31 (*s*, 1H). ^{13}C-NMR (126 MHz, $CDCl_3$): δ 19.71, 20.51, 28.46, 28.68, 39.90, 44.00, 50.46, 51.29, 51.76, 52.02, 54.06, 68.41, 72.90, 117.34 (*d*, *J* = 295.3), 118.79, 122.19, 127.49, 128.82, 129.24, 130.75, 133.32, 139.93, 156.51, 161.52 (*q*, *J* = 33.5).

N-Benzyl-1-((1R,2S,4R)-7,7-dimethyl-2-(3-phenylureido)bicyclo[2.2.1]heptan-1-yl)-N,N-dimethylmethanaminium iodide (VIII)

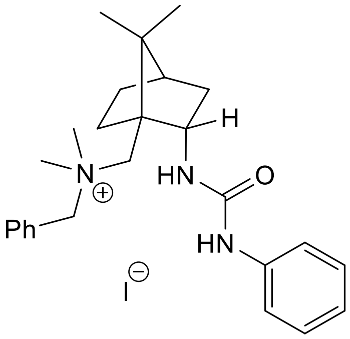

Following *GP5*. Prepared from *N*-benzyl-1-((1*R*,2*S*,4*R*)-7,7-dimethyl-2-(3-phenylureido)bicyclo[2.2.1]heptan-1-yl)-*N*,*N*-dimethylmethanaminium 2,2,2-trifluoroacetate (**VII**) (0.26 mmol, 139 mg), dissolved in ethyl acetate (5 mL) and filtered through a pad of NaI. All volatile components were evaporated *in vacuo*. Yield: 111 mg (0.21 mmol, 80%) of colorless solid, mp = 153–155°C. $[\alpha]_D^{r.t.}$ = +78 (0.073, MeOH). EI-HRMS: *m/z* = 406.2850 $(M)^+$; $C_{26}H_{36}N_3O^+$ requires: *m/z* = 406.2853 $(M)^+$; ν_{max} 3277, 2967, 2881, 1678, 1597, 1543, 1487, 1442, 1377, 1311, 1217, 1158, 1128, 1030, 949, 852, 816, 753, 729, 694 cm^{-1}. ^{1}H-NMR (500 MHz, $CDCl_3$): δ 1.01 (*s*, 6H), 1.14 (*dd*, *J* = 13.3; 3.6, 1H), 1.46 – 1.60 (*m*, 2H), 1.62 (*t*, *J* = 4.5, 1H), 2.23 – 2.31 (*m*, 1H), 2.48 – 2.56 (*m*, 1H), 2.67 – 2.76 (*m*, 1H), 3.08 (*s*, 3H), 3.11 (*s*, 3H), 3.34 (*d*, *J* = 13.7, 1H), 4.07 (*d*, *J* = 13.6, 1H), 4.54 – 4.65 (*m*, 1H), 4.80 (*d*, *J* = 12.3, 1H), 5.05 (*d*, *J* = 12.3, 1H), 6.75 (*d*, *J* = 10.8, 1H), 6.94 – 7.00 (*m*, 1H), 7.19 – 7.25 (*m*, 2H), 7.31 (*t*, *J* = 7.6, 2H), 7.38 – 7.43 (*m*, 1H), 7.48 – 7.53 (*m*, 2H), 7.56 – 7.62 (*m*, 2H), 8.93 (*s*, 1H). ^{13}C-NMR (126 MHz, $CDCl_3$): δ 19.88, 20.81, 28.41, 29.13, 39.96, 44.02, 50.51, 51.57, 51.95, 52.34, 54.45, 68.01, 72.56, 118.82, 122.49, 127.33, 128.83, 129.29, 130.86, 133.38, 139.60, 156.34.

N-Benzyl-1-((1R,2S,4R)-2-((2-((3,5-bis(trifluoromethyl)phenyl)amino)-3,4-dioxocyclobut-1-en-1-yl)amino)-7,7-dimethylbicyclo[2.2.1]heptan-1-yl)-N,N-dimethylmethanaminium 2,2,2-trifluoroacetate (IX)

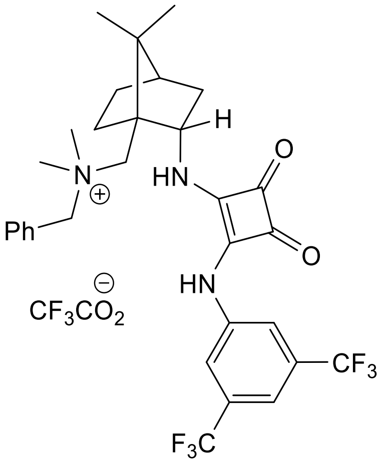

Following *GP4*. Prepared from (1S,2S,4R)-1-((benzyldimethylammonio)methyl)-7,7-dimethylbicyclo[2.2.1]heptan-2-aminium 2,2,2-trifluoroacetate (**7a**) (0.19 mmol, 100 mg) and 3-((3,5-bis(trifluoromethyl)phenyl)amino)-4-ethoxycyclobut-3-ene-1,2-dione (0.30 mmol, 106,4 mg), CH_2Cl_2 (2 mL), 25°C, 16 h. Isolation by evaporation followed by column chromatography (Silica gel 60, EtOAc/MeOH = 4 : 1). Yield: 106 mg (0.15 mmol, 75%) of colorless solid, mp = 148.9–150.1°C. $[\alpha]_D^{r.t.}$ = +65 (0.006, MeOH). EI-HRMS: m/z = 594.2545 $(M)^+$; $C_{31}H_{34}F_6N_3O_2$ requires: m/z = 594.2550 $(M)^+$; ν_{max} 3420, 3153, 3034, 2967, 2888, 1791, 1686, 1603, 1551, 1475, 1427, 1377, 1276, 1176, 1127, 948, 931, 880, 848, 831, 730, 701, 684, 666 cm^{-1}. ^{1}H-NMR (500 MHz, $CDCl_3$): δ 1.02 (*s*, 3H), 1.17 (*s*, 3H), 1.35 (*dd*, J = 13.2, 3.6, 1H), 1.60 – 1.66 (*m*, 1H), 1.77 (*t*, J = 4.5, 1H), 1.89 (br *t*, J = 13.3, 1H), 1.93 – 2.05 (*m*, 1H), 2.52 – 2.62 (*m*, 1H), 3.01 (*s*, 1H), 3.14 (*s*, 3H), 3.16 (*s*, 3H), 3.42 (*d*, J = 13.8, 1H), 4.33 (*d*, J = 13.9, 1H), 4.63 (*d*, J = 12.5, 1H), 4.76 (*d*, J = 12.5, 1H), 5.27 (*t*, J = 9.9, 1H), 7.41 (*t*, J = 7.4, 2H), 7.45 – 7.50 (*m*, 2H), 7.55 – 7.66 (*m*, 2H), 8.21 (*s*, 2H), 9.13 (*d*, J = 9.2, 1H), 11.34 (*s*, 1H). ^{13}C-NMR (126 MHz, $CDCl_3$): δ 19.80, 20.66, 26.61, 28.93, 41.29, 44.05, 50.80, 51.47, 52.79, 55.04, 58.56, 70.65, 73.17, 116.24, 119.01, 123.33 (*q*, J = 272.9), 126.85, 129.59, 131.38, 132.65 (*q*, J = 33.4), 133.40, 140.74, 165.81, 169.02, 181.07, 185.00. (two carbon missing).

1-Benzyl-1-(((1*S*,2*S*,4*R*)-7,7-dimethyl-2-(3-phenylthioureido)bicyclo[2.2.1]heptan-1-yl)methyl)pyrrolidin-1-ium iodide (X)

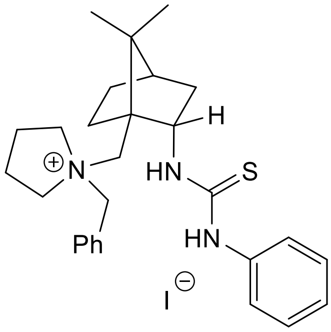

Following *GP4*. Prepared from 1-(((1*S*,2*S*,4*R*)-2-amino-7,7-dimethylbicyclo[2.2.1]heptan-1-yl)methyl)-1-benzylpyrrolidin-1-ium iodide (**7b**) (0.25 mmol, 109 mg) and phenyl isothiocyanate (0.38 mmol, 45 µL), CH_2Cl_2 (2 mL), 25°C, 16 h. Isolation by column chromatography (Silica gel 60, EtOAc/MeOH = 10 : 1). Yield: 72 mg (0.13 mmol, 50%) of colorless solid, mp = 178–180 °C. $[\alpha]_D^{r.t.}$ = +7.4 (0.14, MeOH). EI-HRMS: *m/z* = 448.2776 $(M)^+$; $C_{28}H_{38}N_3S$ requires: *m/z* = 448.2781 $(M)^+$; ν_{max} 3209, 3030, 2953, 1685, 1597, 1528, 1495, 1450, 1360, 1308, 1243, 1144, 1089, 1027, 1002, 915, 758, 716, 698, 607 cm^{-1}. ^{1}H-NMR (500 MHz, $CDCl_3$): δ 0.99 (*s*, 3H), 1.02 (*s*, 3H), 1.12 (*dd*, *J* = 13.4, 3.9, 1H), 1.42 – 1.52 (*m*, 2H), 1.65 (*t*, *J* = 4.3, 1H), 1.71 – 1.89 (*m*, 2H), 1.96 – 2.07 (*m*, 1H), 2.15 – 2.23 (*m*, 2H), 2.53 – 2.64 (br *t*, *J* = 11.8, 1H), 2.97 (br *s*, 1H), 3.40 (*d*, *J* = 13.9, 1H), 3.42 – 3.48 (*m*, 1H), 3.70 (*dt*, *J* = 12.3, 7.4, 1H), 3.81 (*d*, *J* = 13.8, 1H), 3.83 – 3.99 (*m*, 2H), 4.64 (*d*, *J* = 12.7, 1H), 5.12 (*d*, *J* = 12.7, 1H), 5.40 (br *t*, *J* = 10.6, 1H), 7.14 (*t*, *J* = 7.3, 1H), 7.31 (*t*, *J* = 7.6, 2H), 7.41 (*t*, *J* = 7.4, 2H), 7.46 (*t*, *J* = 7.3, 1H), 7.60 (*d*, *J* = 6.8, 2H), 7.76 (*d*, *J* = 7.2, 2H), 8.10 (*d*, *J* = 10.4, 1H), 10.10 (*s*, 1H). ^{13}C-NMR (126 MHz, $CDCl_3$): δ 19.98, 20.70, 21.34, 22.00, 28.48, 29.80, 38.39, 43.70, 51.66, 54.42, 56.54, 60.24, 62.02, 63.82, 67.18, 123.99, 125.16, 127.84, 128.55, 129.49, 130.93, 133.61, 139.32, 182.27.

2. HPLC data

Evaluation of organocatalysts I–IX in the fluorination of *β*-keto ester 9

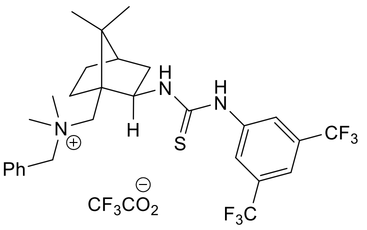

I

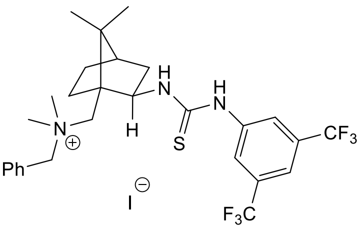

II

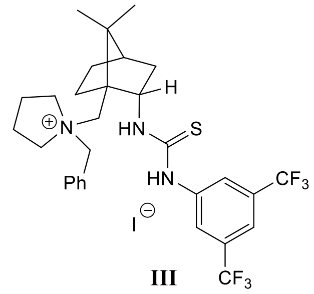

III

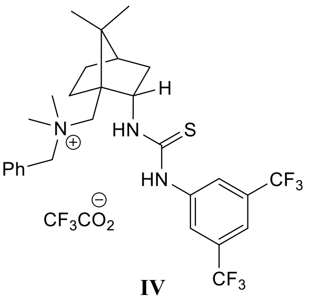

IV

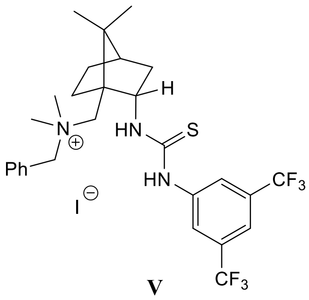

V

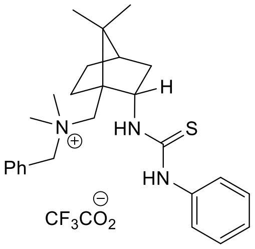

VI

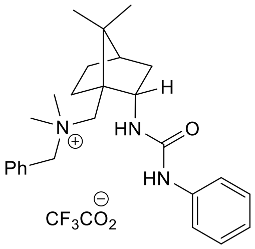

VII

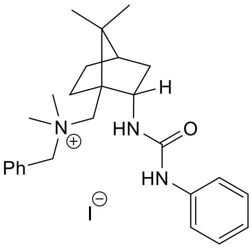

VIII

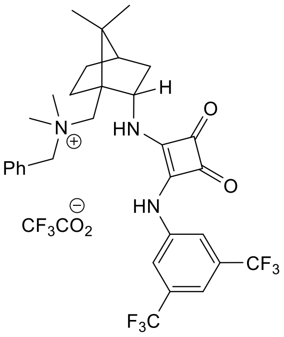

IX

Figure S1. Applied organocatalysts **I**–**IX**.

Table S1. Evaluation of organocatalysts **I–IX** in the fluorination of *β*-keto ester **9**.[2,3] [a]

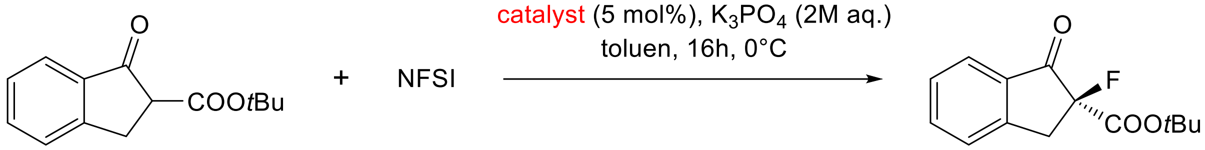

**Entry**	**Catalyst**	**Yield [b] (%)**	***ee* [c] (%)**
1	**I**	63	3 (*S*)
2	**II**	96	9 (*S*)
3	**III**	38	18 (*S*)
4	**IV**	96	1 (*R*)
5	**V**	94	3 (*R*)
6	**VI**	58	5 (*S*)
7	**VII**	71	5 (*S*)
8	**VIII**	87	29 (*S*)
9	**IX**	90	18 (*S*)

[a] Aqueous K_3PO_4 (2 M, 2 equivalents, 0.1 mL) was added to a mixture of *β*-keto ester **9** (0.1 mmol, 24.4 mg, ω = 95%) and organocatalyst **I–IX** (2 mol%) in toluene (2 mL) under argon atmosphere. Mixture was cooled to –10°C and NFSI (1.1 equivalents, 34.7 mg) was added in two portions over 2 h. The reaction mixture was stirred for another 12 h at –10°C. After completion, the reaction was quenched by addition of NH_4Cl (aq. sat, 4 mL) and extracted with CH_2Cl_2 (10 mL). The organic phase was dried over anhydrous Na_2SO_4, filtered, and the volatiles were evaporated *in vacuo*. The residue was purified by column chromatography (Silica gel 60, EtOAc/*n*-Heptane = 1 : 15). [b] Reaction yield determined after isolation with column chromatography (Silica gel 60, EtOAc/Heptane = 1 : 15). [c] *ee* determined by HPLC

(Chiralpak AD-H, *n*-Hexane/*i*-PrOH = 200:1, flow rate 0.75 mL/min, λ = 250 nm, 10°C) after the isolation by column chromatography.

Table S1, Entry 1

	**Catalyst**		**Yield (%)**	***ee* (%)**
1	**I**	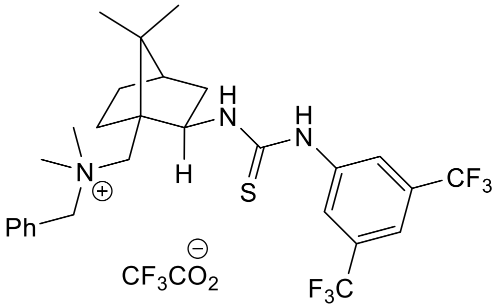	63	3 (*S*)

HPLC: Chiralpak AD-H, *n*-Hexane/*i*PrOH = 200:1, flow rate 0.75 mL/min, λ = 250 nm, 10°C

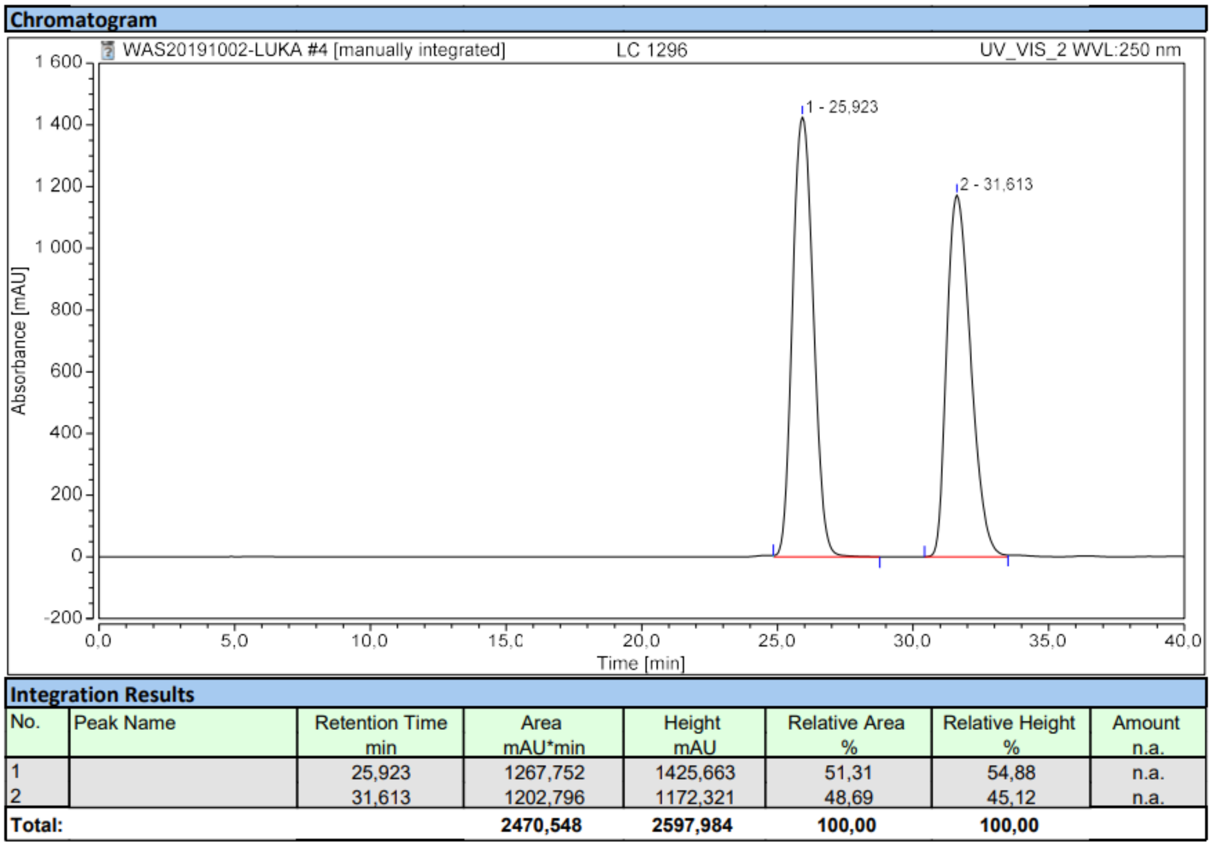

Integration Results

No.	Peak Name	Retention Time min	Area mAU*min	Height mAU	Relative Area %	Relative Height %	Amount n.a.
1		25,923	1267,752	1425,663	51,31	54,88	n.a.
2		31,613	1202,796	1172,321	48,69	45,12	n.a.
**Total:**			**2470,548**	**2597,984**	**100,00**	**100,00**	

Table S1, Entry 2

	Catalyst		Yield (%)	*ee* (%)
2	**II**	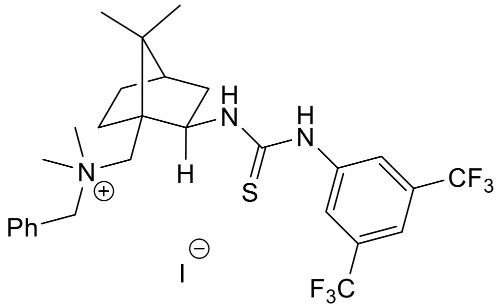	96	9 (*S*)

HPLC: Chiralpak AD-H, *n*-Hexane/*i*PrOH = 200:1, flow rate 0.75 mL/min, λ = 250 nm, 10°C

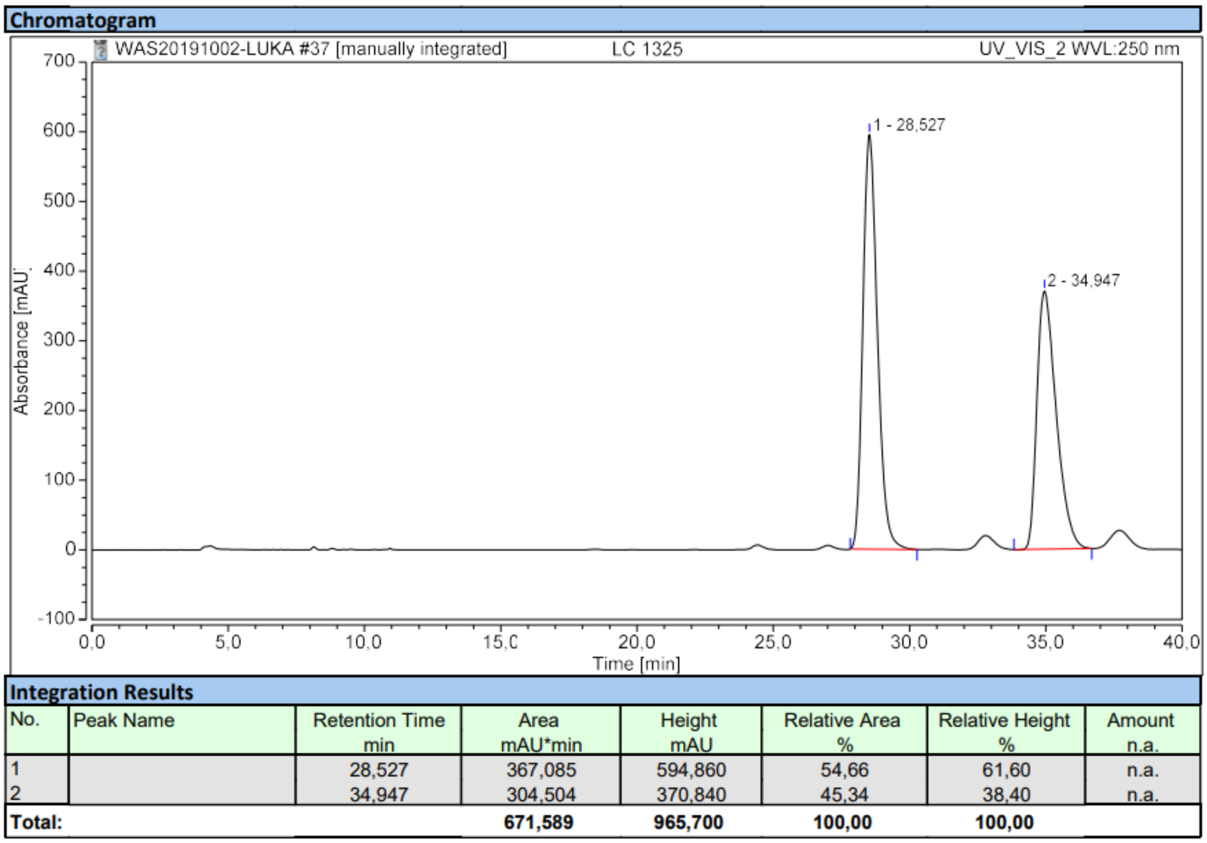

Integration Results

No.	Peak Name	Retention Time min	Area mAU*min	Height mAU	Relative Area %	Relative Height %	Amount n.a.
1		28,527	367,085	594,860	54,66	61,60	n.a.
2		34,947	304,504	370,840	45,34	38,40	n.a.
**Total:**			**671,589**	**965,700**	**100,00**	**100,00**	

Table S1, Entry 3

	Catalyst		Yield (%)	*ee* (%)
3	**III**	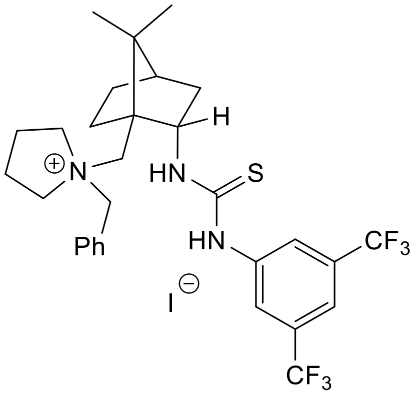	38	18 (*S*)

HPLC: Chiralpak AD-H, *n*-Hexane/*i*PrOH = 200:1, flow rate 0.75 mL/min, λ = 250 nm, 10°C

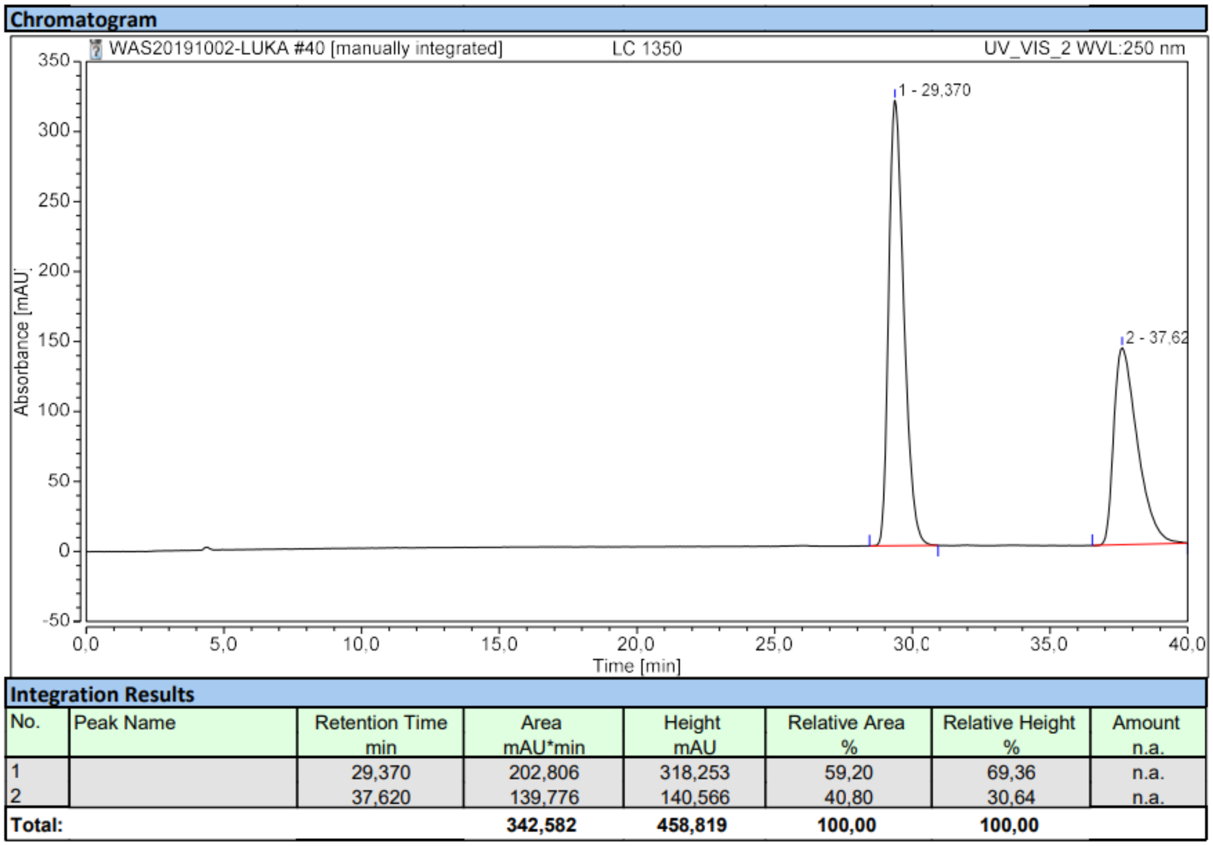

Integration Results

No.	Peak Name	Retention Time min	Area mAU*min	Height mAU	Relative Area %	Relative Height %	Amount n.a.
1		29,370	202,806	318,253	59,20	69,36	n.a.
2		37,620	139,776	140,566	40,80	30,64	n.a.
**Total:**			**342,582**	**458,819**	**100,00**	**100,00**	

Table S1, Entry 4

	Catalyst		Yield (%)	*ee* (%)
4	**IV**	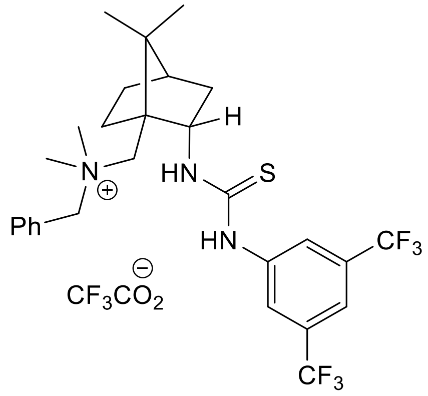	96	1 (*R*)

HPLC: Chiralpak AD-H, *n*-Hexane/*i*PrOH = 200:1, flow rate 0.75 mL/min, λ = 250 nm, 10°C

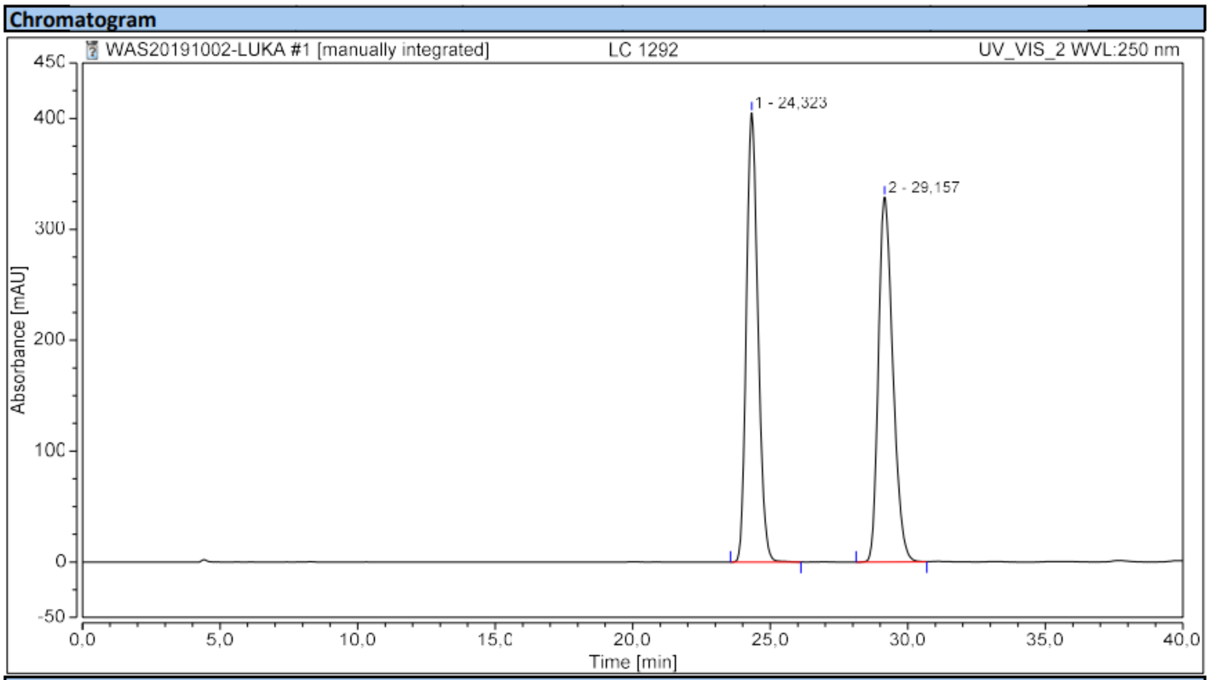

Integration Results

No.	Peak Name	Retention Time min	Area mAU*min	Height mAU	Relative Area %	Relative Height %	Amount n.a.
1		24,323	197,204	404,864	49,57	55,18	n.a.
2		29,157	200,629	328,851	50,43	44,82	n.a.
**Total:**			**397,832**	**733,715**	**100,00**	**100,00**	

Table S1, Entry 5

	Catalyst		Yield (%)	*ee* (%)
5	**V**	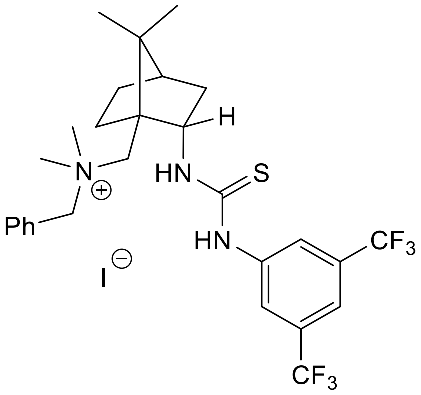	94	3 (*R*)

HPLC: Chiralpak AD-H, *n*-Hexane/*i*PrOH = 200:1, flow rate 0.75 mL/min, λ = 250 nm, 10℃

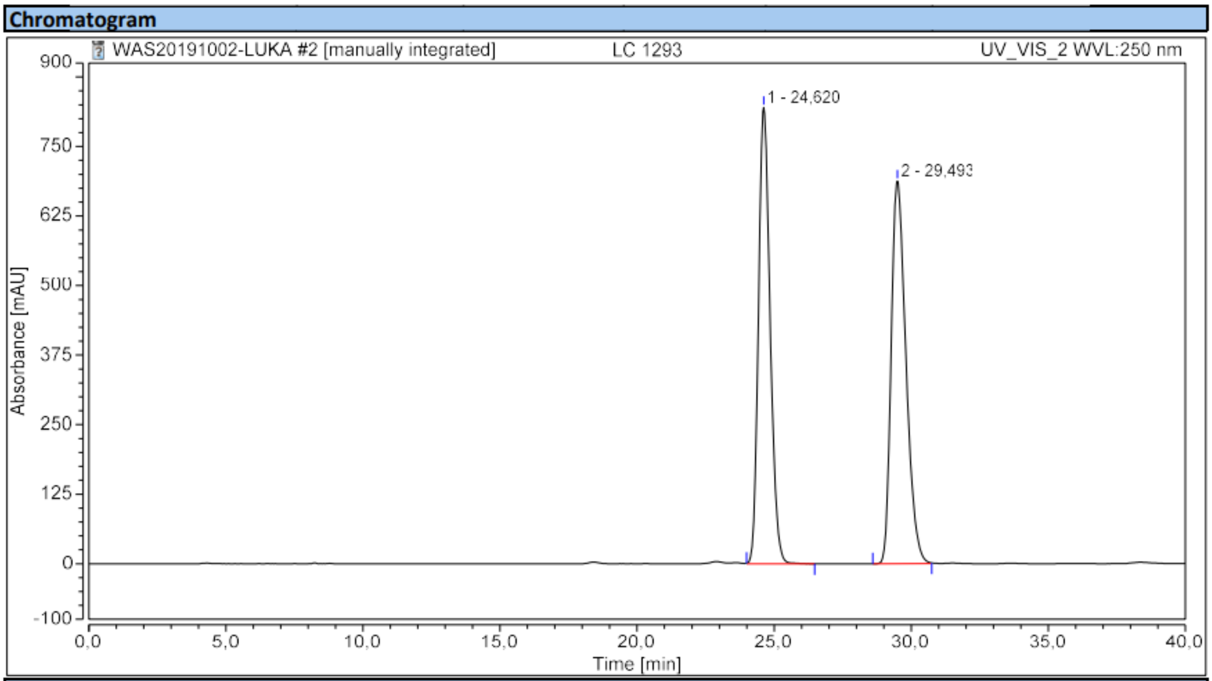

Integration Results

No.	Peak Name	Retention Time min	Area mAU*min	Height mAU	Relative Area %	Relative Height %	Amount n.a.
1		24,620	403,853	820,449	48,44	54,40	n.a.
2		29,493	429,856	687,839	51,56	45,60	n.a.
**Total:**			**833,709**	**1508,288**	**100,00**	**100,00**	

Table S1, Entry 6

	Catalyst		Yield (%)	*ee* (%)
6	**VI**	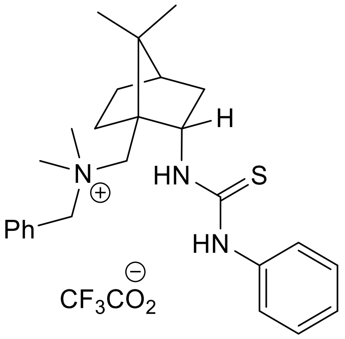	58	5 (*S*)

HPLC: Chiralpak AD-H, *n*-Hexane/*i*PrOH = 200:1, flow rate 0.75 mL/min, λ = 250 nm, 10°C

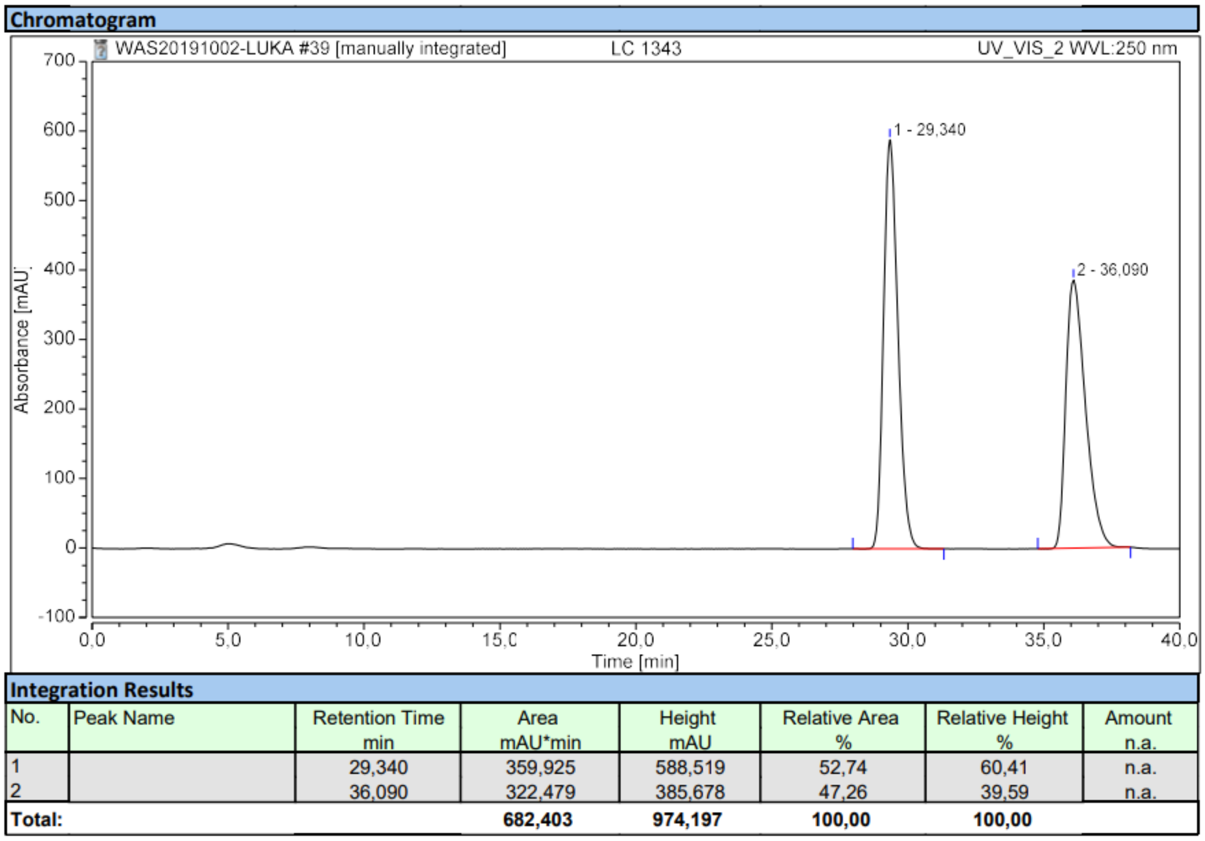

Integration Results

No.	Peak Name	Retention Time min	Area mAU*min	Height mAU	Relative Area %	Relative Height %	Amount n.a.
1		29,340	359,925	588,519	52,74	60,41	n.a.
2		36,090	322,479	385,678	47,26	39,59	n.a.
**Total:**			**682,403**	**974,197**	**100,00**	**100,00**	

Table S1, Entry 7

	Catalyst		Yield (%)	*ee* (%)
7	**VII**	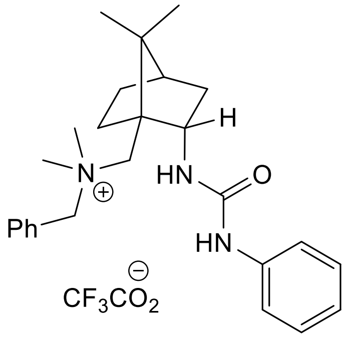	71	5 (*S*)

HPLC: Chiralpak AD-H, *n*-Hexane/*i*PrOH = 200:1, flow rate 0.75 mL/min, λ = 250 nm, 10°C

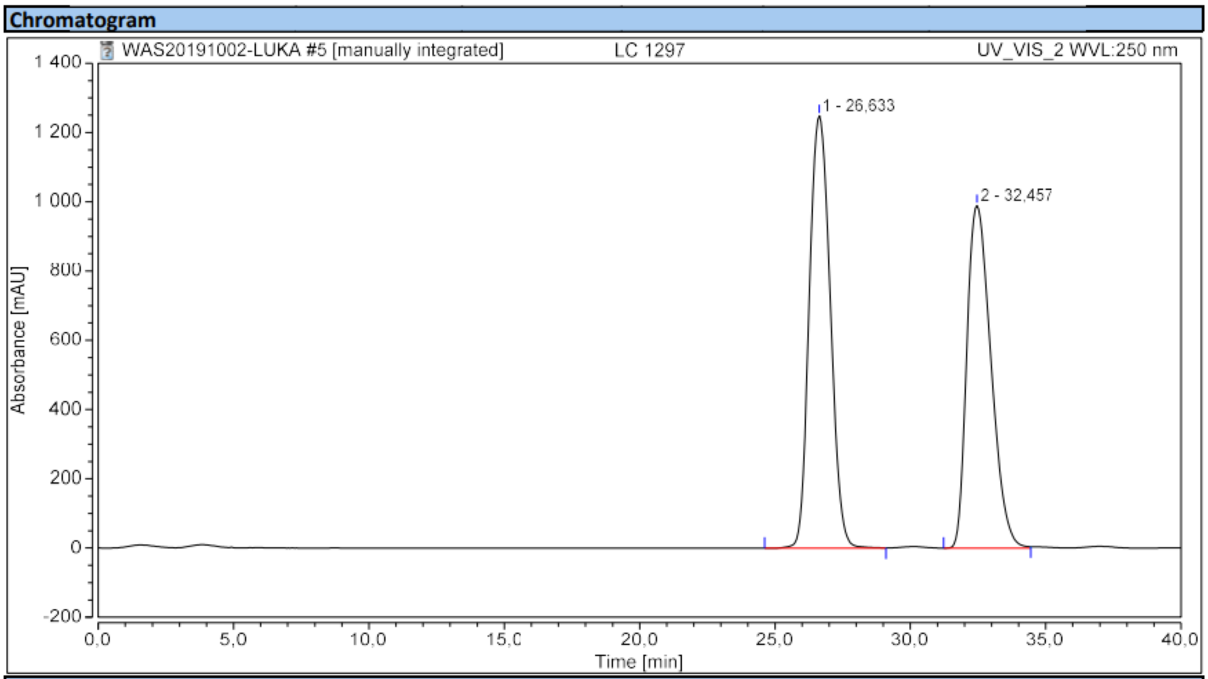

Integration Results

No.	Peak Name	Retention Time min	Area mAU*min	Height mAU	Relative Area %	Relative Height %	Amount n.a.
1		26,633	1123,141	1249,087	52,45	55,79	n.a.
2		32,457	1018,331	989,625	47,55	44,21	n.a.
**Total:**			**2141,472**	**2238,712**	**100,00**	**100,00**	

Table S1, Entry 8

	Catalyst		Yield (%)	*ee* (%)
8	**VIII**	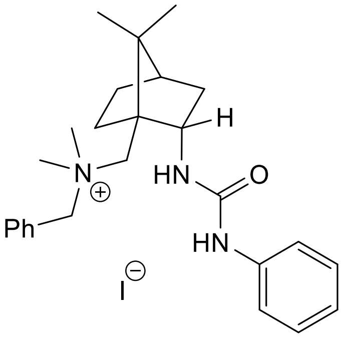	87	29 (*S*)

HPLC: Chiralpak AD-H, *n*-Hexane/*i*PrOH = 200:1, flow rate 0.75 mL/min, λ = 250 nm, 10°C

Chromatogram

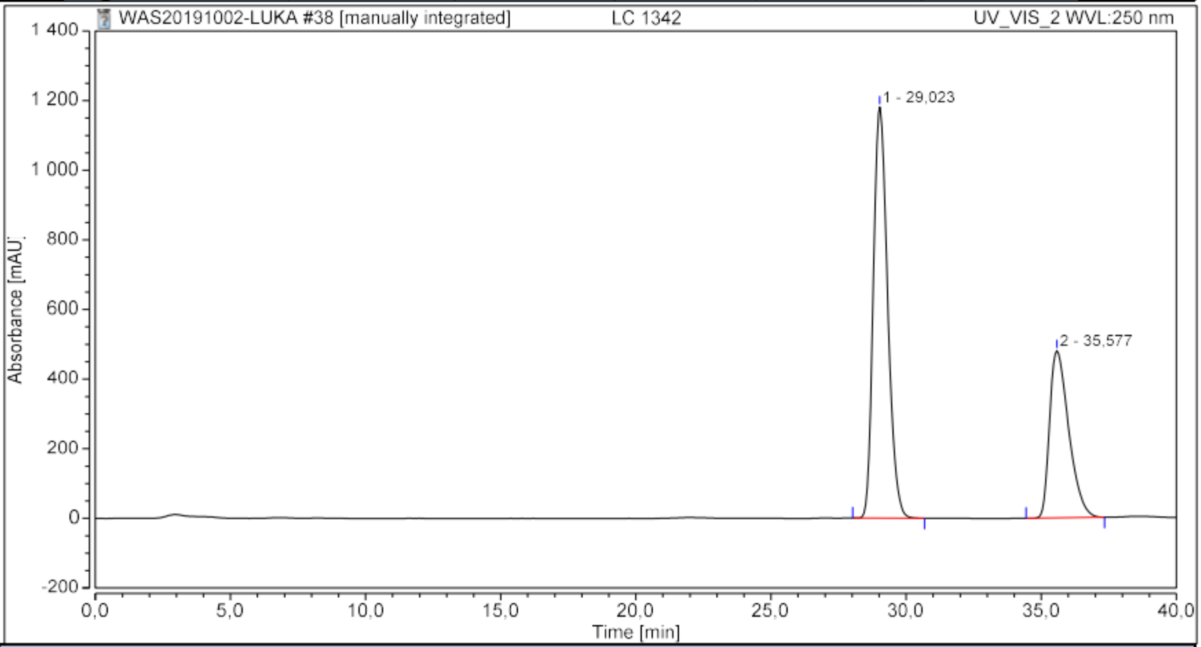

Integration Results

No.	Peak Name	Retention Time<br>min	Area<br>mAU*min	Height<br>mAU	Relative Area<br>%	Relative Height<br>%	Amount<br>n.a.
1		29,023	722,164	1181,046	64,70	71,11	n.a.
2		35,577	394,064	479,905	35,30	28,89	n.a.
**Total:**			**1116,227**	**1660,951**	**100,00**	**100,00**	

Table S1, Entry 9

	Catalyst		Yield (%)	*ee* (%)
9	**IX**	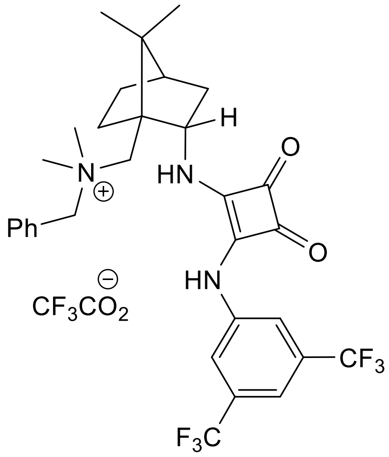	90	18 (*S*)

HPLC: Chiralpak AD-H, *n*-Hexane/*i*PrOH = 200:1, flow rate 0.75 mL/min, λ = 250 nm, 10°C

Chromatogram

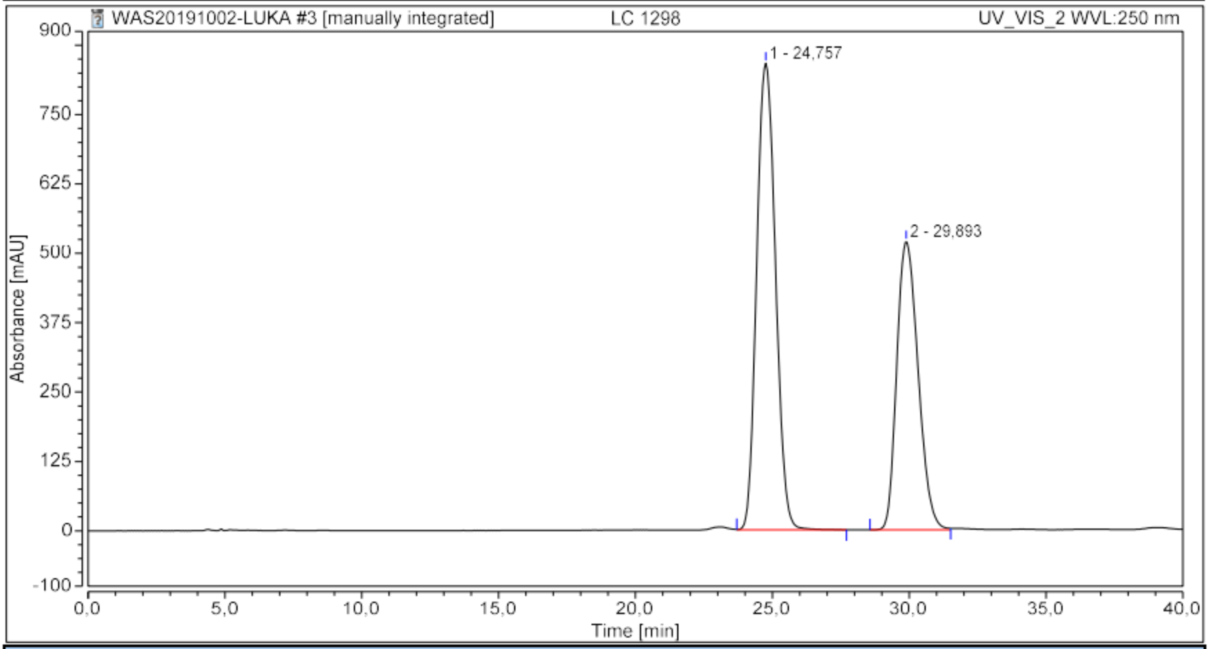

Integration Results

No.	Peak Name	Retention Time min	Area mAU*min	Height mAU	Relative Area %	Relative Height %	Amount n.a.
1		24,757	670,227	840,939	58,82	61,81	n.a.
2		29,893	469,212	519,535	41,18	38,19	n.a.
**Total:**			**1139,440**	**1360,474**	**100,00**	**100,00**	

Table S2. Further evaluation of organocatalysts **III**, **VIII**, and **IX** in the fluorination of β-keto ester **9**.[2,3] [a]

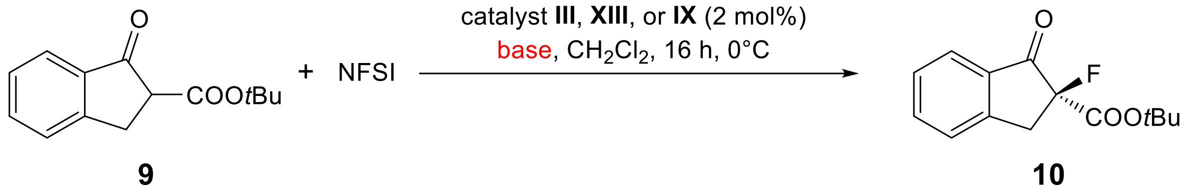

Entry	Catalyst	Base	Yield [b] (%)	*ee* [c] (%)
1	**III**	$K_3PO_{4(aq)}$ (1 eq.)	64	0
2	**III**	$K_3PO_{4(aq)}$ (2 eq.)	48	2 (*S*)
3	**III**	$K_3PO_{4(aq)}$ (1 eq.)	87	0
4	**VIII**	$K_3PO_{4(aq)}$ (1 eq.)	77	28 (S)
5	**VIII**	$K_3PO_{4(s)}$ (1 eq.)	96	23 (*S*)
6	**IX**	$K_3PO_{4(aq)}$ (1 eq.)	80	26 (*S*)
7	**IX**	$K_3PO_{4(aq)}$ (2 eq.)	98	19 (*S*)
8	**IX**	$K_2HPO_{4(aq)}$ (2 eq.)	96	9 (*S*)
9	**IX**	$Cs_2CO_{3(aq)}$ (2 eq.)	49	19 (*S*)
10	**IX**	$K_2CO_{3(aq)}$ (2 eq.)	51	14 (*S*)

[a] Appropriate base (2 M) was added to a mixture of β-keto ester **9** (0.1 mmol, 24.4 mg, ω = 95%) and organocatalyst **III**, **VIII**, or **IX** (2 mol%) in CH_2Cl_2 (2 mL) under argon atmosphere. Mixture was cooled to –10°C and NFSI (1.1 equivalents, 34.7 mg) was added in two portions over 2 h. The reaction mixture was stirred for another 12 h at –10°C. After completion, the reaction was quenched by addition of NH_4Cl (aq. sat, 4 mL) and extracted with CH_2Cl_2 (10

mL). The organic phase was dried over anhydrous Na_2SO_4, filtered, and the volatiles were evaporated *in vacuo*. The residue was purified by column chromatography (Silica gel 60, EtOAc/*n*-Heptane = 1 : 15). [b] Reaction yield determined after isolation with column chromatography (Silica gel 60, EtOAc/*n*-Heptane = 1 : 15). [c] *ee* determined by HPLC (Chiralpak AD-H, *n*-Hexane/*i*-PrOH = 200:1, flow rate 0.75 mL/min, λ = 250 nm, 10°C) after the isolation by column chromatography. [d] 2 equivalents of K_3PO_4.

Table S2, Entry 1

	Catalyst		Base	Yield (%)	*ee* (%)
1	**III**	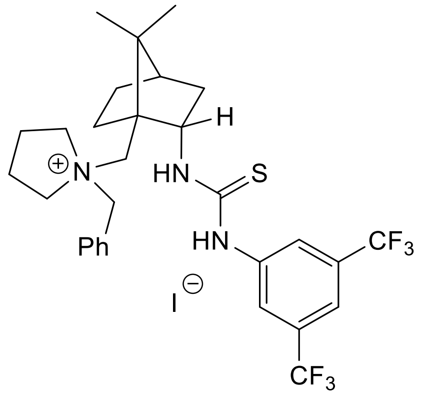	$K_3PO_{4(aq)}$ (1 eq.)	64	0

HPLC: Chiralpak AD-H, *n*-Hexane/*i*PrOH = 200:1, flow rate 0.75 mL/min, λ = 250 nm, 10°C

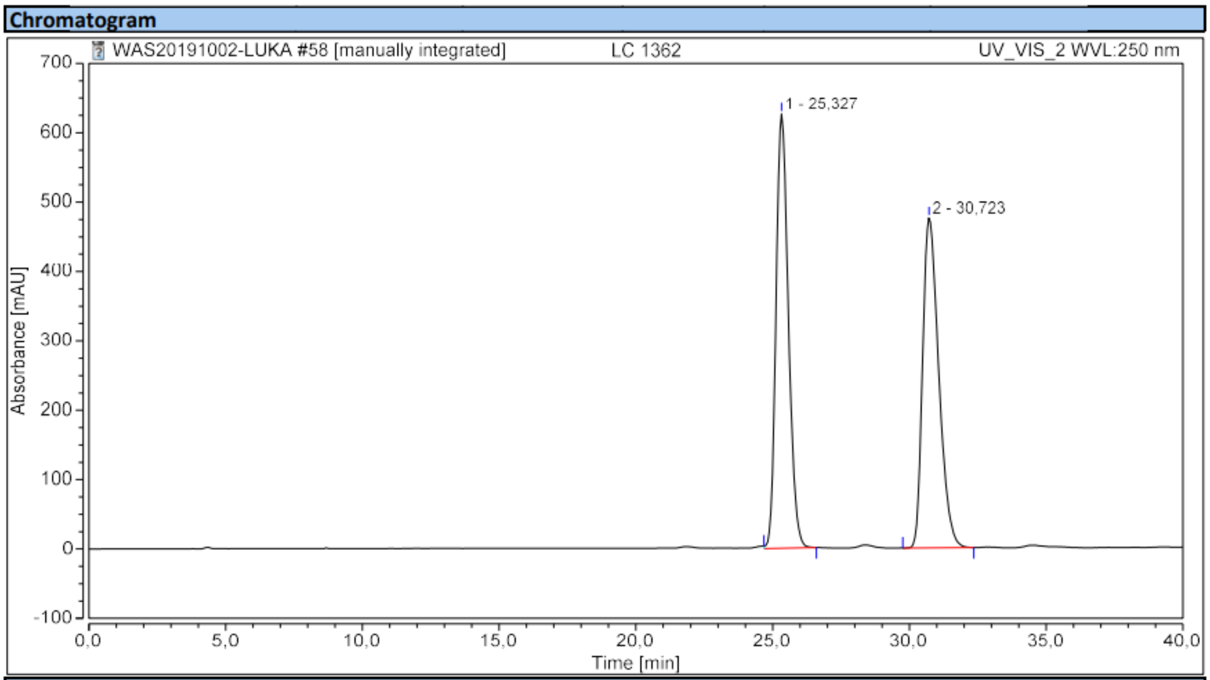

Integration Results

No.	Peak Name	Retention Time min	Area mAU*min	Height mAU	Relative Area %	Relative Height %	Amount n.a.
1		25,327	327,116	626,175	50,21	56,82	n.a.
2		30,723	324,316	475,833	49,79	43,18	n.a.
**Total:**			**651,432**	**1102,008**	**100,00**	**100,00**	

Table S2, Entry 2

	Catalyst		Base	Yield (%)	*ee* (%)
2	**III**	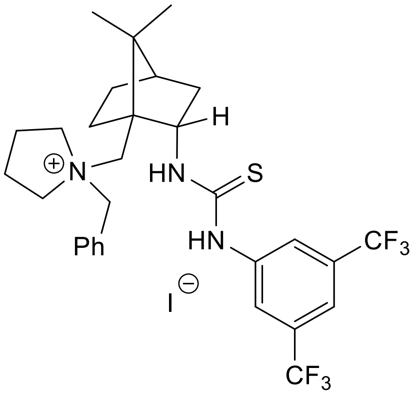	$K_3PO_{4(aq)}$ (2 eq.)	48	2 (*S*)

HPLC: Chiralpak AD-H, *n*-Hexane/*i*PrOH = 200:1, flow rate 0.75 mL/min, λ = 250 nm, 10°C

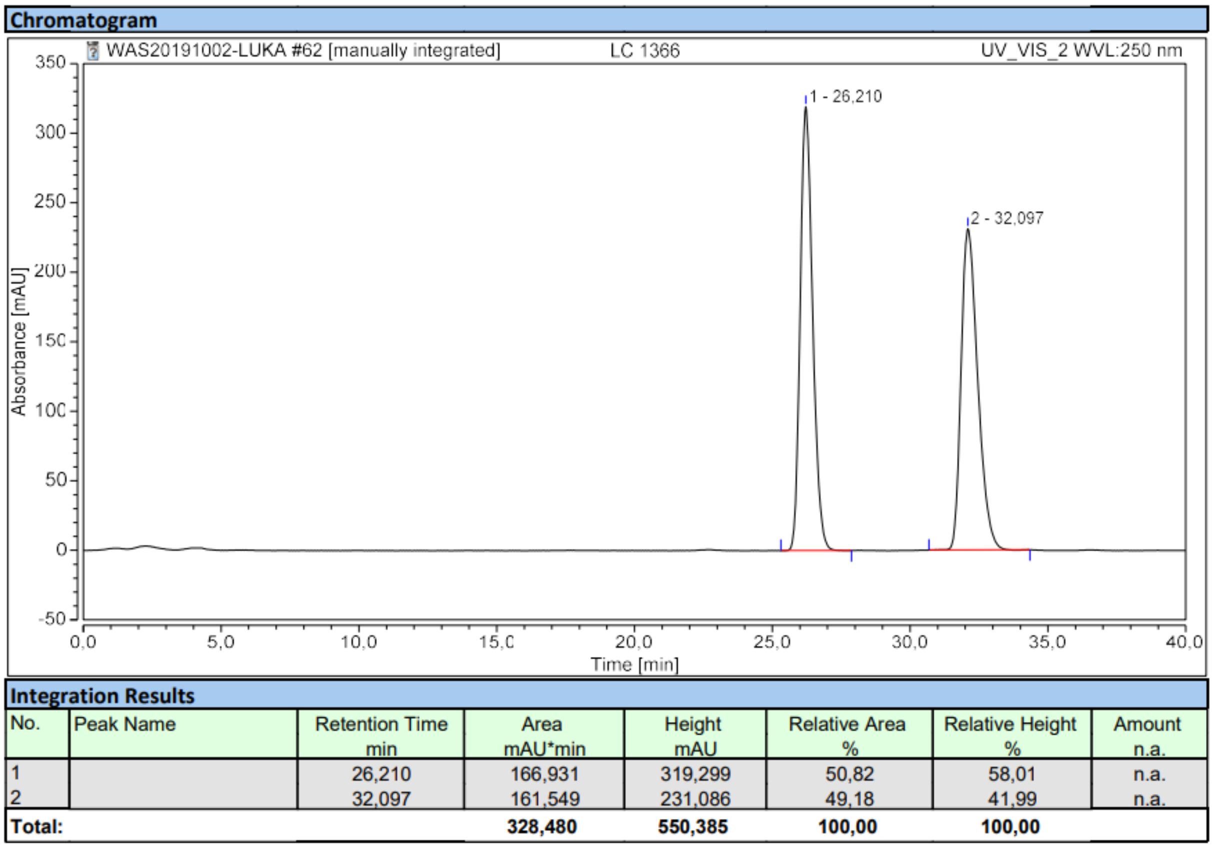

Integration Results

No.	Peak Name	Retention Time min	Area mAU*min	Height mAU	Relative Area %	Relative Height %	Amount n.a.
1		26,210	166,931	319,299	50,82	58,01	n.a.
2		32,097	161,549	231,086	49,18	41,99	n.a.
**Total:**			**328,480**	**550,385**	**100,00**	**100,00**	

Table S2, Entry 3

	Catalyst		Base	Yield (%)	*ee* (%)
3	**III**	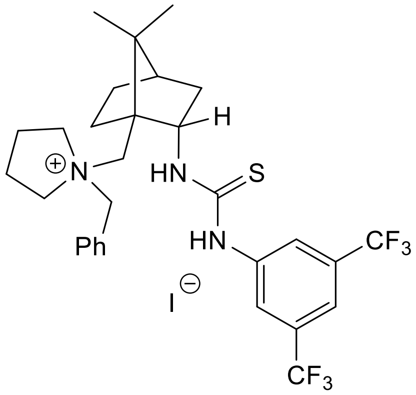	$K_3PO_{4(aq)}$ (1 eq.)	87	0

HPLC: Chiralpak AD-H, *n*-Hexane/*i*PrOH = 200:1, flow rate 0.75 mL/min, λ = 250 nm, 10°C

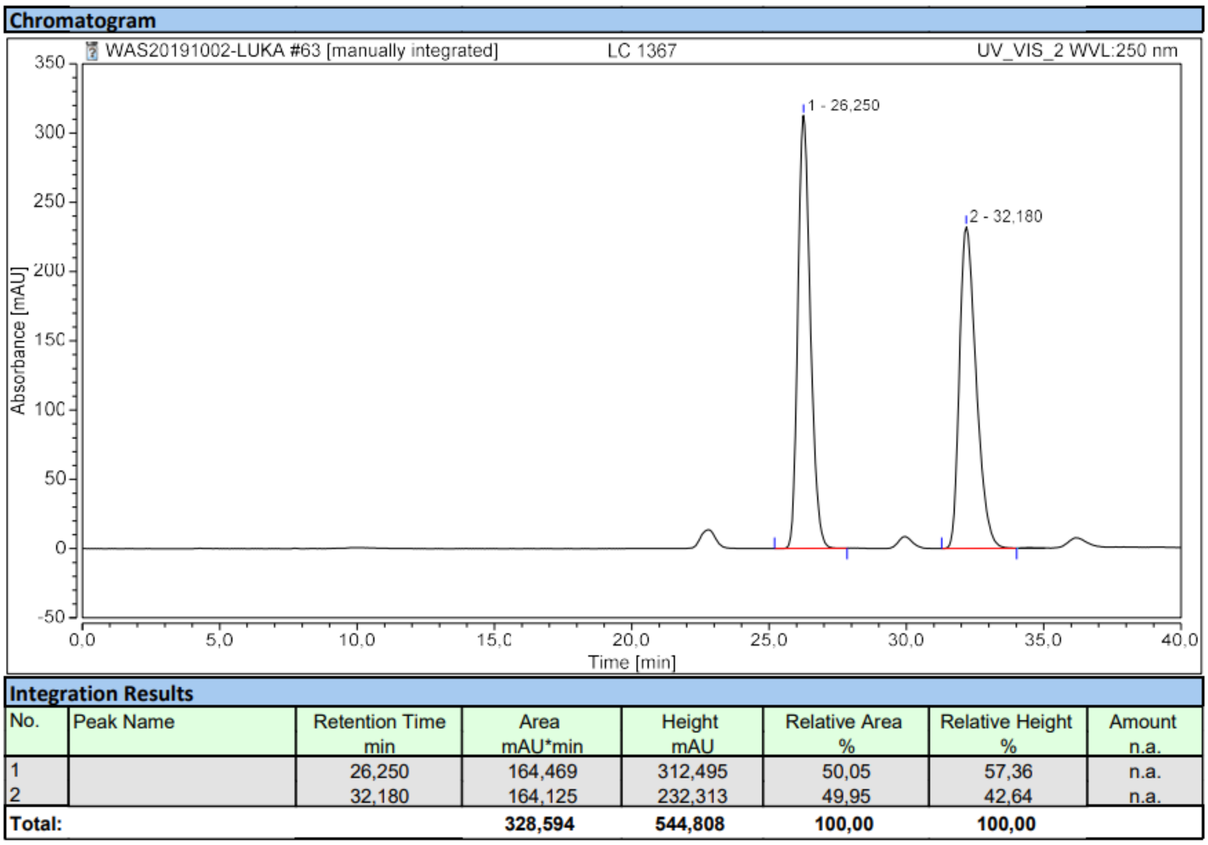

Integration Results

No.	Peak Name	Retention Time min	Area mAU*min	Height mAU	Relative Area %	Relative Height %	Amount n.a.
1		26,250	164,469	312,495	50,05	57,36	n.a.
2		32,180	164,125	232,313	49,95	42,64	n.a.
**Total:**			**328,594**	**544,808**	**100,00**	**100,00**	

Table S2, Entry 4

	Catalyst		Base	Yield (%)	*ee* (%)
4	**VIII**	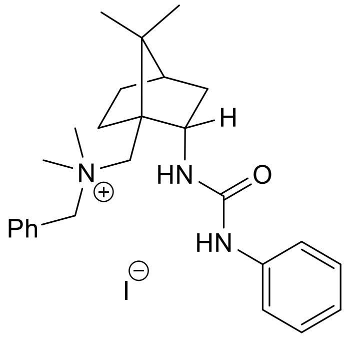	$K_3PO_{4(aq)}$ (1 eq.)	77	28 (*S*)

HPLC: Chiralpak AD-H, *n*-Hexane/*i*PrOH = 200:1, flow rate 0.75 mL/min, λ = 250 nm, 10°C

Chromatogram

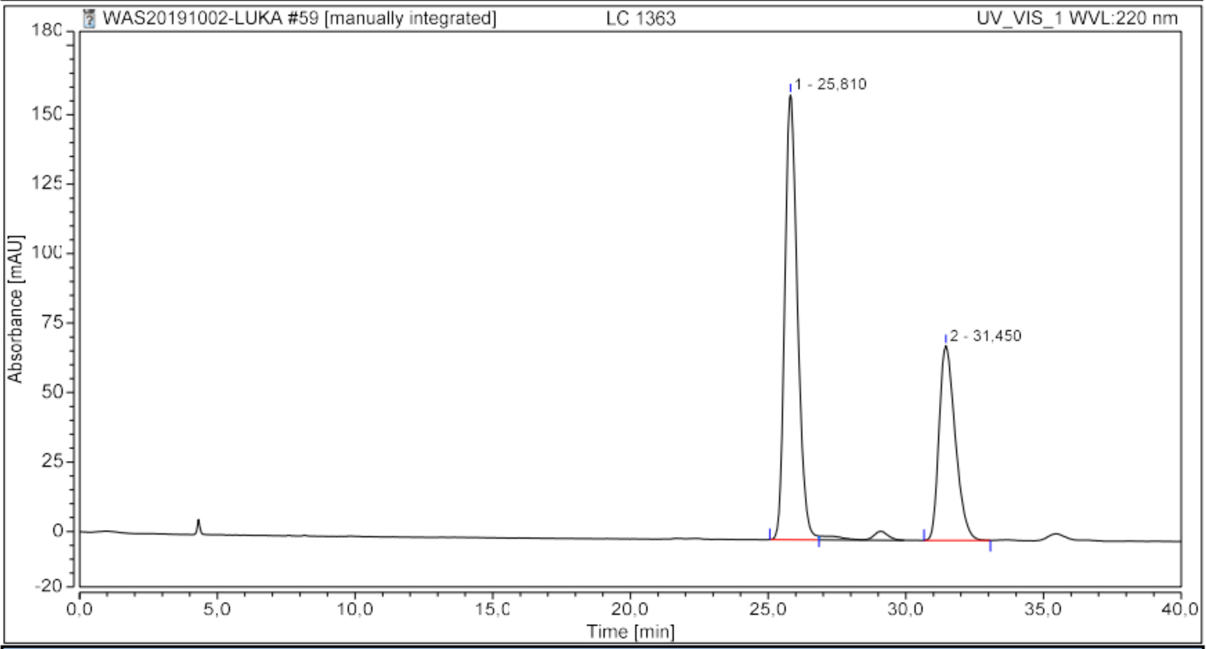

Integration Results

No.	Peak Name	Retention Time min	Area mAU*min	Height mAU	Relative Area %	Relative Height %	Amount n.a.
1		25,810	84,948	160,167	63,92	69,56	n.a.
2		31,450	47,956	70,092	36,08	30,44	n.a.
**Total:**			**132,905**	**230,259**	**100,00**	**100,00**	

Table S2, Entry 5

	Catalyst		Base	Yield (%)	*ee* (%)
5	**VIII**	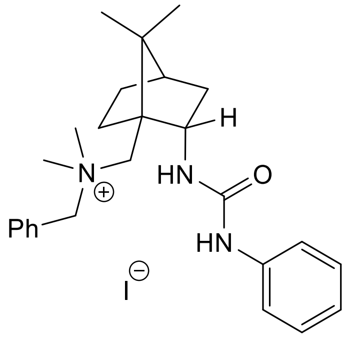	$K_3PO_{4(s)}$ (1 eq.)	96	23 (*S*)

HPLC: Chiralpak AD-H, *n*-Hexane/*i*PrOH = 200:1, flow rate 0.75 mL/min, λ = 250 nm, 10°C

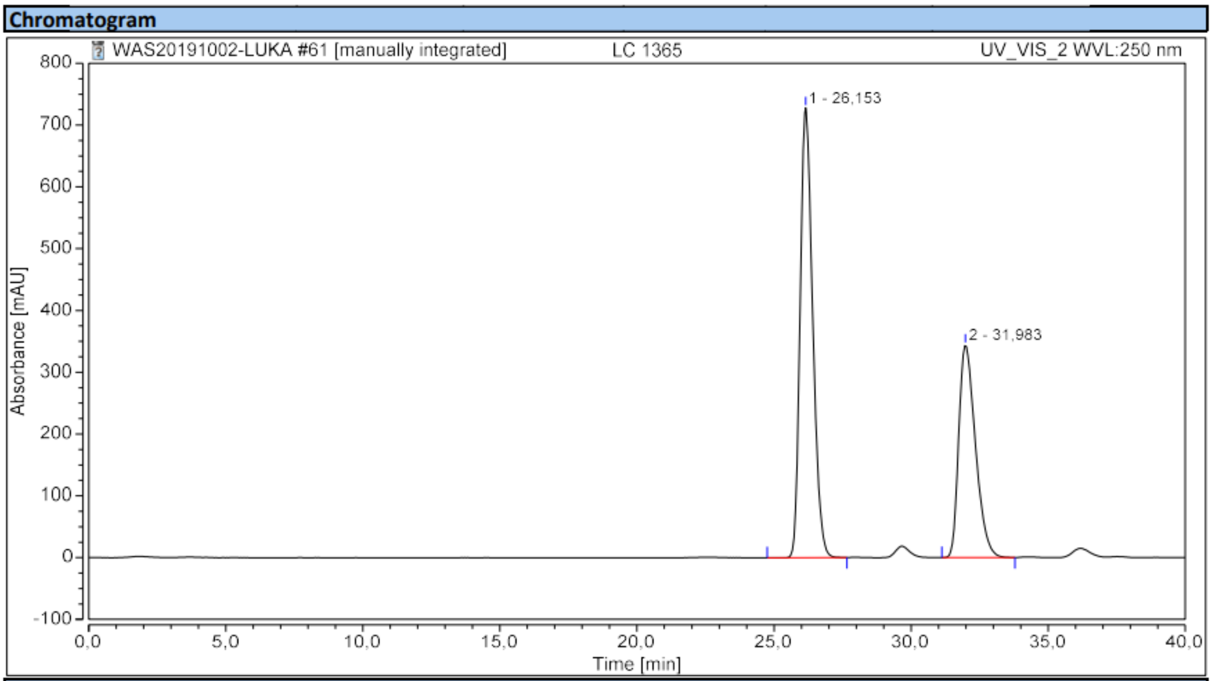

Integration Results

No.	Peak Name	Retention Time min	Area mAU*min	Height mAU	Relative Area %	Relative Height %	Amount n.a.
1		26,153	384,712	727,676	61,36	67,92	n.a.
2		31,983	242,288	343,744	38,64	32,08	n.a.
**Total:**			**627,000**	**1071,419**	**100,00**	**100,00**	

Table S2, Entry 6

	Catalyst		Base	Yield (%)	*ee* (%)
6	**IX**	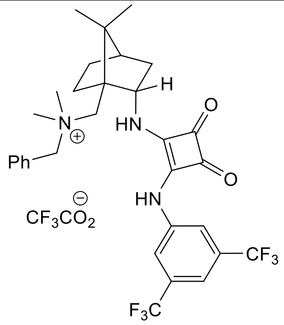	$K_3PO_{4(aq)}$ (1 eq.)	80	26 (*S*)

HPLC: Chiralpak AD-H, *n*-Hexane/*i*PrOH = 200:1, flow rate 0.75 mL/min, λ = 250 nm, 10°C

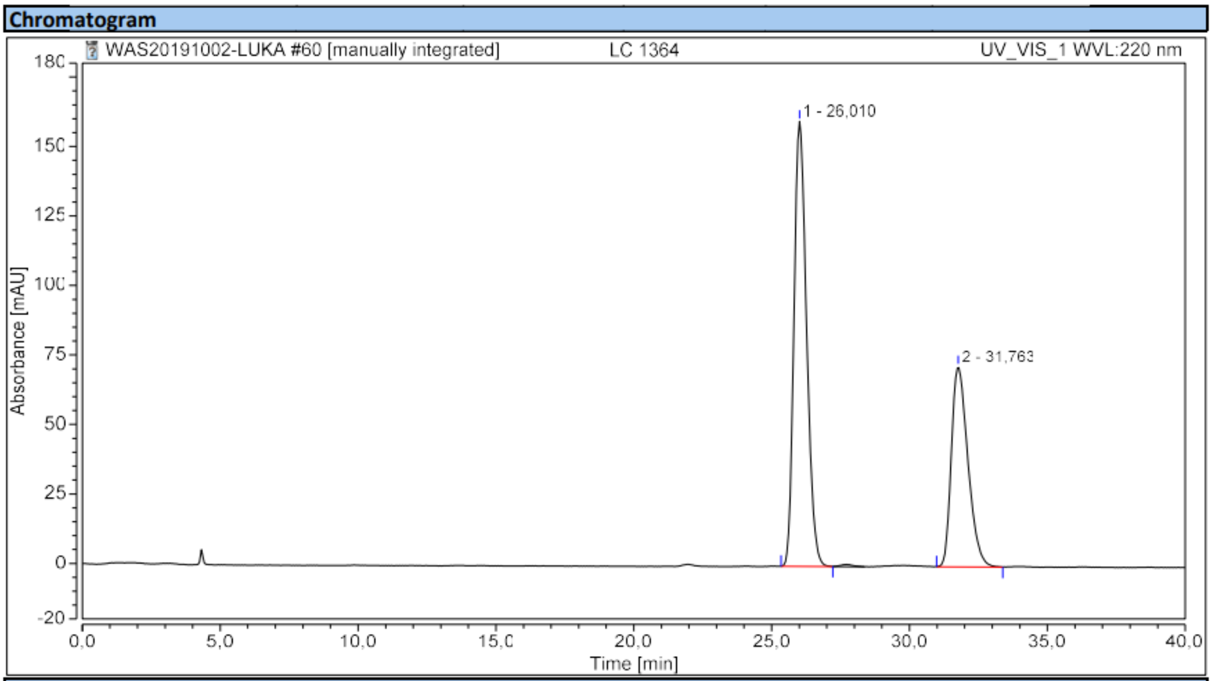

Integration Results

No.	Peak Name	Retention Time min	Area mAU*min	Height mAU	Relative Area %	Relative Height %	Amount n.a.
1		26,010	84,429	160,183	62,77	68,98	n.a.
2		31,763	50,072	72,048	37,23	31,02	n.a.
**Total:**			**134,501**	**232,231**	**100,00**	**100,00**	

Table S2, Entry 7

	Catalyst		Base	Yield (%)	*ee* (%)
7	**IX**	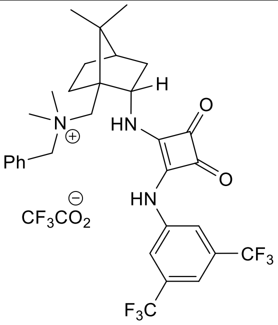	$K_3PO_{4(aq)}$ (2 eq.)	98	19 (*S*)

HPLC: Chiralpak AD-H, *n*-Hexane/*i*PrOH = 200:1, flow rate 0.75 mL/min, λ = 250 nm, 10°C

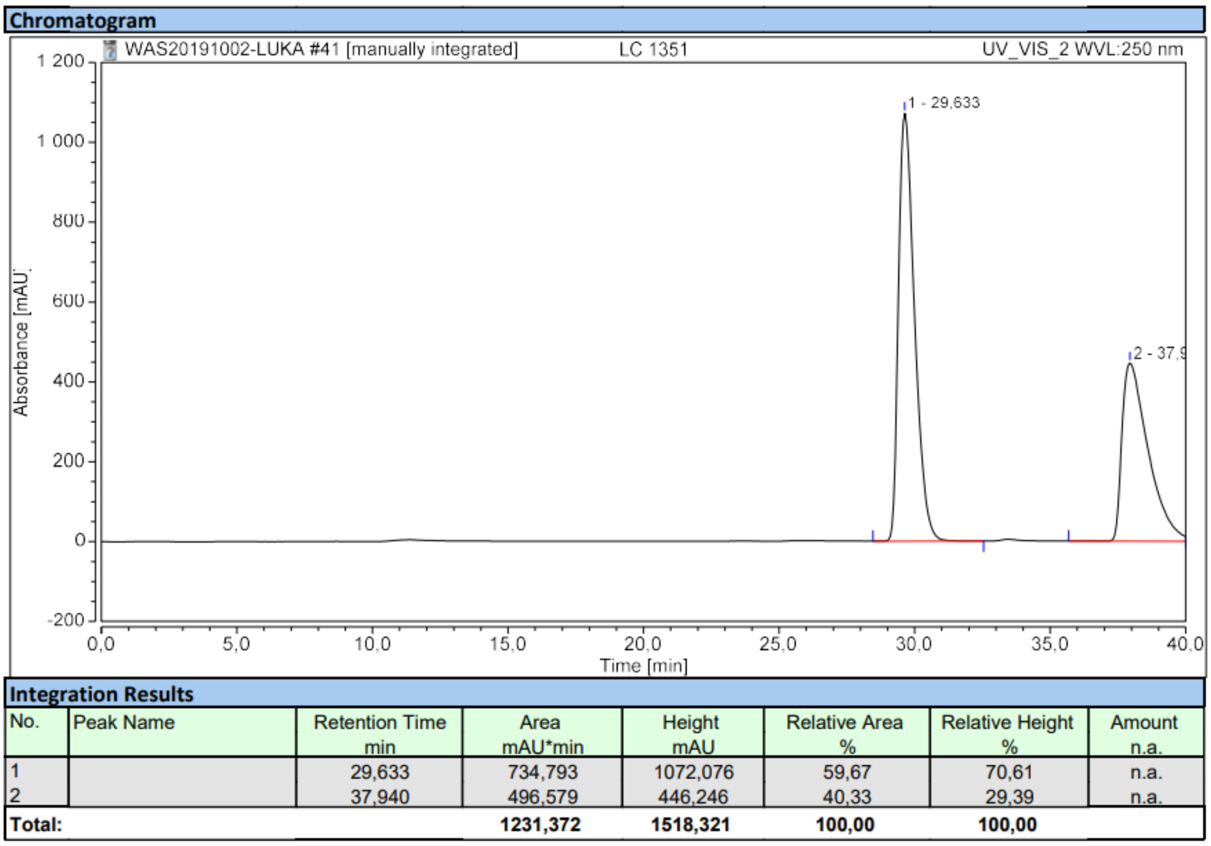

Integration Results

No.	Peak Name	Retention Time min	Area mAU*min	Height mAU	Relative Area %	Relative Height %	Amount n.a.
1		29,633	734,793	1072,076	59,67	70,61	n.a.
2		37,940	496,579	446,246	40,33	29,39	n.a.
**Total:**			**1231,372**	**1518,321**	**100,00**	**100,00**	

Table S2, Entry 8

	Catalyst		Base	Yield (%)	*ee* (%)
8	**IX**	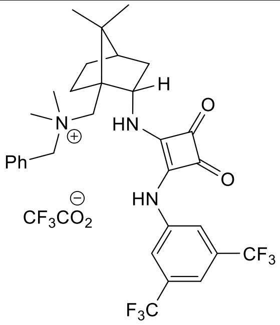	$K_2HPO_{4(aq)}$ (2 eq.)	96	9 (*S*)

HPLC: Chiralpak AD-H, *n*-Hexane/*i*PrOH = 200:1, flow rate 0.75 mL/min, λ = 250 nm, 10°C

Chromatogram

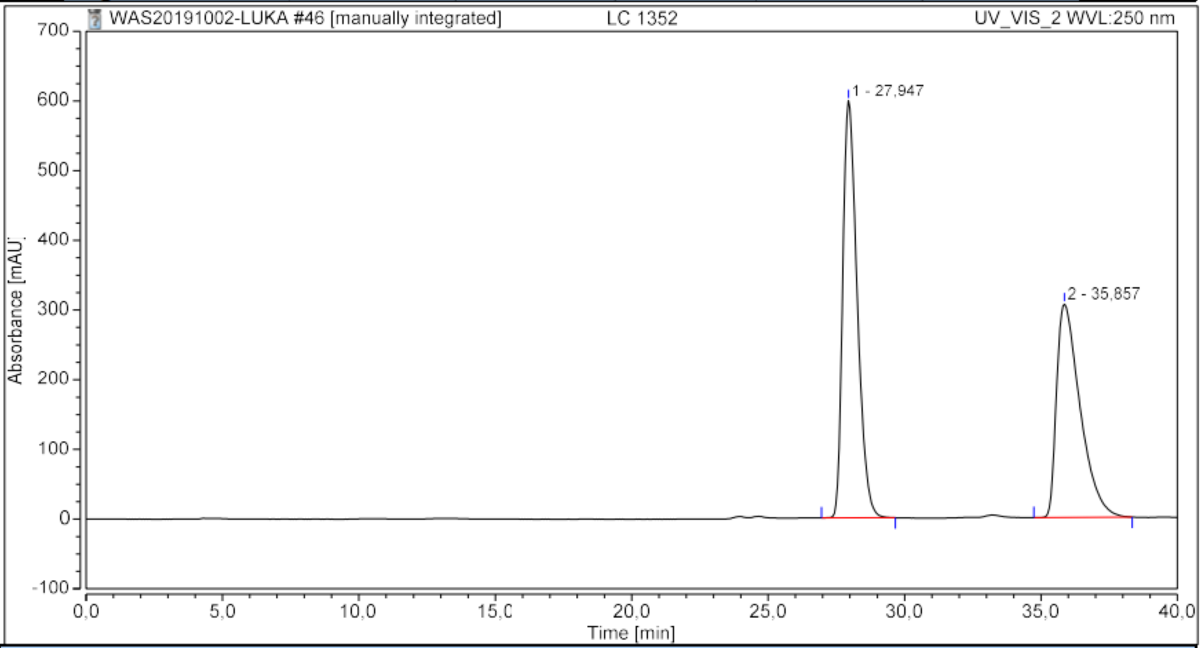

Integration Results

No.	Peak Name	Retention Time min	Area mAU*min	Height mAU	Relative Area %	Relative Height %	Amount n.a.
1		27,947	368,111	598,349	54,60	66,15	n.a.
2		35,857	306,136	306,251	45,40	33,85	n.a.
**Total:**			**674,247**	**904,599**	**100,00**	**100,00**	

Table S2, Entry 9

	Catalyst		Base	Yield (%)	*ee* (%)
9	**IX**	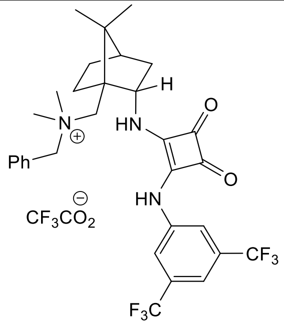	$Cs_2CO_{3(aq)}$ (2 eq.)	49	19 (*S*)

HPLC: Chiralpak AD-H, *n*-Hexane/*i*PrOH = 200:1, flow rate 0.75 mL/min, λ = 250 nm, 10°C

Chromatogram

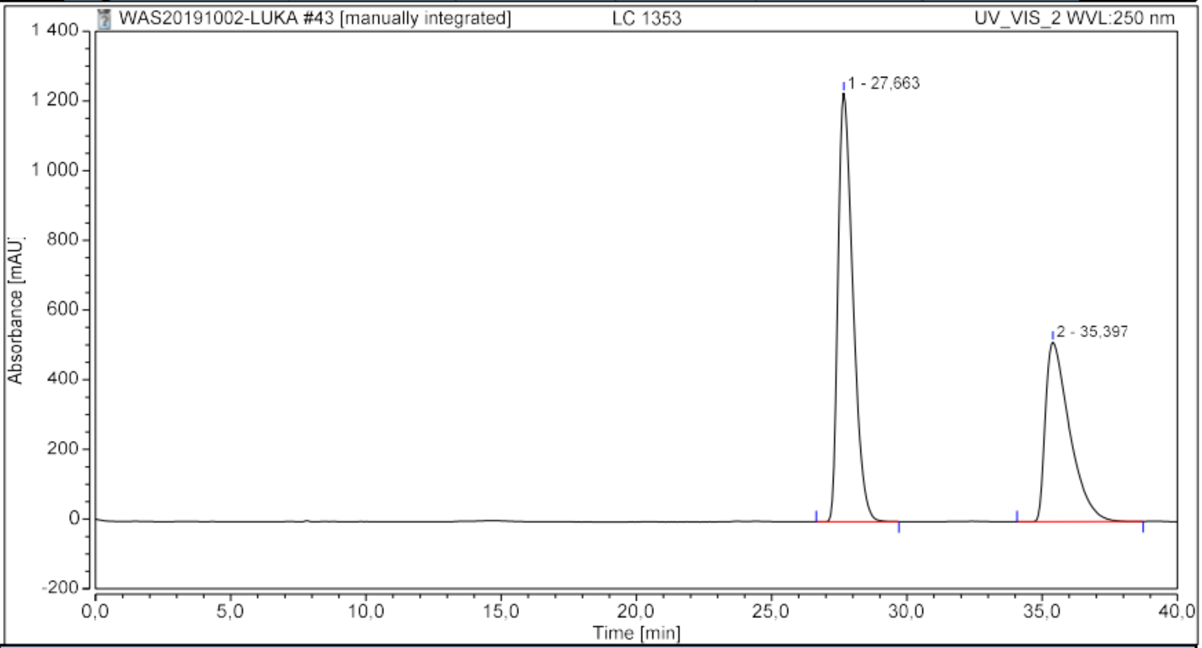

Integration Results

No.	Peak Name	Retention Time min	Area mAU*min	Height mAU	Relative Area %	Relative Height %	Amount n.a.
1		27,663	789,287	1230,343	59,69	70,48	n.a.
2		35,397	533,047	515,228	40,31	29,52	n.a.
**Total:**			**1322,333**	**1745,571**	**100,00**	**100,00**	

Table S2, Entry 10

	**Catalyst**		**Base**	**Yield (%)**	***ee* (%)**
10	**IX**	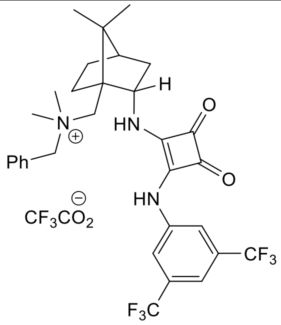	$K_2CO_{3(aq)}$ (2 eq.)	51	14 (*S*)

HPLC: Chiralpak AD-H, *n*-Hexane/*i*PrOH = 200:1, flow rate 0.75 mL/min, λ = 250 nm, 10°C

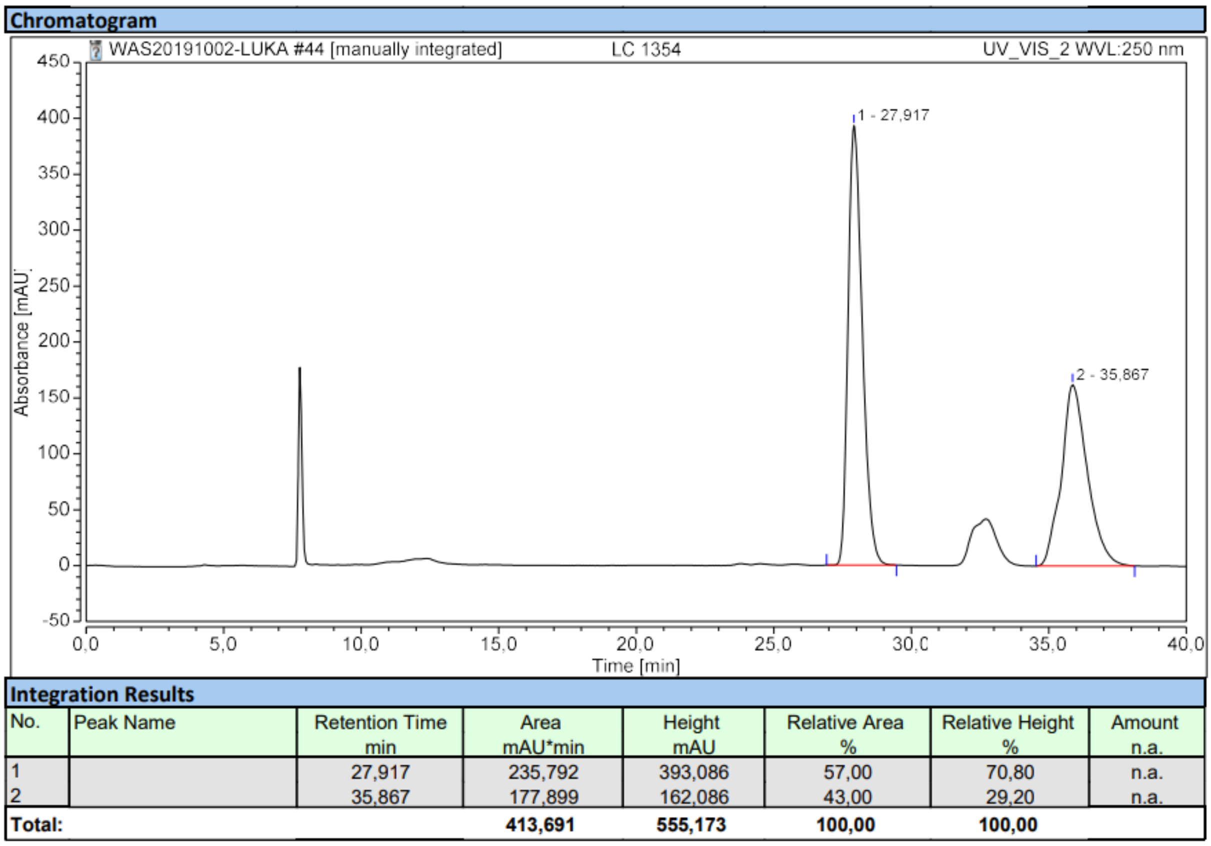

Integration Results

No.	Peak Name	Retention Time min	Area mAU*min	Height mAU	Relative Area %	Relative Height %	Amount n.a.
1		27,917	235,792	393,086	57,00	70,80	n.a.
2		35,867	177,899	162,086	43,00	29,20	n.a.
**Total:**			**413,691**	**555,173**	**100,00**	**100,00**	

Table S3. Evaluation of organocatalysts **II**, **III**, **VI–VIII**, and **IX** in the chlorination of *β*-keto ester **9**.[4] [a]

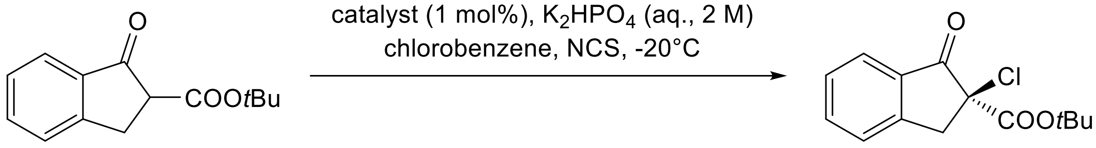

9 **11**

**Entry**	**Catalyst**	**Yield [b] (%)**	***ee* [c] (%)**
1	**II**	73	0
2	**III**	27	0
3	**VI**	55	0
4	**VII**	27	0
5	**VIII**	64	0
6	**IX**	98	7 (*S*)

[a] To a mixture of *β*-keto ester **9** (0.1 mmol, 24.4 mg, ω = 95%), organocatalyst **II**, **III**, **VI–VIII**, or **IX** (1 mol%), K_2HPO_4 (solid, 1 equivalent, 17.4 mg) in chlorobenzene (2 mL) at –20°C under argon atmosphere was added *N*-chlorosuccinimide (NCS, 1.2 equivalents, 16 mg) and the reaction mixture was stirred for 2 h at –20°C. After completion, the reaction was quenched by addition of NH_4Cl (aq. sat, 4 mL) and extracted with CH_2Cl_2 (10 mL). The organic phase was dried over anhydrous Na_2SO_4, filtered, and the volatiles were evaporated *in vacuo*. The residue was purified by column chromatography (Silica gel 60, EtOAc/*n*-Heptane = 1 : 12). [b] Reaction yield determined after isolation with column chromatography (Silica gel 60, EtOAc/*n*-Heptane = 1 : 12). [c] *ee* determined by HPLC (Chiralpak OJ-H, *n*-Hexane/*i*PrOH = 70:30, flow rate 0.7 mL/min, λ = 250 nm, 10°C) isolation with column chromatography (Silica gel 60, EtOAc/*n*-Heptane = 1 : 12).

Table S3, Entry 1

		Catalyst	Yield (%)	*ee* (%)
1	**II**	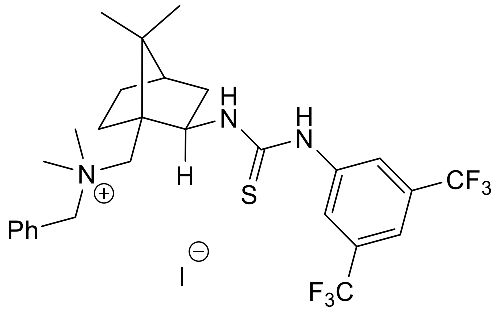	73	0

HPLC: Chiralpak OJ-H, *n*-Hexane/*i*PrOH = 70:30, flow rate 0.7 mL/min, λ = 250 nm, 10℃

Chromatogram

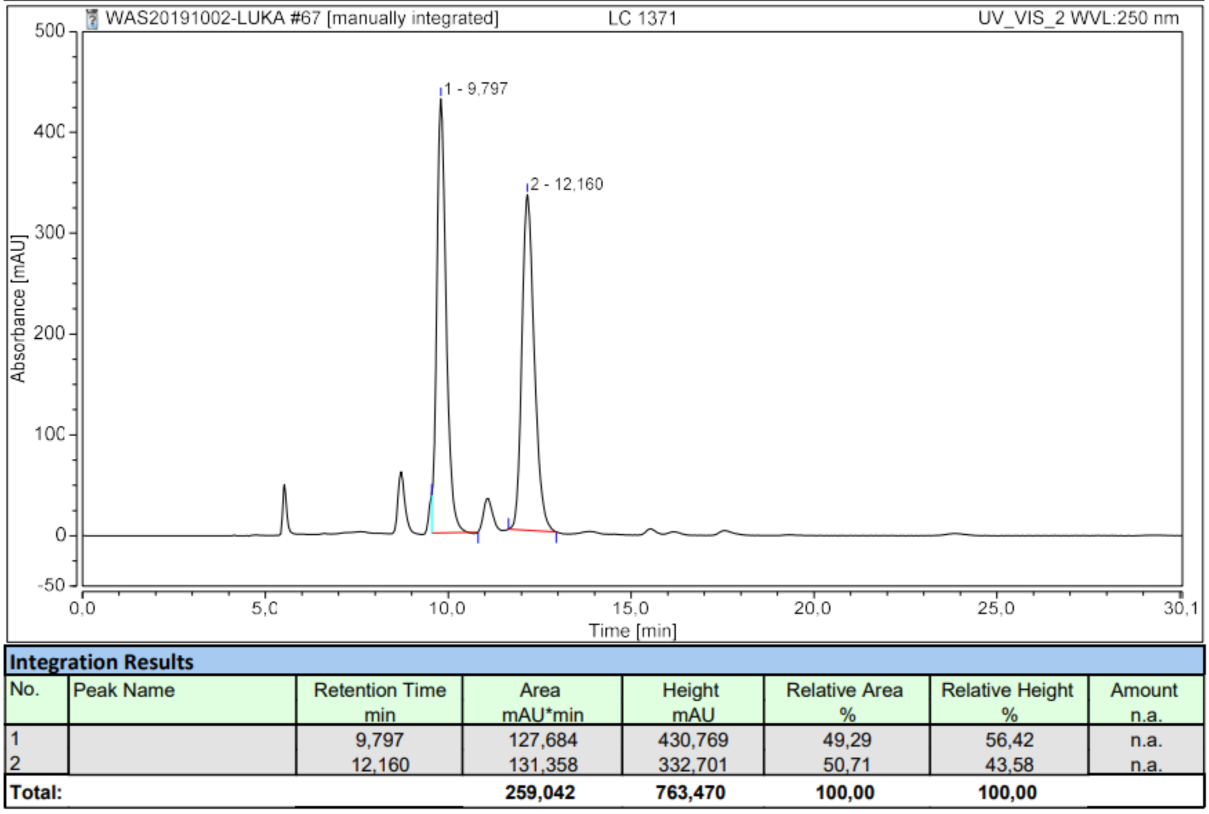

Integration Results

No.	Peak Name	Retention Time min	Area mAU*min	Height mAU	Relative Area %	Relative Height %	Amount n.a.
1		9,797	127,684	430,769	49,29	56,42	n.a.
2		12,160	131,358	332,701	50,71	43,58	n.a.
**Total:**			**259,042**	**763,470**	**100,00**	**100,00**	

Table S3, Entry 2

	Catalyst		Yield (%)	*ee* (%)
2	**III**	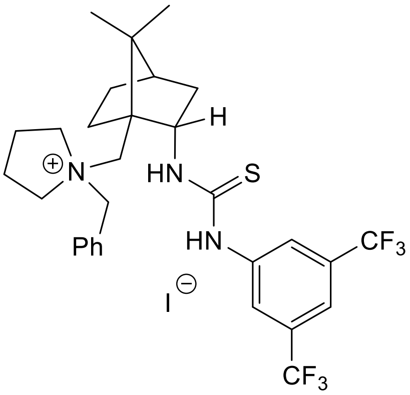	27	0

HPLC: Chiralpak OJ-H, *n*-Hexane/*i*PrOH = 70:30, flow rate 0.7 mL/min, λ = 250 nm, 10°C

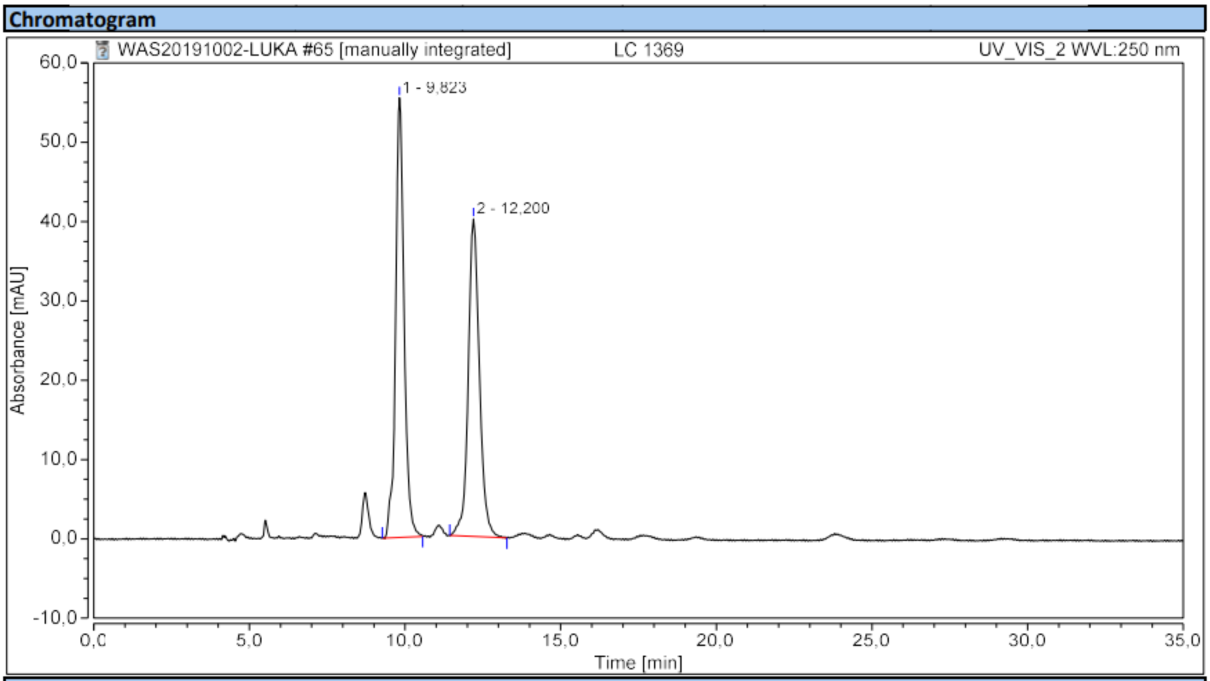

Integration Results

No.	Peak Name	Retention Time min	Area mAU*min	Height mAU	Relative Area %	Relative Height %	Amount n.a.
1		9,823	17,307	55,402	50,98	58,05	n.a.
2		12,200	16,643	40,030	49,02	41,95	n.a.
**Total:**			**33,950**	**95,432**	**100,00**	**100,00**	

Table S3, Entry 3

	Catalyst		Yield (%)	*ee* (%)
3	**VI**	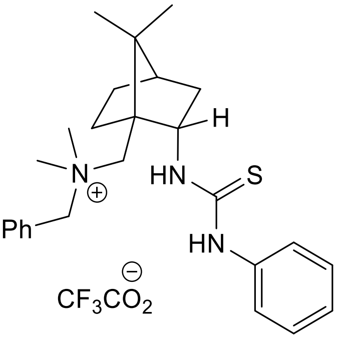	55	0

HPLC: Chiralpak OJ-H, *n*-Hexane/*i*PrOH = 70:30, flow rate 0.7 mL/min, λ = 250 nm, 10°C

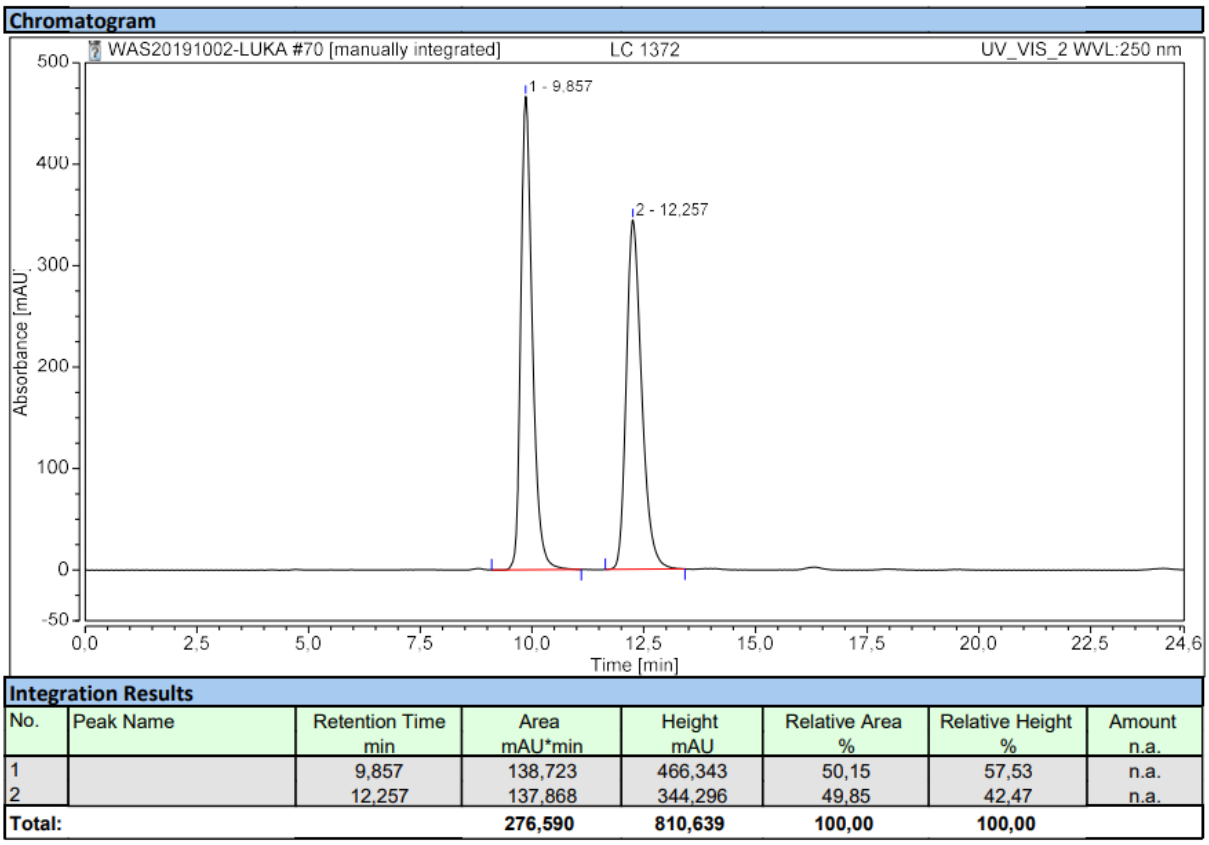

Integration Results

No.	Peak Name	Retention Time min	Area mAU*min	Height mAU	Relative Area %	Relative Height %	Amount n.a.
1		9,857	138,723	466,343	50,15	57,53	n.a.
2		12,257	137,868	344,296	49,85	42,47	n.a.
**Total:**			**276,590**	**810,639**	**100,00**	**100,00**	

Table S3, Entry 4

	Catalyst		Yield (%)	*ee* (%)
4	**VII**	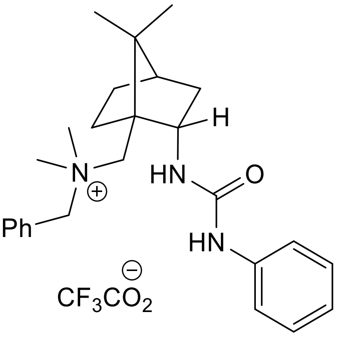	27	0

HPLC: Chiralpak OJ-H, *n*-Hexane/*i*PrOH = 70:30, flow rate 0.7 mL/min, λ = 250 nm, 10°C

Chromatogram

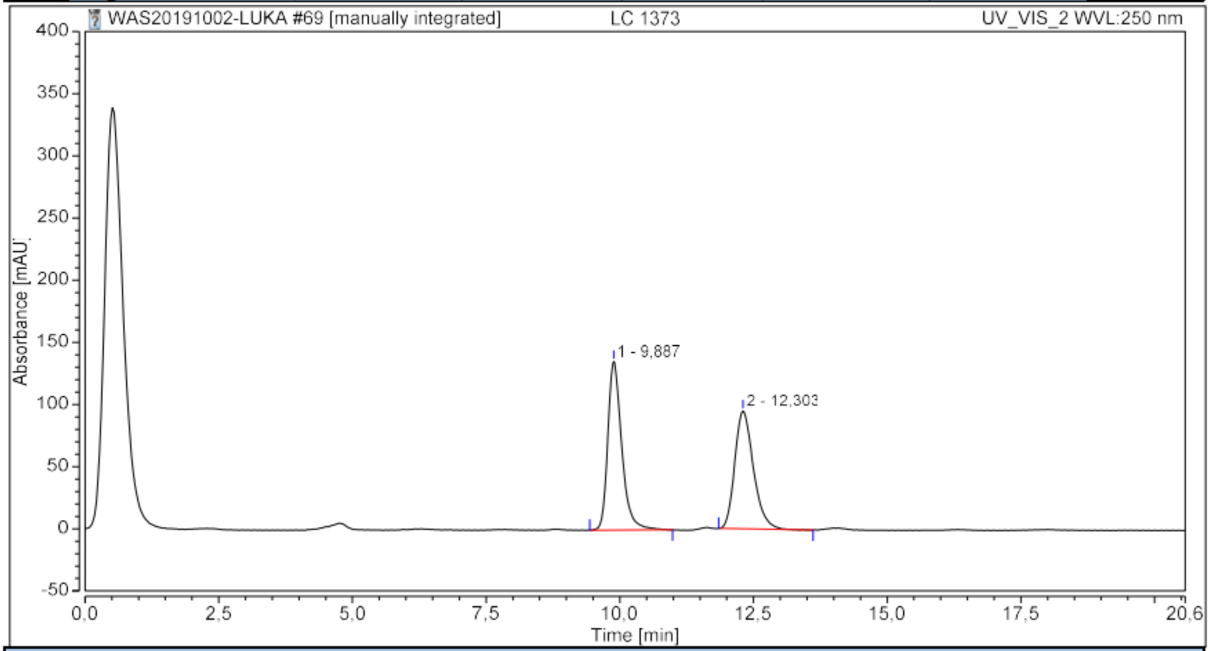

Integration Results

No.	Peak Name	Retention Time min	Area mAU*min	Height mAU	Relative Area %	Relative Height %	Amount n.a.
1		9,887	40,996	135,632	51,70	58,82	n.a.
2		12,303	38,304	94,946	48,30	41,18	n.a.
**Total:**			**79,300**	**230,578**	**100,00**	**100,00**	

Table S3, Entry 5

	Catalyst		Yield (%)	*ee* (%)
5	**VIII**	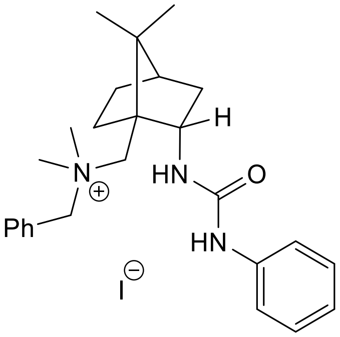	64	0

HPLC: Chiralpak OJ-H, *n*-Hexane/*i*PrOH = 70:30, flow rate 0.7 mL/min, λ = 250 nm, 10°C

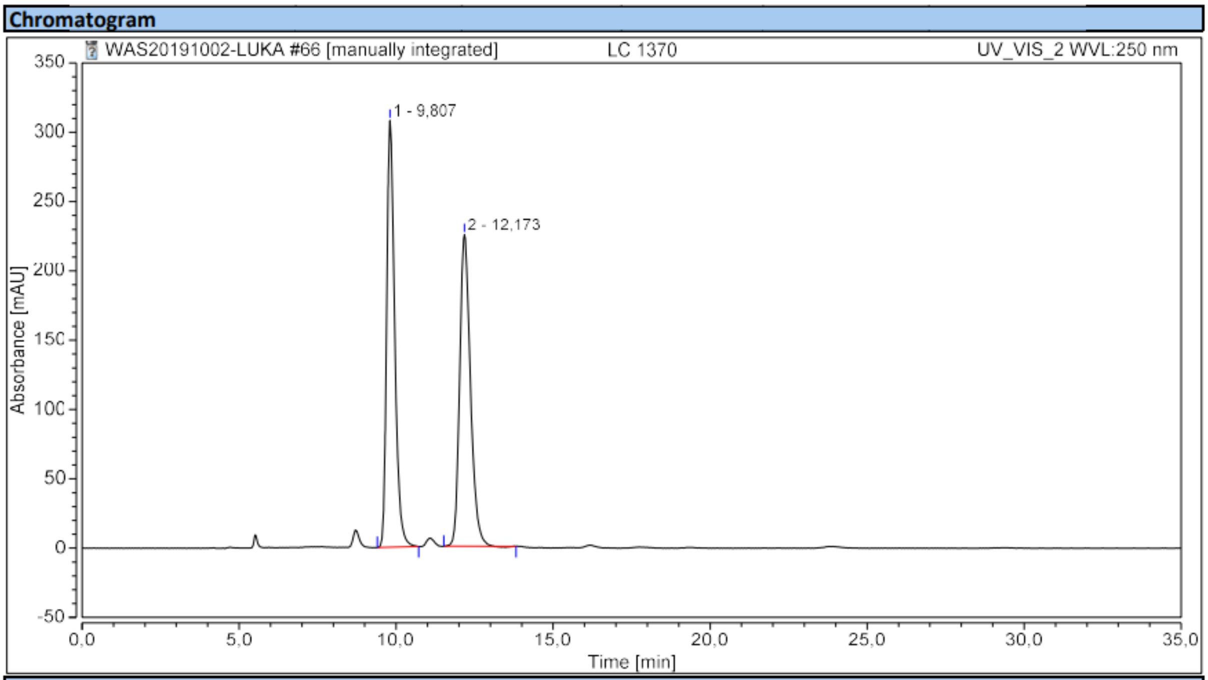

Integration Results

No.	Peak Name	Retention Time min	Area mAU*min	Height mAU	Relative Area %	Relative Height %	Amount n.a.
1		9,807	90,533	307,795	50,35	57,79	n.a.
2		12,173	89,291	224,781	49,65	42,21	n.a.
**Total:**			**179,824**	**532,576**	**100,00**	**100,00**	

Table S3, Entry 6

	Catalyst		Yield (%)	*ee* (%)
6	**IX**	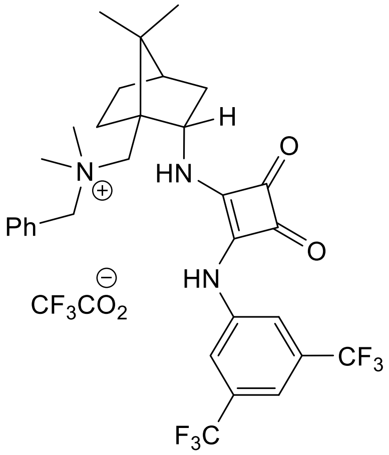	98	7

HPLC: Chiralpak OJ-H, *n*-Hexane/*i*PrOH = 70:30, flow rate 0.7 mL/min, λ = 250 nm, 10°C

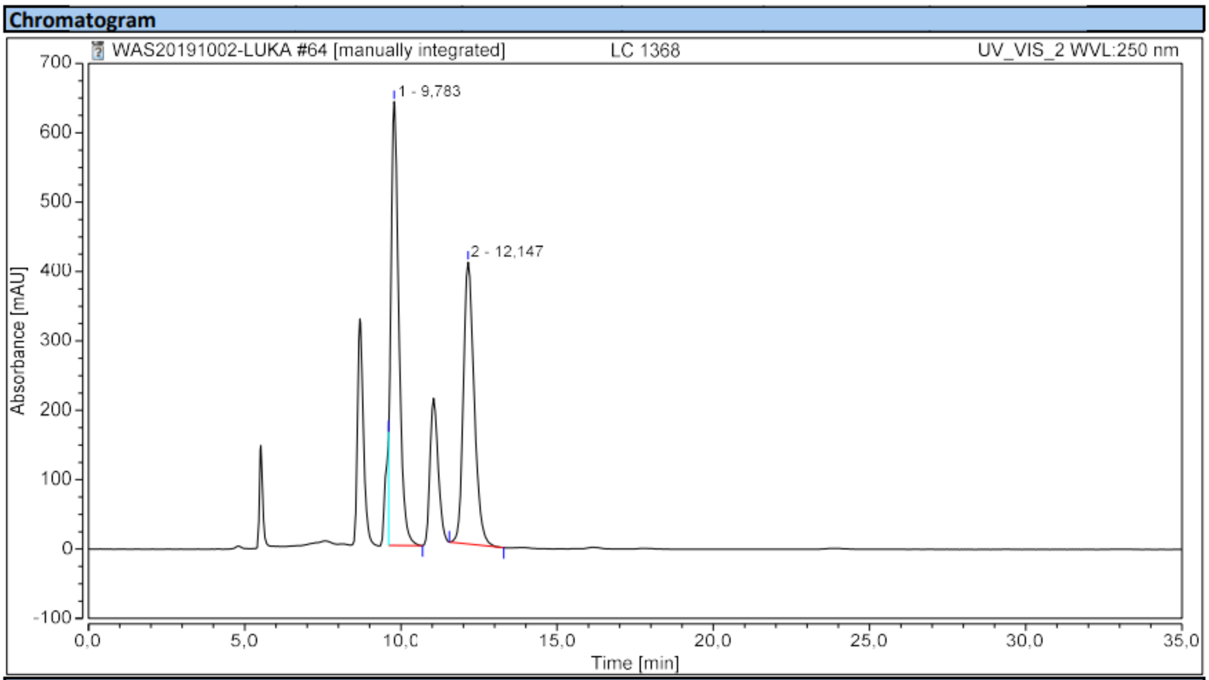

Integration Results

No.	Peak Name	Retention Time min	Area mAU*min	Height mAU	Relative Area %	Relative Height %	Amount n.a.
1		9,783	187,679	639,838	53,56	61,16	n.a.
2		12,147	162,720	406,257	46,44	38,84	n.a.
**Total:**			**350,399**	**1046,095**	**100,00**	**100,00**	

Table S4. Evaluation of organocatalysts **III**, **IV**, **VII**, **VIII**, and **IX** in the hydroxylation of *β*-keto ester **9**.[5] [a]

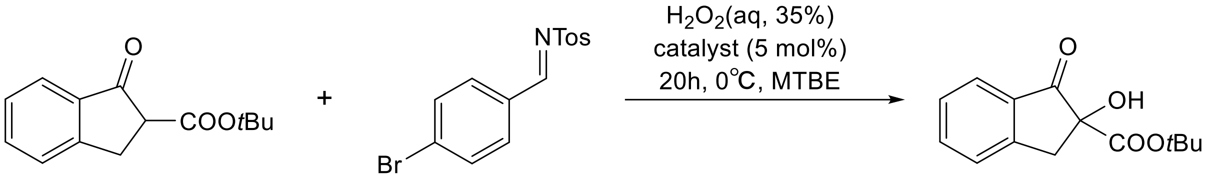

9 **12** **13**

**Entry**	**Catalyst**	**Yield (%)**	***ee* (%)**
1	**III**	no conversion	-
2	**IV**	no conversion	-
3	**VII**	no conversion	-
4	**VIII**	no conversion	-
5	**IX**	no conversion	-

[a] Into a flame dried Schlenk flask under argon atmosphere at 0°C, a mixture of *β*-keto ester **9** (0.1 mmol, 24.4 mg, ω = 95%), and *N*-(4-bromobenzylidene)-4-methylbenzenesulfonamide (**12**) (1 equivalent, 33.8 mg) was added. Catalyst **III**, **IV**, **VII**, **VIII**, or **IX** (5 mol%) was dissolved in anhydrous methyl *tert*-butyl ether (MTBE, 5 mL) and slowly added via syringe into the reaction mixture. After addition of H_2O_2 (1 equivalent, 35% in water, 8.6 µL), the reaction mixture was stirred for 20 h at room temperature. After 24 h at 25°C, the reaction mixture was filtrated trough a plug of anhydrous Na_2SO_4 and washed with dichloromethane.

Table S5. Evaluation of organocatalysts **IV**, **VIII**, and **IX** in the addition of *β*-keto ester **9** to tosylaziridine **14**.[6] [a]

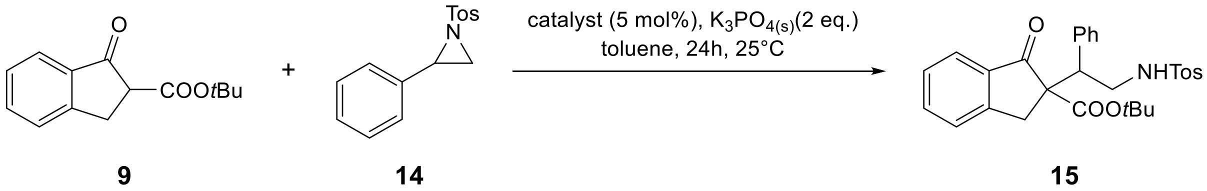

Entry	Catalyst	Yield (%)	*ee* (%)
1	**IV**	no conversion	-
2	**VIII**	no conversion	-
3	**IX**	no conversion	-

[a] To a mixture of *β*-keto ester **9** (0.1 mmol, 24.4 mg, ω = 95%), catalyst **IV**, **VIII**, or **IX** (5 mol%), and K_3PO_4 (2 equivalents, 42 mg) in toluene (2.5 mL) under argon atmosphere 2-phenyl-1-tosylaziridine (**14**) (2 equivalent, 54.6 mg) was added and stirred at room temperature for 24 h. After 24 h at 25°C, the reaction mixture was filtrated trough a plug of anhydrous Na_2SO_4 and washed with dichloromethane.

Table S6. Evaluation of organocatalysts **I**, **III**–**V**, **VII**, **VIII**, and **IX** in the addition of *tert*-butyl 2-((diphenylmethylene)amino)acetate (**16**) to methyl acrylate (**17**).[7] [a]

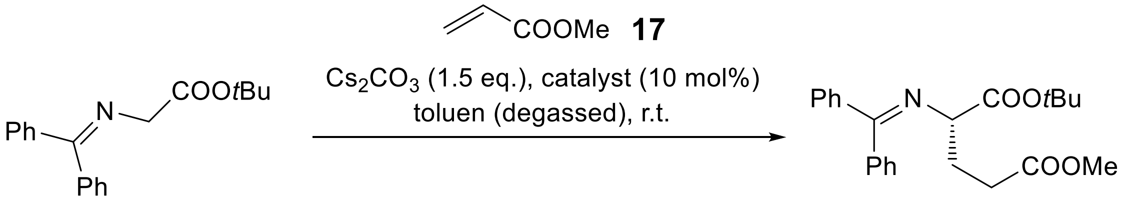

16 **18**

**Entry**	**Catalyst**	**Yield** [b] **(%)**	***ee*** [c] **(%)**
1	**I**	no conversion	-
2	**III**	79	5 (*S*)
3	**IV**	no conversion	-
4	**V**	63	6 (*S*)
5	**VII**	no conversion	-
6	**VIII**	73	10 (*S*)
7	**IX**	no conversion	-
8 [d]	**V**	58	11 (*S*)
9 [e]	**V**	98	1 (*S*)
10 [f]	**V**	no conversion	-
11 [g]	**IV**	no conversion	-
12 [h]	**IV**	58	1 (*S*)

[a] Degassed toluene (2.5 mL) was added to a mixture of *tert*-butyl 2-((diphenylmethylene)amino)acetate (**16**) (0.05 mmol, 14.8 mg), catalyst **I**, **III**–**V**, **VII**, **VIII**, or **IX** (10 mol%), and Cs_2CO_3 (1.5 equivalents, 48.8 mg) in a Schlenk tube, then methyl acrylate (**17**) (1.5 equivalents, 6.8 µL) was added. After 24 h at 25°C, the reaction mixture was filtrated trough a plug of anhydrous Na_2SO_4 and washed with ethyl acetate. The volatiles were evaporated *in vacuo*. The crude product **18** was purified by column chromatography (Silica gel 60, EtOAc/Heptane = 1 : 15). [b] Reaction yield determined after isolation with column chromatography (Silica gel 60, EtOAc/Heptane = 1 : 15). [c] *ee* determined by HPLC (Chiralpak AD-H, *n*-Hexane/*i*-PrOH = 95:5, flow rate 0.5 mL/min, λ = 250 nm, 10°C) after filtration trough a plug of Na_2SO_4. [d] Reaction in methyl *tert*-butyl ether (MTBE). [e] Reaction in dichloromethane. [f] Reaction proceeded at 0°C. [g] 2 equivalents of Cs_2CO_3 were used. [h] 10 equivalents of Cs_2CO_3 were used.

Table S6, Entry 2

	Catalyst		Yield (%)	*ee* (%)
2	**III**	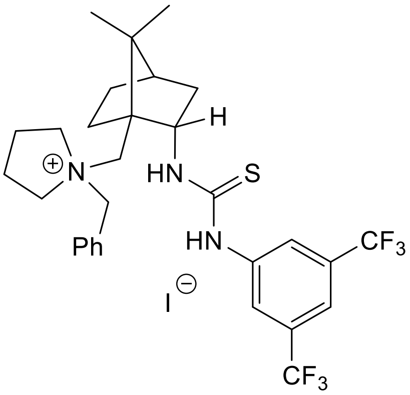	79	5 (*S*)

HPLC: Chiralpak AD-H, *n*-Hexane/*i*PrOH = 95:5, flow rate 0.5 mL/min, λ = 250 nm, 10°C

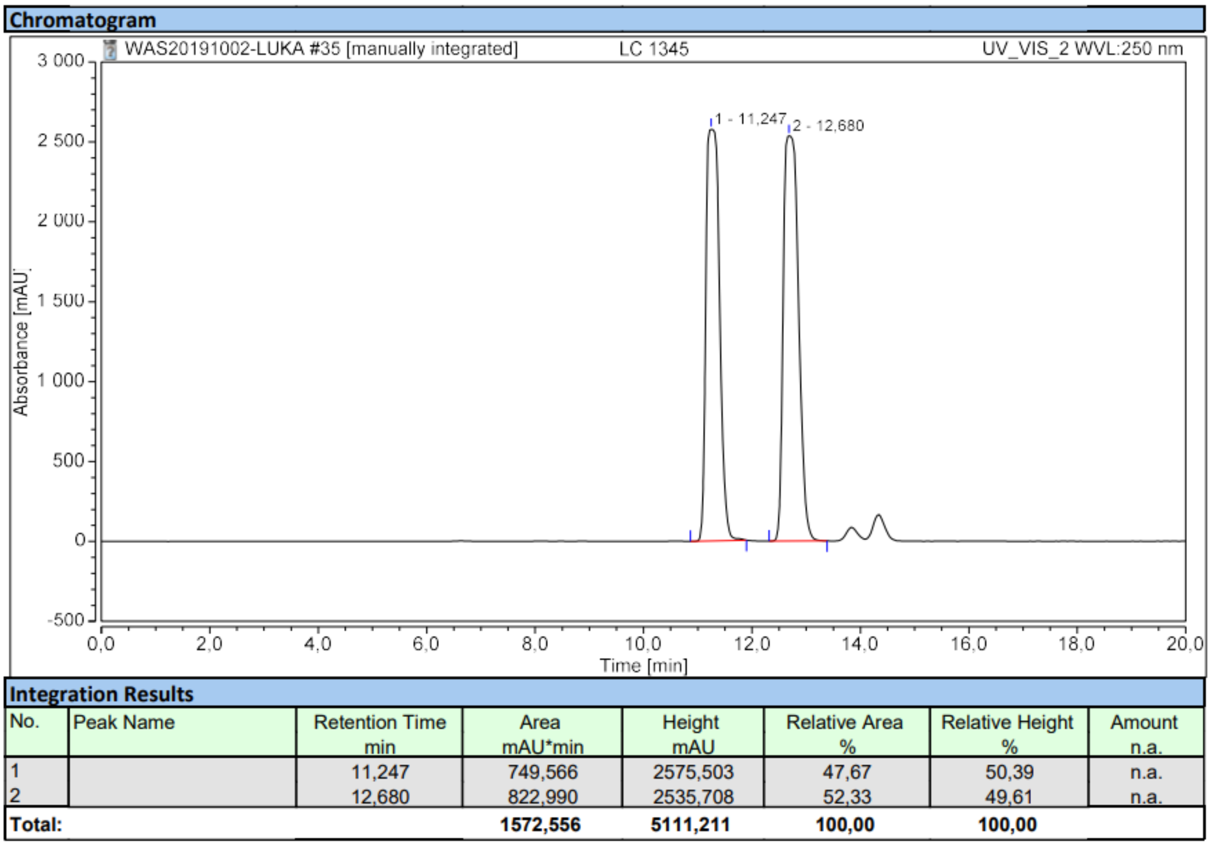

Integration Results

No.	Peak Name	Retention Time min	Area mAU*min	Height mAU	Relative Area %	Relative Height %	Amount n.a.
1		11,247	749,566	2575,503	47,67	50,39	n.a.
2		12,680	822,990	2535,708	52,33	49,61	n.a.
**Total:**			**1572,556**	**5111,211**	**100,00**	**100,00**	

Table S6, Entry 4

	Catalyst	Yield (%)	*ee* (%)
4	**V** 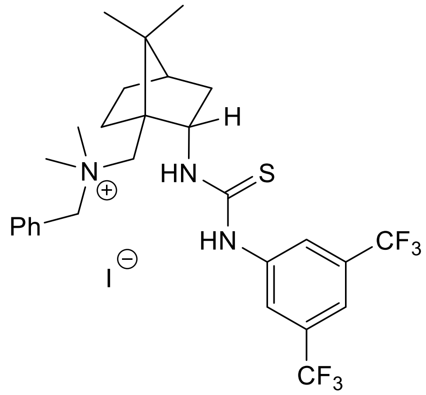	63	6 (*S*)

HPLC: Chiralpak AD-H, *n*-Hexane/*i*PrOH = 95:5, flow rate 0.5 mL/min, λ = 250 nm, 10°C

Chromatogram

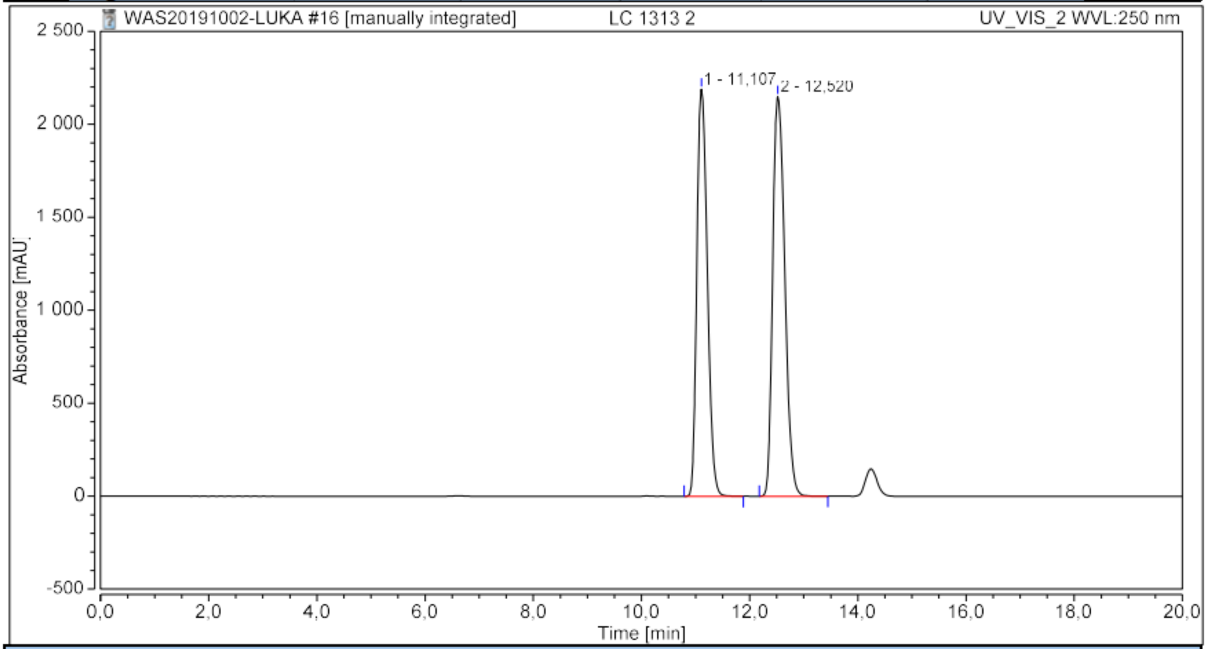

Integration Results

No.	Peak Name	Retention Time min	Area mAU*min	Height mAU	Relative Area %	Relative Height %	Amount n.a.
1		11,107	490,811	2190,670	46,79	50,47	n.a.
2		12,520	558,084	2149,622	53,21	49,53	n.a.
**Total:**			**1048,895**	**4340,292**	**100,00**	**100,00**	

Table S6, Entry 6

	Catalyst		Yield (%)	*ee* (%)
6	**VIII**	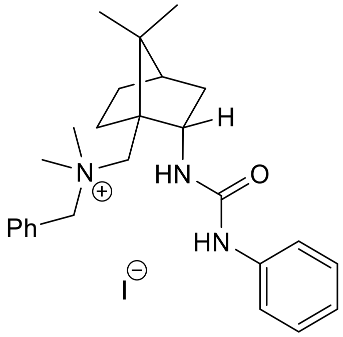	73	10 (*S*)

HPLC: Chiralpak AD-H, *n*-Hexane/*i*PrOH = 95:5, flow rate 0.5 mL/min, λ = 250 nm, 10°C

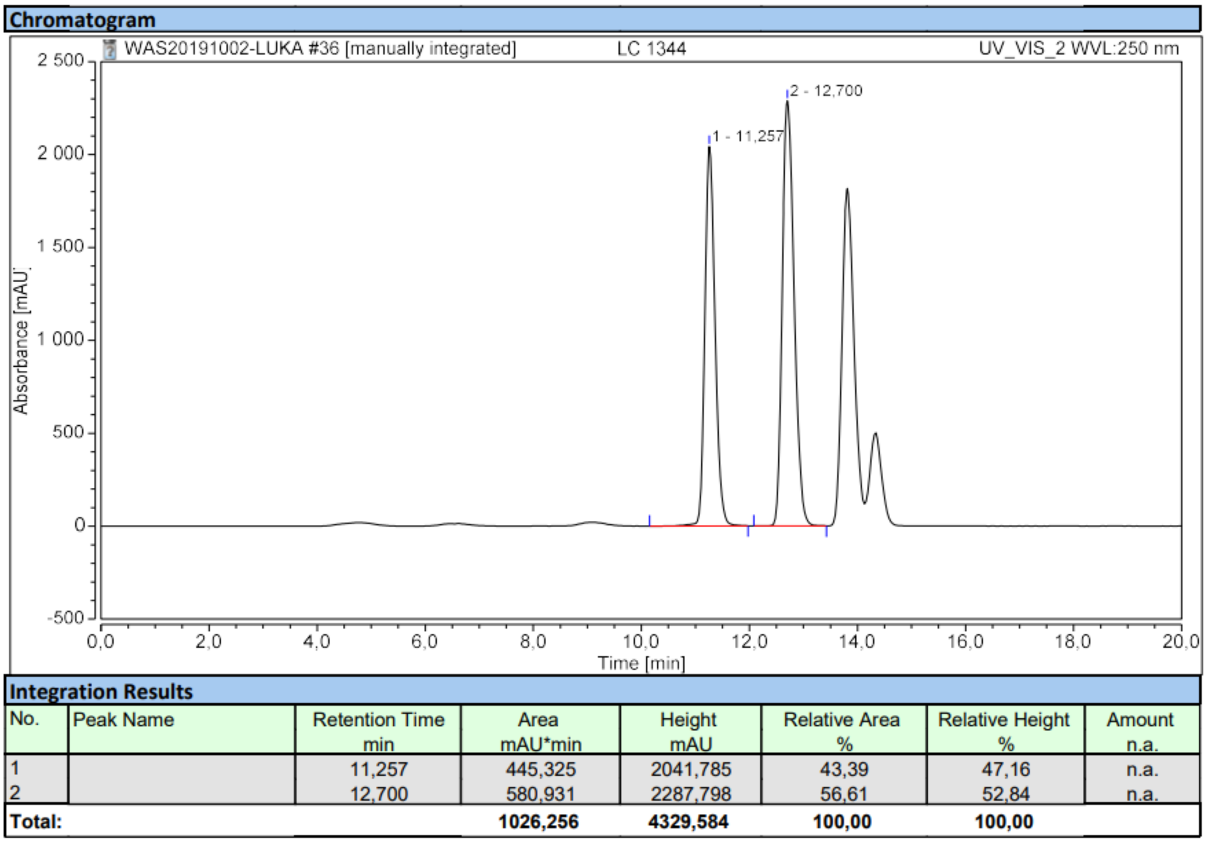

Integration Results

No.	Peak Name	Retention Time min	Area mAU*min	Height mAU	Relative Area %	Relative Height %	Amount n.a.
1		11,257	445,325	2041,785	43,39	47,16	n.a.
2		12,700	580,931	2287,798	56,61	52,84	n.a.
**Total:**			**1026,256**	**4329,584**	**100,00**	**100,00**	

Table S6, Entry 8

		Catalyst	Yield (%)	*ee* (%)
8	**V**	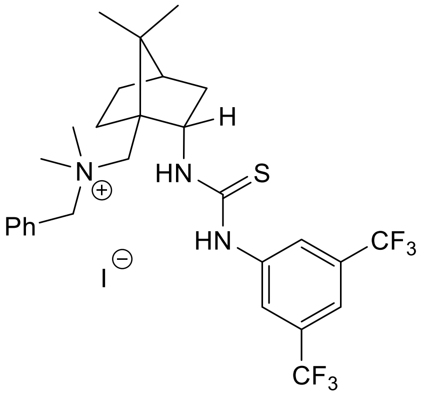	58	11 (*S*)

HPLC: Chiralpak AD-H, *n*-Hexane/*i*PrOH = 95:5, flow rate 0.5 mL/min, λ = 250 nm, 10°C

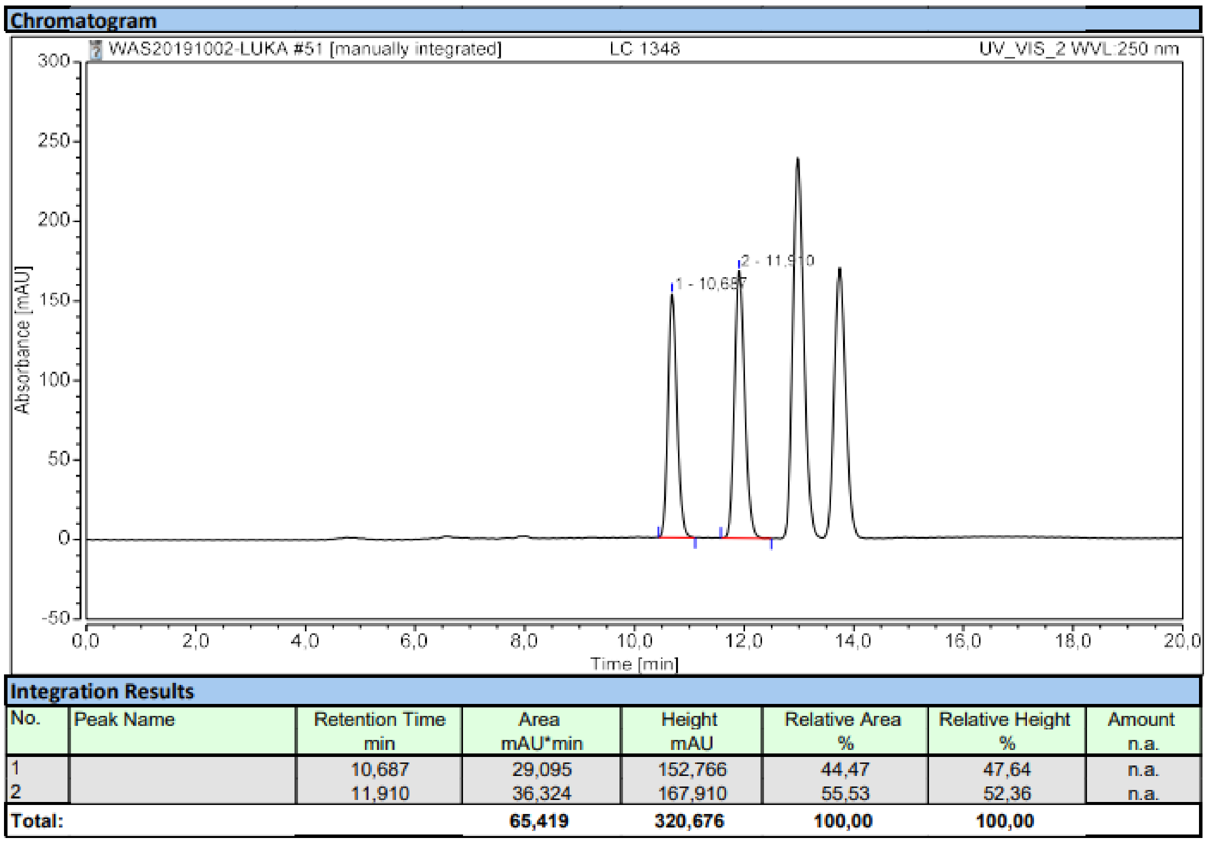

Integration Results

No.	Peak Name	Retention Time<br>min	Area<br>mAU*min	Height<br>mAU	Relative Area<br>%	Relative Height<br>%	Amount<br>n.a.
1		10,687	29,095	152,766	44,47	47,64	n.a.
2		11,910	36,324	167,910	55,53	52,36	n.a.
**Total:**			**65,419**	**320,676**	**100,00**	**100,00**	

Table S6, Entry 9

	Catalyst		Yield (%)	*ee* (%)
9	**V**	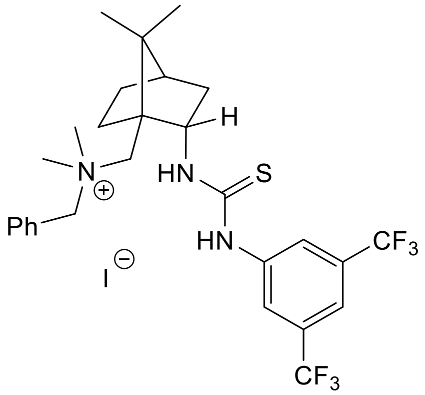	98	1 (*S*)

HPLC: Chiralpak AD-H, *n*-Hexane/*i*PrOH = 95:5, flow rate 0.5 mL/min, λ = 250 nm, 10°C

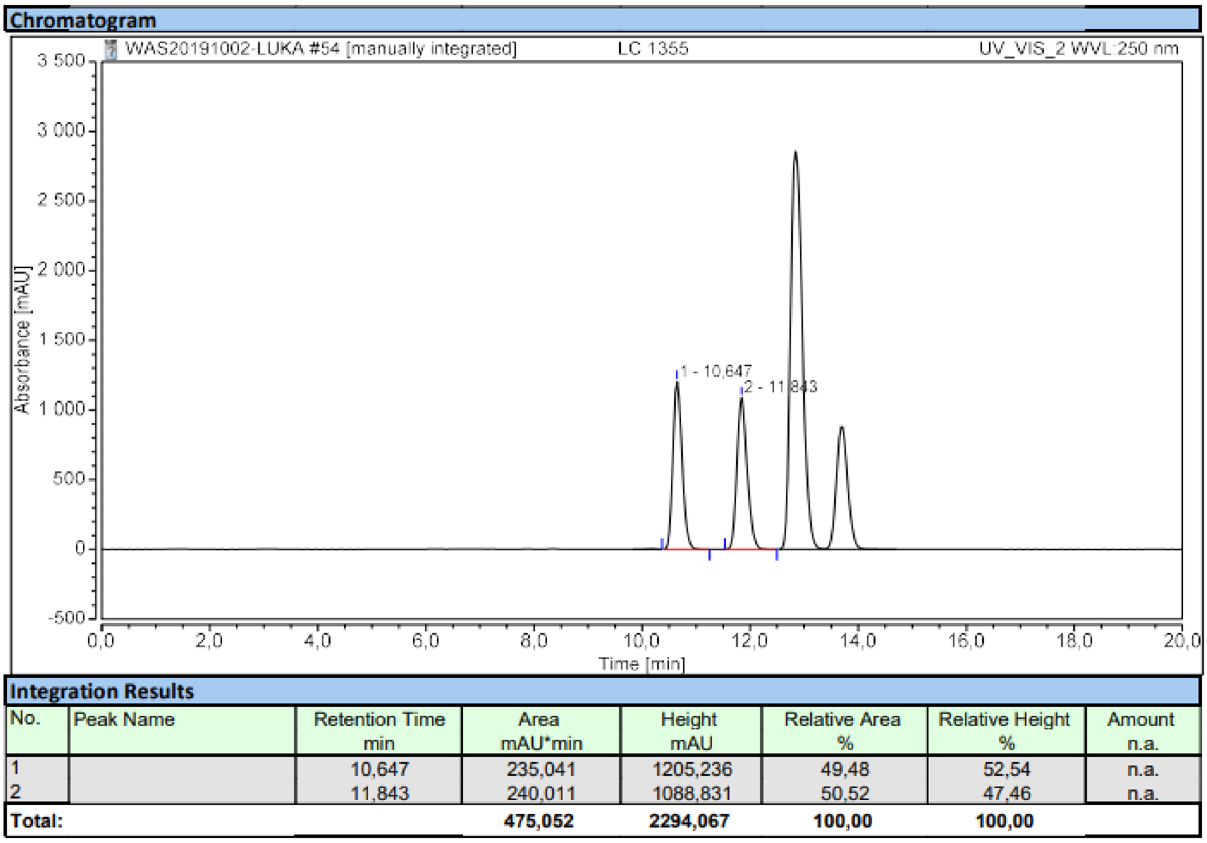

Integration Results

No.	Peak Name	Retention Time min	Area mAU*min	Height mAU	Relative Area %	Relative Height %	Amount n.a.
1		10,647	235,041	1205,236	49,48	52,54	n.a.
2		11,843	240,011	1088,831	50,52	47,46	n.a.
**Total:**			**475,052**	**2294,067**	**100,00**	**100,00**	

Table S6, Entry 12

	Catalyst		Yield (%)	*ee* (%)
12	**IV**	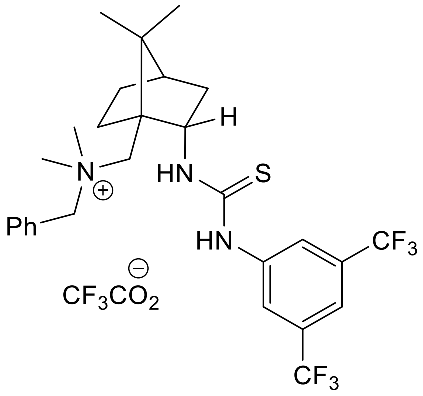	58	1 (*S*)

HPLC: Chiralpak AD-H, *n*-Hexane/*i*PrOH = 95:5, flow rate 0.5 mL/min, λ = 250 nm, 10°C

Chromatogram

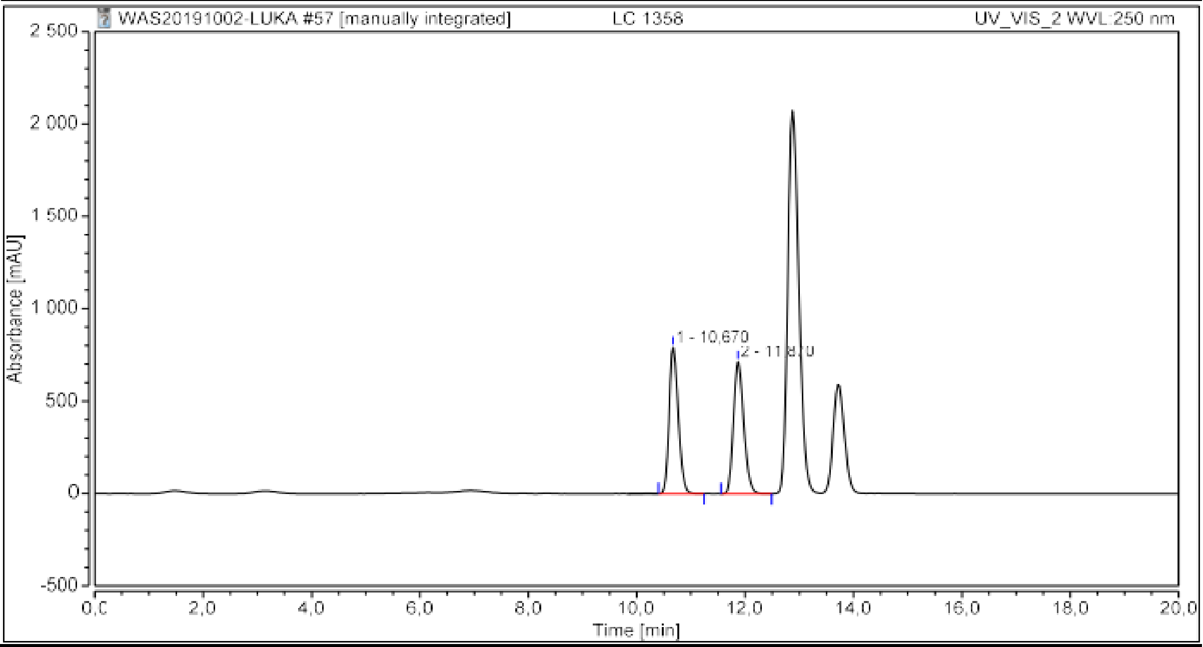

Integration Results

No.	Peak Name	Retention Time min	Area mAU*min	Height mAU	Relative Area %	Relative Height %	Amount n.a.
1		10,670	154,338	791,600	49,51	52,57	n.a.
2		11,870	157,419	714,232	50,49	47,43	n.a.
**Total:**			**311,757**	**1505,833**	**100,00**	**100,00**	

3. Copies of ^{1}H- and ^{13}C-NMR spectra

(1*S*,2*S*,4*R*)-1-((Dimethylamino)methyl)-7,7-dimethylbicyclo[2.2.1]heptan-2-amine (1a)

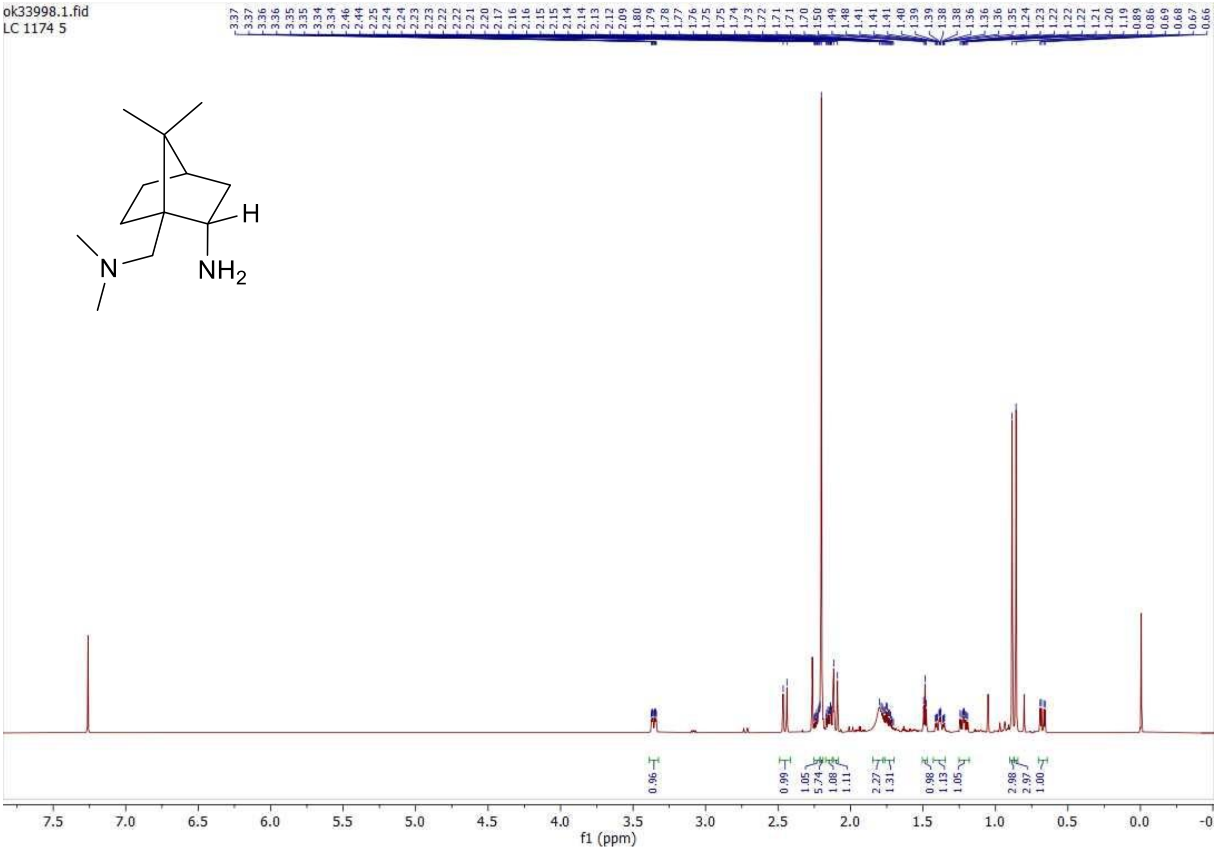

(1*S*,2*R*,4*R*)-1-((Dimethylamino)methyl)-7,7-dimethylbicyclo[2.2.1]heptan-2-amine (2a)

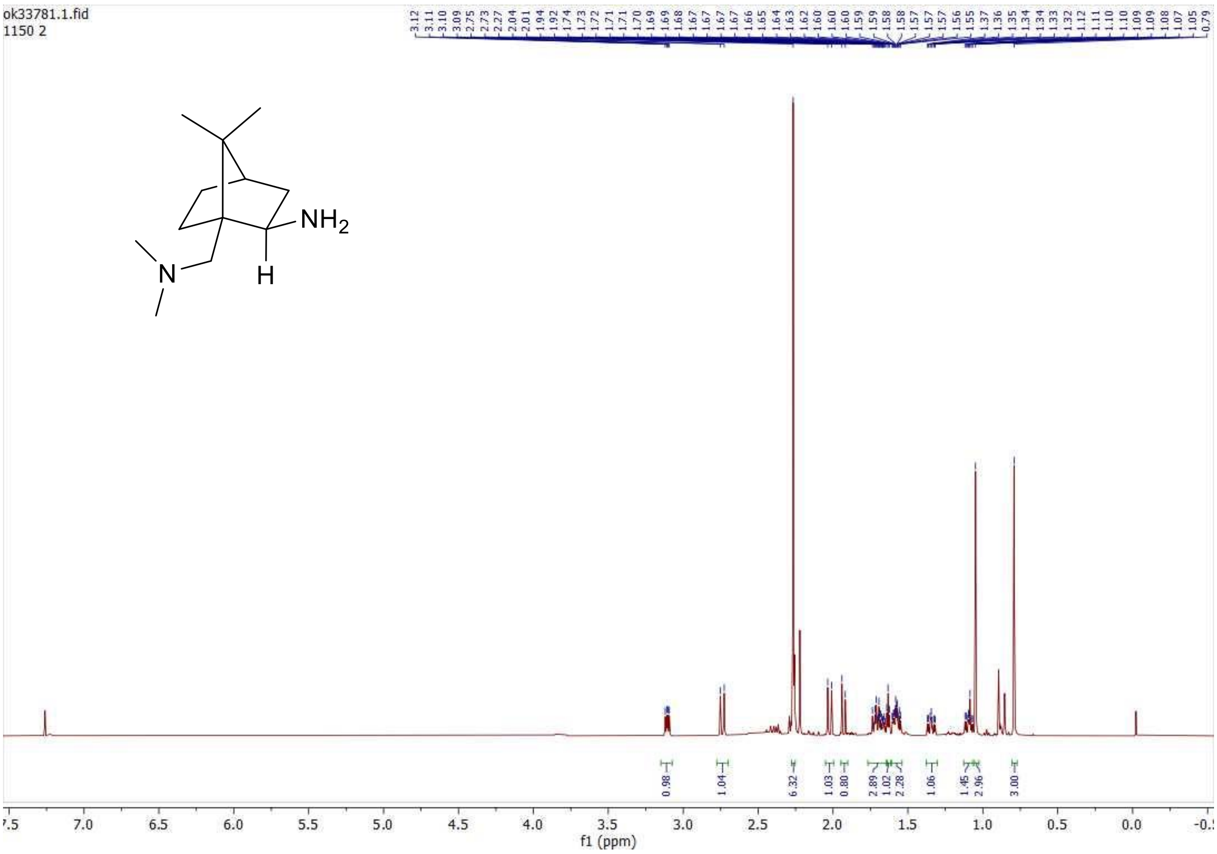

tert-Butyl ((1_S_,2_S_,4_R_)-1-((dimethylamino)methyl)-7,7-dimethylbicyclo[2.2.1]heptan-2-yl)carbamate (3a)

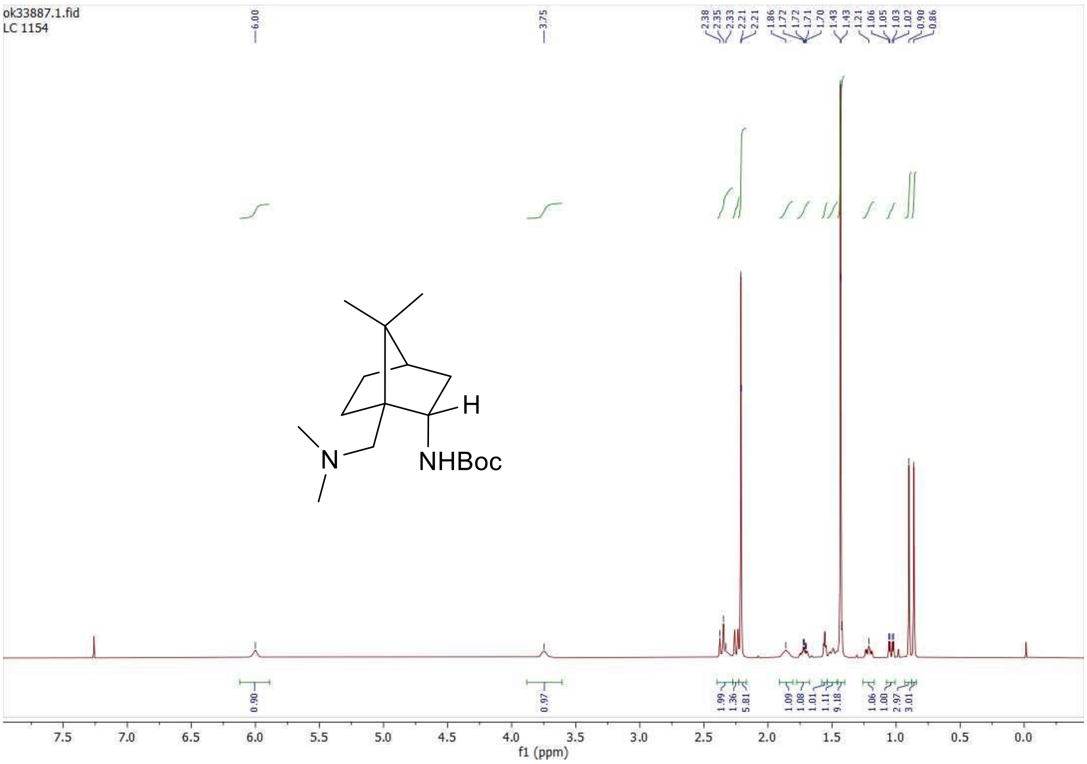

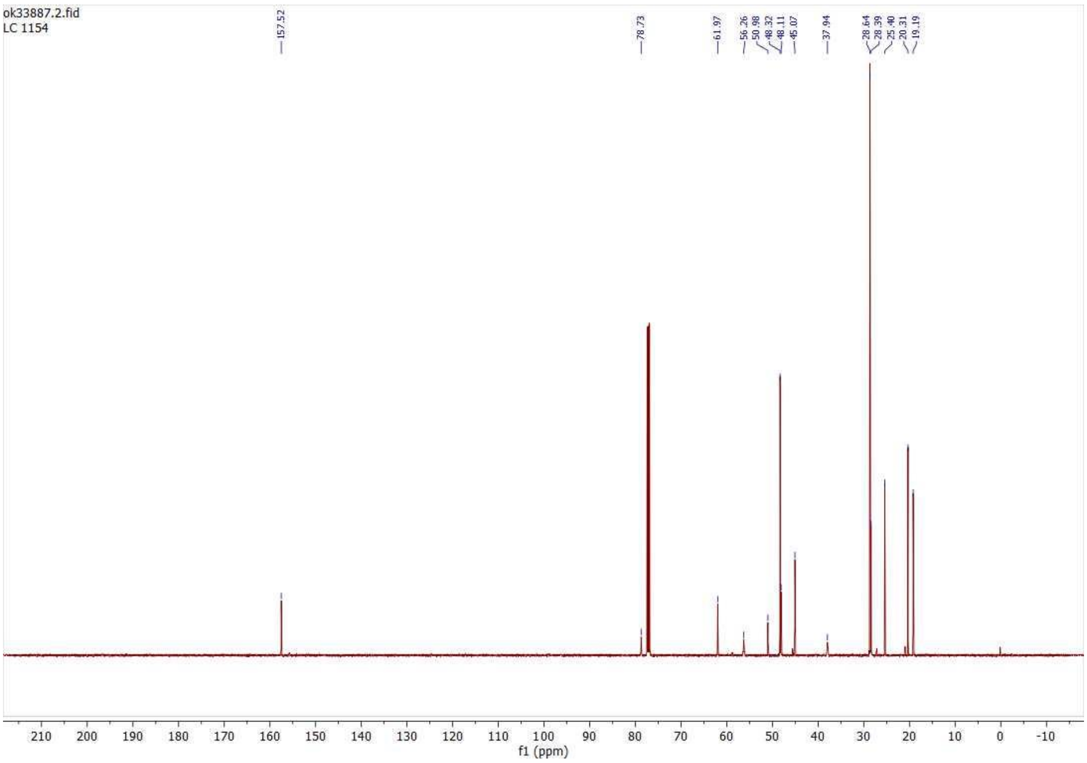

***tert*-Butyl ((1*S*,2*R*,4*R*)-1-((dimethylamino)methyl)-7,7-dimethylbicyclo[2.2.1]heptan-2-yl)carbamate (4a)**

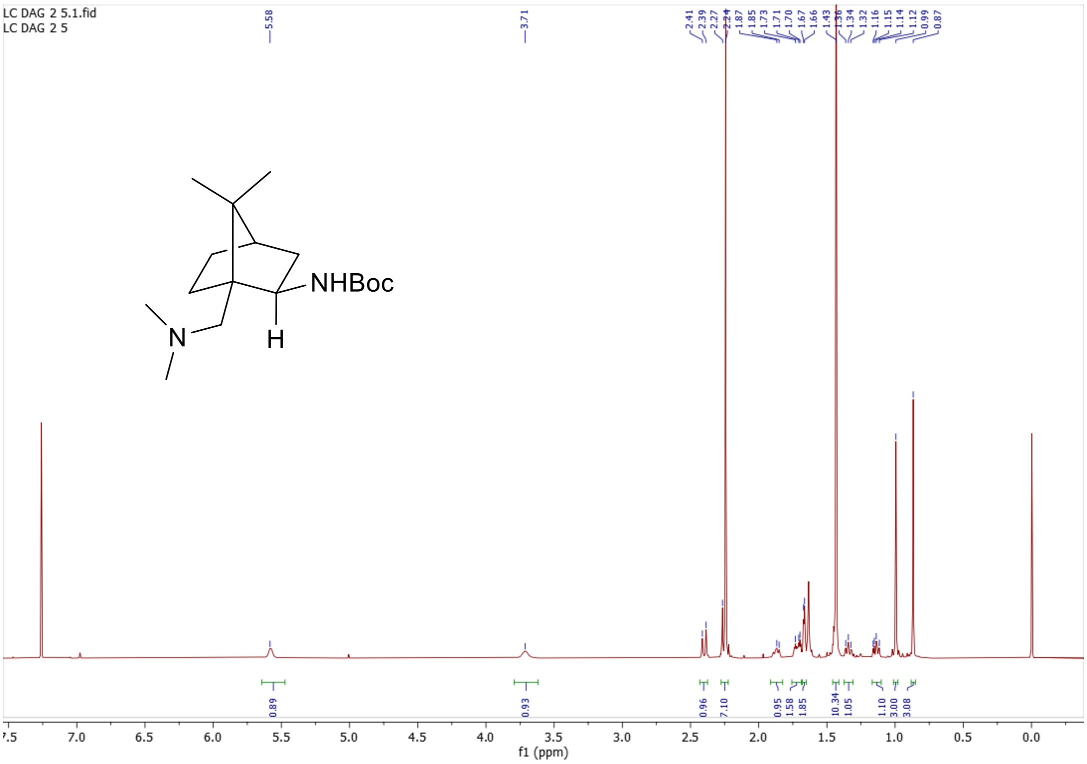

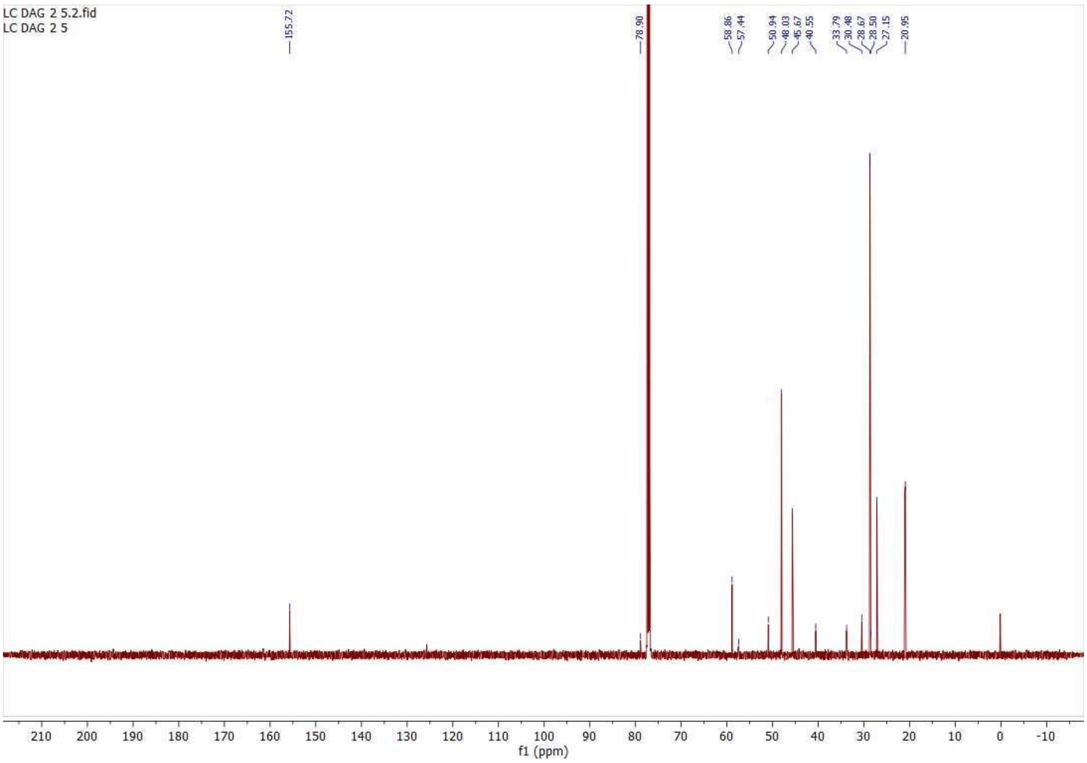

***tert*-Butyl ((1*S*,2*S*,4*R*)-7,7-dimethyl-1-(pyrrolidin-1-ylmethyl)bicyclo[2.2.1]heptan-2-yl)carbamate (3b)**

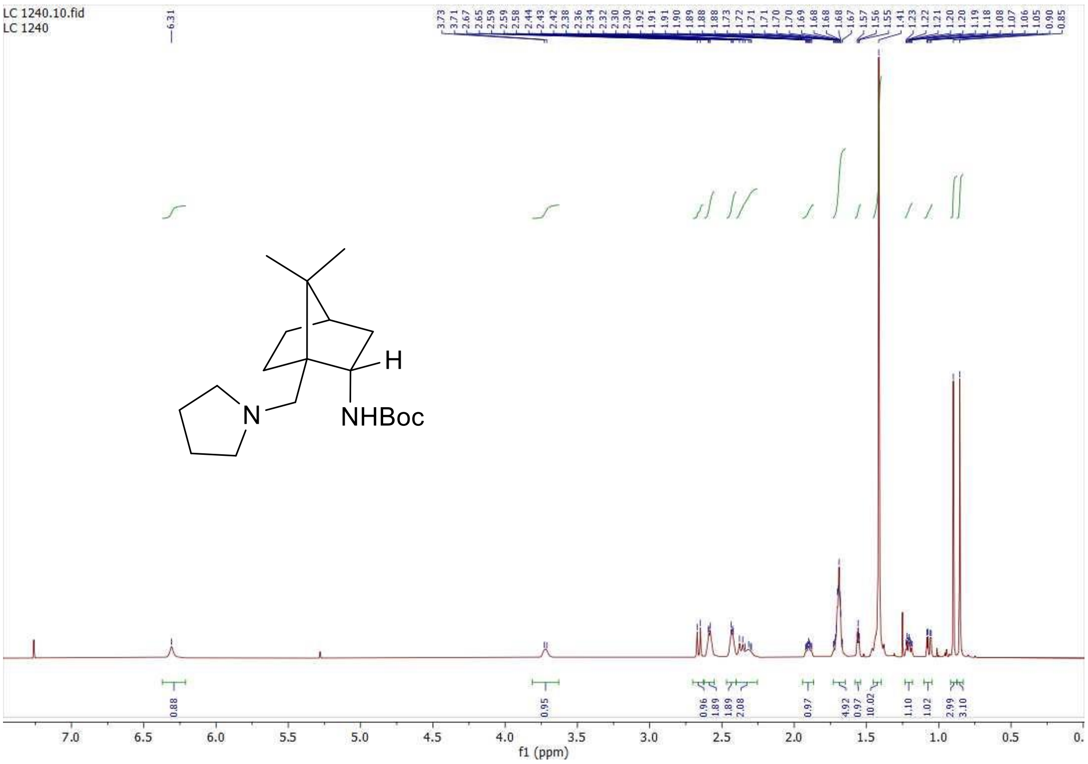

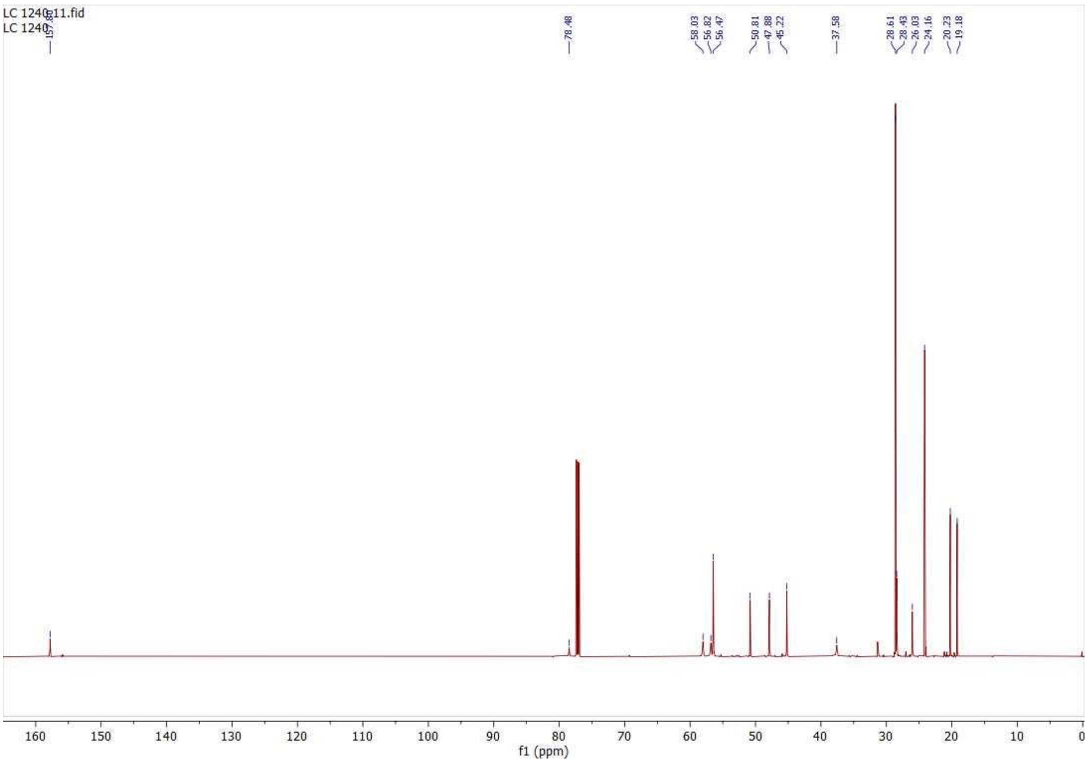

N-Benzyl-1-((1S,2S,4R)-2-((tert-butoxycarbonyl)amino)-7,7-dimethylbicyclo[2.2.1]heptan-1-yl)-N,N-dimethylmethanaminium bromide (5a)

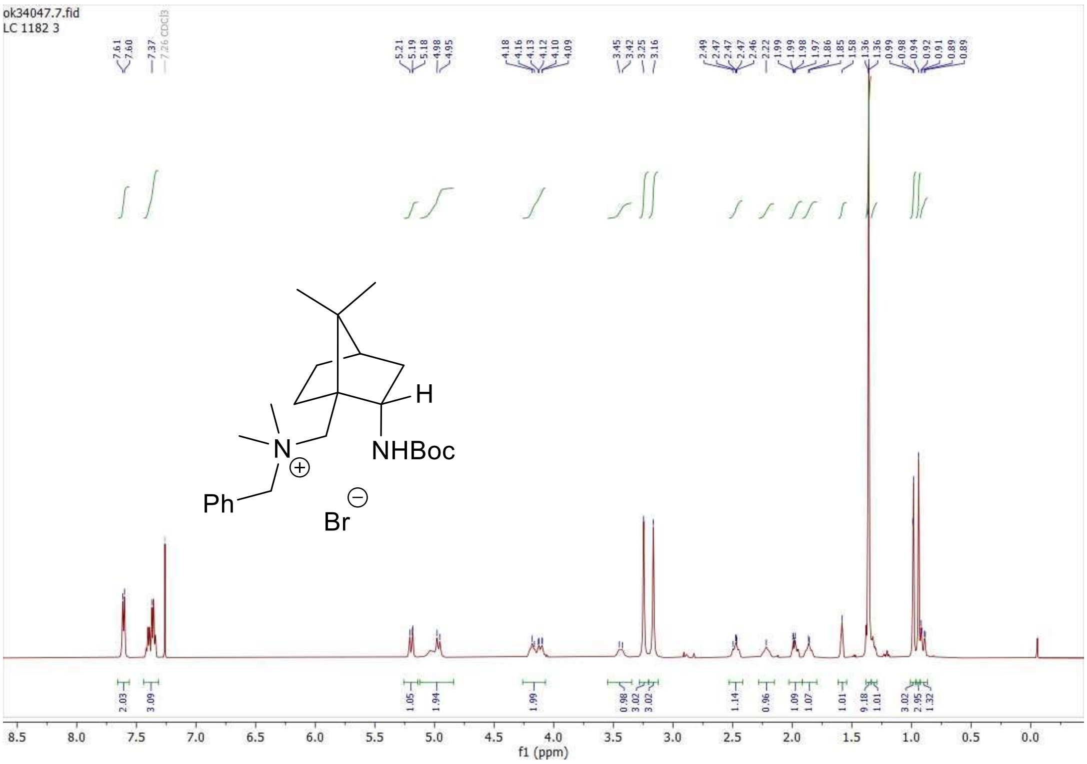

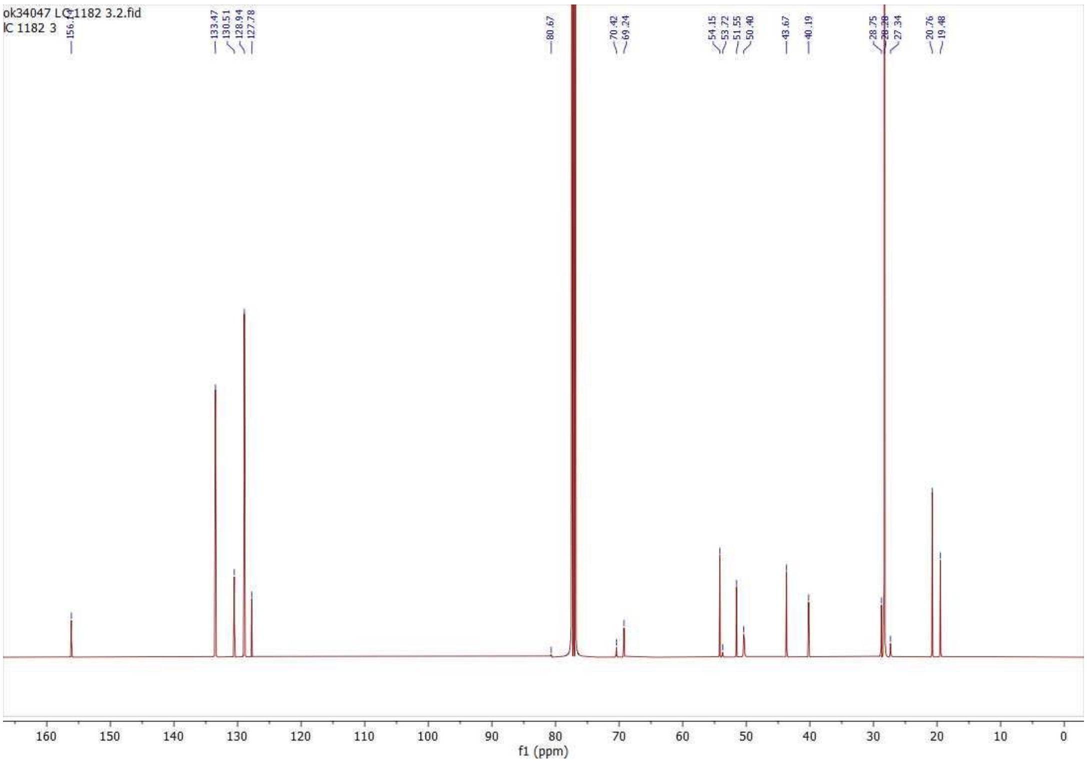

_N_-Benzyl-1-((1_S_,2_R_,4_R_)-2-((_tert_-butoxycarbonyl)amino)-7,7-dimethylbicyclo[2.2.1]heptan-1-yl)-_N_,_N_-dimethylmethanaminium bromide (6a)

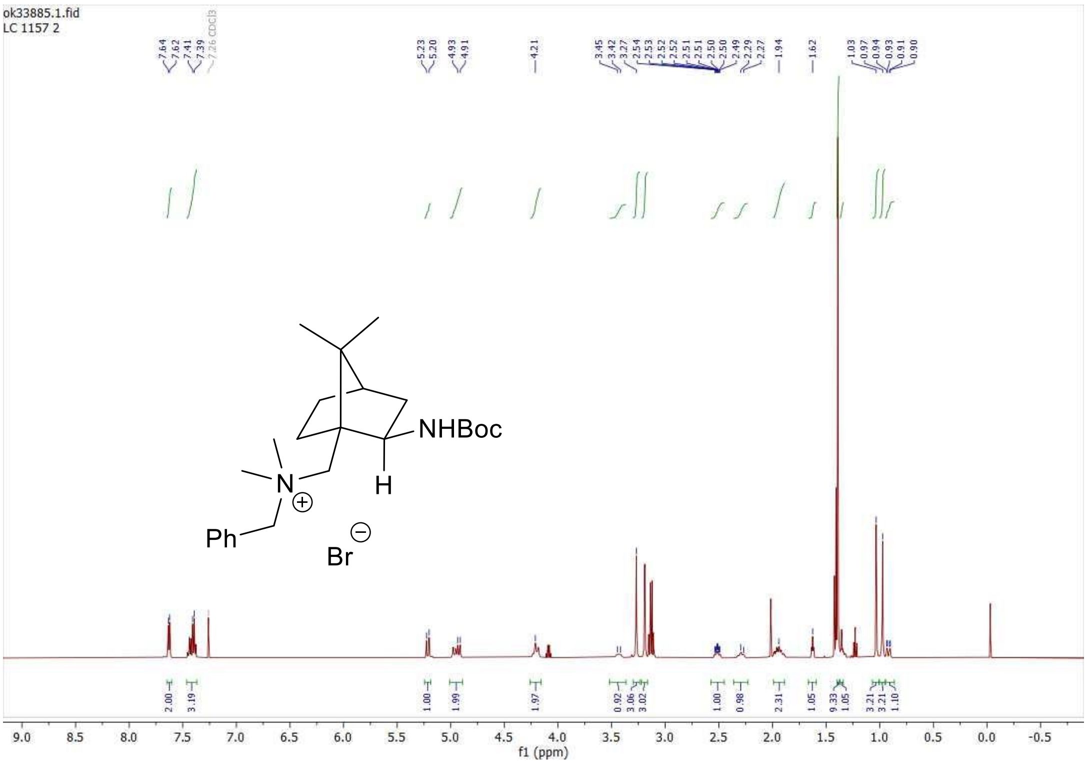

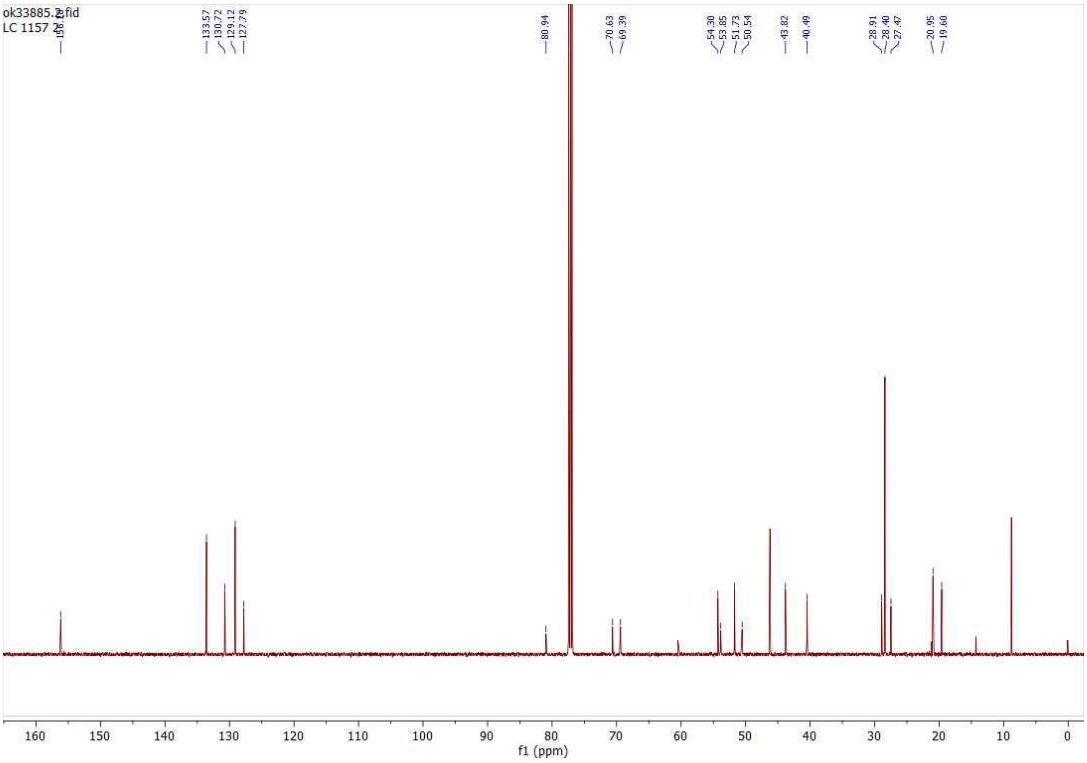

1-Benzyl-1-(((1*S*,2*S*,4*R*)-2-((*tert*-butoxycarbonyl)amino)-7,7-dimethylbicyclo[2.2.1]heptan-1-yl)methyl)pyrrolidin-1-ium bromide (5b)

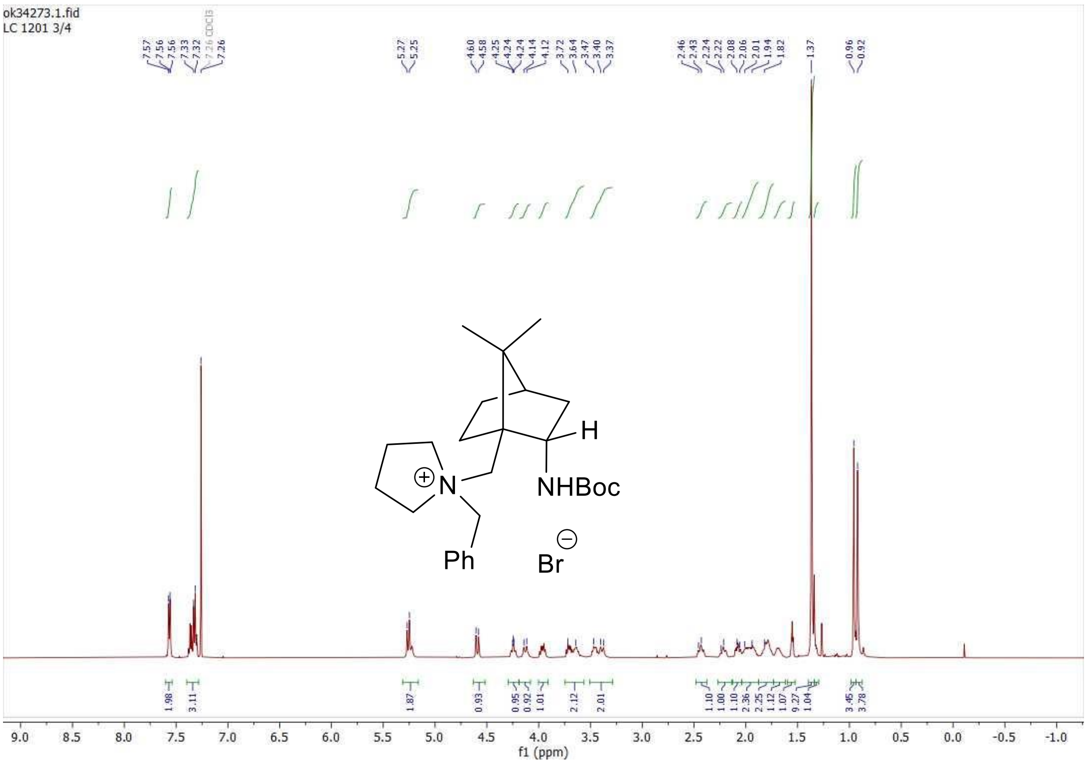

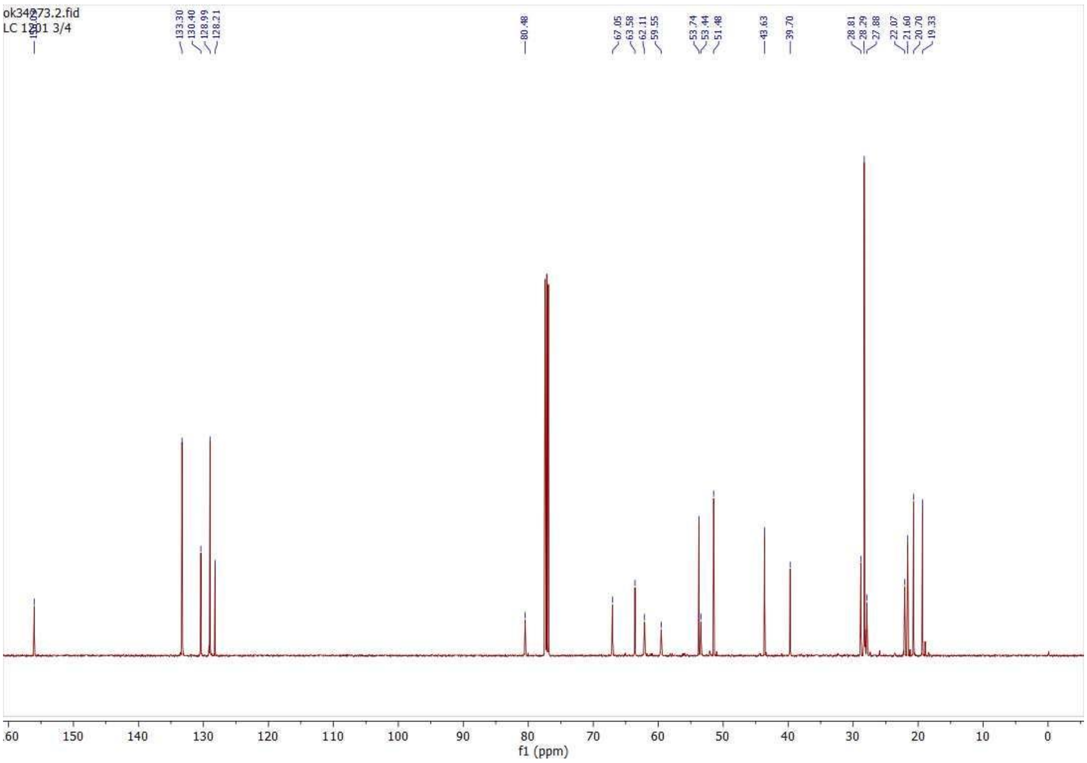

(1*S*,2*S*,4*R*)-1-((Benzyldimethylammonio)methyl)-7,7-dimethylbicyclo[2.2.1]heptan-2-aminium 2,2,2-trifluoroacetate (7a)

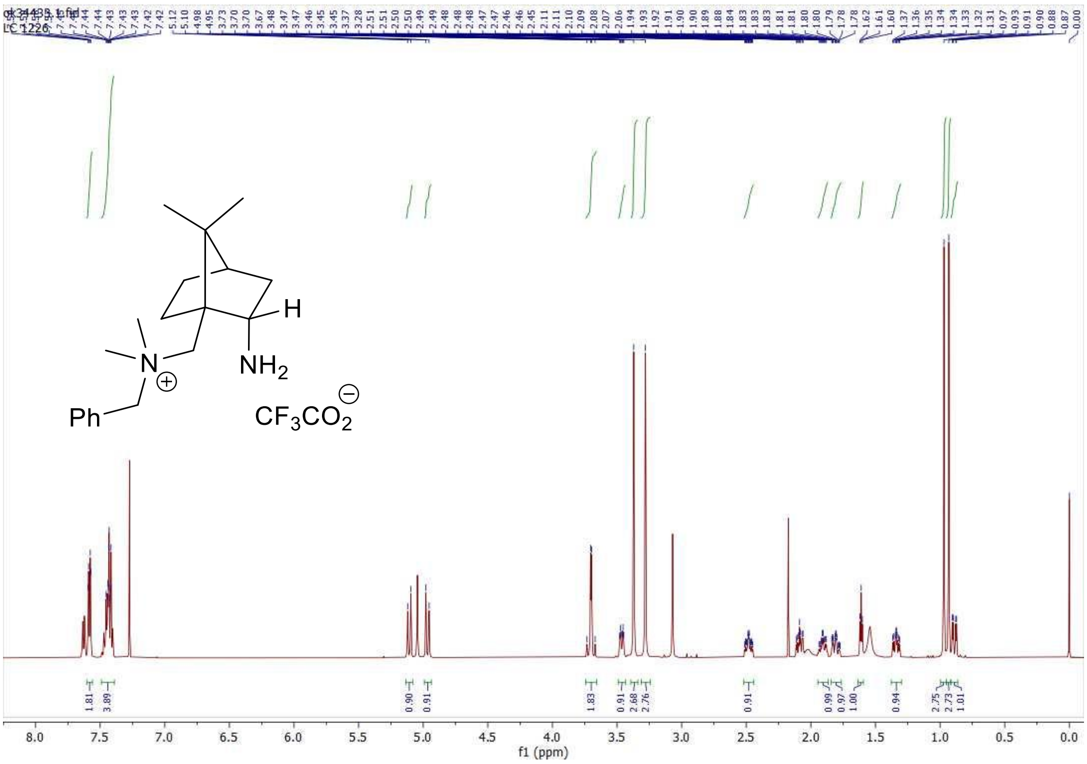

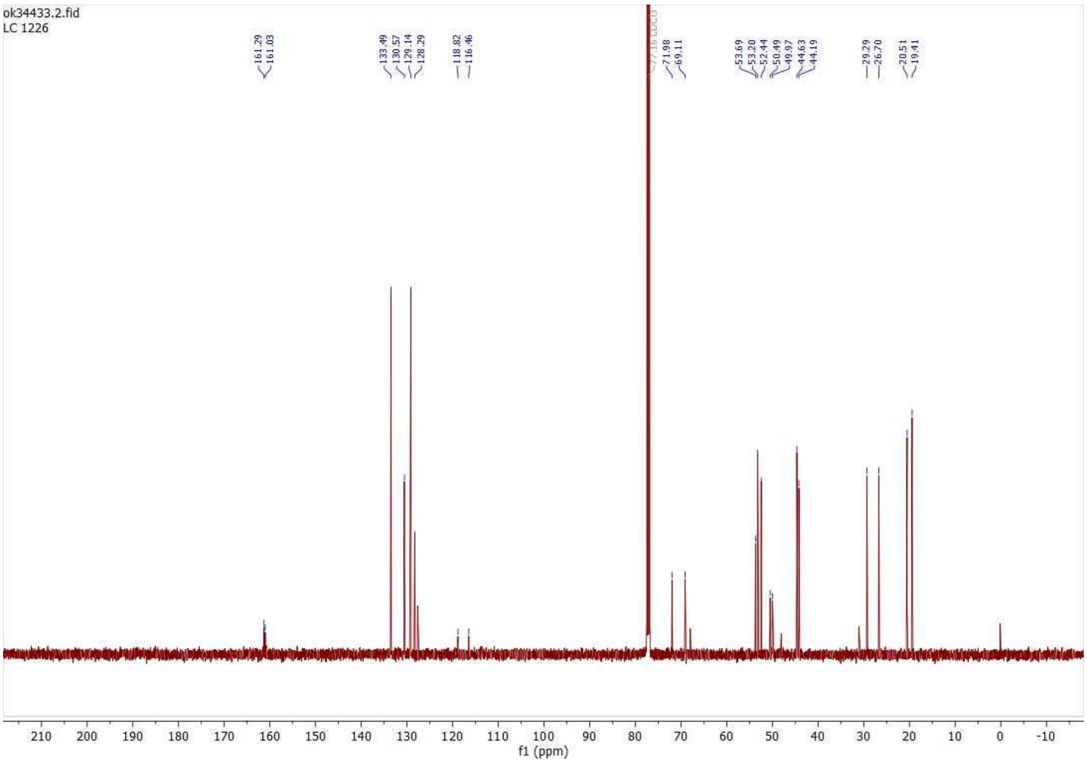

(1*S*,2*R*,4*R*)-1-((Benzyldimethylammonio)methyl)-7,7-dimethylbicyclo[2.2.1]heptan-2-aminium 2,2,2-trifluoroacetate (8a)

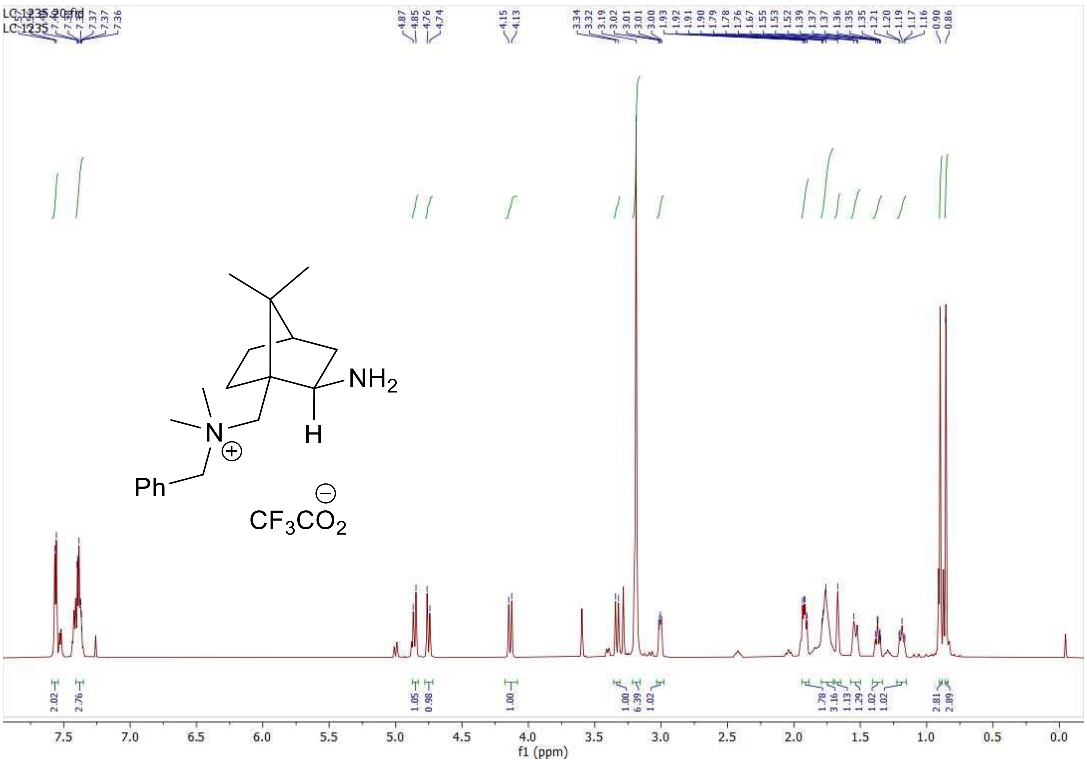

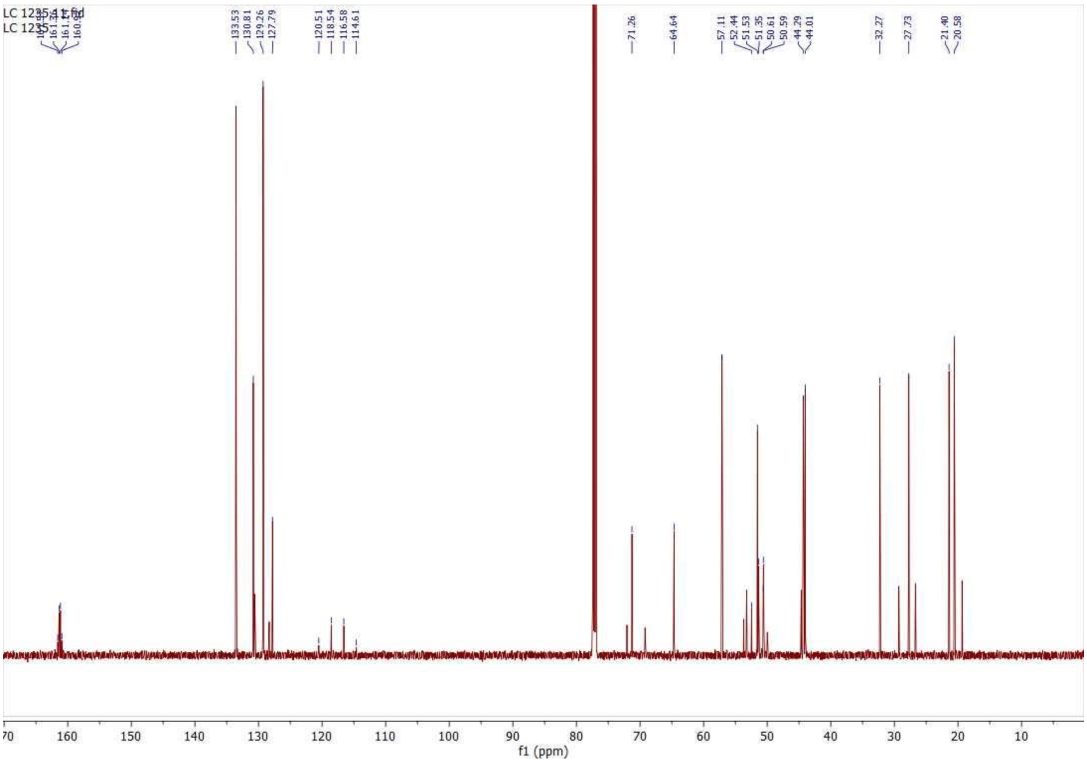

1-(((1*S*,2*S*,4*R*)-2-Ammonio-7,7-dimethylbicyclo[2.2.1]heptan-1-yl)methyl)-1-benzylpyrrolidin-1-ium iodide (7b)

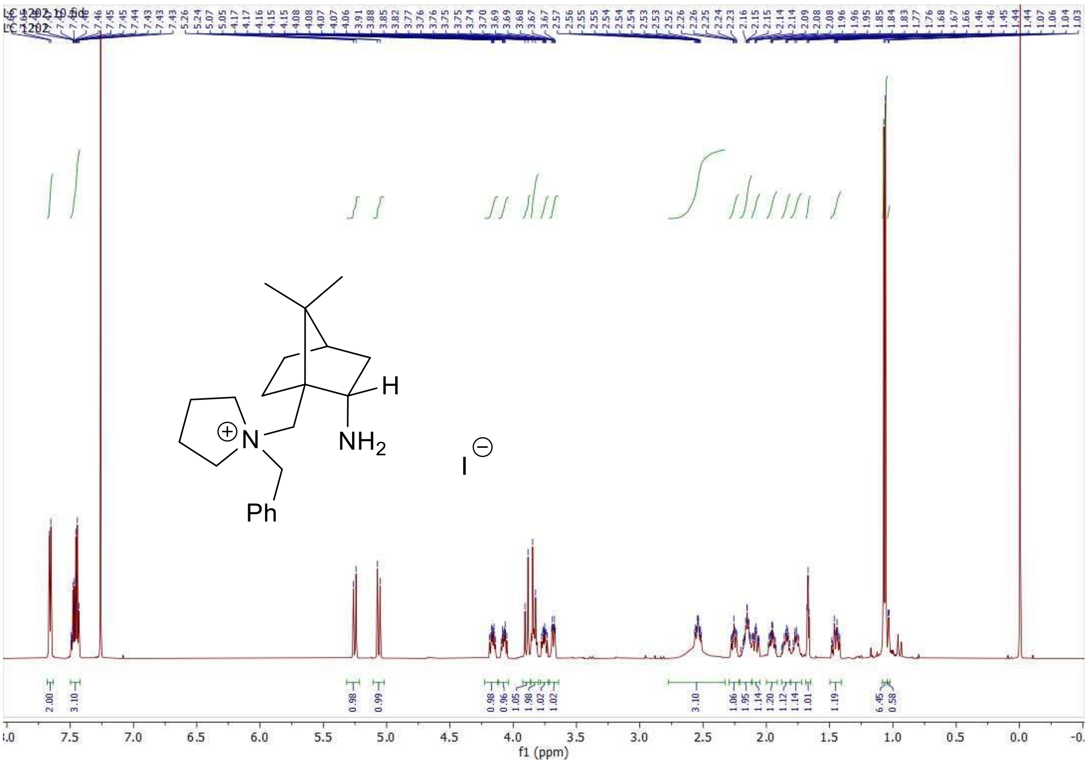

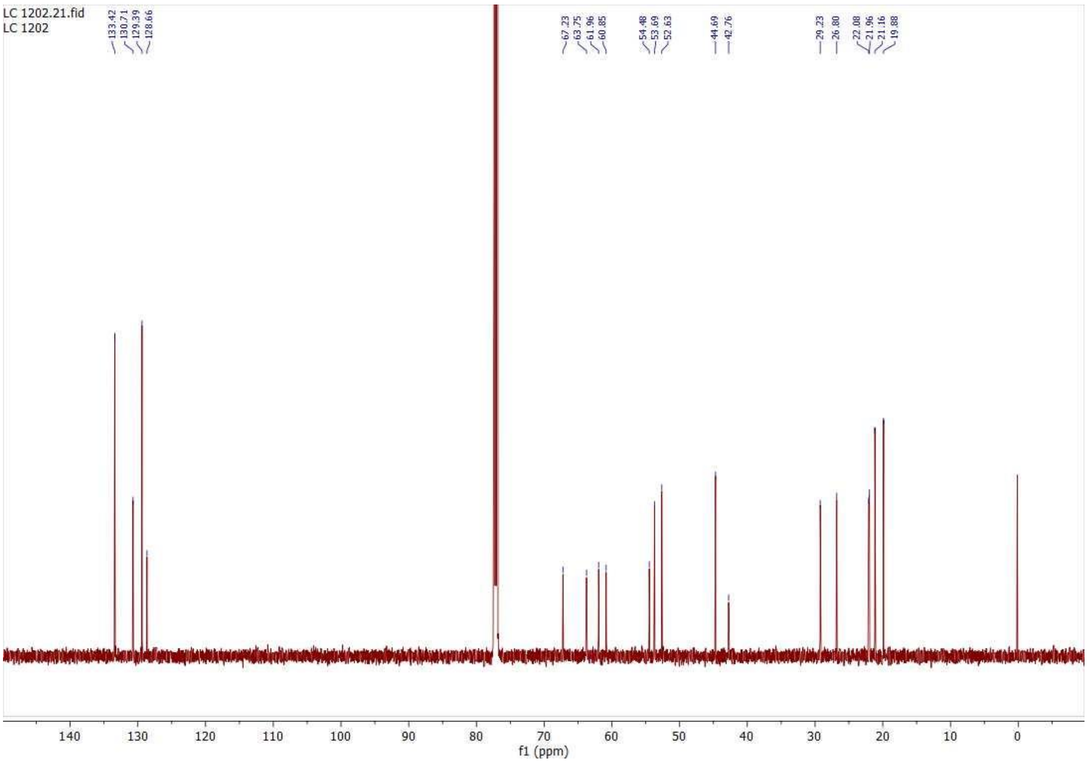

***N*-Benzyl-1-((1*R*,2*R*,4*R*)-2-(3-(3,5-bis(trifluoromethyl)phenyl)thioureido)-7,7-dimethylbicyclo[2.2.1]heptan-1-yl)-*N*,*N*-dimethylmethanaminium 2,2,2-trifluoroacetate (I)**

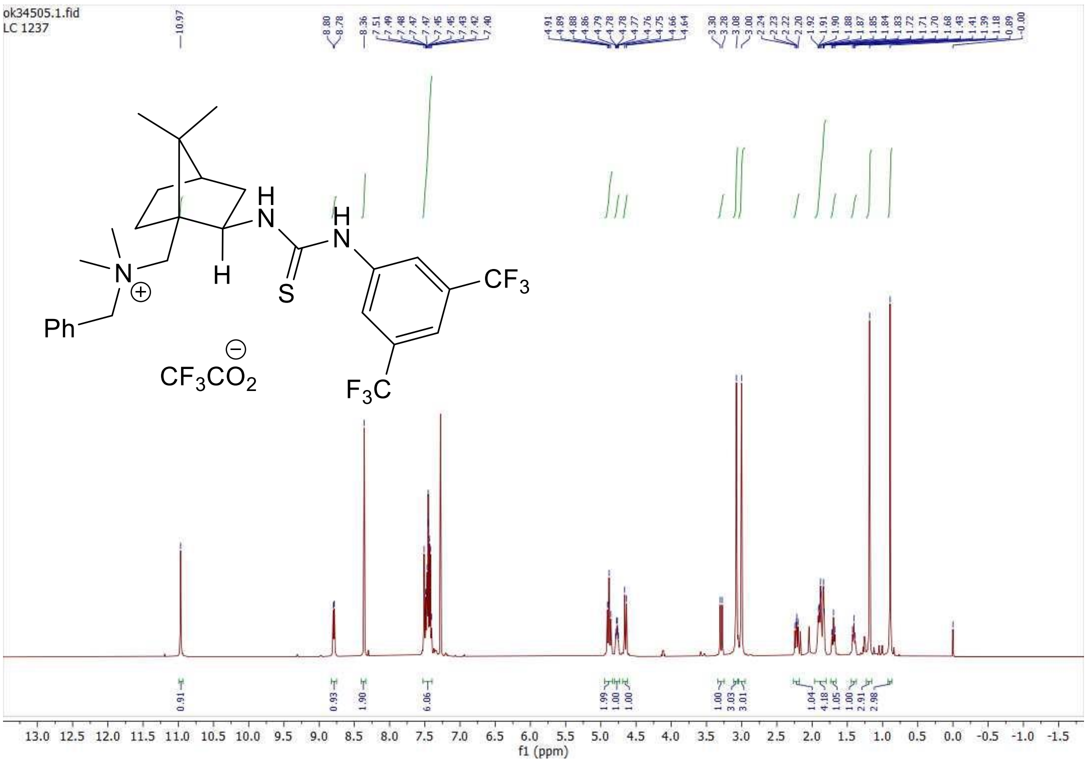

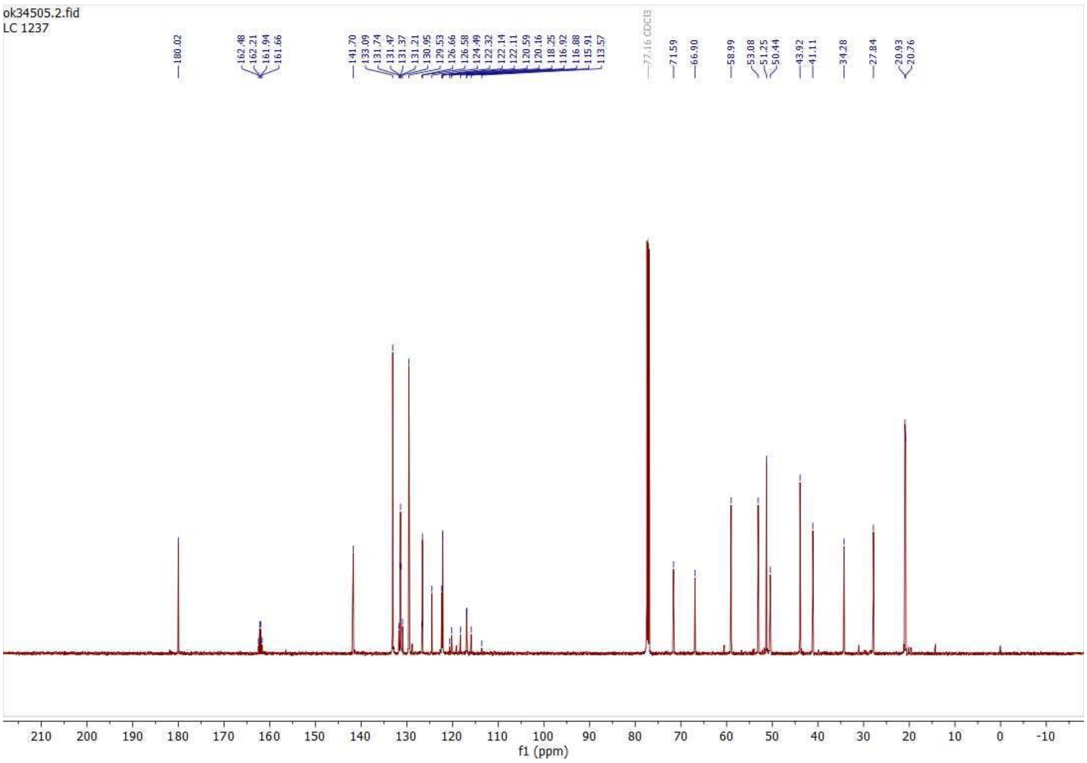

NOESY

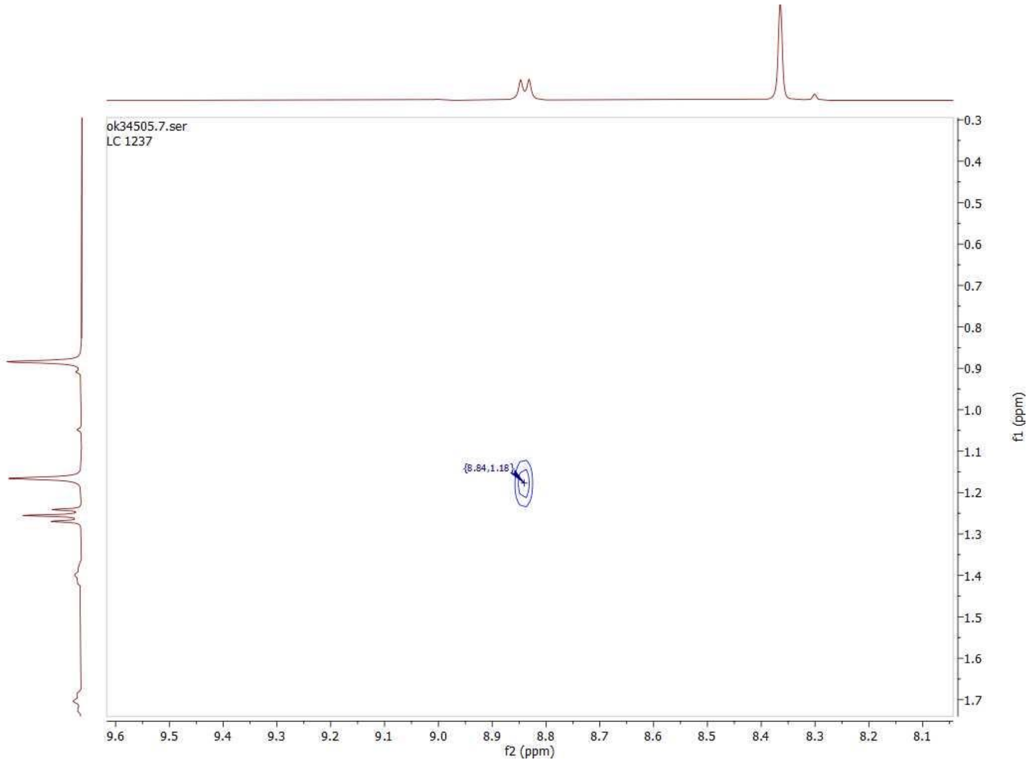

^{19}F-NMR

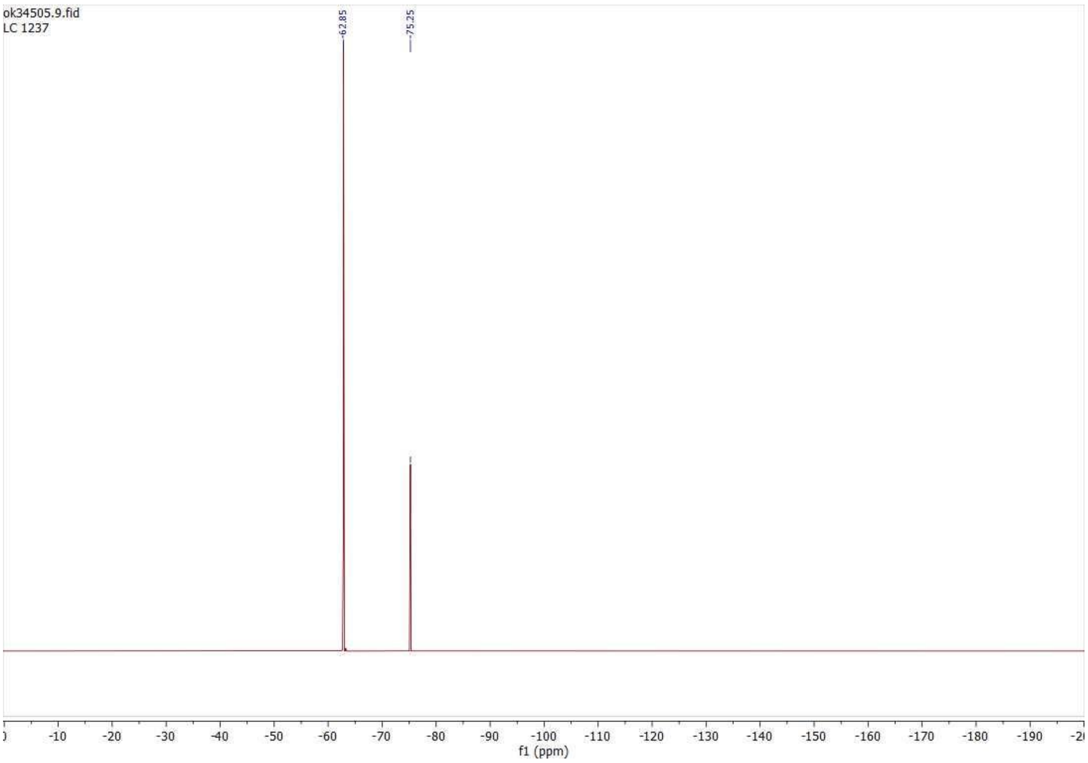

N-Benzyl-1-((1R,2R,4R)-2-(3-(3,5-bis(trifluoromethyl)phenyl)thioureido)-7,7-dimethylbicyclo[2.2.1]heptan-1-yl)-N,N-dimethylmethanaminium iodide (II)

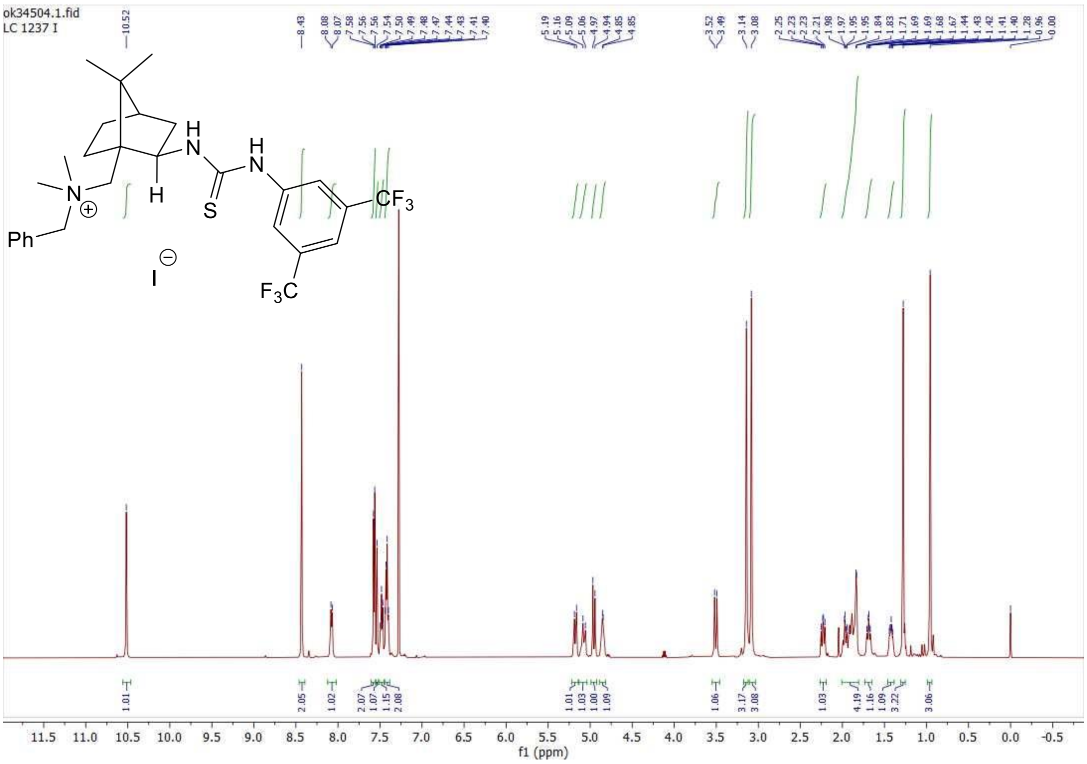

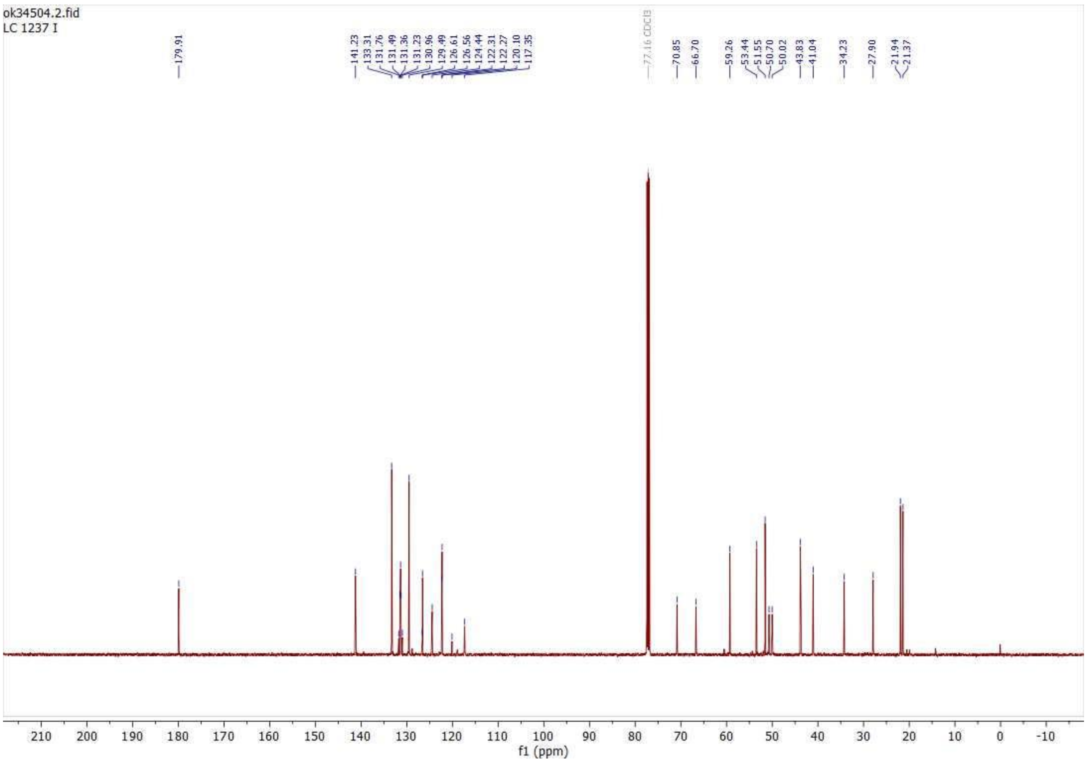

NOESY

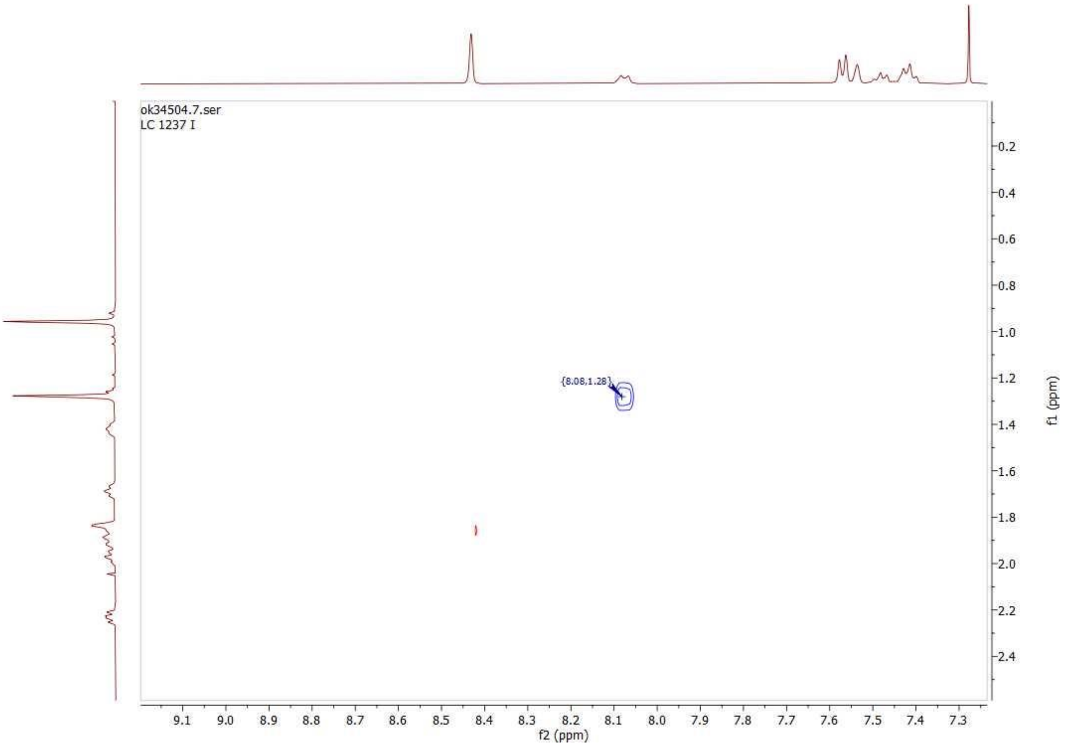

^{19}F-NMR

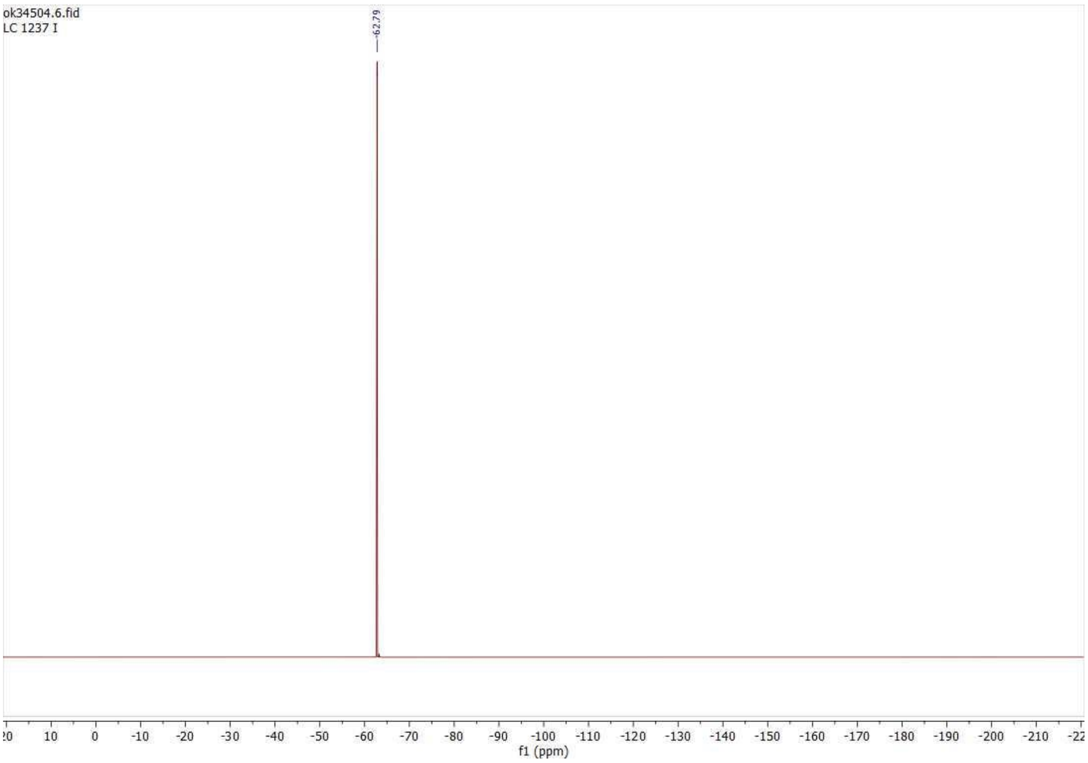

1-Benzyl-1-(((1*S*,2*S*,4*R*)-2-(3-(3,5-bis(trifluoromethyl)phenyl)thioureido)-7,7-dimethylbicyclo[2.2.1]heptan-1-yl)methyl)pyrrolidin-1-ium iodide (III)

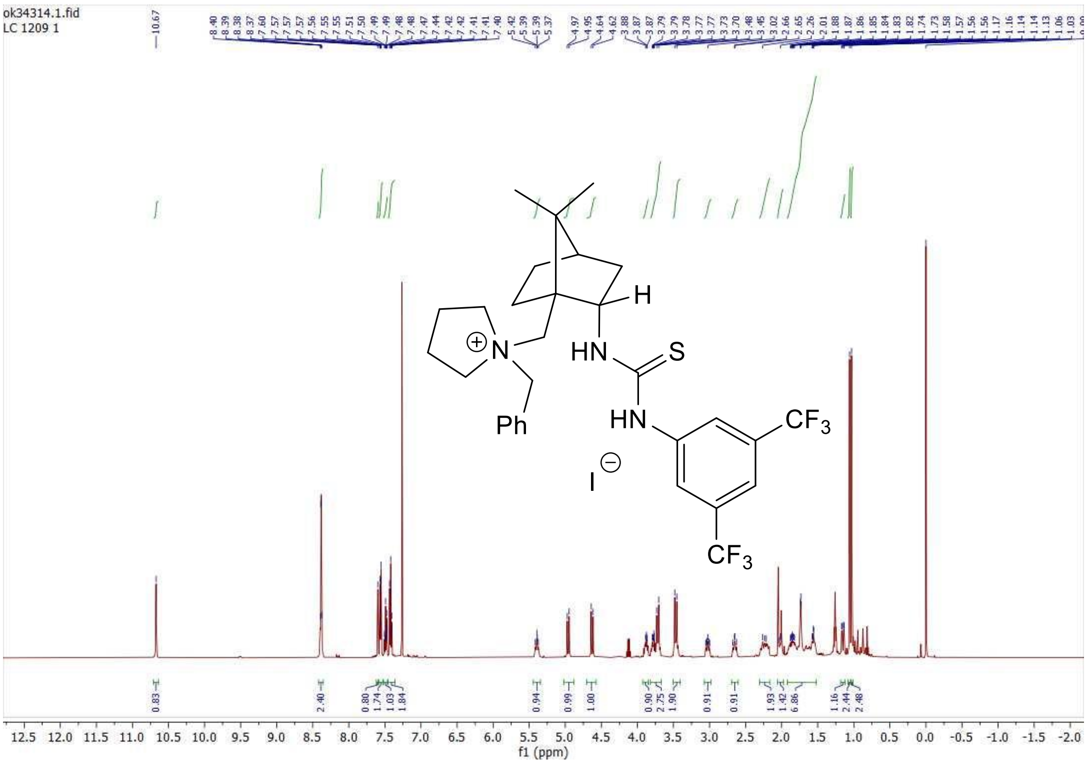

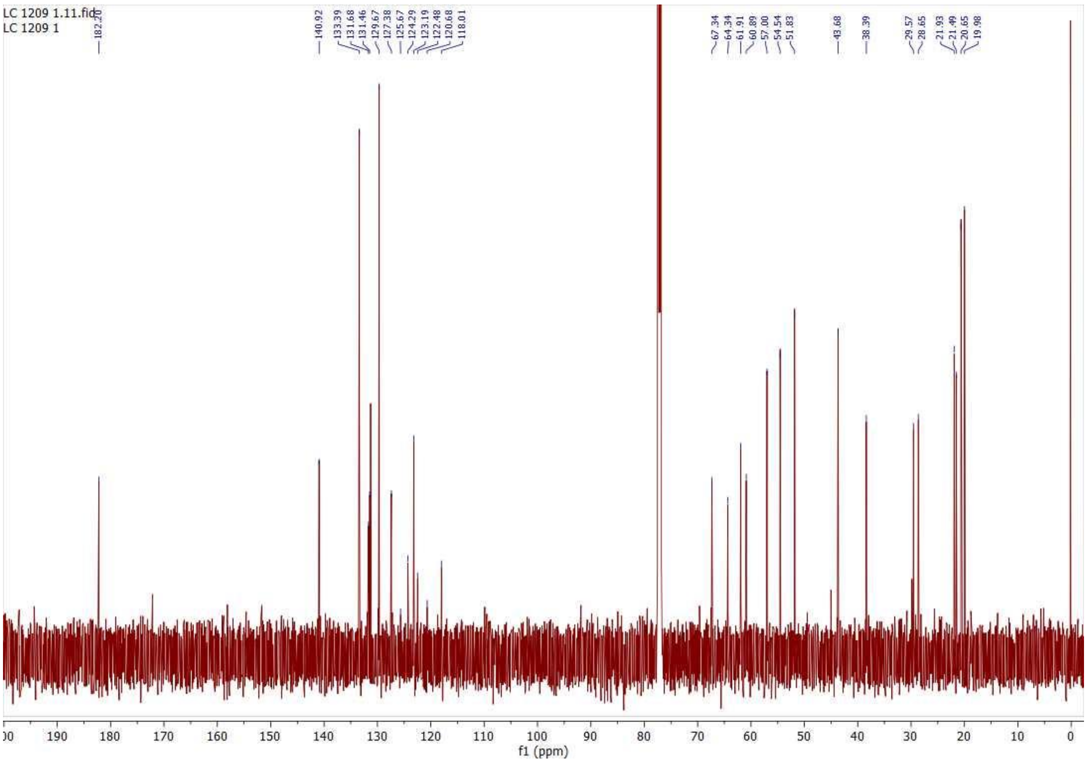

NOESY

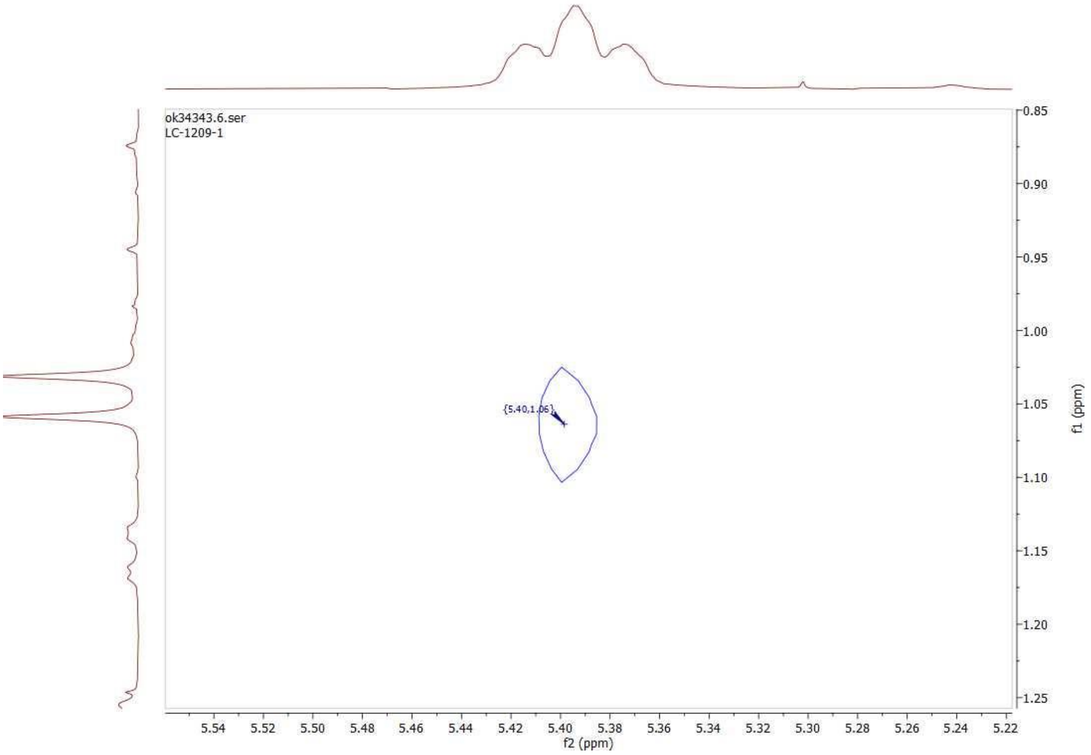

^{19}F-NMR

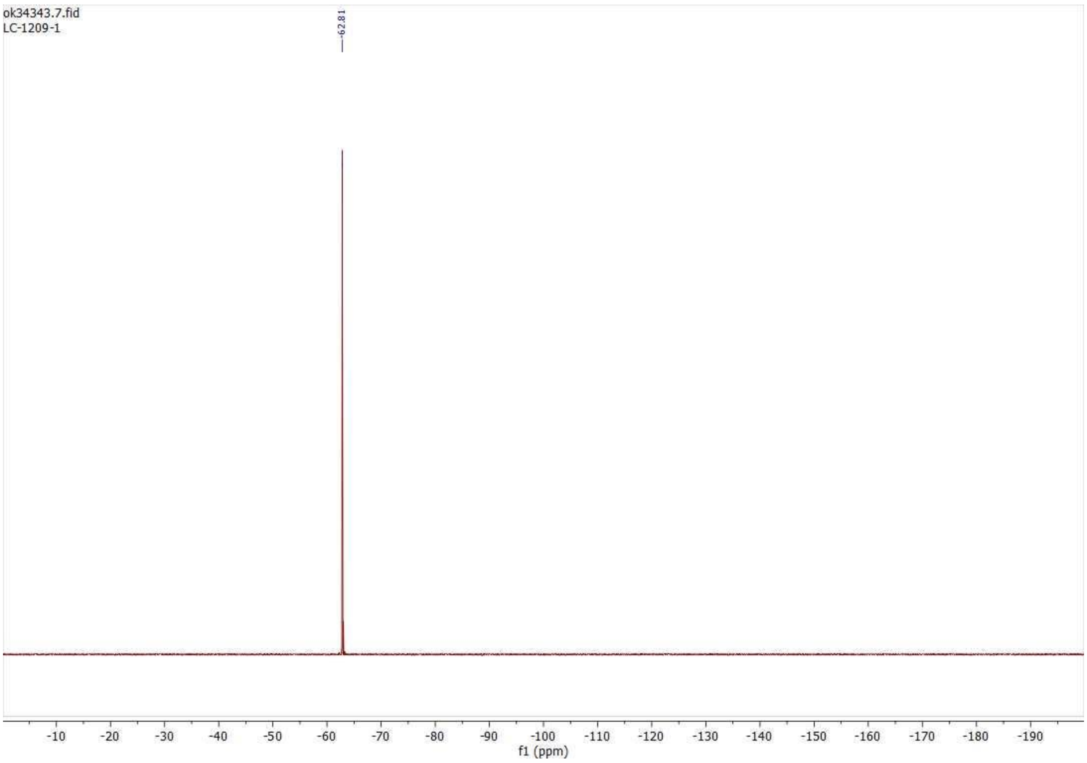

***N*-Benzyl-1-((1*R*,2*S*,4*R*)-2-(3-(3,5-bis(trifluoromethyl)phenyl)thioureido)-7,7-dimethylbicyclo[2.2.1]heptan-1-yl)-*N*,*N*-dimethylmethanaminium 2,2,2-trifluoroacetate (IV)**

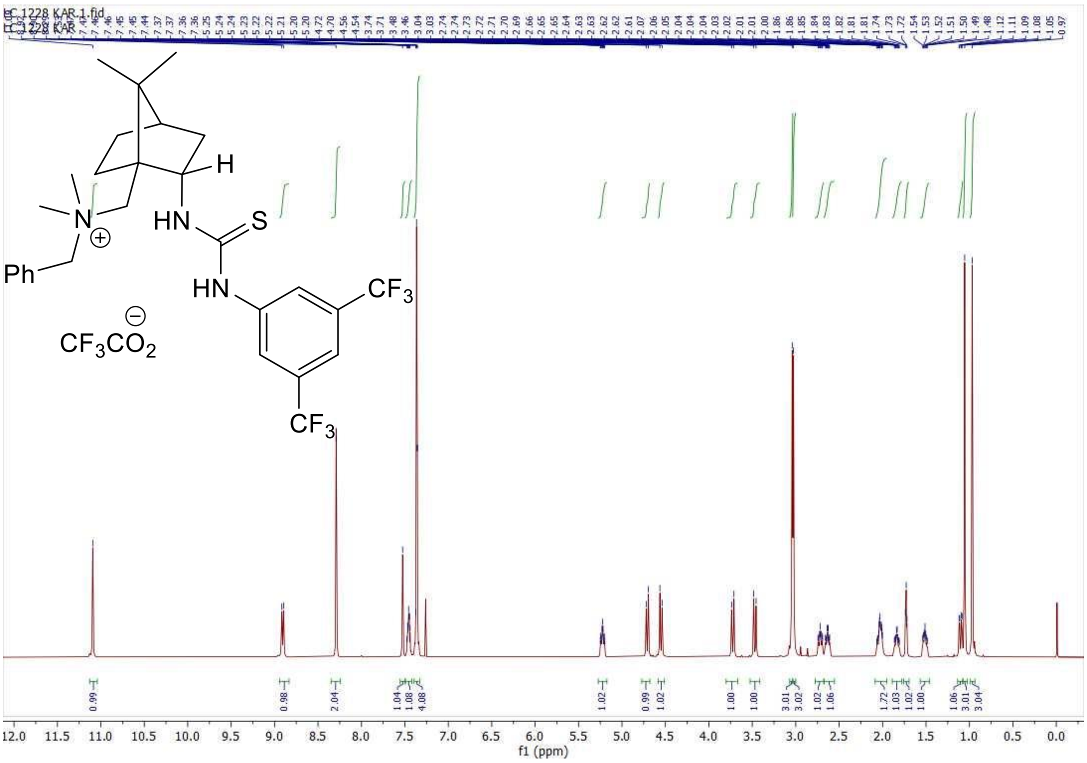

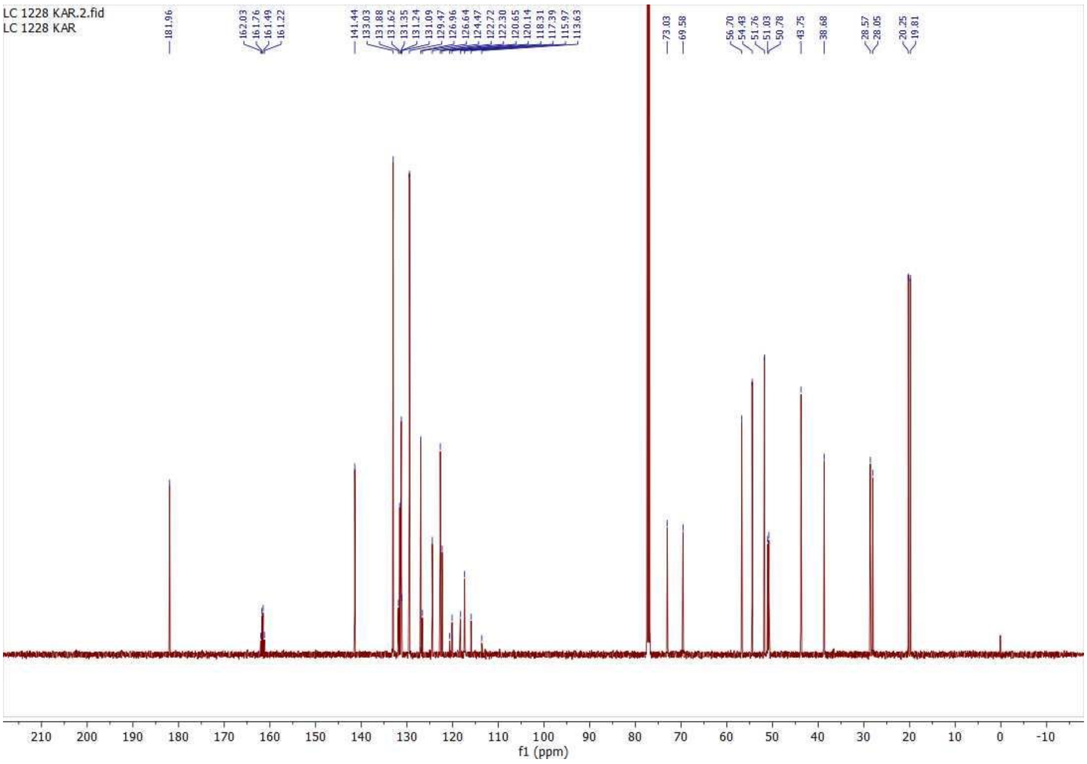

NOESY

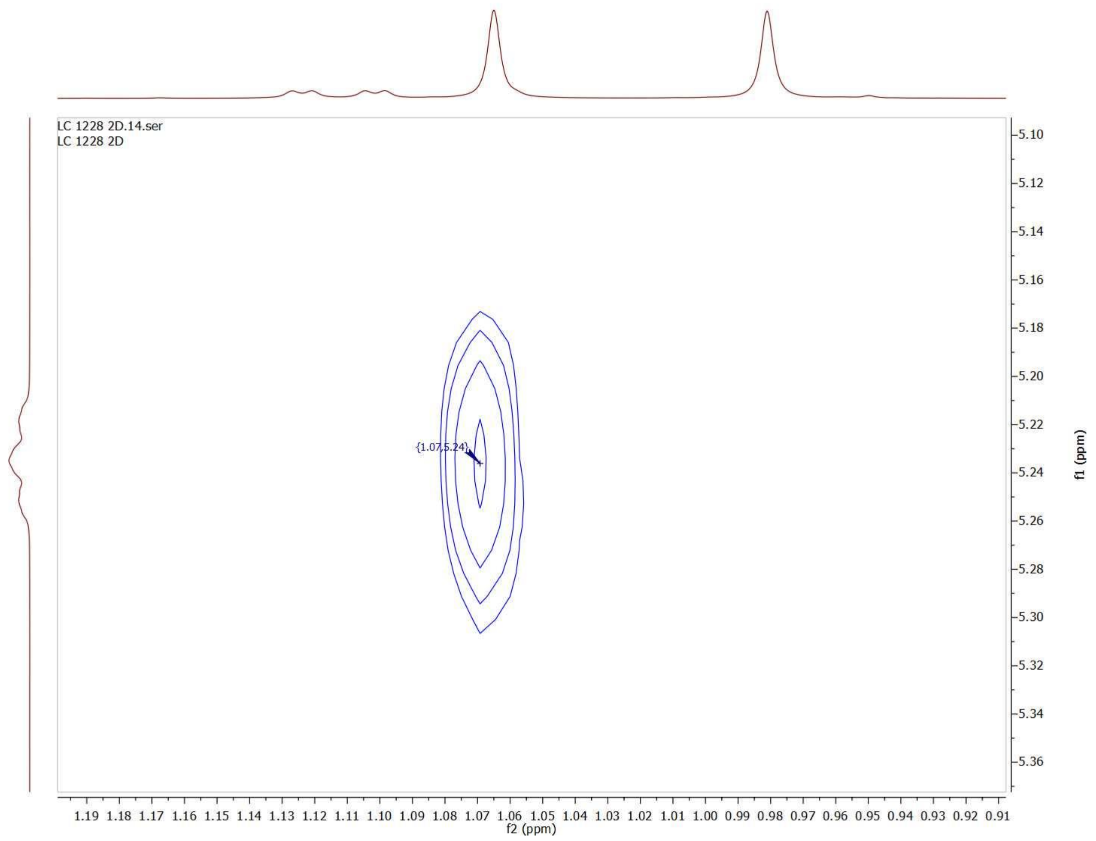

^{19}F-NMR

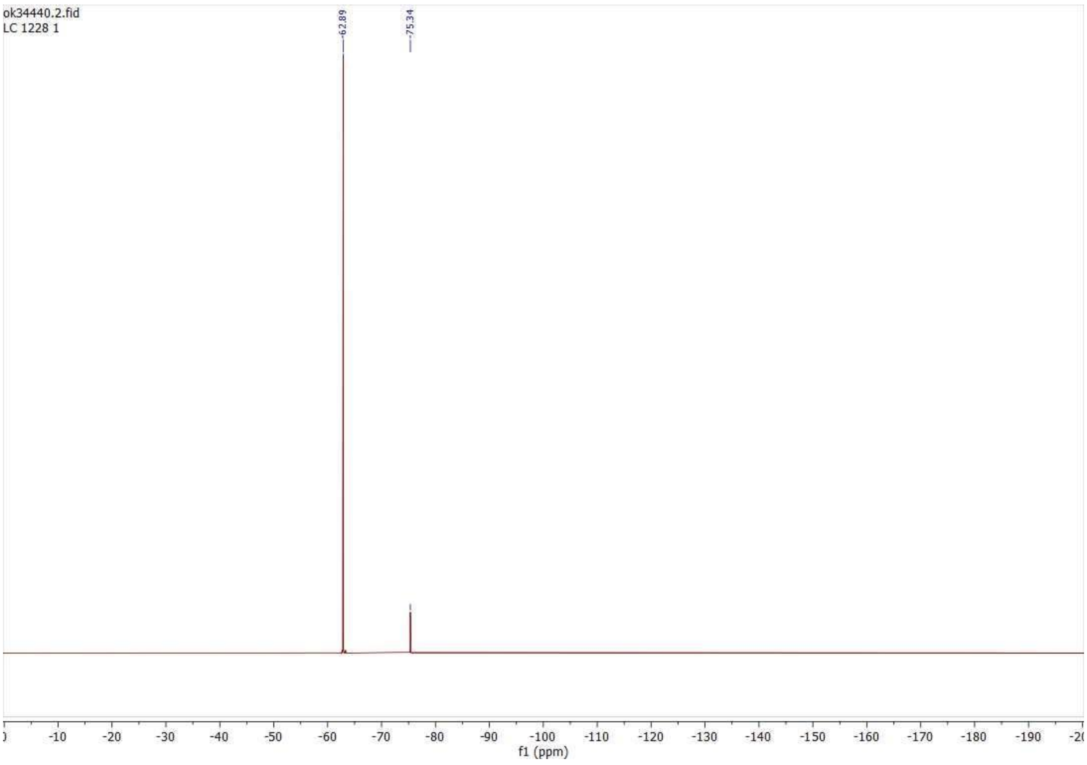

***N*-Benzyl-1-((1*R*,2*S*,4*R*)-2-(3-(3,5-bis(trifluoromethyl)phenyl)thioureido)-7,7-dimethylbicyclo[2.2.1]heptan-1-yl)-*N*,*N*-dimethylmethanaminium iodide (V)**

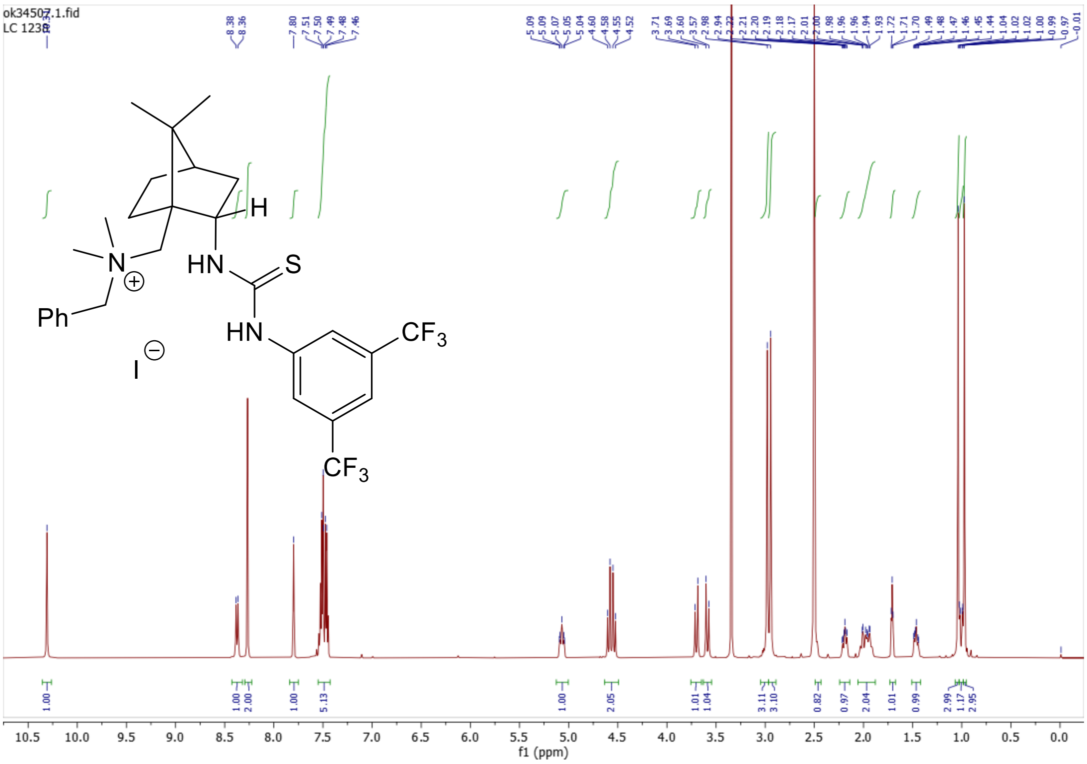

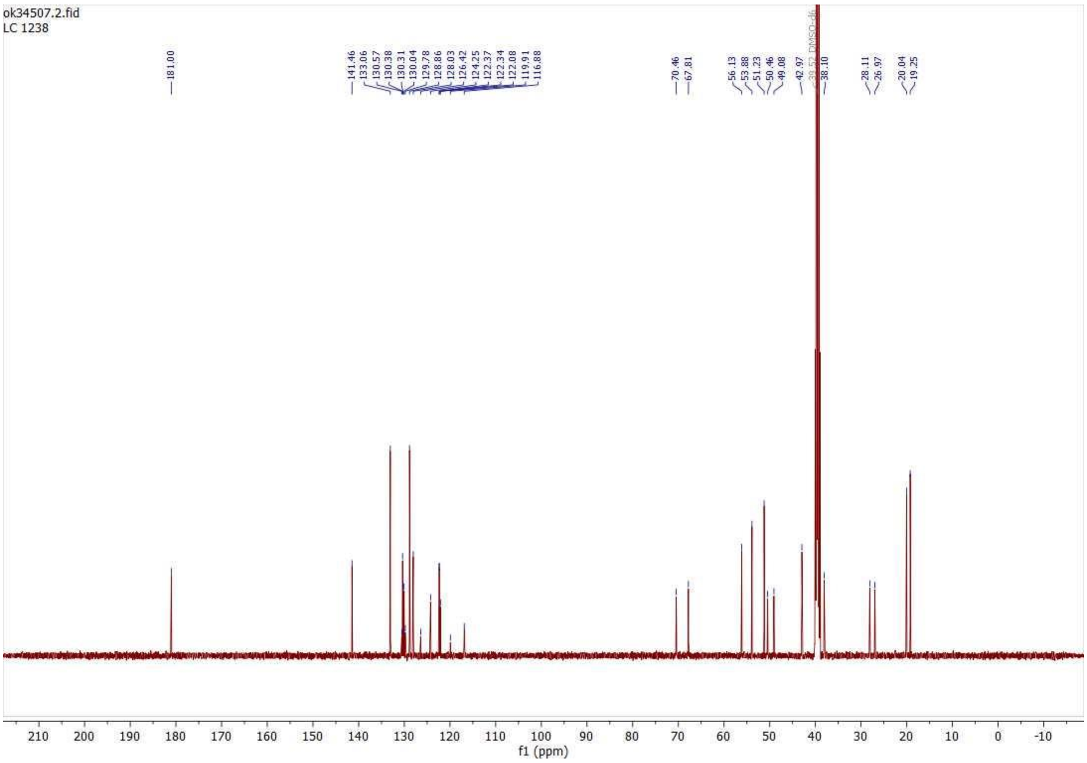

NOESY

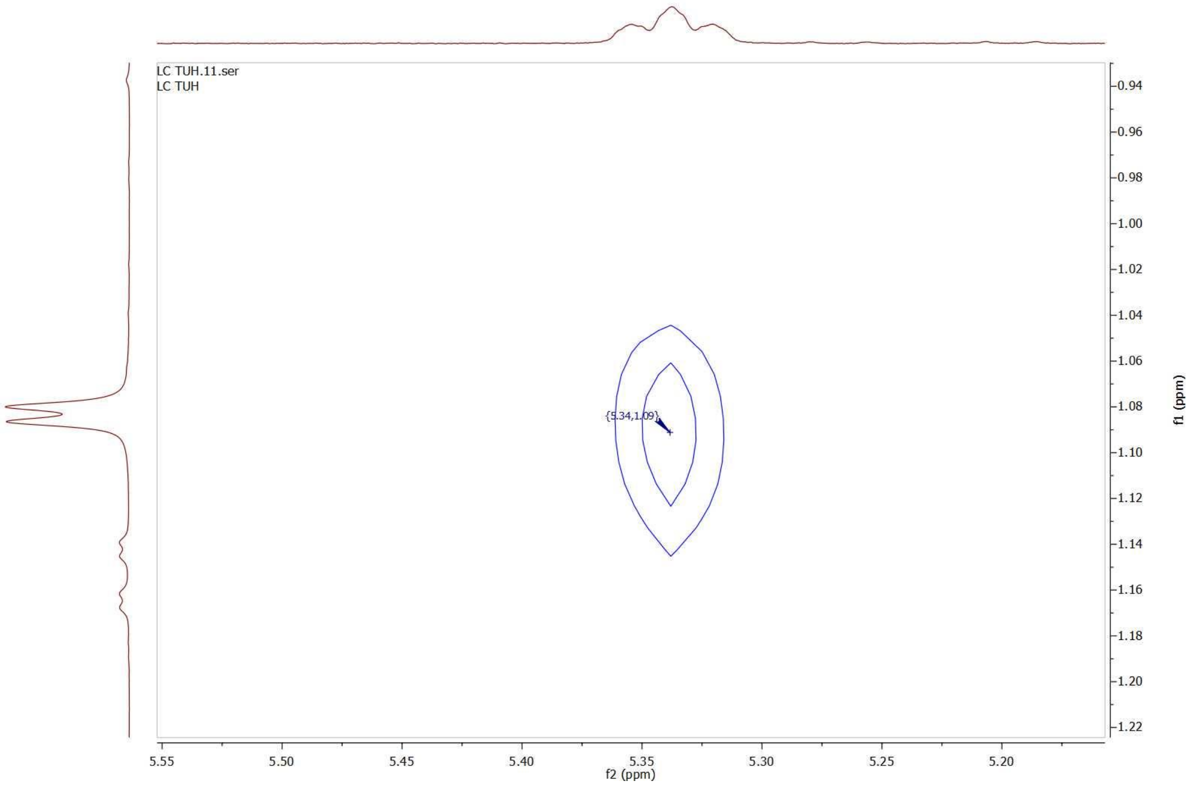

^{19}F-NMR

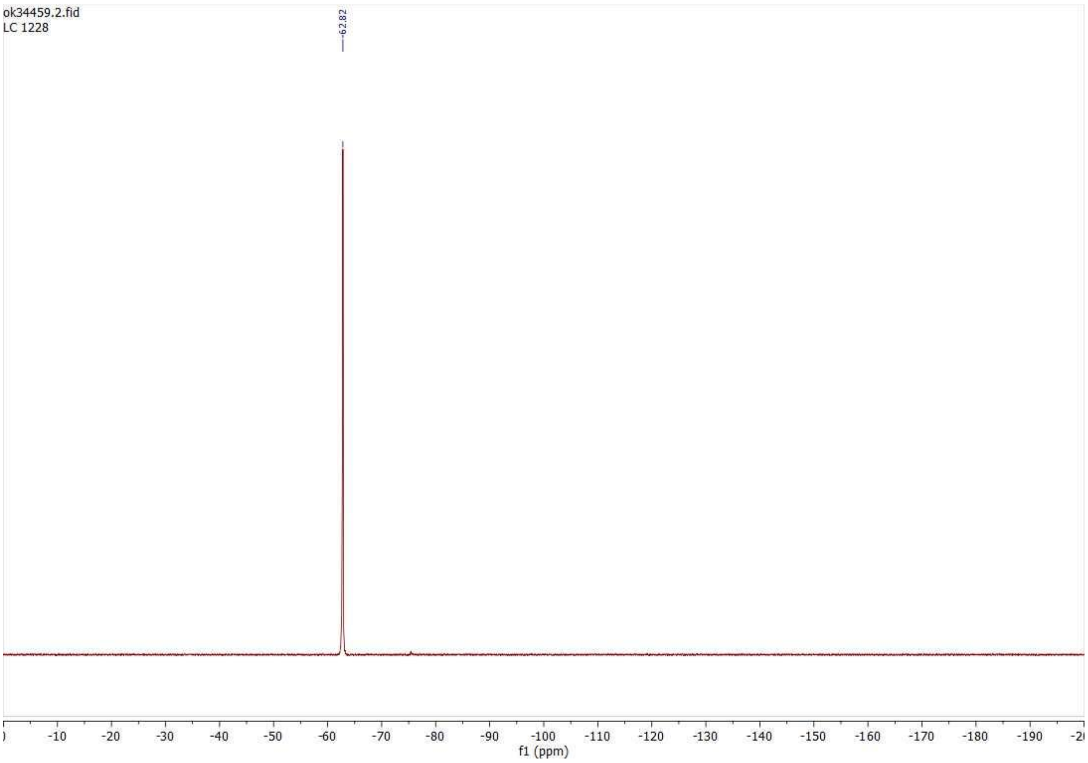

***N*-Benzyl-1-((1*R*,2*S*,4*R*)-7,7-dimethyl-2-(3-phenylthioureido)bicyclo[2.2.1]heptan-1-yl)-*N*,*N*-dimethylmethanaminium 2,2,2-trifluoroacetate (VI)**

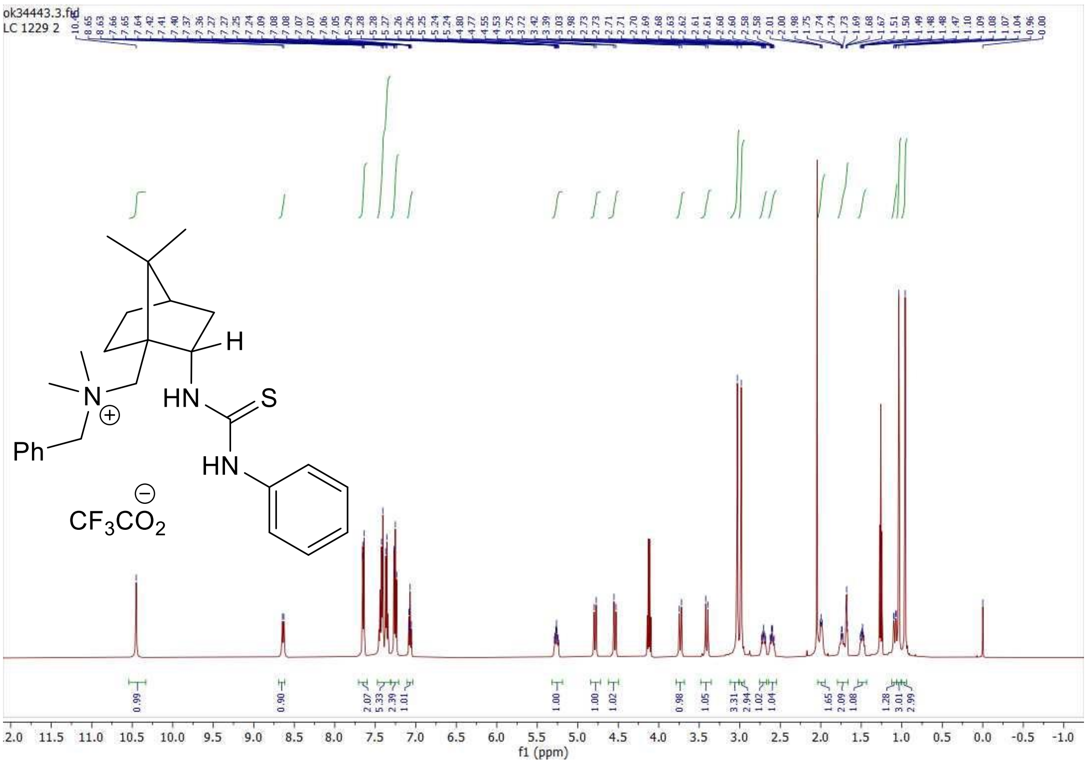

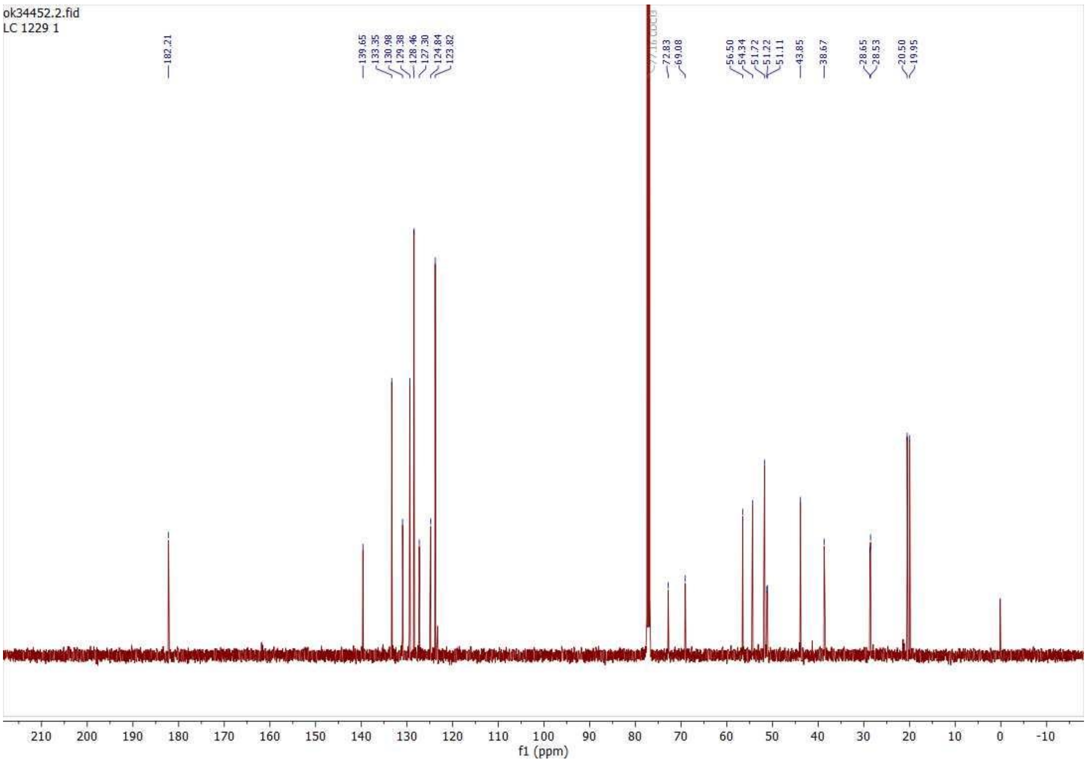

NOESY

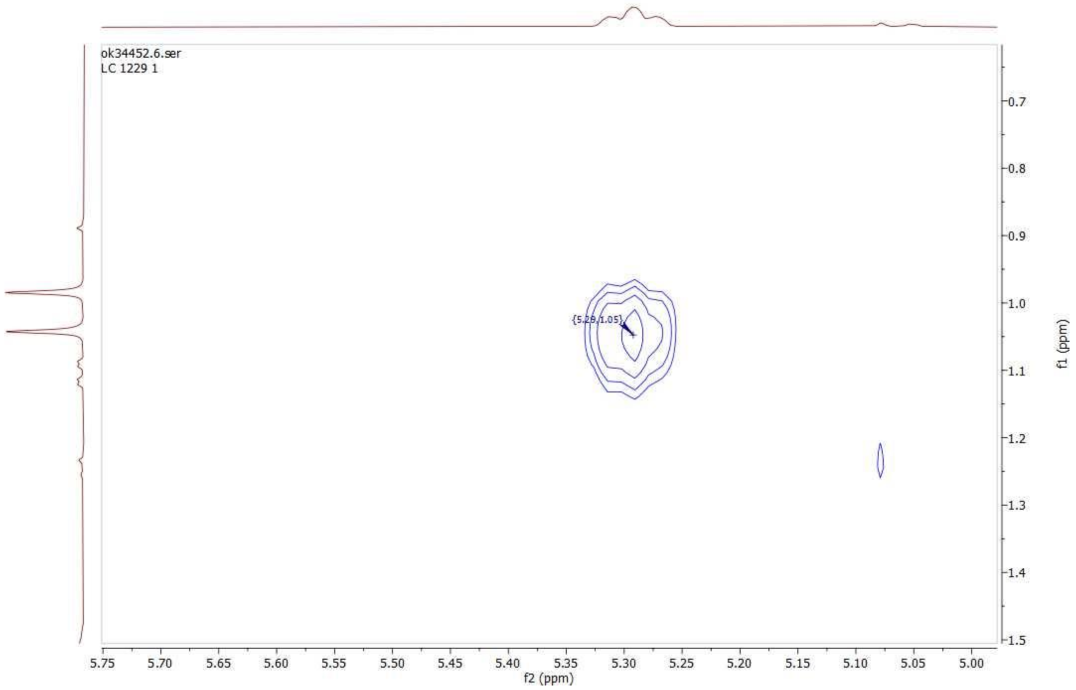

^{19}F-NMR

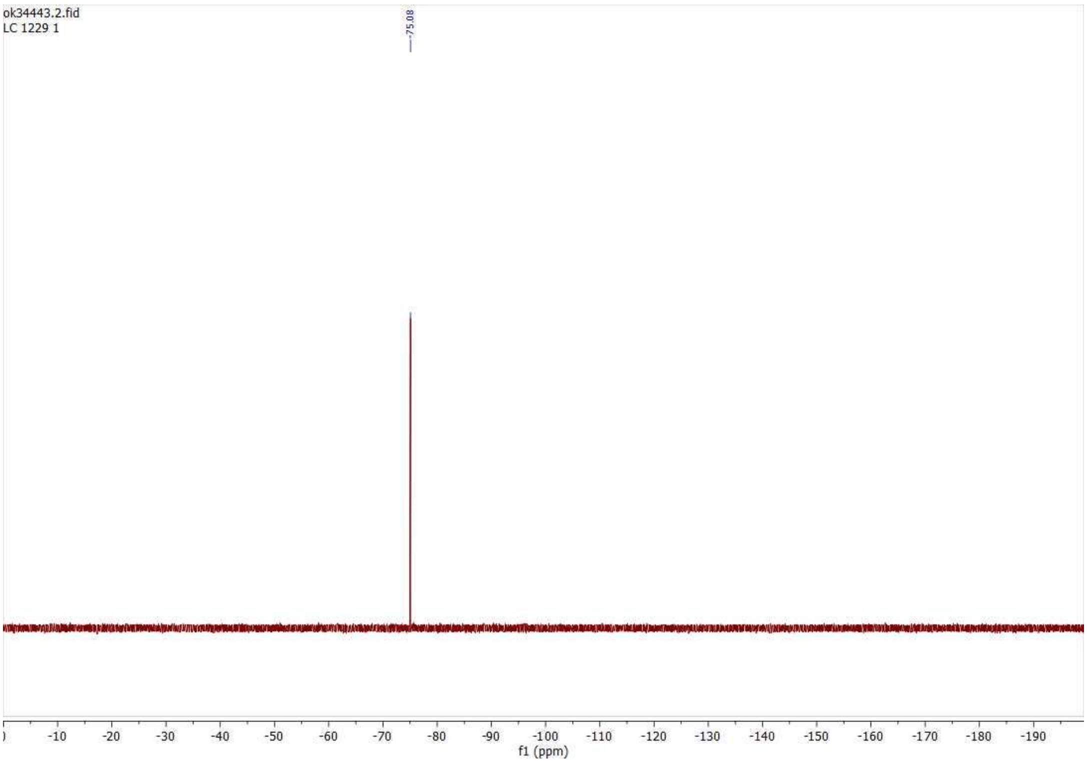

***N*-Benzyl-1-((1*R*,2*S*,4*R*)-7,7-dimethyl-2-(3-phenylureido)bicyclo[2.2.1]heptan-1-yl)-*N*,*N*-dimethylmethanaminium 2,2,2-trifluoroacetate (VII)**

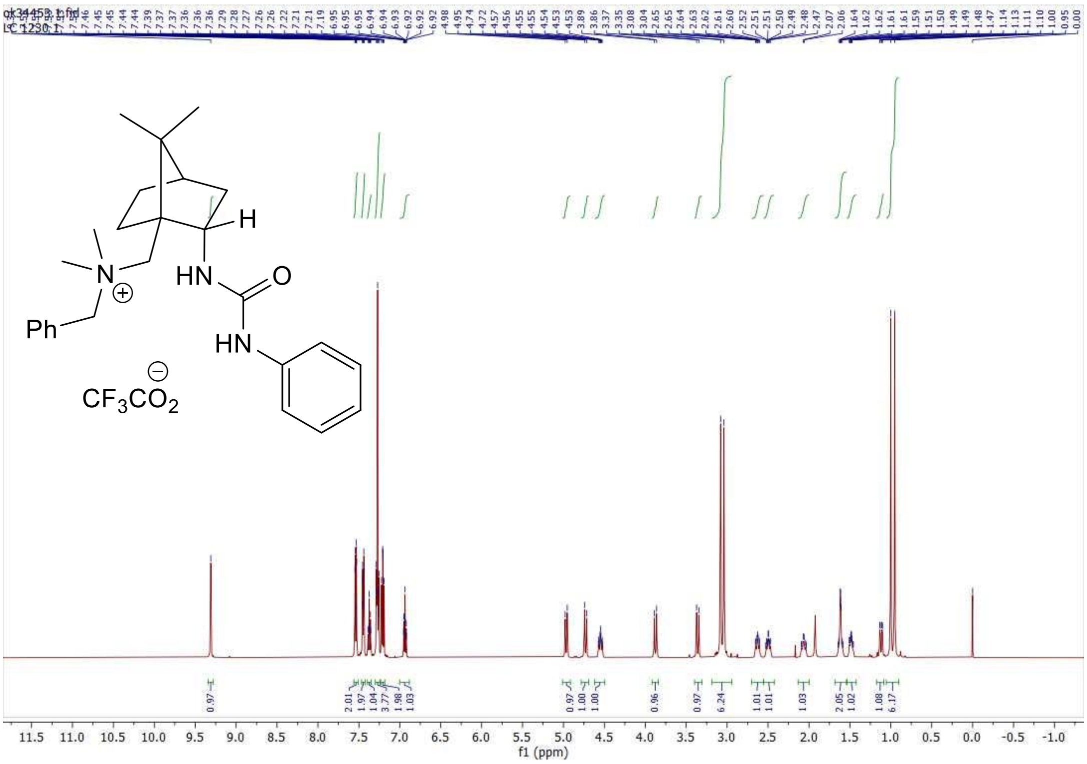

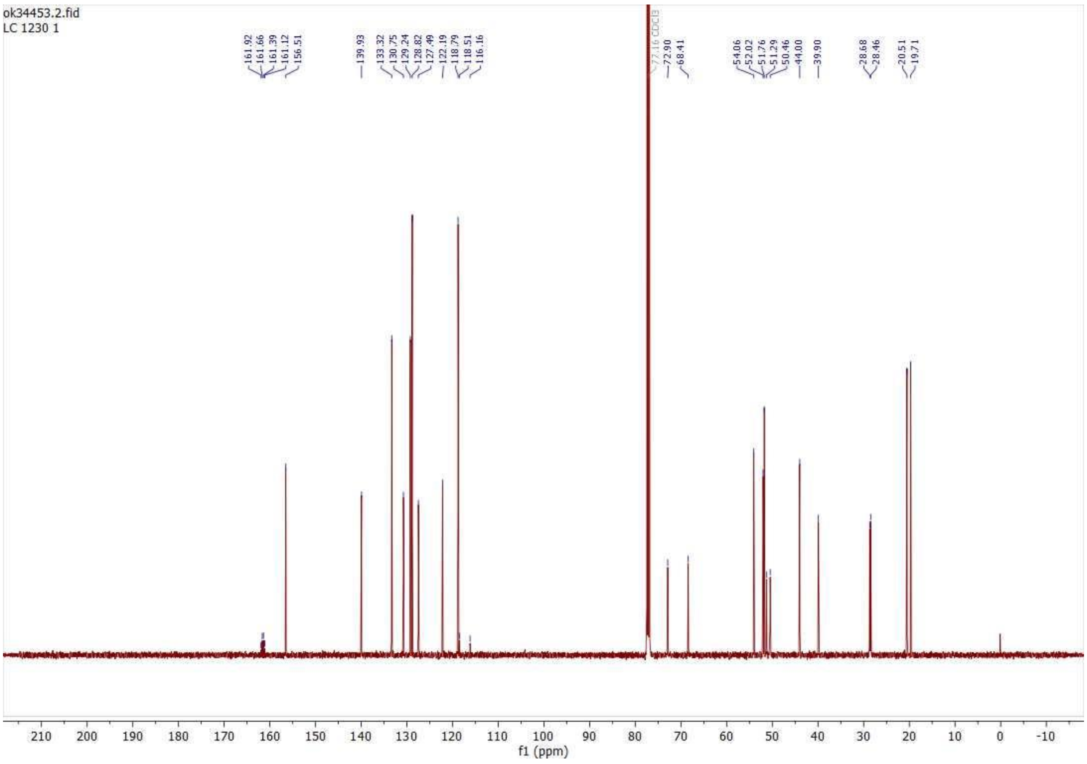

NOESY

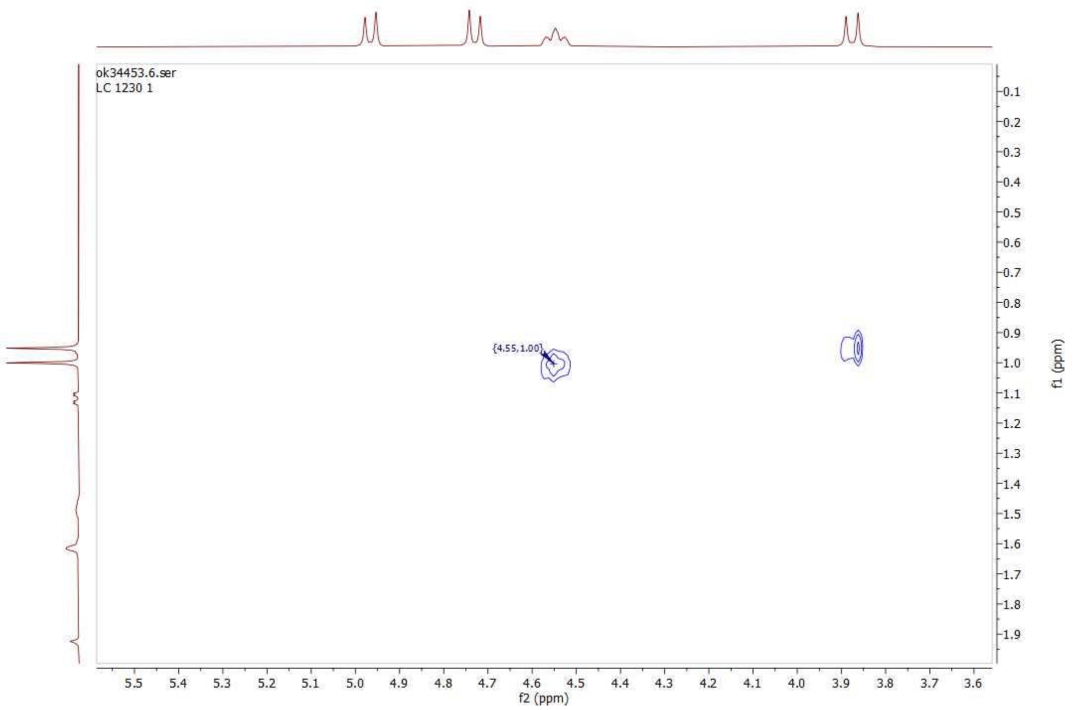

^{19}F-NMR

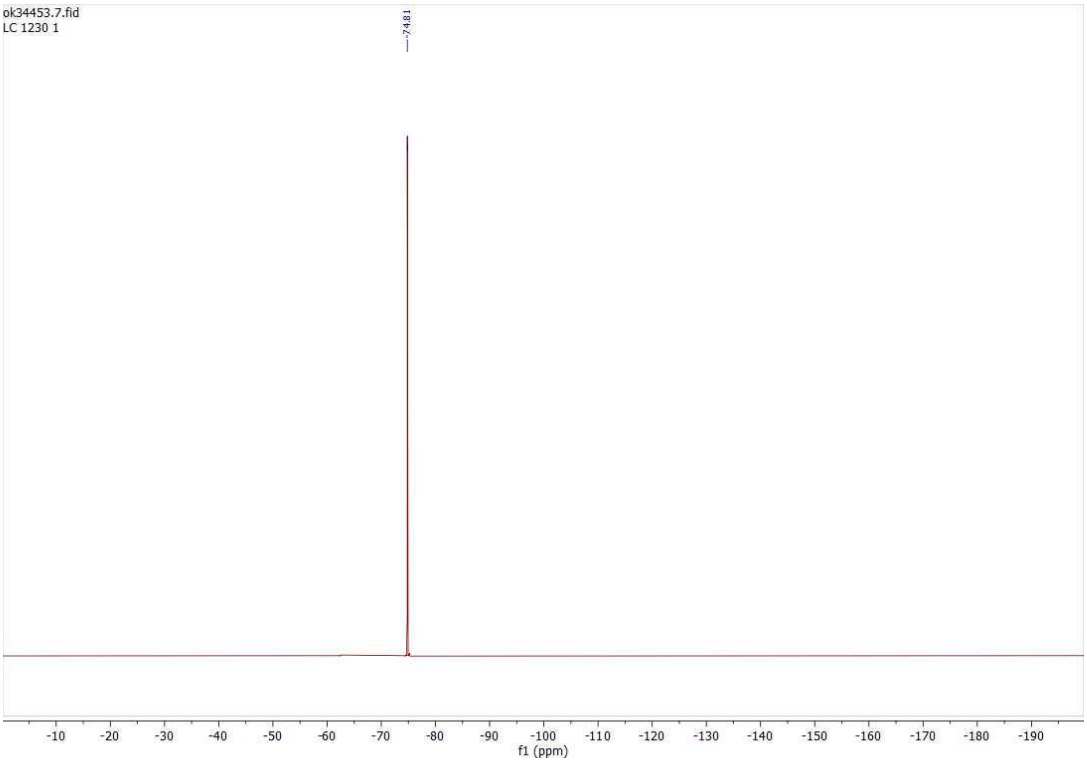

N-Benzyl-1-((1R,2S,4R)-7,7-dimethyl-2-(3-phenylureido)bicyclo[2.2.1]heptan-1-yl)-N,N-dimethylmethanaminium iodide (VIII)

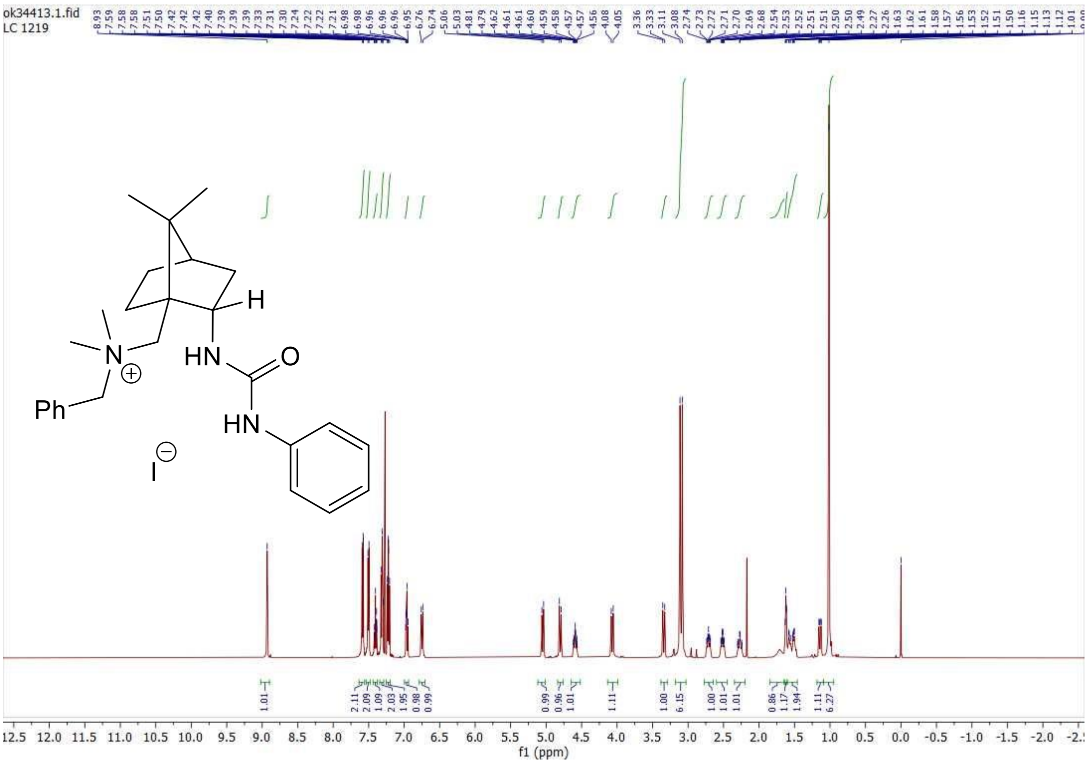

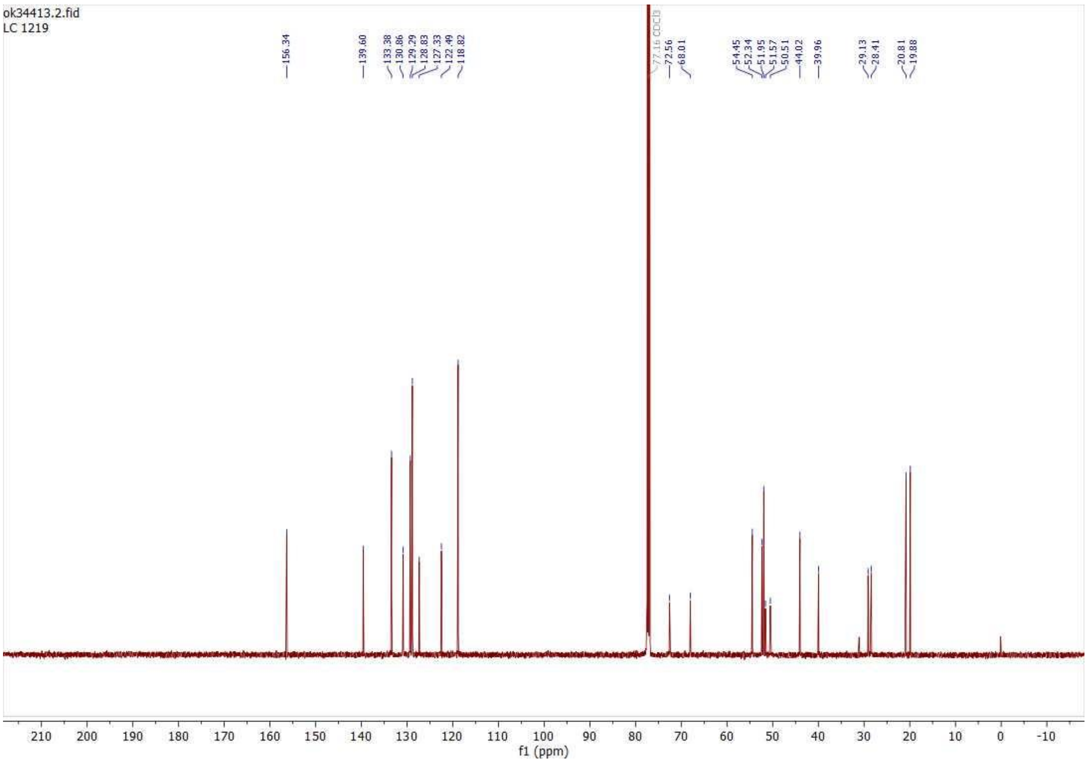

NOESY

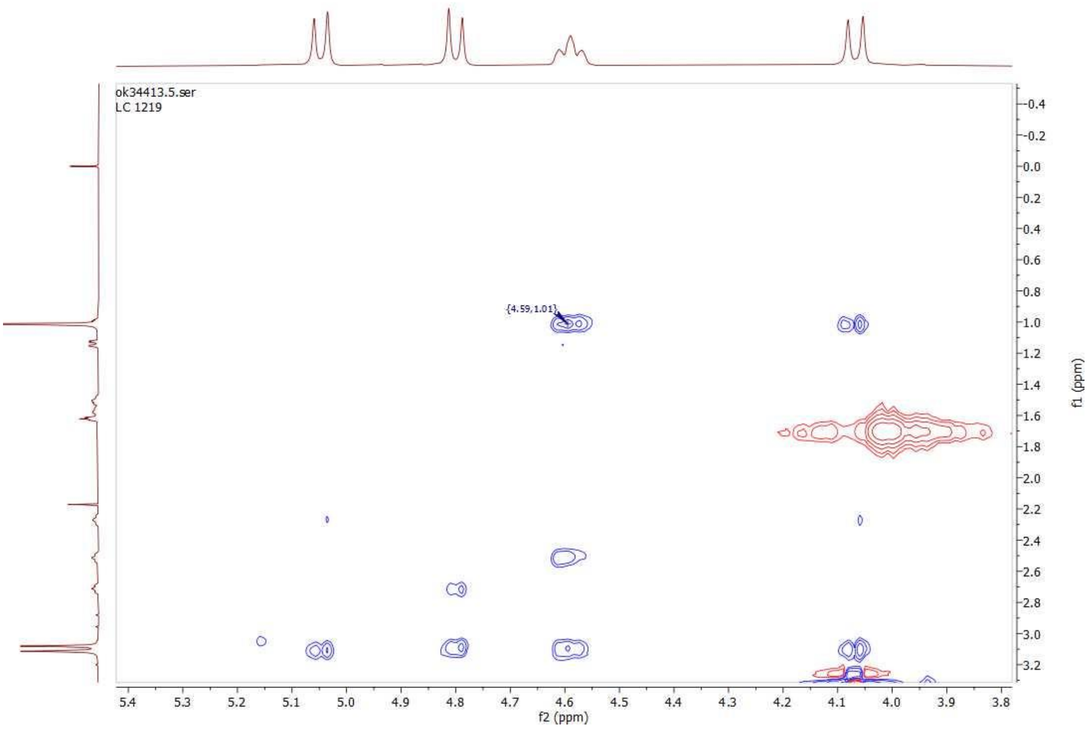

NOESY

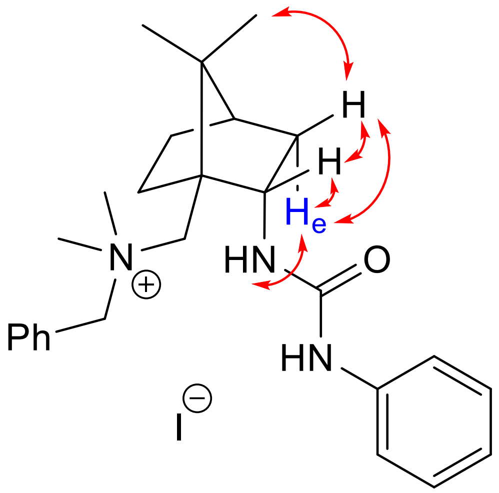

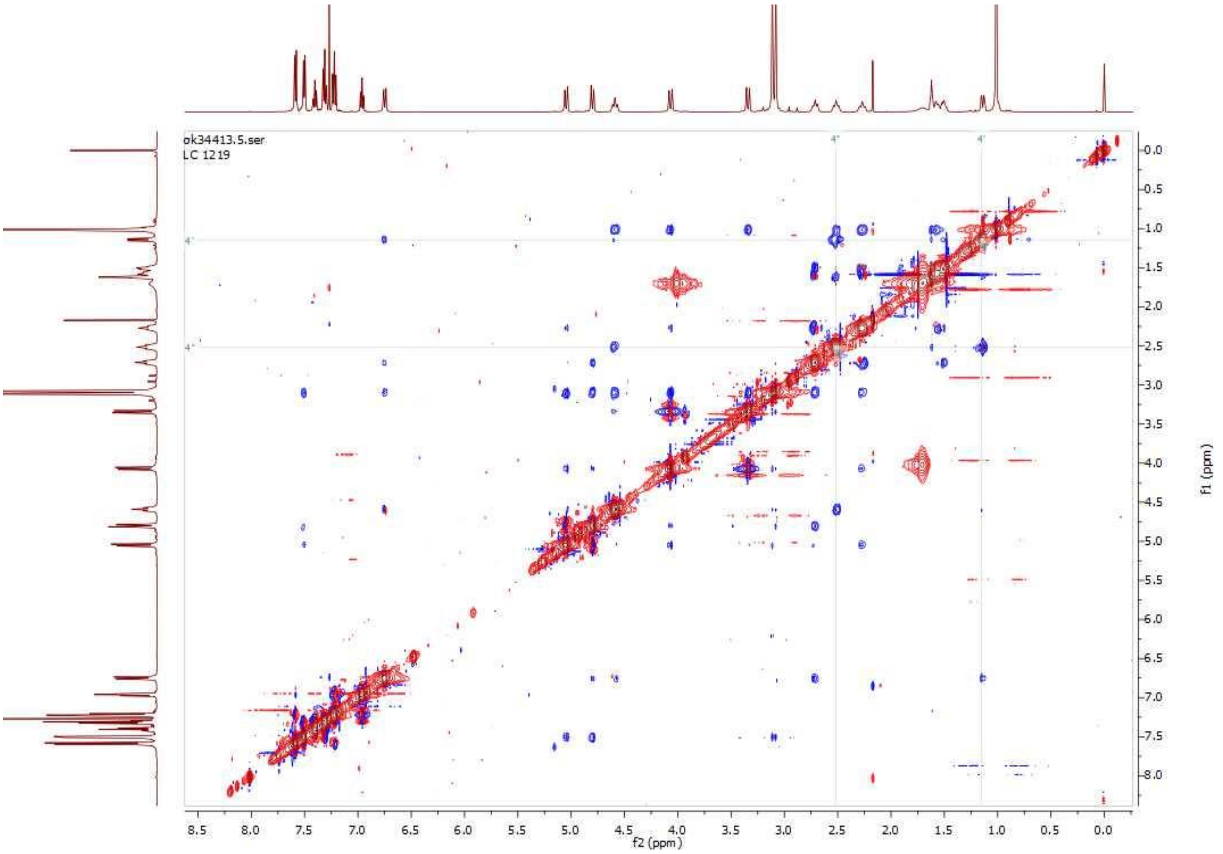

***N*-Benzyl-1-((1*R*,2*S*,4*R*)-2-((2-((3,5-bis(trifluoromethyl)phenyl)amino)-3,4-dioxocyclobut-1-en-1-yl)amino)-7,7-dimethylbicyclo[2.2.1]heptan-1-yl)-*N*,*N*-dimethylmethanaminium 2,2,2-trifluoroacetate (IX)**

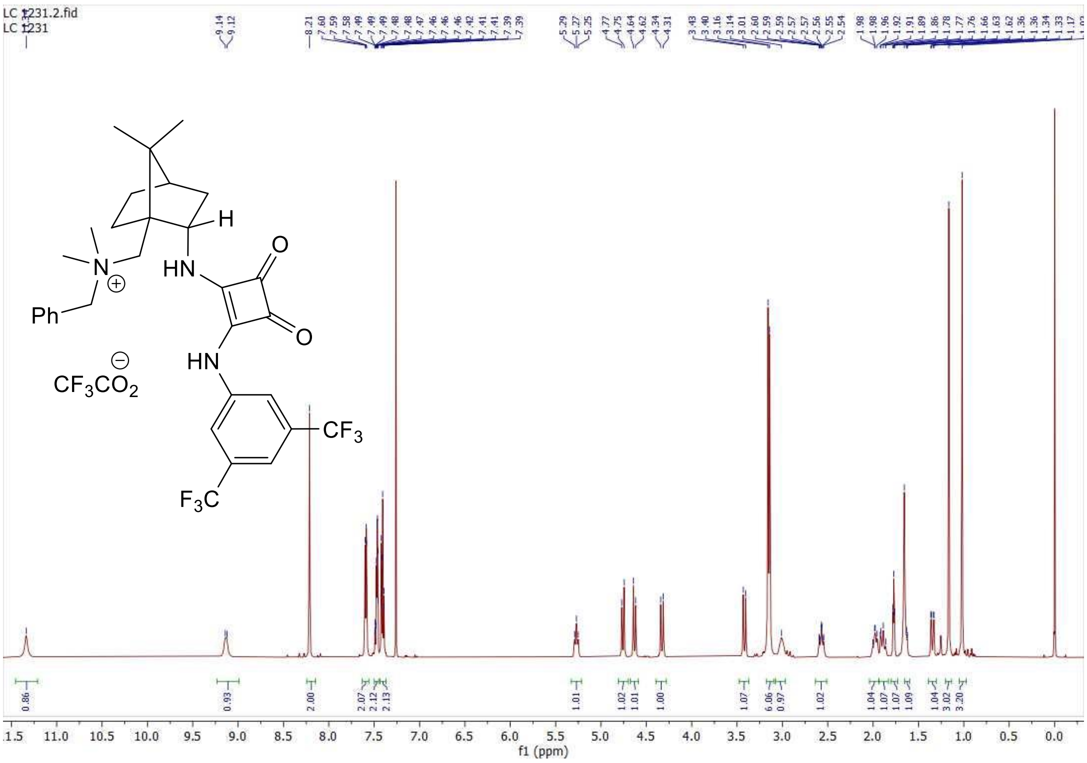

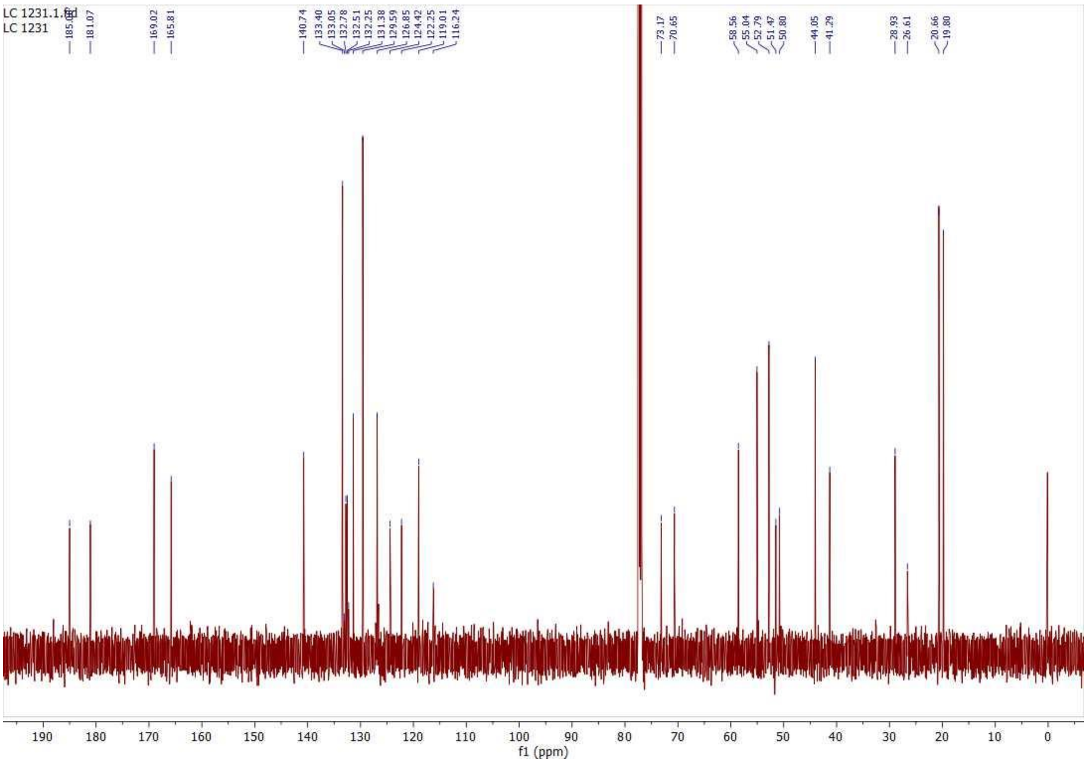

NOESY

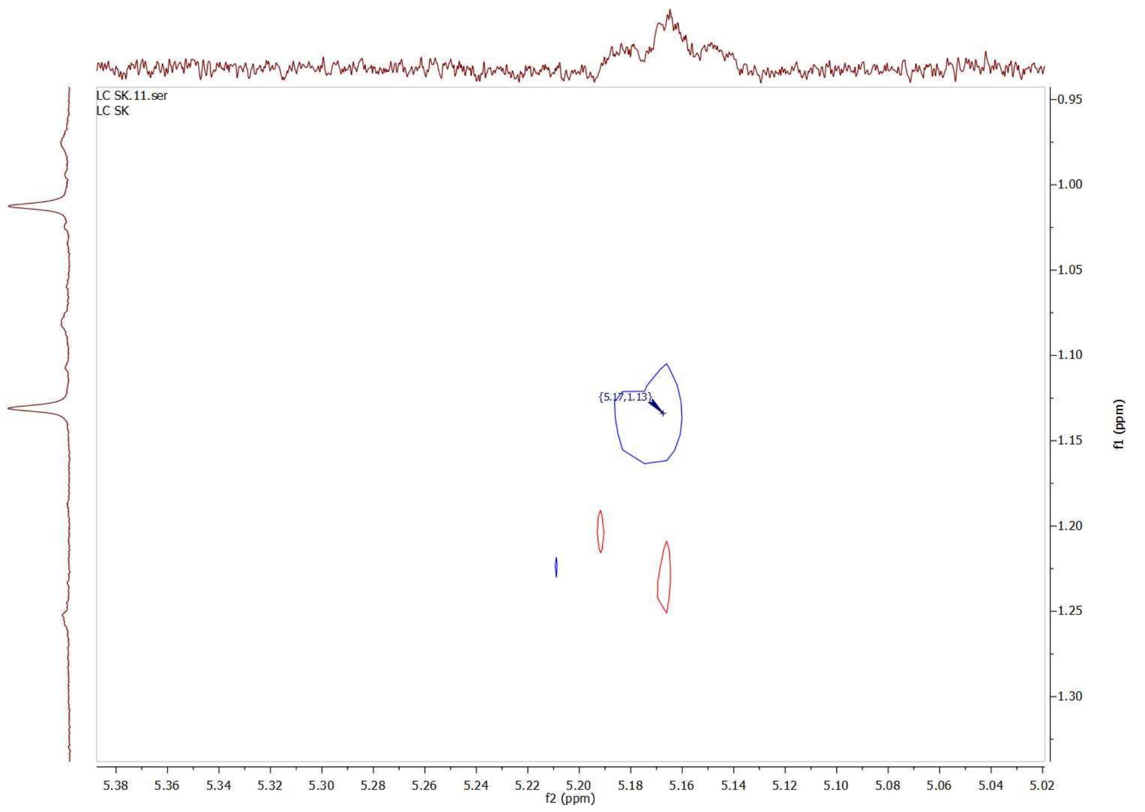

^{19}F-NMR

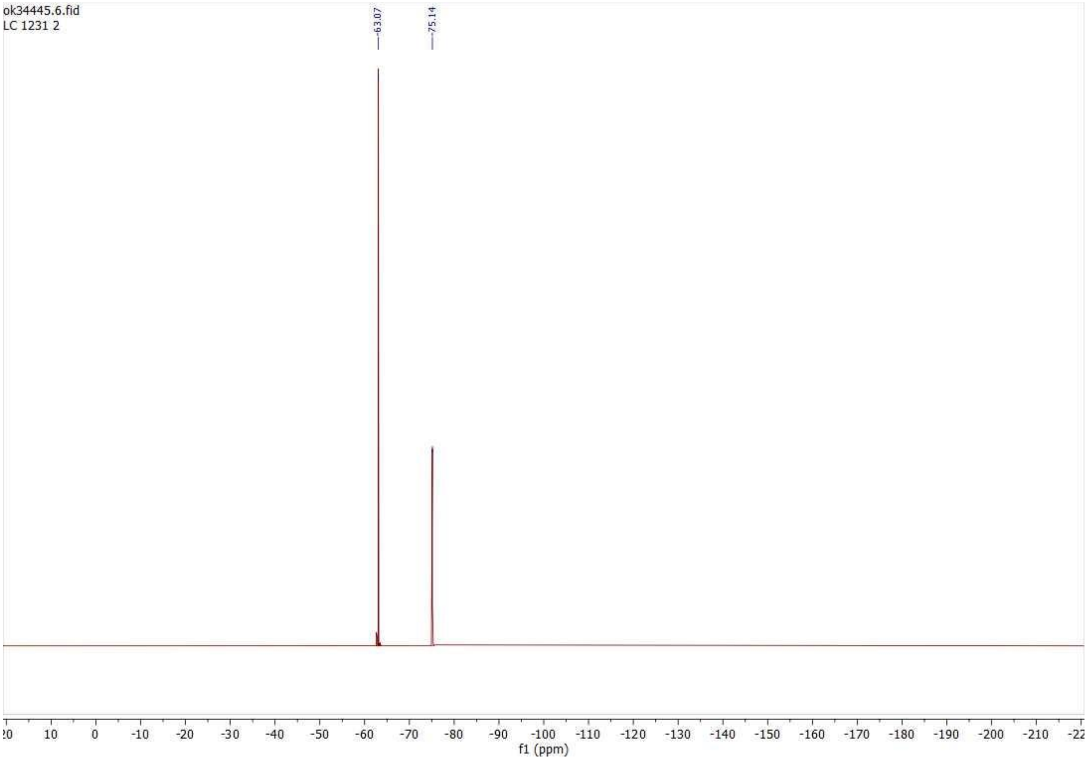

1-benzyl-1-(((1*S*,2*S*,4*R*)-7,7-dimethyl-2-(3-phenylthioureido)bicyclo[2.2.1]heptan-1-yl)methyl)pyrrolidin-1-ium iodide (X)

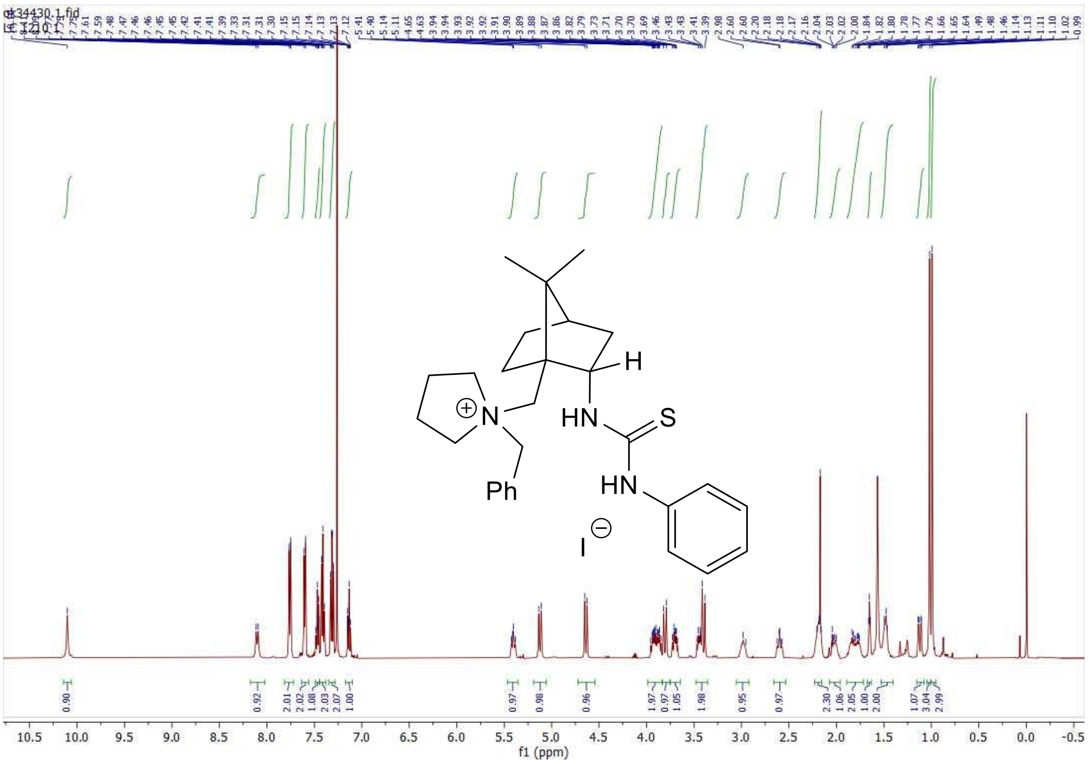

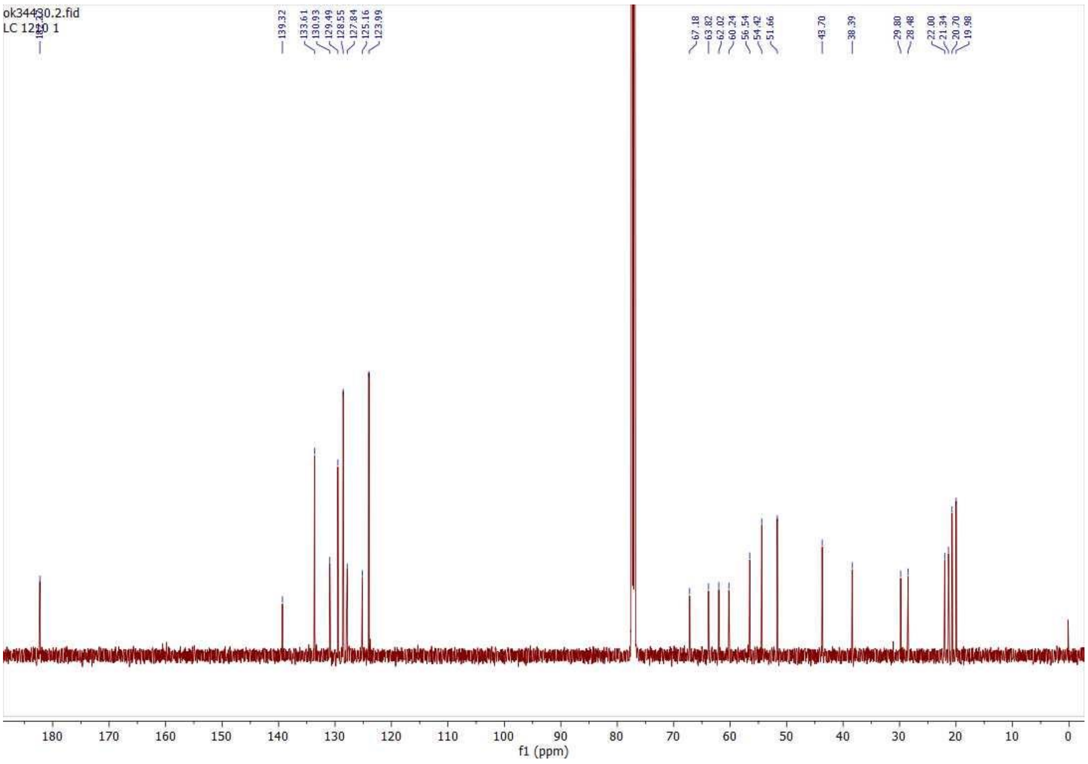

NOESY

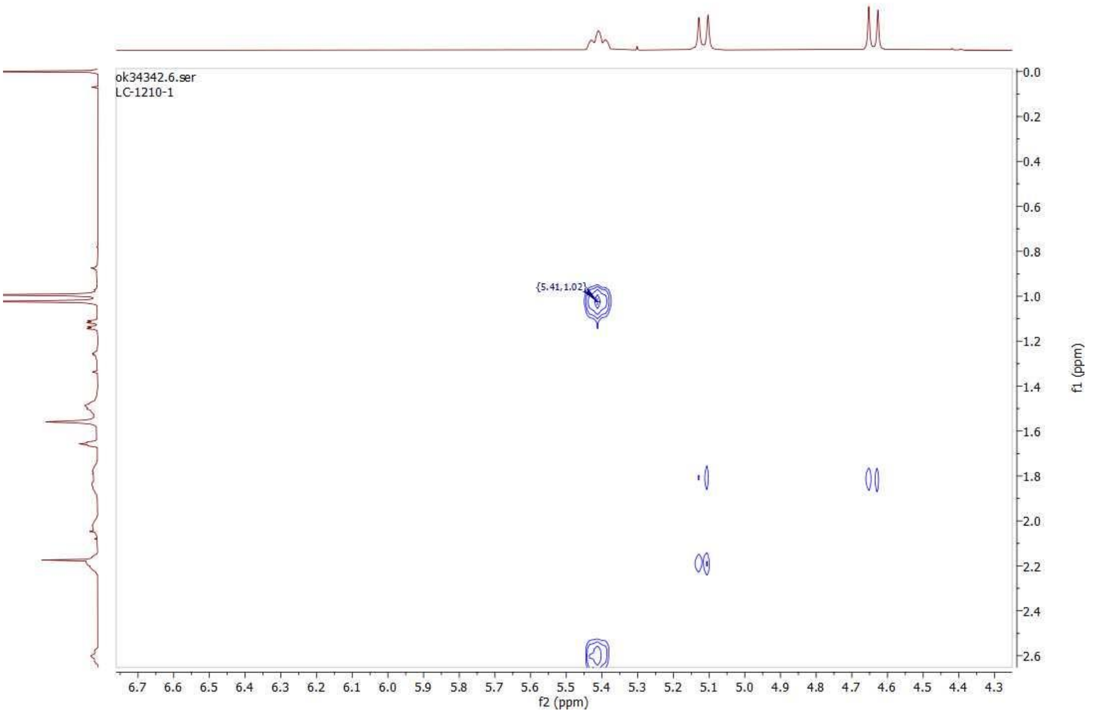
ok34342.6.ser
LC-1210-1
(5.41,1.02)
6.7 6.6 6.5 6.4 6.3 6.2 6.1 6.0 5.9 5.8 5.7 5.6 5.5 5.4 5.3 5.2 5.1 5.0 4.9 4.8 4.7 4.6 4.5 4.4 4.3
f2 (ppm)
0.0 0.2 0.4 0.6 0.8 1.0 1.2 1.4 1.6 1.8 2.0 2.2 2.4 2.6
f1 (ppm)

4. Determination of the absolute configuration at the C-2 chiral center

Table S7. Correlation between the multiplicity of the H-C(3)-*endo* proton (H_e) and the *endo* absolute configuration at the C-2 chiral center of compounds **1a**, **3b**, **5a**, **7a**,**b**, and **III**–**X**.[a]

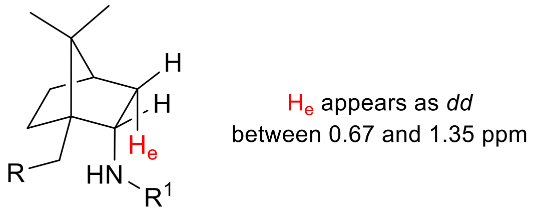

***endo*-isomers**
1a, 3b, 5a, 7a,b, and III–X

Compound	Chemical shift (ppm)	Multiplicity[b]	Compound	Chemical shift (ppm)	Multiplicity[b]
**1a**	0.67	*dd*	**I**	-	-
**2a**	1.06–1.13	-	**II**	-	-
**3a**	1.04	*dd*	**III**	1.15	*dd*
**3b**	1.07	*dd*	**IV**	1.10	*dd*
**4a**	-	-	**V**	1.02	*dd*
**5a**	0.92	*dd*	**VI**	1.08	*dd*
**5b**	0.91–0.96	[c]	**VII**	1.12	*dd*
**6a**	-	-	**VIII**	1.14	*dd*
**7a**	0.89	*dd*	**IX**	1.35	*dd*
**7b**	1.03–1.06	[c]	**X**	1.12	*dd*
**8a**	-	-			

[a] Spectra were recorded in $CDCl_3$. [b] *dd* = doublet of doublet. [c] The multiplicity could not be unambiguously determined due to the overlap with other signals.

5. Copies of HRMS reports of products

tert-Butyl ((1_S_,2_S_,4_R_)-1-((dimethylamino)methyl)-7,7-dimethylbicyclo[2.2.1]heptan-2-yl)carbamate (3a)

Qualitative Compound Report

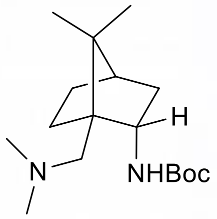

**Data File**	LC1204_001.d	**Sample Name**	LC1204
**Sample Type**	Sample	**Position**	Vial 43
**Instrument Name**	US10310002	**User Name**	TOF-PC\admin
**Acq Method**	Bypass.m	**Acquired Time**	12/15/2022 10:53:06 AM
**IRM Calibration Status**	Success	**DA Method**	Damijana.m
**Comment**			

Compound Label	_m/z_	RT	Algorithm	Mass
Cpd 6: C17 H30 N2 O2	295.2377	0.265	Find by Molecular Feature	294.2305

Compound Identification Results

Ion Mass	Calc Ion Mass	Difference	IonFormula	IonSpecies	Best
295.2377	295.238	0.3	C17 H31 N2 O2	(M+H)+	✓
295.2377	295.2367	-1.1	C15 H29 N5 O	(M+H)+	

MS Spectrum

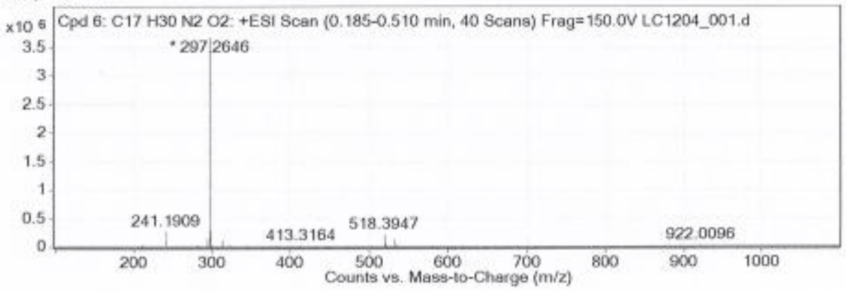

MFE MS Zoomed Spectrum

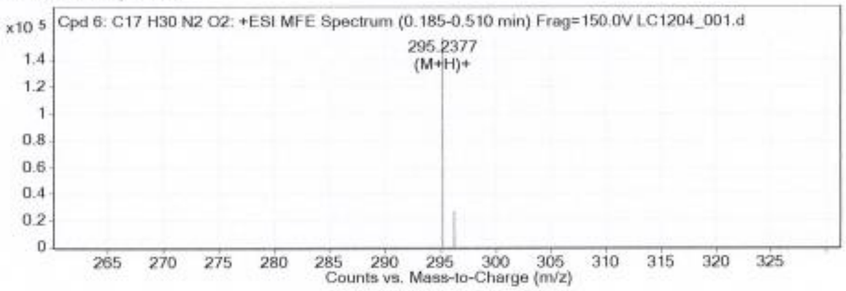

--- End Of Report ---

tert-Butyl ((1_S_,2_R_,4_R_)-1-((dimethylamino)methyl)-7,7-dimethylbicyclo[2.2.1]heptan-2-yl)carbamate (4a)

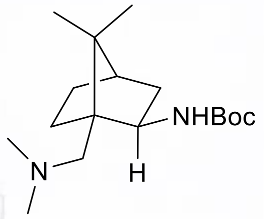

Qualitative Compound Report

**Data File**	LCDAG25_001.d	**Sample Name**	LCDAG25
**Sample Type**	Sample	**Position**	Vial 17
**Instrument Name**	US10310002	**User Name**	TOF-PC\admin
**Acq Method**	Bypass.m	**Acquired Time**	1/10/2023 10:06:38 AM
**IRM Calibration Status**	Success	**DA Method**	Damijana.m
**Comment**			

Compound Label	*m/z*	RT	Algorithm	Mass
Cpd 4: C17 H32 N2 O2	297.2536	0.283	Find by Molecular Feature	296.2464

Compound Identification Results

Ion Mass	Calc Ion Mass	Difference	IonFormula	IonSpecies	Best
297.2536	297.2537	0.1	C17 H33 N2 O2	(M+H)+	✓
297.2536	297.2523	-1.2	C15 H31 N5 O	(M+H)+	

MS Spectrum

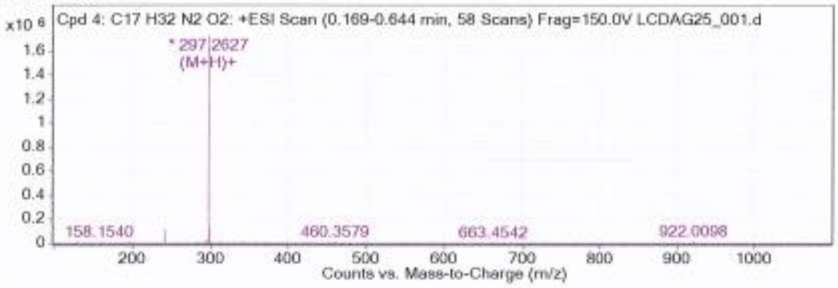

MFE MS Zoomed Spectrum

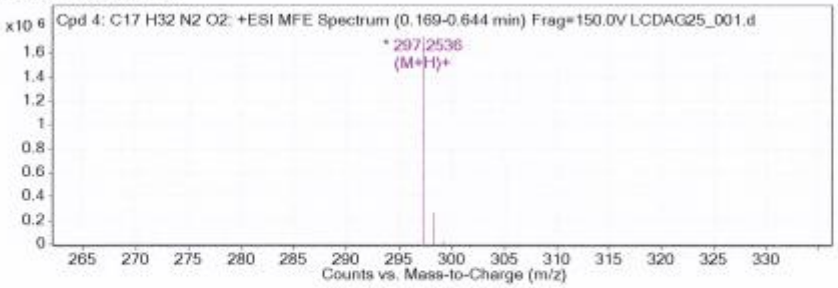

--- End Of Report ---

Agilent Technologies Page 1 of 1 Printed at: 10:09 AM on:1/10/2023

***tert*-Butyl ((1*S*,2*S*,4*R*)-7,7-dimethyl-1-(pyrrolidin-1-ylmethyl)bicyclo[2.2.1]heptan-2-yl)carbamate (3b)**

Qualitative Compound Report

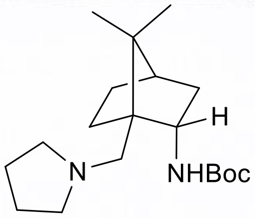

**Data File**	LC1192_001.d	**Sample Name**	LC1192
**Sample Type**	Sample	**Position**	Vial 66
**Instrument Name**	US10310002	**User Name**	TOF-PC\admin
**Acq Method**	Bypass.m	**Acquired Time**	12/20/2022 2:12:08 PM
**IRM Calibration Status**	Success	**DA Method**	Damijana.m
**Comment**			

Compound Label	m/z	RT	Algorithm	Mass
Cpd 2: C19 H34 N2 O2	323.2688	0.261	Find by Molecular Feature	322.2616

Compound Identification Results

Ion Mass	Calc Ion Mass	Difference	IonFormula	IonSpecies	Best
323.2688	323.2693	0.5	C19 H35 N2 O2	(M+H)+	✓
323.2688	323.268	-0.8	C17 H33 N5 O	(M+H)+	

MS Spectrum

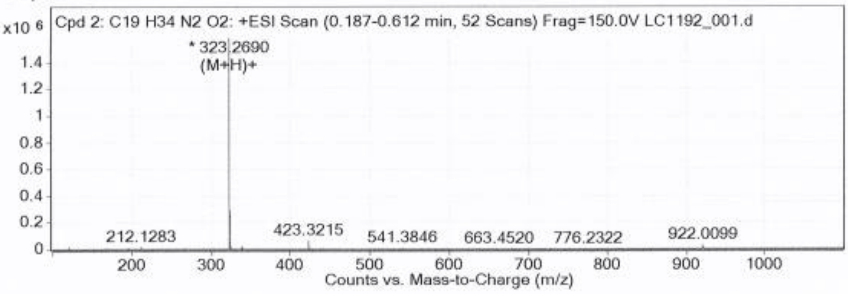

MFE MS Zoomed Spectrum

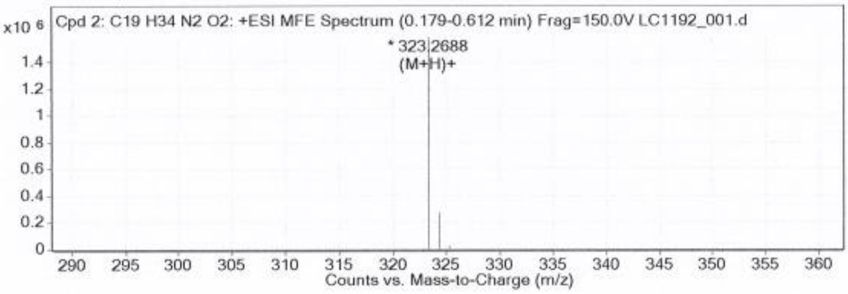

--- End Of Report ---

_N_-Benzyl-1-((1_S_,2_S_,4_R_)-2-((_tert_-butoxycarbonyl)amino)-7,7-dimethylbicyclo[2.2.1]heptan-1-yl)-_N_,_N_-dimethylmethanaminium bromide (5a)

Qualitative Compound Report

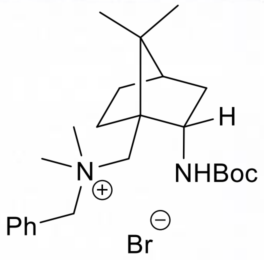

**Data File**	LC1205_001.d	**Sample Name**	LC1205
**Sample Type**	Sample	**Position**	Vial 65
**Instrument Name**	US10310002	**User Name**	TOF-PC\admin
**Acq Method**	Bypass.m	**Acquired Time**	12/20/2022 1:57:45 PM
**IRM Calibration Status**	Success	**DA Method**	Damijana.m
**Comment**			

Compound Label	*m/z*	RT	Algorithm	Mass
Cpd 2: C24 H38 N2 O2	387.3003	0.262	Find by Molecular Feature	386.293

Compound Identification Results

Ion Mass	Calc Ion Mass	Difference	IonFormula	IonSpecies	Best
387.3003	387.3006	0.3	C24 H39 N2 O2	(M+H)+	✓
387.3003	387.2993	-1	C22 H37 N5 O	(M+H)+	
387.3003	387.3025	2.2	C11 H37 N11 O4	(M+H)+	

MS Spectrum

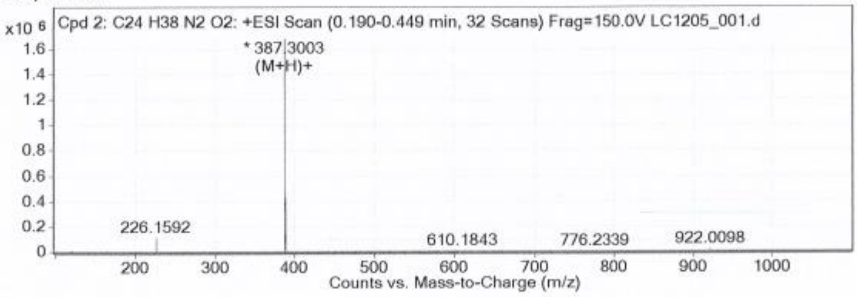

MFE MS Zoomed Spectrum

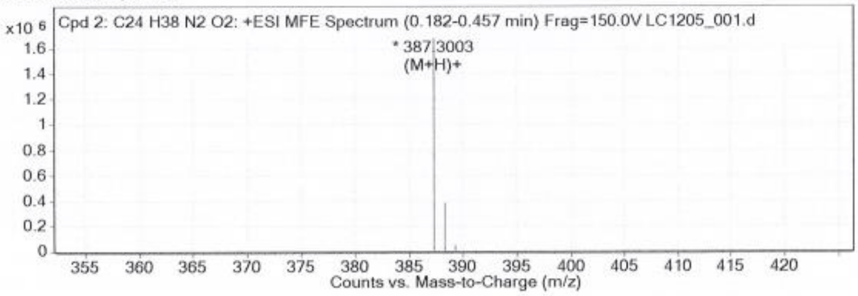

--- End Of Report ---

_N_-Benzyl-1-((1_S_,2_R_,4_R_)-2-((_tert_-butoxycarbonyl)amino)-7,7-dimethylbicyclo[2.2.1]heptan-1-yl)-_N_,_N_-dimethylmethanaminium bromide (6a)

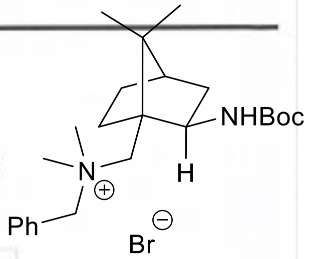

Qualitative Compound Report

**Data File**	LC1157_001.d	**Sample Name**	LC1157
**Sample Type**	Sample	**Position**	Vial 6
**Instrument Name**	US10310002	**User Name**	TOF-PC\admin
**Acq Method**	Bypass.m	**Acquired Time**	1/6/2023 11:10:57 AM
**IRM Calibration Status**	Success	**DA Method**	Damijana.m
**Comment**			

Compound Label	*m/z*	RT	Algorithm	Mass
Cpd 5: C24 H38 N2 O2	387.3004	0.273	Find by Molecular Feature	386.2931

Compound Identification Results

Ion Mass	Calc Ion Mass	Difference	IonFormula	IonSpecies	Best
387.3004	387.3006	0.2	C24 H39 N2 O2	(M+H)+	✓
387.3004	387.2993	-1.1	C22 H37 N5 O	(M+H)+	
387.3004	387.3025	2	C11 H37 N11 O4	(M+H)+	

MS Spectrum

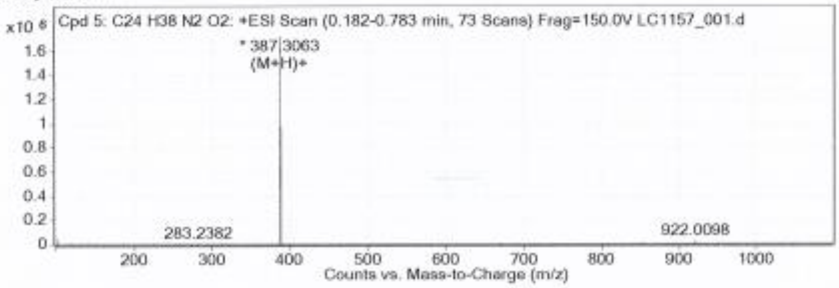

MFE MS Zoomed Spectrum

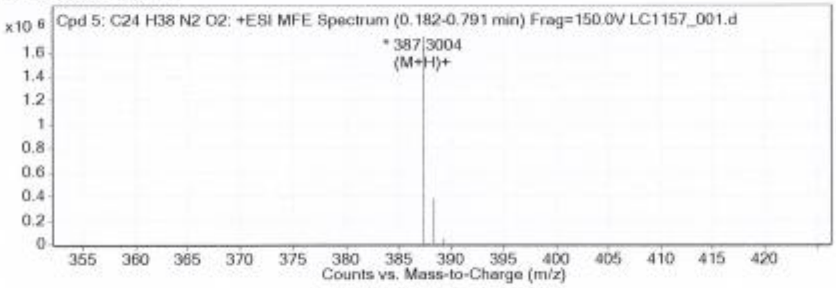

--- End Of Report ---

1-Benzyl-1-(((1*S*,2*S*,4*R*)-2-((*tert*-butoxycarbonyl)amino)-7,7-dimethylbicyclo[2.2.1]heptan-1-yl)methyl)pyrrolidin-1-ium bromide (5b)

Qualitative Compound Report

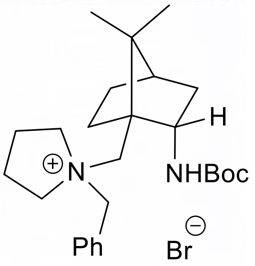

**Data File**	LC1195_002.d	**Sample Name**	LC1195
**Sample Type**	Sample	**Position**	Vial 42
**Instrument Name**	US10310002	**User Name**	TOF-PC\admin
**Acq Method**	Bypass.m	**Acquired Time**	12/15/2022 10:59:08 AM
**IRM Calibration Status**	Success	**DA Method**	Damijana.m
**Comment**			

Compound Label	*m/z*	RT	Algorithm	Mass
Cpd 2: C26 H40 N2 O2	413.3159	0.258	Find by Molecular Feature	412.3088

Compound Identification Results

Ion Mass	Calc Ion Mass	Difference	IonFormula	IonSpecies	Best
413.3159	413.3163	0.3	C26 H41 N2 O2	(M+H)+	✓
413.3159	413.3149	-1	C24 H39 N5 O	(M+H)+	
413.3159	413.3181	2.2	C13 H39 N11 O4	(M+H)+	

MS Spectrum

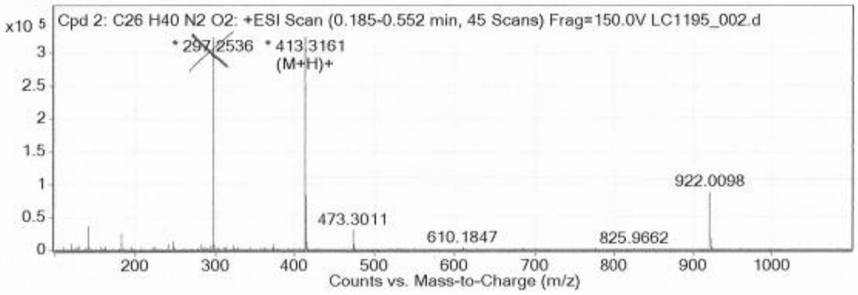

MFE MS Zoomed Spectrum

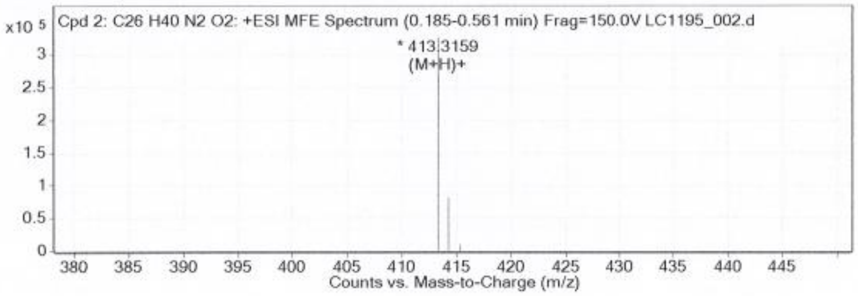

--- End Of Report ---

(1*S*,2*S*,4*R*)-1-((Benzyldimethylammonio)methyl)-7,7-dimethylbicyclo[2.2.1]heptan-2-aminium 2,2,2-trifluoroacetate (7a)

Qualitative Compound Report

**Data File**	LC 1226_001.d	**Sample Name**	LC 1226
**Sample Type**	Sample	**Position**	Vial 3
**Instrument Name**	US10310002	**User Name**	TOF-PC\admin
**Acq Method**	Bypass.m	**Acquired Time**	12/13/2022 12:57:29 PM
**IRM Calibration Status**	Success	**DA Method**	Damijana.m
**Comment**			

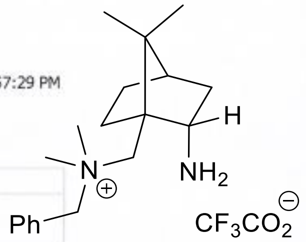

Compound Label	*m/z*	RT	Algorithm	Mass
Cpd 3: C19 H30 N2	287.2483	0.259	Find by Molecular Feature	286.2409

Compound Identification Results

Ion Mass	Calc Ion Mass	Difference	IonFormula	IonSpecies	Best
287.2483	287.2482	-0.1	C19 H31 N2	(M+H)+	✓

MS Spectrum

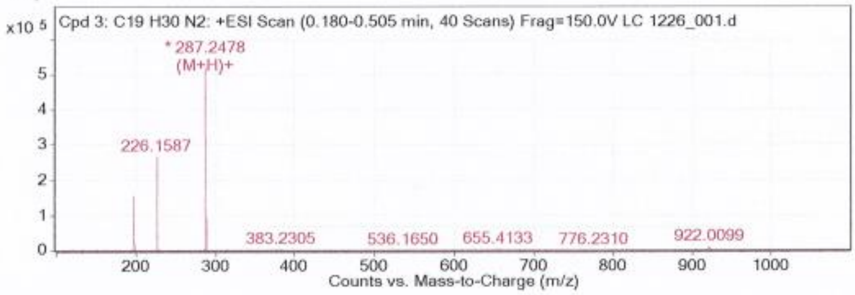

MFE MS Zoomed Spectrum

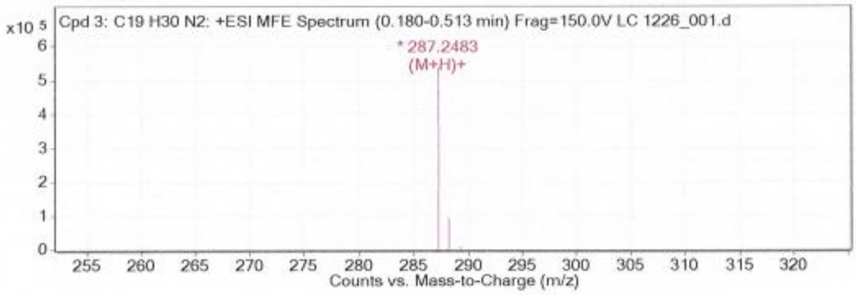

--- End Of Report ---

(1*S*,2*R*,4*R*)-1-((Benzyldimethylammonio)methyl)-7,7-dimethylbicyclo[2.2.1]heptan-2-aminium 2,2,2-trifluoroacetate (8a)

Qualitative Compound Report

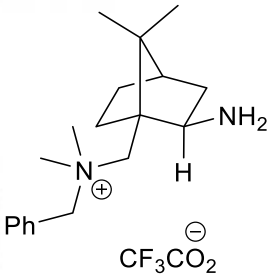

**Data File**	LC1159_001.d	**Sample Name**	LC1159
**Sample Type**	Sample	**Position**	Vial 19
**Instrument Name**	US10310002	**User Name**	TOF-PC\admin
**Acq Method**	Bypass.m	**Acquired Time**	3/2/2021 11:35:10 AM
**IRM Calibration Status**	Success	**DA Method**	Damijana.m
**Comment**			

**Compound Label**	***m/z***	**RT**	**Algorithm**	**Mass**
Cpd 3: C19 H30 N2	287.2477	0.25	Find by Molecular Feature	286.2405

Compound Identification Results

**Ion Mass**	**Calc Ion Mass**	**Difference**	**IonFormula**	**IonSpecies**	**Best**
287.2477	287.2482	0.5	C19 H31 N2	(M+H)+	✓

MS Spectrum

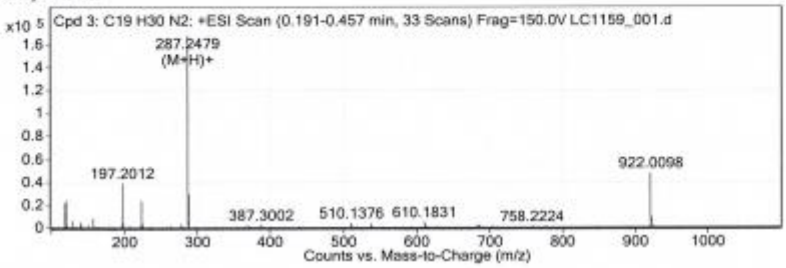

MFE MS Zoomed Spectrum

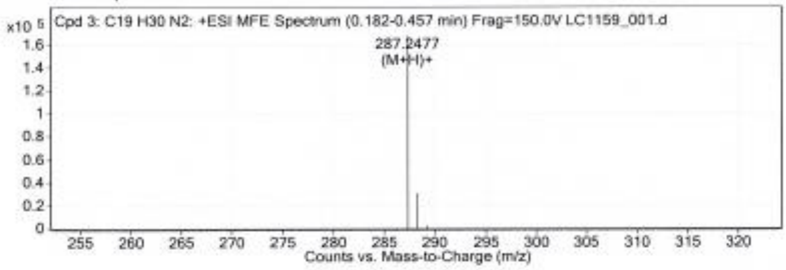

--- End Of Report ---

1-(((1*S*,2*S*,4*R*)-2-Ammonio-7,7-dimethylbicyclo[2.2.1]heptan-1-yl)methyl)-1-benzylpyrrolidin-1-ium iodide (7b)

Qualitative Compound Report

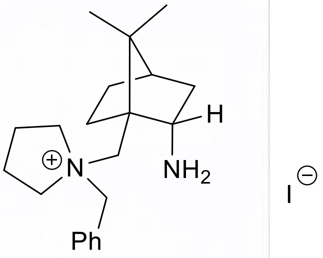

**Data File**	LC1202_001.d	**Sample Name**	LC1202
**Sample Type**	Sample	**Position**	Vial 67
**Instrument Name**	US10310002	**User Name**	TOF-PC\admin
**Acq Method**	Bypass.m	**Acquired Time**	12/20/2022 2:14:32 PM
**IRM Calibration Status**	Success	**DA Method**	Damijana.m
**Comment**			

Compound Label	*m/z*	RT	Algorithm	Mass
Cpd 4: C21 H32 N2	313.2635	0.261	Find by Molecular Feature	312.2564

Compound Identification Results

Ion Mass	Calc Ion Mass	Difference	IonFormula	IonSpecies	Best
313.2635	313.2638	0.3	C21 H33 N2	(M+H)+	✓

MS Spectrum

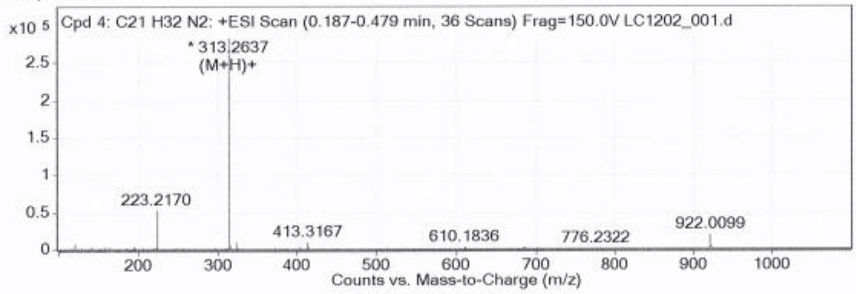

MFE MS Zoomed Spectrum

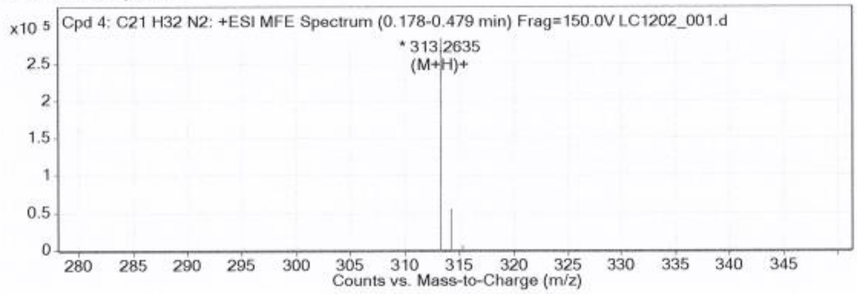

--- End Of Report ---

_N_-Benzyl-1-((1_R_,2_R_,4_R_)-2-(3-(3,5-bis(trifluoromethyl)phenyl)thioureido)-7,7-dimethylbicyclo[2.2.1]heptan-1-yl)-_N_,_N_-dimethylmethanaminium 2,2,2-trifluoroacetate (I)

Qualitative Compound Report

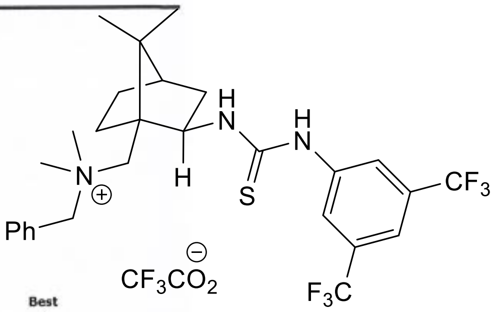

**Data File**	LC1237_001.d	**Sample Name**	LC1237
**Sample Type**	Sample	**Position**	Vial 31
**Instrument Name**	US10310002	**User Name**	TOF-PC\admin
**Acq Method**	Bypass.m	**Acquired Time**	12/8/2022 9:38:34 AM
**IRM Calibration Status**	Success	**DA Method**	Damijana.m
**Comment**			

Compound Label	*m/z*	RT	Algorithm	Mass
Cpd 9: C28 H33 F6 N3 S	558.2374	0.264	Find by Molecular Feature	557.23

Compound Identification Results

Ion Mass	Calc Ion Mass	Difference	IonFormula	IonSpecies	Best
558.2374	558.2372	-0.2	C28 H34 F6 N3 S	(M+H)+	✓
558.2374	558.2377	0.3	C13 H30 F6 N15 O S	(M+H)+	
558.2374	558.2386	1.1	C30 H36 F6 O S	(M+H)+	
558.2374	558.2391	1.7	C15 H32 F6 N12 O2 S	(M+H)+	
558.2374	558.2345	-2.9	C25 H36 F6 N2 O3 S	(M+H)+	

MS Spectrum

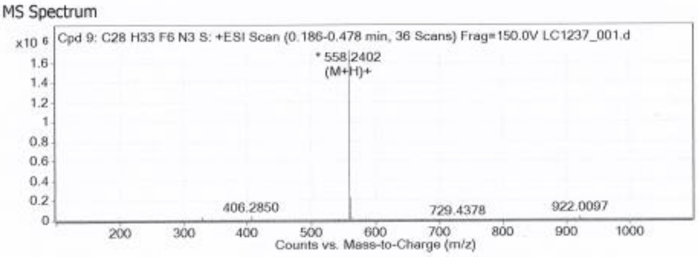

MFE MS Zoomed Spectrum

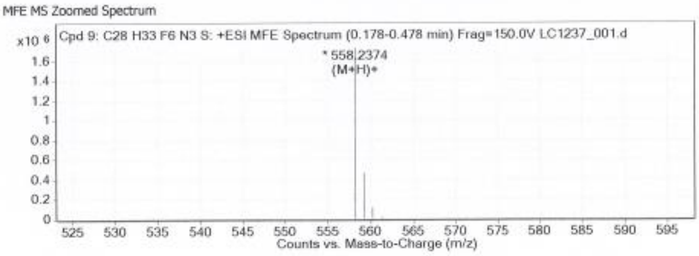

--- End Of Report ---

Agilent Technologies Page 1 of 1 Printed at: 9:41 AM on:12/8/2022

_N_-Benzyl-1-((1_R_,2_R_,4_R_)-2-(3-(3,5-bis(trifluoromethyl)phenyl)thioureido)-7,7-dimethylbicyclo[2.2.1]heptan-1-yl)-_N_,_N_-dimethylmethanaminium iodide (II)

Qualitative Compound Report

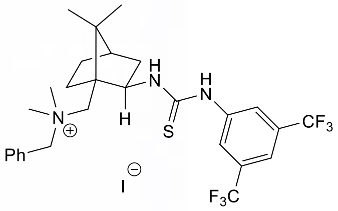

**Data File**	LC1237 1_001.d	**Sample Name**	LC1237 1
**Sample Type**	Sample	**Position**	Vial 32
**Instrument Name**	US10310002	**User Name**	TOF-PC\admin
**Acq Method**	Bypass.m	**Acquired Time**	12/8/2022 9:43:33 AM
**IRM Calibration Status**	Success	**DA Method**	Damijana.m
**Comment**			

Compound Label	*m/z*	RT	Algorithm	Mass
Cpd 4: C28 H33 F6 N3 S	558.2365	0.259	Find by Molecular Feature	557.2294

Compound Identification Results

Ion Mass	Calc Ion Mass	Difference	IonFormula	IonSpecies	Best
558.2365	558.2372	0.8	C28 H34 F6 N3 S	(M+H)+	✓
558.2365	558.2377	1.3	C13 H30 F6 N15 O S	(M+H)+	
558.2365	558.2345	-1.9	C25 H36 F6 N2 O3 S	(M+H)+	
558.2365	558.2386	2.1	C30 H36 F6 O S	(M+H)+	
558.2365	558.2391	2.6	C15 H32 F6 N12 O2 S	(M+H)+	

MS Spectrum

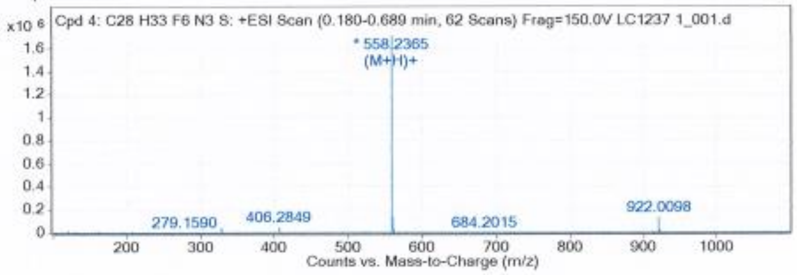

MFE MS Zoomed Spectrum

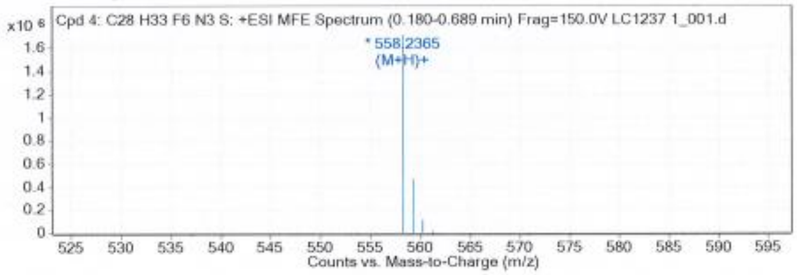

--- End Of Report ---

Agilent Technologies   Page 1 of 1   Printed at: 9:45 AM on:12/8/2022

1-Benzyl-1-(((1*S*,2*S*,4*R*)-2-(3-(3,5-bis(trifluoromethyl)phenyl)thioureido)-7,7-dimethylbicyclo[2.2.1]heptan-1-yl)methyl)pyrrolidin-1-ium iodide (III)

Qualitative Compound Report

**Data File**	LC 1209 1_001.d	**Sample Name**	LC 1209 1
**Sample Type**	Sample	**Position**	Vial 45
**Instrument Name**	US10310002	**User Name**	TOF-PC\admin
**Acq Method**	Bypass.m	**Acquired Time**	4/21/2021 2:53:30 PM
**IRM Calibration Status**	Success	**DA Method**	Damijana.m
**Comment**			

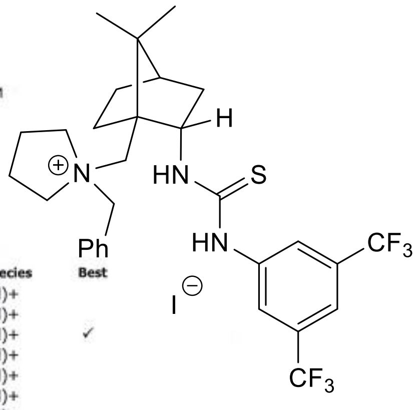

Compound Label	*m/z*	RT	Algorithm	Mass
Cpd 1: C30 H35 F6 N3 S	584.2519	0.258	Find by Molecular Feature	583.245

Compound Identification Results

Ion Mass	Calc Ion Mass	Difference	IonFormula	IonSpecies	Best
584.2519	584.252	0.1	C14 H36 F6 N11 O5 S	(M+H)+	
584.2519	584.252	0.1	C13 H30 F6 N18 S	(M+H)+	
584.2519	584.2529	1	C30 H36 F6 N3 S	(M+H)+	✓
584.2519	584.2534	1.5	C15 H32 F6 N15 O S	(M+H)+	
584.2519	584.2534	1.5	C16 H38 F6 N8 O6 S	(M+H)+	
584.2519	584.2502	-1.7	C27 H38 F6 N2 O3 S	(M+H)+	
584.2519	584.2542	2.3	C32 H38 F6 O S	(M+H)+	
584.2519	584.2547	2.8	C17 H34 F6 N12 O2 S	(M+H)+	

MS Spectrum

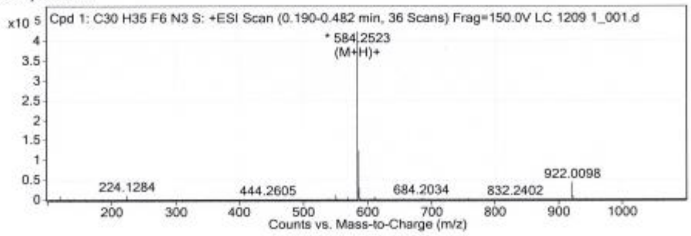

MFE MS Zoomed Spectrum

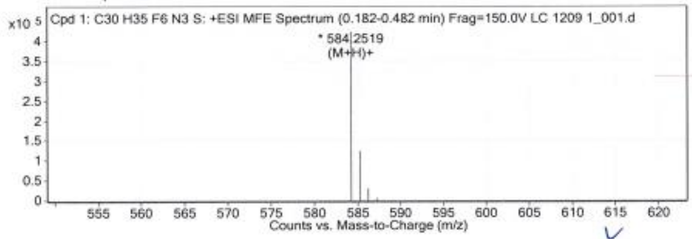

--- End Of Report ---

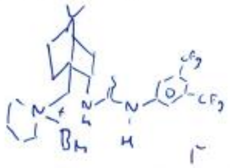

_N_-Benzyl-1-((1_R_,2_S_,4_R_)-2-(3-(3,5-bis(trifluoromethyl)phenyl)thioureido)-7,7-dimethylbicyclo[2.2.1]heptan-1-yl)-_N_,_N_-dimethylmethanaminium 2,2,2-trifluoroacetate (IV)

Qualitative Compound Report

**Data File**	LC1228 2_001.d	**Sample Name**	LC1228 2
**Sample Type**	Sample	**Position**	Vial 22
**Instrument Name**	US10310002	**User Name**	TOF-PC\admin
**Acq Method**	Bypass.m	**Acquired Time**	5/12/2021 2:42:17 PM
**IRM Calibration Status**	Success	**DA Method**	Damijana.m
**Comment**			

Compound Label	*m/z*	RT	Algorithm	Mass
Cpd 10: C28 H33 F6 N3 S	558.2363	0.279	Find by Molecular Feature	557.2292

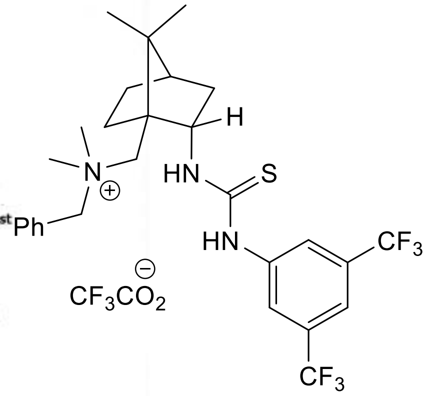

Compound Identification Results

Ion Mass	Calc Ion Mass	Difference	IonFormula	IonSpecies	Best
558.2363	558.2357	-0.6	C19 H34 F6 N5 O7	(M+H)+	
558.2363	558.2357	-0.6	C18 H28 F6 N12 O2	(M+H)+	
558.2363	558.237	0.7	C21 H36 F6 N2 O8	(M+H)+	
558.2363	558.237	0.7	C20 H30 F6 N9 O3	(M+H)+	
558.2363	558.2372	0.9	C28 H34 F6 N3 S	(M+H)+	✓
558.2363	558.2377	1.4	C13 H30 F6 N15 O S	(M+H)+	
558.2363	558.2345	-1.8	C25 H36 F6 N2 O3 S	(M+H)+	
558.2363	558.2344	-1.9	C17 H32 F6 N8 O6	(M+H)+	
558.2363	558.2343	-1.9	C16 H26 F6 N15 O	(M+H)+	
558.2363	558.2384	2.1	C22 H32 F6 N6 O4	(M+H)+	

MS Spectrum

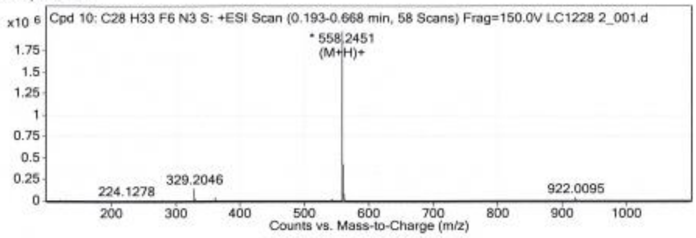

MFE MS Zoomed Spectrum

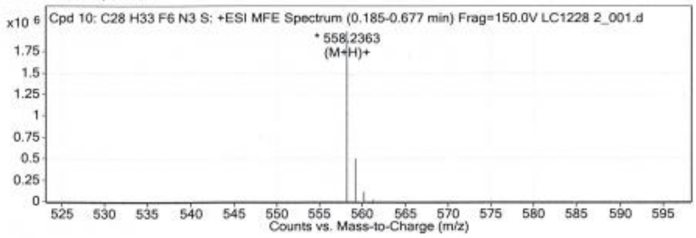

--- End Of Report ---

[illegible]

_N_-Benzyl-1-((1_R_,2_S_,4_R_)-2-(3-(3,5-bis(trifluoromethyl)phenyl)thioureido)-7,7-dimethylbicyclo[2.2.1]heptan-1-yl)-_N_,_N_-dimethylmethanaminium iodide (V)

Qualitative Compound Report

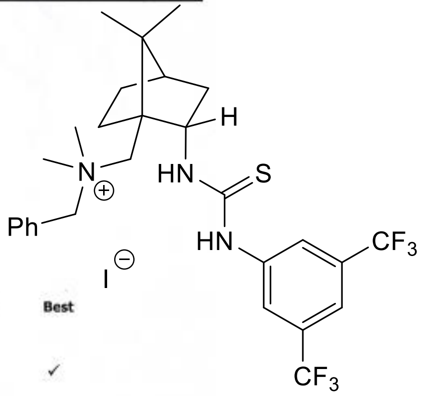

**Data File**	LC1228 I_001.d	**Sample Name**	LC1228 I
**Sample Type**	Sample	**Position**	Vial 16
**Instrument Name**	US10310002	**User Name**	TOF-PC\admin
**Acq Method**	Bypass.m	**Acquired Time**	5/12/2021 2:25:31 PM
**IRM Calibration Status**	Success	**DA Method**	Damijana.m
**Comment**			

Compound Label	_m/z_	RT	Algorithm	Mass
Cpd 1: C28 H33 F6 N3 S	558.2368	0.259	Find by Molecular Feature	557.2296

Compound Identification Results

Ion Mass	Calc Ion Mass	Difference	IonFormula	IonSpecies	Best
558.2368	558.2368	0	C18 H32 F8 N9 S	(M+H)+	
558.2368	558.237	0.2	C23 H33 F7 N6 S	(M+H)+	
558.2368	558.2372	0.4	C28 H34 F6 N3 S	(M+H)+	✓
558.2368	558.2377	0.9	C13 H30 F6 N15 O S	(M+H)+	
558.2368	558.2357	-1.1	C22 H37 F7 N2 O4 S	(M+H)+	
558.2368	558.238	1.2	C15 H33 F9 N9 O S	(M+H)+	
558.2368	558.2382	1.4	C20 H34 F8 N6 O S	(M+H)+	
558.2368	558.2384	1.6	C25 H35 F7 N3 O S	(M+H)+	
558.2368	558.2386	1.8	C30 H36 F6 O S	(M+H)+	
558.2368	558.2345	-2.2	C25 H36 F6 N2 O3 S	(M+H)+	

MS Spectrum

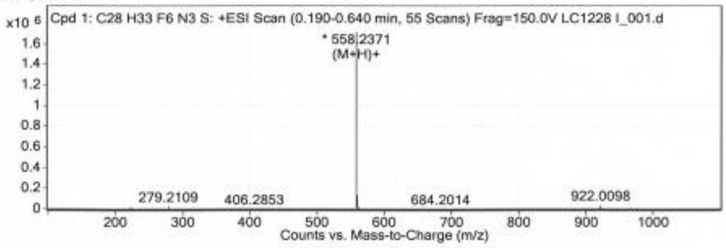

MFE MS Zoomed Spectrum

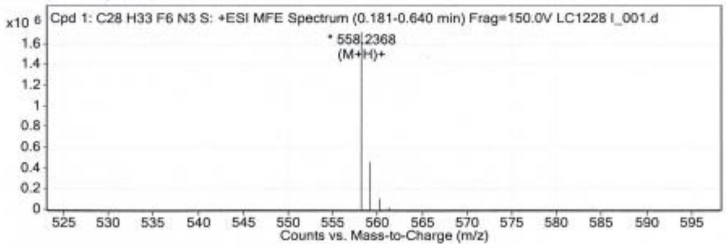

--- End Of Report ---

N-Benzyl-1-((1R,2S,4R)-7,7-dimethyl-2-(3-phenylthioureido)bicyclo[2.2.1]heptan-1-yl)-N,N-dimethylmethanaminium 2,2,2-trifluoroacetate (VI)

Qualitative Compound Report

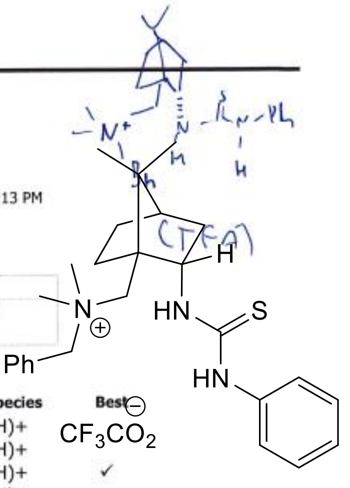

**Data File**	LC1229_001.d	**Sample Name**	LC1229
**Sample Type**	Sample	**Position**	Vial 21
**Instrument Name**	US10310002	**User Name**	TOF-PC\admin
**Acq Method**	Bypass.m	**Acquired Time**	5/12/2021 2:38:13 PM
**IRM Calibration Status**	Success	**DA Method**	Damijana.m
**Comment**			

Compound Label	*m/z*	RT	Algorithm	Mass
Cpd 1: C26 H35 N3 S	422.2618	0.261	Find by Molecular Feature	421.2546

Compound Identification Results

Ion Mass	Calc Ion Mass	Difference	IonFormula	IonSpecies	Best
422.2618	422.2623	0.5	C18 H32 N9 O3	(M+H)+	
422.2618	422.2623	0.5	C19 H38 N2 O8	(M+H)+	
422.2618	422.2624	0.7	C26 H36 N3 S	(M+H)+	✓
422.2618	422.2609	-0.8	C17 H36 N5 O7	(M+H)+	
422.2618	422.2609	-0.9	C16 H30 N12 O2	(M+H)+	
422.2618	422.2629	1.2	C11 H32 N15 O S	(M+H)+	
422.2618	422.2604	-1.4	C31 H34 O	(M+H)+	
422.2618	422.2636	1.8	C20 H34 N6 O4	(M+H)+	
422.2618	422.2598	-2	C23 H38 N2 O3 S	(M+H)+	
422.2618	422.2638	2	C28 H38 O S	(M+H)+	

MS Spectrum

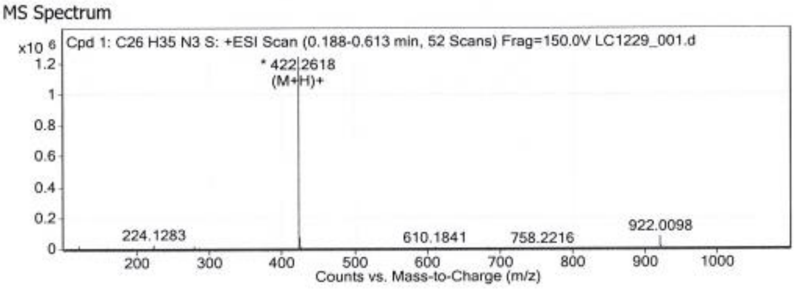

MFE MS Zoomed Spectrum

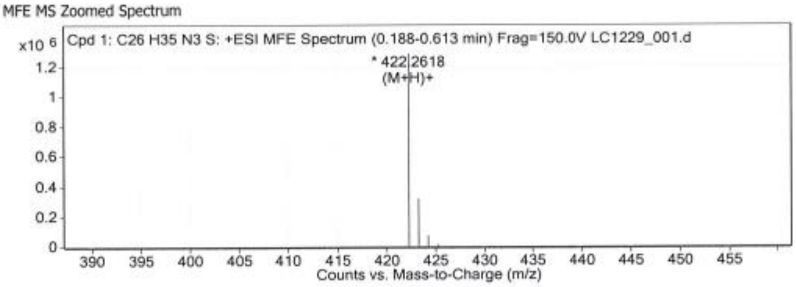

--- End Of Report ---

N-Benzyl-1-((1R,2S,4R)-7,7-dimethyl-2-(3-phenylureido)bicyclo[2.2.1]heptan-1-yl)-N,N-dimethylmethanaminium 2,2,2-trifluoroacetate (VII)

Qualitative Compound Report

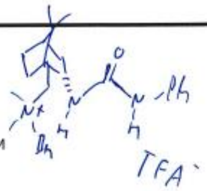

**Data File**	LC1230_001.d	**Sample Name**	LC1230
**Sample Type**	Sample	**Position**	Vial 17
**Instrument Name**	US10310002	**User Name**	TOF-PC\admin
**Acq Method**	Bypass.m	**Acquired Time**	5/12/2021 2:30:53 PM
**IRM Calibration Status**	Success	**DA Method**	Damijana.m
**Comment**			

Compound Label	m/z	RT	Algorithm	Mass
Cpd 2: C26 H35 N3 O	406.285	0.266	Find by Molecular Feature	405.2777

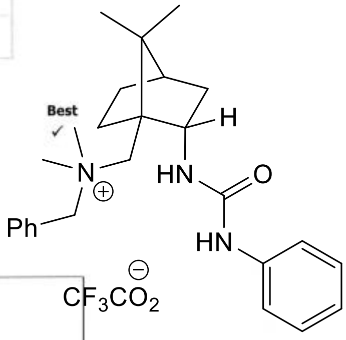

Compound Identification Results

Ion Mass	Calc Ion Mass	Difference	IonFormula	IonSpecies	Best
406.285	406.2853	0.2	C26 H36 N3 O	(M+H)+	✓
406.285	406.2858	0.7	C11 H32 N15 O2	(M+H)+	
406.285	406.2839	-1.1	C24 H34 N6	(M+H)+	
406.285	406.2866	1.6	C28 H38 O2	(M+H)+	
406.285	406.2871	2.1	C13 H34 N12 O3	(M+H)+	

MS Spectrum

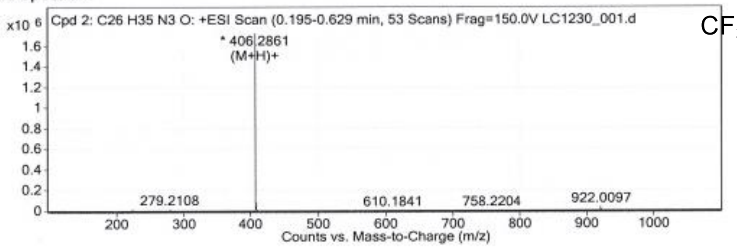

MFE MS Zoomed Spectrum

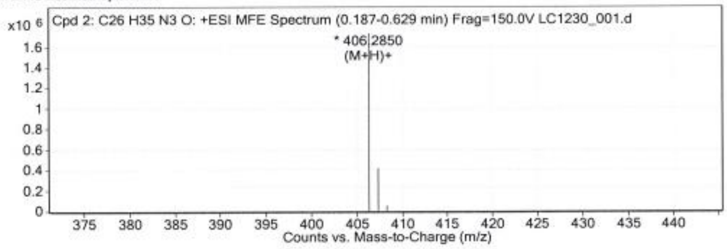

--- End Of Report ---

***N*-Benzyl-1-((1*R*,2*S*,4*R*)-7,7-dimethyl-2-(3-phenylureido)bicyclo[2.2.1]heptan-1-yl)-*N*,*N*-dimethylmethanaminium iodide (VIII)**

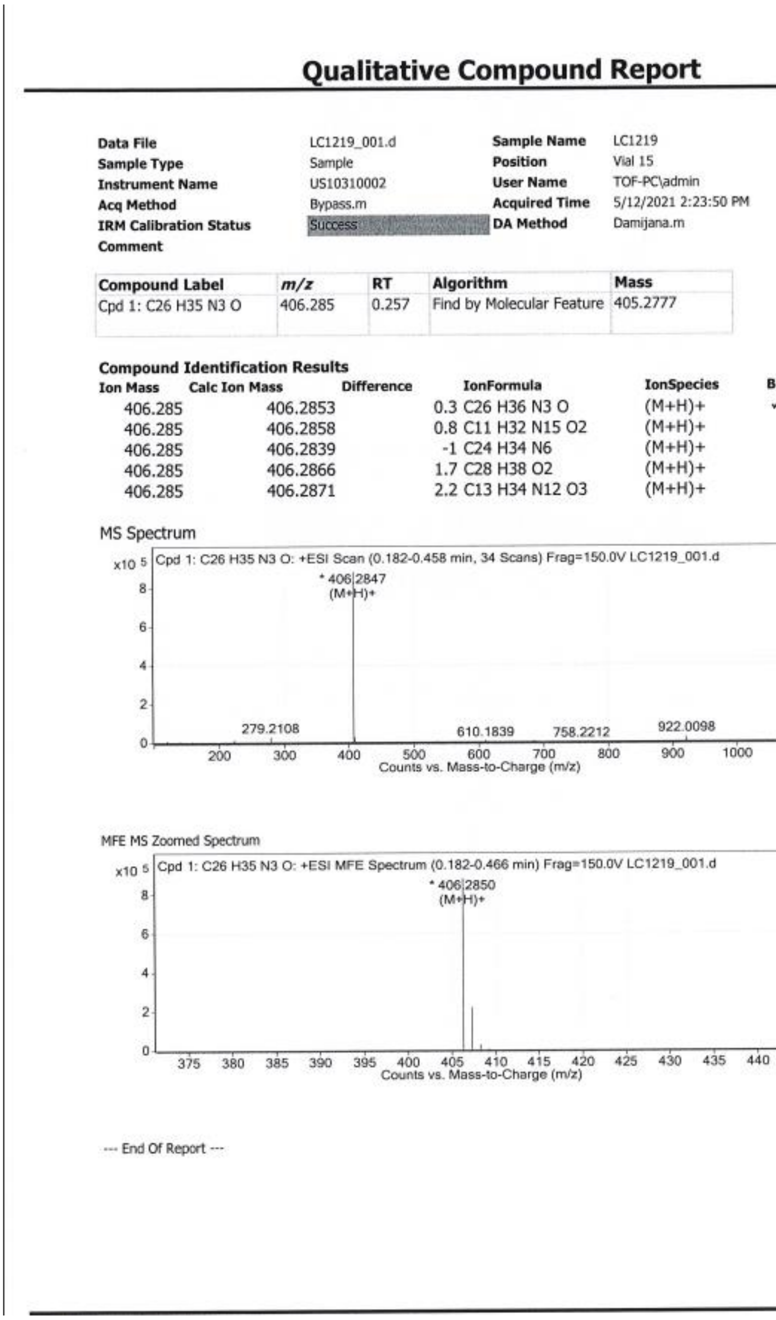

Qualitative Compound Report

**Data File**	LC1219_001.d	**Sample Name**	LC1219
**Sample Type**	Sample	**Position**	Vial 15
**Instrument Name**	US10310002	**User Name**	TOF-PC\admin
**Acq Method**	Bypass.m	**Acquired Time**	5/12/2021 2:23:50 PM
**IRM Calibration Status**	Success	**DA Method**	Damijana.m
**Comment**			

Compound Label	*m/z*	RT	Algorithm	Mass
Cpd 1: C26 H35 N3 O	406.285	0.257	Find by Molecular Feature	405.2777

Compound Identification Results

Ion Mass	Calc Ion Mass	Difference	IonFormula	IonSpecies	Best
406.285	406.2853	0.3	C26 H36 N3 O	(M+H)+	✓
406.285	406.2858	0.8	C11 H32 N15 O2	(M+H)+	
406.285	406.2839	-1	C24 H34 N6	(M+H)+	
406.285	406.2866	1.7	C28 H38 O2	(M+H)+	
406.285	406.2871	2.2	C13 H34 N12 O3	(M+H)+	

MS Spectrum

MFE MS Zoomed Spectrum

--- End Of Report ---

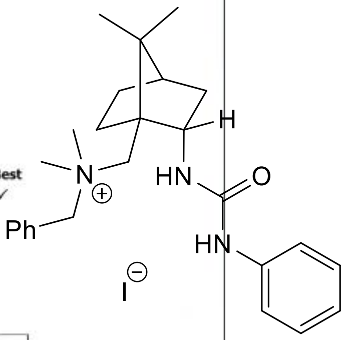

***N*-Benzyl-1-((1*R*,2*S*,4*R*)-2-((2-((3,5-bis(trifluoromethyl)phenyl)amino)-3,4-dioxocyclobut-1-en-1-yl)amino)-7,7-dimethylbicyclo[2.2.1]heptan-1-yl)-*N*,*N*-dimethylmethanaminium 2,2,2-trifluoroacetate (IX)**

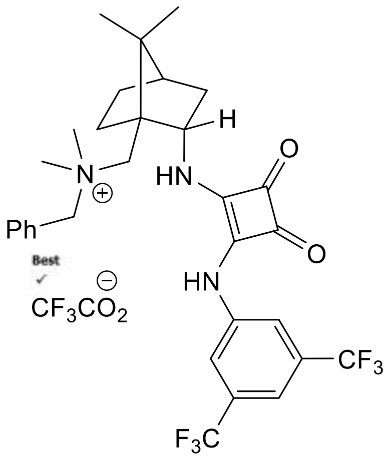

Qualitative Compound Report

**Data File**	LC1231_001.d	**Sample Name**	LC1231
**Sample Type**	Sample	**Position**	Vial 28
**Instrument Name**	US10310002	**User Name**	TOF-PC\admin
**Acq Method**	Bypass.m	**Acquired Time**	5/4/2021 1:50:20 PM
**IRM Calibration Status**	Success	**DA Method**	Damijana.m
**Comment**			

Compound Label	*m/z*	RT	Algorithm	Mass
Cpd 1: C31 H33 F6 N3 O2	594.2545	0.261	Find by Molecular Feature	593.2474

Compound Identification Results

Ion Mass	Calc Ion Mass	Difference	IonFormula	IonSpecies	Best
594.2545	594.255	0.4	C31 H34 F6 N3 O2	(M+H)+	✓
594.2545	594.2541	-0.4	C15 H34 F6 N11 O7	(M+H)+	
594.2545	594.2536	-0.9	C29 H32 F6 N6 O	(M+H)+	
594.2545	594.2555	0.9	C16 H30 F6 N15 O3	(M+H)+	
594.2545	594.2555	0.9	C17 H36 F6 N8 O8	(M+H)+	
594.2545	594.2563	1.8	C33 H36 F6 O3	(M+H)+	
594.2545	594.2523	-2.2	C28 H36 F6 N2 O5	(M+H)+	
594.2545	594.2523	-2.2	C27 H30 F6 N9	(M+H)+	
594.2545	594.2568	2.3	C18 H32 F6 N12 O4	(M+H)+	
594.2545	594.2568	2.3	C19 H38 F6 N5 O9	(M+H)+	

MS Spectrum

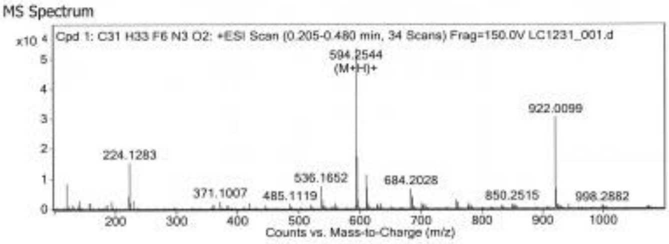

MFE MS Zoomed Spectrum

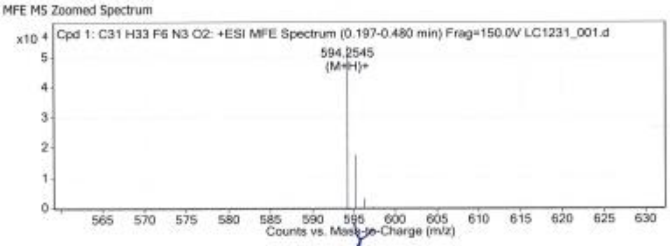

--- End Of Report ---

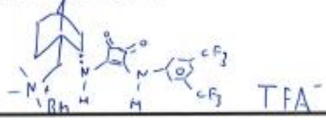

1-benzyl-1-(((1*S*,2*S*,4*R*)-7,7-dimethyl-2-(3-phenylthioureido)bicyclo[2.2.1]heptan-1-yl)methyl)pyrrolidin-1-ium iodide (X)

Qualitative Compound Report

neoznačen vzorec

**Data File**	LC1210_001.d	**Sample Name**	LC1210
**Sample Type**	Sample	**Position**	Vial 34
**Instrument Name**	US10310002	**User Name**	TOF-PC\admin
**Acq Method**	Bypass.m	**Acquired Time**	12/8/2022 9:52:38 AM
**IRM Calibration Status**	Success	**DA Method**	Damijana.m
**Comment**			

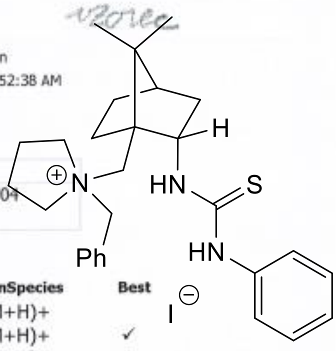

Compound Label	*m/z*	RT	Algorithm	Mass
Cpd 5: C28 H37 N3 S	448.2776	0.257	Find by Molecular Feature	447.2704

Compound Identification Results

Ion Mass	Calc Ion Mass	Difference	IonFormula	IonSpecies	Best
448.2776	448.2773	-0.3	C12 H38 N11 O5 S	(M+H)+	
448.2776	448.2781	0.5	C28 H38 N3 S	(M+H)+	✓
448.2776	448.2786	1	C13 H34 N15 O S	(M+H)+	
448.2776	448.2786	1	C14 H40 N8 O6 S	(M+H)+	
448.2776	448.2794	1.9	C30 H40 O S	(M+H)+	
448.2776	448.2754	-2.2	C25 H40 N2 O3 S	(M+H)+	
448.2776	448.2799	2.4	C15 H36 N12 O2 S	(M+H)+	

MS Spectrum

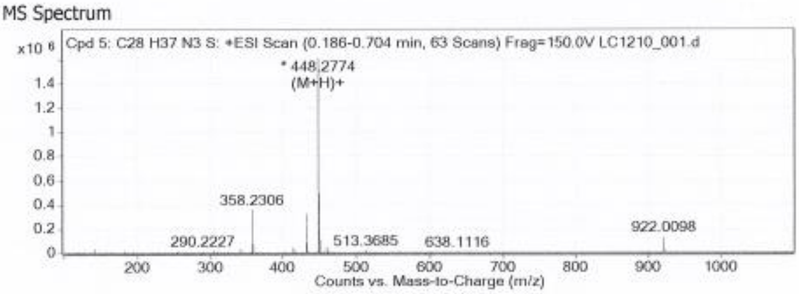

MFE MS Zoomed Spectrum

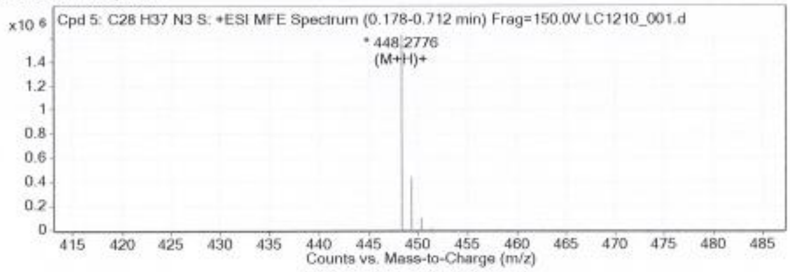

--- End Of Report ---

Agilent Technologies Page 1 of 1 Printed at: 9:55 AM on:12/8/2022

6. X-Ray crystallography

Table S8. Crystal data and structure refinement for compound **VI-Br**.

Empirical formula	$C_{26}H_{36}BrN_3S$
Formula weight	502.55
Temperature/K	149.9(3)
Crystal system	monoclinic
Space group	$P2_1$
a [Å^3]	12.4910(10)
b [Å^3]	9.1813(4)
c [Å^3]	12.5429(9)
α [°]	90
β [°]	118.740(9)
γ [°]	90
V [Å^3]	1261.26(17)
Z	2
ρ_{calc} [g/cm^3]	1.323
μ [mm^{-1}]	1.730
F(000)	528.0
Crystal size/mm^3	0.6 × 0.5 × 0.4
Radiation	MoKα (λ = 0.71073)
Reflections collected	11048
Independent reflections	6225
R_{int}	0.0295
Data/restraints/parameters	6225/0/284
GOF	1.052
R_1, wR_2 [I>=2σ (I)]	0.0506, 0.1378
R_1, wR_2 (all data)	0.0608, 0.1469
$(\Delta\rho)_{max}$ [e Å^{-3}]	0.64
$(\Delta\rho)_{min}$ [e Å^{-3}]	–1.11
Flack parameter	0.036(5)

Table S9. Crystal data and structure refinement for compound **III**.

Empirical formula	$C_{30}H_{36}F_6IN_3S$
Formula weight	711.58
Temperature/K	150.00(10)
Crystal system	orthorhombic
Space group	$P2_12_12_1$
a [Å^3]	10.8722(3)
b [Å^3]	14.7059(6)
c [Å^3]	19.2008(7)
α [°]	90
β [°]	90
γ [°]	90
V [Å^3]	3069.93(19)
Z	4
ρ_{calc} [g/cm^3]	1.540
μ [mm^{-1}]	1.172
F(000)	1440.0
Crystal size/mm^3	0.4 × 0.2 × 0.2
Radiation	MoKα (λ = 0.71073)
Reflections collected	29198
Independent reflections	8371
R_{int}	0.0386
Data/restraints/parameters	8371/0/382
GOF	1.035
R_1, wR_2 [I>=2σ (I)]	0.0443, 0.1029
R_1, wR_2 (all data)	0.0590, 0.1151
$(\Delta\rho)_{max}$ [e Å^{-3}]	0.97
$(\Delta\rho)_{min}$ [e Å^{-3}]	–1.40
Flack parameter	–0.031(8)

General Information

Single-crystal X-ray diffraction data was collected on Agilent Technologies SuperNova Dual diffractometer with an Atlas detector using monochromated Mo-K_{α} radiation ($\lambda = 0.71073$ Å) at 150 K. The data was processed using CrysAlis PRO[8]. Using Olex2.1.2.[9], the structures were solved by direct methods implemented in SHELXS[10] or SHELXT[11] and refined by a full-matrix least-squares procedure based on F^2 with SHELXT-2014/7[12]. All nonhydrogen atoms were refined anisotropically. Hydrogen atoms were placed in geometrically calculated positions and were refined using a riding model. The drawings and the analysis of bond lengths, angles and intermolecular interactions were carried out using Mercury[13] and Platon[14] (Figure S2 and S3). Structural and other crystallographic details on data collection and refinement for compounds **VI-Br** and **III** have been deposited with the Cambridge Crystallographic Data Centre as supplementary publication number CCDC Deposition Numbers **2204647** and **2204648**, respectively. These data can be obtained free of charge via www.ccdc.cam.ac.uk/conts/retrieving.html (or from the CCDC, 12 Union Road, Cambridge CB2 1EZ, UK; fax: +44 1223 336033; e-mail: deposit@ccdc.cam.ac.uk).

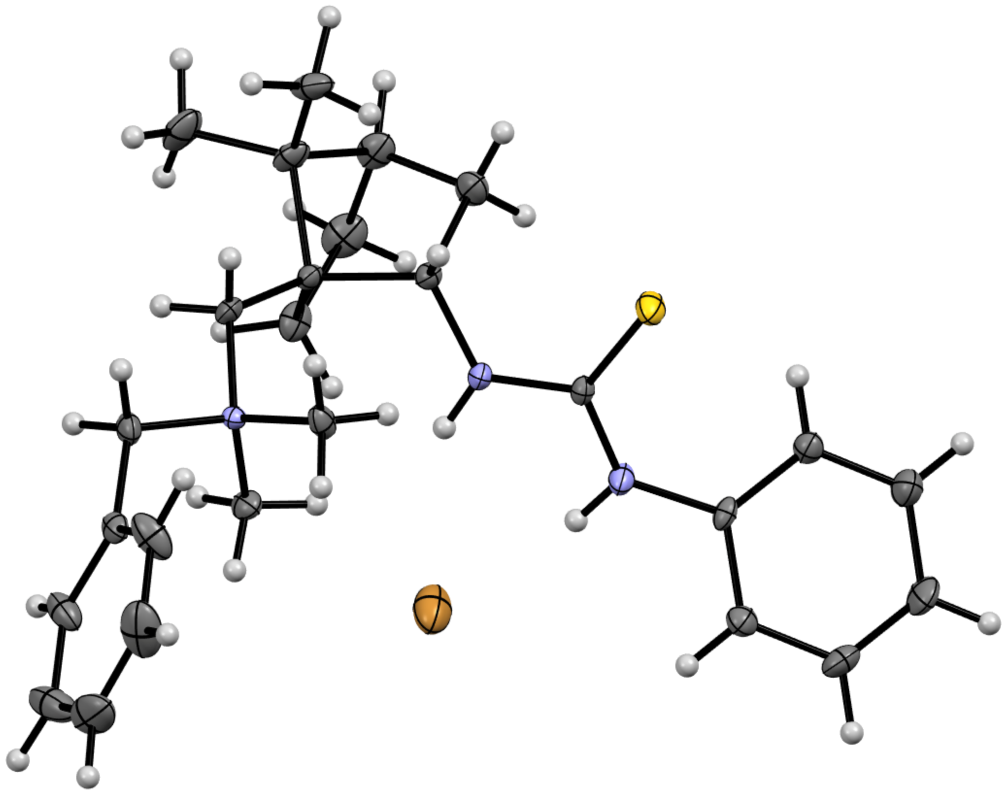

Figure S2. Molecular structure of compound **VI-Br**. Thermal ellipsoids are shown at 50% probability.

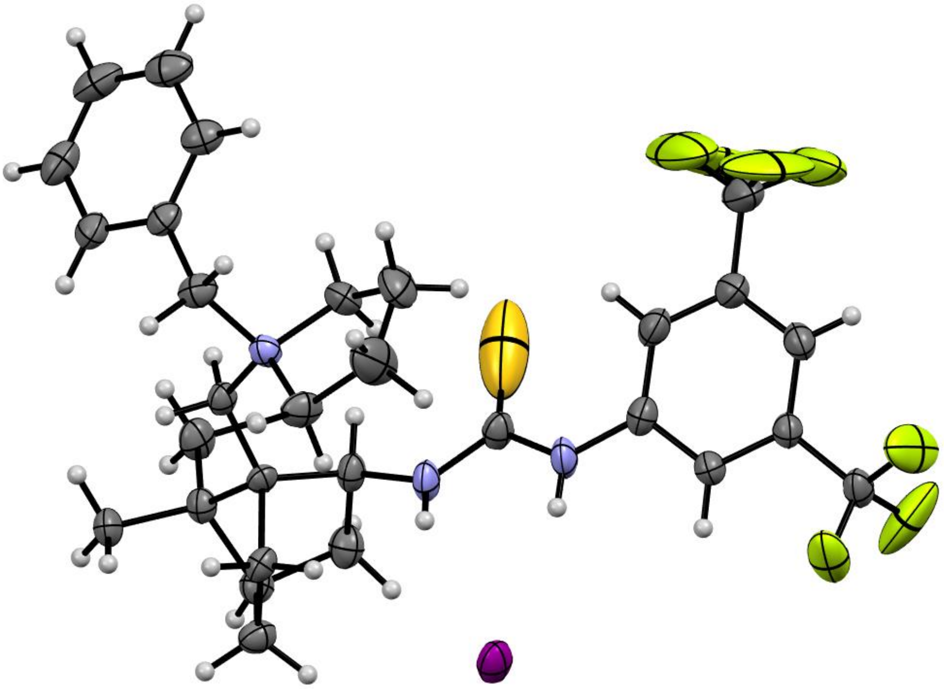

Figure S3. Molecular structure of compound **III**. Thermal ellipsoids are shown at 50% probability.

7. References

[1] Ričko, S.; Svete, J.; Štefane, B.; Perdih, A.; Golobič, A.; Meden, A.; Grošelj, U. 1,3-Diamine-Derived Bifunctional Organocatalyst Prepared from Camphor. *Adv. Synth. Catal.* **2016**, *358*, 3786–3796. https://doi.org/10.1002/adsc.201600498.

[2] Xu, J.; Hu, Y.; Huang, D.; Wang, K.-H.; Xu, C.; Niua T. Thiourea-Catalyzed Enantioselective Fluorination of *β*-Keto Esters. *Adv. Synth. Catal.* **2012**, *354*, 515–526. https://doi.org/10.1002/adsc.201100660.

[3] Novacek, J.; Waser, M. Syntheses and Applications of (Thio)Urea-Containing Chiral Quaternary Ammonium Salt Catalysts. *Eur. J. Org. Chem.* **2013**, *4*, 802–809. https://doi.org/10.1002/ejoc.201301594.

[4] Novacek, J.; Monkowius, U.; Himmelsbach, M.; Waser, M. Asymmetric α-chlorination of β-ketoesters using bifunctional ammonium salt catalysis. *Monatsh Chem.* **2016**, *147*, 533–538. https://doi.org/10.1007/s00706-015-1604-7.

[5] Mairhofer, C.; Novacek, J.; Waser, M. Synergistic Ammonium (Hypo)Iodite/Imine Catalysis for the Asymmetric α-Hydroxylation of β-Ketoesters. *Org. Lett.* **2020**, *22*, 15, 6138–6142. https://doi.org/10.1021/acs.orglett.0c02198.

[6] Haider, V.; Kreuzer, V.; Tiffner, M.; Spingler, B.; Waser, M. Ammonium Salt-Catalyzed Ring-Opening of Aryl-Aziridines with β-Keto Esters. *Eur. J. Org. Chem.* **2020**, *32*, 5173–5177. https://doi.org/10.1002/ejoc.202000916.

[7] Tiffner, M.; Novacek, J.; Busillo, A.; Gratzer, K.; Massa, A.; Waser, M. Design of chiral urea-quaternary ammonium salt hybrid catalysts for asymmetric reactions of glycine Schiff bases. *RSC Adv.* **2015**, *5*, 78941–78949. https://doi.org/10.1039/C5RA14466C.

[8] *CrysAlis PRO*, *Agilent Technologies UK Ltd*, Yarnton, Oxfordshire, England, **2011**.

[9] Dolomanov, O.V.; Bourhis, L.J.; Gildea, R.J.; Howard, J.A.K.; Puschmann, H. *OLEX2*: a complete structure solution, refinement and analysis program, *J. Appl. Cristallogr.* **2009**, *42*, 339–341. https://doi.org/10.1107/S0021889808042726.

[10] Sheldrick, G.M. A short history of *SHELX*, *Acta Crystallogr. A* **2008**, *64*, 112–122. https://doi.org/10.1107/S0108767307043930.

[11] Sheldrick, G.M. *SHELXT* - Integrated space-group and crystal-structure determination, *Acta Crystallogr. Sect. A Found. Adv.* **2015**, *71*, 3–8. https://doi.org/10.1107/S2053273314026370.

[12] Sheldrick, G.M. Crystal structure refinement with *SHELXL*, *Acta Crystallogr. Sect. C Struct. Chem.* **2015**, *71*, 3–8. https://doi.org/10.1107/S2053229614024218.

[13] Macrae, C.F.; Edgington, P.R.; McCabe, P.; Pidcock, E.; Shields, G.P.; Taylor, R.; Towler, M.; van de Streek, J. Synthesis, *Mercury*: visualization and analysis of crystal structures. *J. Appl. Crystallogr.* **2006**, *39*, 453–457. https://doi.org/10.1107/S002188980600731X.

[14] Spek, A.L. Single-crystal structure validation with the program PLATON, *J. Appl. Crystallogr*. **2003**, *36*, 7–13. https://doi.org/10.1107/S0021889802022112.
